# Supplementary material for: Iterative SuFEx approach for sequence-regulated oligosulfates and its extension to periodic copolymers
Source: Nat Commun. 2024 Apr 20;15:3381. doi: 10.1038/s41467-024-47567-z (PMC11032359; doi:10.1038/s41467-024-47567-z)
Supplement: Supplementary file 1 — Supplementary Information [file 41467_2024_47567_MOESM1_ESM.pdf]

Supplementary Information:

Iterative SuFEx approach for sequence-regulated oligosulfates and its extension to periodic copolymers

Min Pyeong Kim, Swatilekha Kayal, Chiwon Hwang, Jonghoon Bae, Hyunseok Kim, Dong Gyu Hwang, Min Ho Jeon, Jeong Kon Seo, Dowon Ahn, Wonjoo Lee, Sangwon Seo, Joong-Hyun Chun,* Youngchang Yu* and Sung You Hong*

*E-mails: jchun@yuhs.ac (J.-H.C.), ycyu@krict.re.kr (Y.Y.), syhong@unist.ac.kr (S.Y.H.)

Table of Contents

1. General Information 2
1.1. Characterisation 2
1.2. Basic Building Blocks Structures 3
2. Orthogonality Evaluations 4
2.1. Compatibility of SuFEx Coupling Reaction and Imidazylate Moieties 4
2.2. Optimisation of Nucleophilic Fluorination Preserving Sulfate Linkage 4
3. Synthesis of Building Blocks 4
4. Synthesis of Sequence-Regulated Oligosulfates 10
4.1. Uni-directional Synthesis 10
4.2. Bi-directional Synthesis 22
4.3. Tri-directional Synthesis 26
5. Sequencing of Oligosulfates After Base Hydrolysis 27
5.1. Degradation Test on Oligosulfate 27
5.2. HRMS (ESI) Analysis for the Fragments of Sequence-Regulated Oligomers 28
5.2.1. Fragments from 11 28
5.2.2. Fragments from 30 30
6. Synthesis of Polysulfate Periodic Copolymers 31
7. Hydrolysis of Polysulfates Periodic Copolymer and Analysis of Fragments 33
7.1. GPC Chromatograms of Hydrolysed Polysulfates Compared to Pristine Polymer 33
7.2. HRMS (ESI) Analysis for the Fragments from P-2 34
7.3. Degradation Test on P-2 36
8. Preliminary Adhesion Test 37
8.1. Adhesion Test of Linear Polysulfates 37
8.2. Adhesion Test of P-4 & P-5 37
9. Iterative Synthesis through Protecting Group Approach 38
10. NMR Spectra 43
11. References 128

1. General Information

1.1. Characterisation

Nuclear magnetic resonance (NMR) spectra were recorded on a Bruker Avance III HD 400 in CD_3CN, CD_3Cl, CD_2Cl_2, and DMSO-d_6 and the chemical shift was quoted in ppm. The following abbreviations were used to indicate multiplicities: s = singlet, d = doublet, t = triplet, q = quartet, and m = multiplet. Coupling constants (*J*) was expressed in hertz (Hz). Residual solvent peaks were referenced for the chemical shift for ^{1}H NMR and ^{13}C NMR ($CDCl_3$: 7.26 / 77.16 ppm, CD_3CN : 1.94 / 1.32 ppm, CD_2Cl_2 : 5.32 / 53.84 ppm, DMSO-d_6 : 2.50 / 39.52 ppm, and Acetone-d_6: 2.05 / 29.84 ppm). ^{19}F spectra were referenced to trifluorotoluene (-63.72 ppm).

Reactions were monitored by using thin layer chromatography (TLC) by using Merck silica gel 60 F_{254} glass plates. And the plates were visualized by UV light or $KMnO_4$ staining solution.

High resolution mass spectrometry (HRMS) data were obtained using a Q Exactive™ Plus Hybrid Quadrupole-Orbitrap™ mass spectrometer from Thermo Scientific in electrospray ionization (ESI) mode, an AccuTOF 4G+ DART from JEOL in direct analysis in real time (DART) mode, or a JMS-700 GC-HRMS from JEOL in electron ionization (EI) mode.

Melting points were measured by using a digital melting point (mp) apparatus SMP-10 from Stuart (Stone, UK) and were uncorrected.

For the detailed gel permeation chromatography (GPC) analysis, see the below.
(One-column system) GPC analysis was performed using an Agilent 1200 infinity series equipped with a Waters Styragel HR-3 column for the GPC trace monitoring. The system was calibrated with polystyrene calibration kit from Agilent Technologies. DMF with 0.05 M LiBr was used as a mobile phase with an eluent flow rate of 1.0 mL/min and a column temperature of 40 ˚C.
(Three-column system) GPC analysis was conducted using a Jasco LC-4000 Series equipped with three polystyrene-gel columns in series (Shodex; KD-802, KD-803, and KD-804), along with guard column. This method was used for the M_n and $Đ$ values. Reflex index detector (RI-4030) and UV detector (UV-4075) were employed. DMF with 0.05 M LiBr was used as a mobile phase with an eluent flow rate of 0.2 mL/min and a column temperature of 45 ˚C. Results were calibrated by Shodex polystyrene standard (M_p = 1230–2400000; $Đ$ = 1.02–1.08).

1.2. Basic Building Blocks Structures

Supplementary Table 1. Structures of Basic Building Blocks

Entry	Building Blocks	Entry	Building Blocks
1	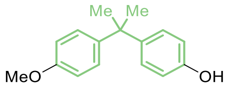 **S-1**	10	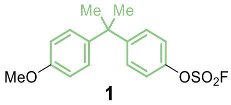 **1**
2	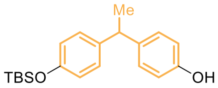 **S-2**	11	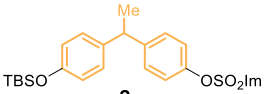 **2**
3	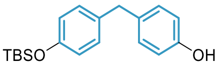 **S-3**	12	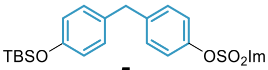 **5**
4	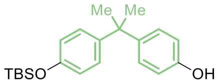 **S-4**	13	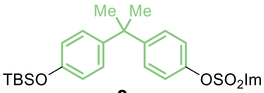 **8**
5	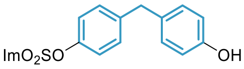 **S-5**	14	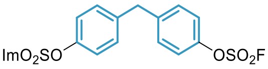 **37**
6	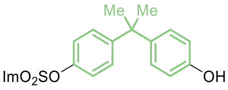 **S-6**	15	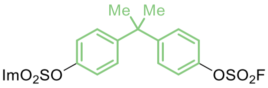 **S-7**
7	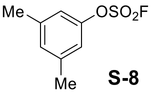 **S-8**	16	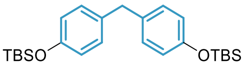 **50**
8	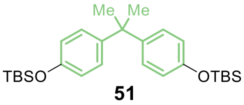 **51**	17	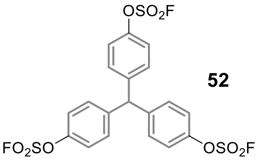 **52**
9	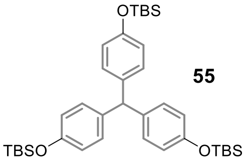 **55**	-	-

2. Orthogonality Evaluations

2.1. Compatibility of SuFEx Coupling Reaction and Imidazylate Moieties

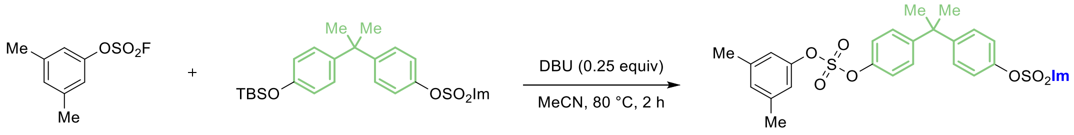

S-8 **8** **S-9**, 95%

1.0 equiv 1.0 equiv

See also Section 4.1 for the detailed synthetic procedure for **S-9**.

2.2. Optimisation of Nucleophilic Fluorination Preserving Sulfate Linkage

Supplementary Table 2. Optimisation of Nucleophilic Fluorination

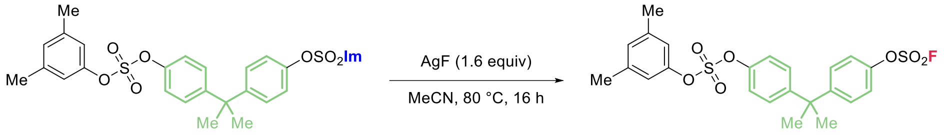

S-9 **S-10**

Entry	Changes from standard conditions[a]	Yield (%)
**1**	**None**	**88**
2	AgF (1.4 equiv)	85
3	AgF (1.8 equiv)	85
4	AgF (2.0 equiv)	75
5	MeCN/*tert*-BuOH(95:5, v/v) instead of MeCN	85
6	MeCN/*tert*-amyl alcohol(95:5, v/v) instead of MeCN	87
7	MeCN/Dimethyl carbonate(95:5, v/v) instead of MeCN	83
8	MeCN/diglyme(95:5, v/v) instead of MeCN	74

[a]Standard conditions: **S-9** (0.40 mmol, 1.0 equiv), AgF (0.64 mmol, 1.6 equiv) in acetonitrile (MeCN) (1.3 mL) for 16 h at 80 ˚C. Isolated yield.

3. Synthesis of Building Blocks

General Procedure A. Bisphenol substrate was treated with iodomethane and potassium carbonate in DMF. After stirring for 16 h at room temperature (rt), the mixture was diluted with ethyl acetate and washed with brine 3 times. Then, organic phase was dried over Na_2SO_4, concentrated *in vacuo*, and purified by flash column chromatography to afford the corresponding bisphenol derivative.

General Procedure B. *tert*-Butyldimethylsilyl chloride (TBSCl) was added to a mixture of bisphenol substrate, and imidazole in tetrahydrofuran (THF). After stirring for 16 h at rt, the mixture was concentrated *in vacuo*, and purified by flash column chromatography to afford the corresponding TBS-substituted bisphenol.

General Procedure C. 1,1′-Sulfonyldiimidazole (SDI) was added to a mixture of bisphenol substrate and cesium carbonate in THF. After stirring for 16 h at rt, the mixture was purified by flash column chromatography to afford the corresponding imidazylate derivative.

General Procedure D. *Ex situ* generated SO_2F_2 gas from the reaction between SDI and potassium fluoride in the presence of trifluoroacetic acid (TFA, *ca.* 1-1.5 mL) at rt in Flask A was transferred through still head adapter to a suspension/solution of phenolic substrate (1 equiv) with triethylamine (Et_3N) in dichloromethane (DCM) in Flask B. After stirring for 16 h at rt, the mixture was purified by flash column chromatography to afford the corresponding aryl fluorosulfate.

*4-[1-(4-Methoxyphenyl)-1-methylethyl]phenol (**S-1**)*[1]

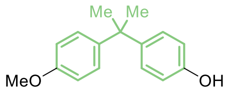

The title compound was prepared according to General Procedure A using 2,2-bis(4-hydroxyphenyl)propane (9.13 g, 40.0 mmol, 1.0 equiv), iodomethane (2.24 mL, 36.0 mmol, 0.9 equiv), and potassium carbonate (8.29 g, 60.0 mmol, 1.5 equiv) in DMF (80 mL). After stirring for 16 h at rt, the mixture was diluted with ethyl acetate (800 mL) and washed with brine 3 times. Then, the organic phase was dried over Na_2SO_4, concentrated *in vacuo*, and purified by flash column chromatography(hexanes : ethyl acetate = 5 : 1 to 3 : 1) to afford a colorless oil (4.81 g, 50%). ^1H NMR (400 MHz, $CDCl_3$) δ 7.18 – 7.13 (m, 2H), 7.13 – 7.08 (m, 2H), 6.85 – 6.79 (m, 2H), 6.76 – 6.71 (m, 2H), 4.79 (s, 1H), 3.80 (s, 3H), 1.64 (s, 6H).

*4-[1-[4-[[(1,1-Dimethylethyl)dimethylsilyl]oxy]phenyl]ethyl]phenol (**S-2**)*[2]

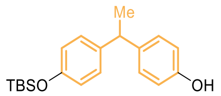

The title compound was prepared according to General Procedure B using 4,4′-ethylidenebisphenol (8.57 g, 40.0 mmol, 1.0 equiv), TBSCl (6.03 g, 40.0 mmol, 1.0 equiv), and imidazole (3.27 g, 48.0 mmol, 1.2 equiv) in THF (130 mL). After stirring for 16 h at rt, the mixture was purified by flash column chromatography (hexanes : ethyl acetate = 8 : 1) to afford a colorless oil (7.82 g, 60%). ^1H NMR (400 MHz, DMSO-d_6) δ 9.15 (s, 1H), 7.16 – 6.90 (m, 4H), 6.81 – 6.54 (m, 4H), 4.05 – 3.85 (m, 1H), 1.52 – 1.41 (m, 3H), 0.93 (s, 9H), 0.15 (s, 6H).

*4-[[4-[[(1,1-Dimethylethyl)dimethylsilyl]oxy]phenyl]methyl]phenol (**S-3**)*[3]

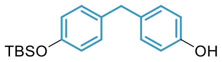

The title compound was prepared according to General Procedure B using 4,4′-dihydroxydiphenylmethane (8.01 g, 40.0 mmol, 1.0 equiv), TBSCl (6.03 g, 40.0 mmol, 1.0 equiv), and imidazole (3.27 g, 48.0 mmol, 1.2 equiv) in THF (130 mL). After stirring for 16 h at rt, the mixture was purified by flash column chromatography (hexanes :

ethyl acetate = 8 : 1) to afford a colorless oil (6.59 g, 52%). ^{1}H NMR (400 MHz, $CDCl_3$) δ 7.06 – 6.99 (m, 4H), 6.78 – 6.72 (m, 4H), 4.63 (s, 1H), 3.84 (s, 2H), 0.98 (s, 9H), 0.19 (s, 6H).

*4-[1-[4-[[(1,1-Dimethylethyl)dimethylsilyl]oxy]phenyl]-1-methylethyl]phenol (**S-4**)*[4]

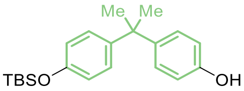

The title compound was prepared according to General Procedure B using 2,2-bis(4-hydroxyphenyl)propane (9.13 g, 40.0 mmol, 1.0 equiv), TBSCl (6.03 g, 40.0 mmol, 1.0 equiv), and imidazole (3.27 g, 48.0 mmol, 1.2 equiv) in THF (130 mL). After stirring for 16 h at rt, the mixture was purified by flash column chromatography (hexanes : ethyl acetate = 7 : 1) to afford a white solid (6.88 g, 50%). ^{1}H NMR (400 MHz, $CDCl_3$) δ 7.12 – 7.03 (m, 4H), 6.76 – 6.68 (m, 4H), 4.69 (s, 1H), 1.62 (s, 6H), 0.97 (s, 9H), 0.18 (s, 6H).

*4-(4-Hydroxybenzyl)phenyl 1H-imidazole-1-sulfonate (**S-5**)*

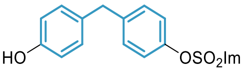

The title compound was prepared according to General Procedure C using 4,4′-dihydroxydiphenylmethane (2.00 g, 10.0 mmol, 1.0 equiv), SDI (1.98 g, 10.0 mmol, 1.0 equiv), and cesium carbonate (1.63 g, 5.00 mmol, 0.5 equiv) in THF (30 mL). After stirring for 16 h at rt, the mixture was purified by flash column chromatography (hexanes : ethyl acetate = 1 : 1) to afford a colorless oil (632 mg, 19%). ^{1}H NMR (400 MHz, CD_3CN) δ 7.81 – 7.77 (m, 1H), 7.45 – 7.41 (m, 1H), 7.24 – 7.19 (m, 2H), 7.16 – 7.13 (m, 1H), 7.05 – 6.97 (m, 2H), 6.93 – 6.88 (m, 3H), 6.76 – 6.69 (m, 2H), 3.86 (s, 2H). ^{13}C NMR (101 MHz, CD_3CN) δ 156.4, 148.3, 144.2, 138.7, 132.8, 132.2, 131.4, 130.8, 122.1, 119.7, 116.3, 40.6. HRMS (ESI): *m/z* [M-H]$^-$ calcd for $C_{16}H_{13}N_2O_4S$ 329.0602; found 329.0606.

*4-(2-(4-Hydroxyphenyl)propan-2-yl)phenyl 1H-imidazole-1-sulfonate (**S-6**)*

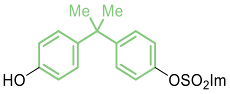

The title compound was prepared according to General Procedure C using 2,2-bis(4-hydroxyphenyl)propane (2.28 g, 10.0 mmol, 1.0 equiv), SDI (1.98 g, 10.0 mmol, 1.0 equiv), and cesium carbonate (1.63 g, 5.00 mmol, 0.5 equiv) in THF (30 mL). After stirring for 16 h at rt, the mixture was purified by flash column chromatography (hexanes : ethyl acetate = 3 : 2) to afford a white solid (1.42 g, 40%). mp: 64 – 66 ˚C. ^{1}H NMR (400 MHz, DMSO-d_6) δ 9.22 (s, 1H), 8.23 – 8.13 (m, 1H), 7.83 – 7.74 (m, 1H), 7.32 – 7.20 (m, 3H), 7.04 – 6.89 (m, 4H), 6.72 – 6.60 (m, 2H), 1.56 (s, 6H). ^{13}C NMR (101 MHz, DMSO-d_6) δ 155.2, 151.5, 146.4, 139.7, 138.1, 131.3, 128.5, 127.3, 120.4, 119.1, 114.8, 41.7, 30.5. HRMS (ESI): *m/z* [M-H]$^-$ calcd for $C_{18}H_{17}N_2O_4S$ 357.0915; found 357.0914.

*4-(2-(4-(((1H-imidazol-1-yl)sulfonyl)oxy)phenyl)propan-2-yl)phenyl fluorosulfate (**S-7**)*

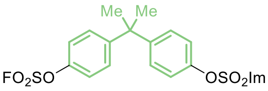

The title compound was prepared according to General Procedure D using SDI (1.95 g, 9.83 mmol, 2.5 equiv), potassium fluoride (1.44 g, 24.8 mmol, 6.3 equiv) in TFA (16 mL) for Flask A and **S-6** (1.41 g, 3.93 mmol, 1.0 equiv) and Et_3N (1.37 mL, 9.83 mmol, 2.6 equiv) in DCM (13 mL) for Flask B. After stirring for 16 h at rt, the mixture was purified by flash column chromatography (hexanes : ethyl acetate = 2 : 1) to afford a colorless oil (1.57 g, 91%). ^1H NMR (400 MHz, CD_3CN) δ 7.82 – 7.78 (m, 1H), 7.45 – 7.42 (m, 1H), 7.35 (s, 4H), 7.31 – 7.24 (m, 2H), 7.15 – 7.12 (m, 1H), 6.95 – 6.90 (m, 2H), 1.66 (s, 6H). ^{13}C NMR (101 MHz, CD_3CN) δ 152.1, 151.6, 149.2, 148.2, 138.7, 132.2, 130.0, 129.7, 121.9, 121.5, 119.7, 43.7, 30.7. ^{19}F NMR (376 MHz, CD_3CN) δ 36.0. HRMS (ESI): *m/z* $[M+Na]^+$ calcd for $C_{18}H_{17}FN_2NaO_6S_2$ 463.0404; found 463.0403.

*3,5-Dimethylphenyl fluorosulfate (**S-8**)*[5]

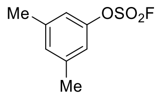

The title compound was prepared according to General Procedure D using SDI (5.00 g, 2.52 mmol, 2.5 equiv), potassium fluoride (3.67 g, 63.2 mmol, 6.3 equiv) in TFA (10 mL) for Flask A and 3,5-dimethylphenol (1.22 g, 10.0 mmol, 1.0 equiv) and Et_3N (3.48 mL, 25.0 mmol, 2.5 equiv) in DCM (20 mL) for Flask B. After stirring for 16 h at rt, the mixture was purified by flash column chromatography (hexanes : ethyl acetate = 8 : 1) to afford a white solid (1.94 g, 95%). ^1H NMR (400 MHz, $CDCl_3$) δ 7.34 – 7.29 (m, 2H), 7.24 – 7.18 (m, 2H), 7.16 – 7.10 (m, 2H), 6.86 – 6.80 (m, 2H), 3.80 (s, 3H), 1.67 (s, 6H). ^{19}F NMR (376 MHz, $CDCl_3$) δ 36.5.

*4-[1-(4-Methoxyphenyl)-1-methylethyl]phenol (**1**)*

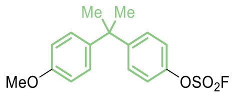

The title compound was prepared according to General Procedure D using SDI (9.02 g, 45.5 mmol, 2.5 equiv), potassium fluoride (6.68 g, 115 mmol, 6.3 equiv) in TFA (20 mL) for Flask A and **S-1** (4.41 g, 18.1 mmol, 1.0 equiv) and Et_3N (6.34 mL, 45.5 mmol, 2.5 equiv) in DCM (60 mL) for Flask B. After stirring for 16 h at rt, the mixture was purified by flash column chromatography (hexanes : ethyl acetate = 8 : 1) to afford a colorless oil (4.75 g, 81%). ^1H NMR (400 MHz, $CDCl_3$) δ 7.34 – 7.29 (m, 2H), 7.24 – 7.18 (m, 2H), 7.16 – 7.10 (m, 2H), 6.86 – 6.80 (m, 2H), 3.80 (s, 3H), 1.67 (s, 6H). ^{13}C NMR (101 MHz, $CDCl_3$) δ 157.9, 152.1, 148.1, 141.8, 128.9, 127.8, 120.3, 113.7, 55.4, 42.4, 31.0. ^{19}F NMR (376 MHz, $CDCl_3$) δ 36.3. HRMS (EI): *m/z* $[M]^+$ calcd for $C_{16}H_{17}FO_4S$ 324.0832; found 324.0837.

*4-(1-(4-((tert-Butyldimethylsilyl)oxy)phenyl)ethyl)phenyl 1H-imidazole-1-sulfonate (**2**)*

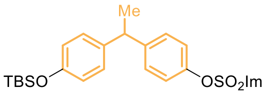

The title compound was prepared according to General Procedure C using **S-2** (7.68 g, 23.4 mmol, 1.0 equiv), SDI (6.96 g, 35.1 mmol, 1.5 equiv), and cesium carbonate (3.81 g, 11.7 mmol, 0.5 equiv) in THF (80 mL). After stirring for 16 h at rt, the mixture was purified by flash column chromatography (hexanes : ethyl acetate = 6 : 1 to 2 : 1) to afford a white solid (5.85 g, 54%). mp: 46 – 48 ˚C. ^1H NMR (400 MHz, $CDCl_3$) δ 7.75 – 7.71 (m, 1H), 7.31 – 7.28

(m, 1H), 7.19 – 7.14 (m, 3H), 7.02 – 6.96 (m, 2H), 6.83 – 6.73 (m, 4H), 4.07 (q, *J* = 7.2 Hz, 1H), 1.56 (d, *J* = 7.2 Hz, 3H), 0.97 (s, 9H), 0.18 (s, 6H). ^{13}C NMR (101 MHz, $CDCl_3$) δ 154.2, 147.8, 147.2, 137.9, 137.7, 131.4, 129.5, 128.5, 121.1, 120.1, 118.4, 43.6, 25.8, 22.2, 18.3, -4.3. HRMS (ESI): *m/z* $[M+H]^+$ calcd for $C_{23}H_{31}N_2O_4SSi$ 459.1768; found 459.1761.

*4-(4-((tert-Butyldimethylsilyl)oxy)benzyl)phenyl 1H-imidazole-1-sulfonate (**5**)*

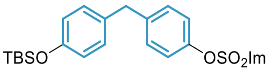

The title compound was prepared according to General Procedure C using **S-3** (6.41 g, 20.4 mmol, 1.0 equiv), SDI (6.06 g, 30.6 mmol, 1.5 equiv), and cesium carbonate (3.32 g, 10.2 mmol, 0.5 equiv) in THF (80 mL). After stirring for 16 h at rt, the mixture was purified by flash column chromatography (hexanes : ethyl acetate = 6 : 1 to 2 : 1) to afford a white solid (4.43 g, 49%). mp: 42 – 43 ˚C. ^{1}H NMR (400 MHz, $CDCl_3$) δ 7.75 – 7.68 (m, 1H), 7.30 – 7.28 (m, 1H), 7.17 – 7.15 (m, 1H), 7.15 – 7.10 (m, 2H), 7.01 – 6.94 (m, 2H), 6.84 – 6.79 (m, 2H), 6.79 – 6.74 (m, 2H), 3.89 (s, 2H), 0.98 (s, 9H), 0.19 (s, 6H). ^{13}C NMR (101 MHz, $CDCl_3$) δ 154.4, 147.4, 142.6, 137.7, 132.5, 131.4, 130.6, 130.0, 121.3, 120.3, 118.4, 40.5, 25.8, 18.3, -4.3. HRMS (ESI): *m/z* $[M+H]^+$ calcd for $C_{22}H_{29}N_2O_4SSi$ 445.1612; found 445.1614.

*4-(2-(4-((tert-Butyldimethylsilyl)oxy)phenyl)propan-2-yl)phenyl 1H-imidazole-1-sulfonate (**8**)*

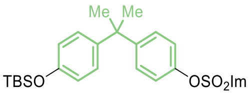

The title compound was prepared according to General Procedure C using **S-4** (7.00 g, 20.4 mmol, 1.0 equiv), SDI (6.03 g, 30.4 mmol, 1.5 equiv), and cesium carbonate (3.32 g, 10.2 mmol, 0.5 equiv) in THF (70 mL). After stirring for 16 h at rt, the mixture was purified by flash column chromatography (hexanes : ethyl acetate = 7 : 1) to afford a white solid (4.55 g, 48%). mp: 98 – 101 ˚C. ^{1}H NMR (400 MHz, $CDCl_3$) δ 7.76 – 7.72 (m, 1H), 7.31 – 7.29 (m, 1H), 7.22 – 7.15 (m, 3H), 7.03 – 6.97 (m, 2H), 6.82 – 6.71 (m, 4H), 1.61 (s, 6H), 0.97 (s, 9H), 0.19 (s, 6H). ^{13}C NMR (101 MHz, $CDCl_3$) δ 153.8, 152.1, 146.9, 142.2, 137.6, 131.3, 128.8, 127.7, 120.7, 119.6, 118.4, 42.4, 31.0, 25.8, 18.3, -4.3. HRMS (ESI): *m/z* $[M+H]^+$ calcd for $C_{24}H_{33}N_2O_4SSi$ 473.1925; found 473.1925.

*4,4'-(Propane-2,2-diyl)bis(4,1-phenylene) difluorosulfate (**36**)*[6]

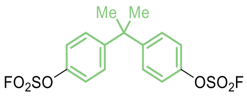

The title compound was prepared according to General Procedure D using SDI (8.92 g, 45.0 mmol, 5.0 equiv), potassium fluoride (6.59 g, 113 mmol, 12.6 equiv) in TFA (30 mL) for Flask A and 2,2-bis(4-hydroxyphenyl)propane (2.05 g, 9.00 mmol, 1.0 equiv) and Et_3N (3.26 mL, 23.4 mmol, 2.6 equiv) in DCM (45 mL) for Flask B. After stirring for 16 h at rt, the mixture was purified by flash column chromatography (hexanes : ethyl acetate = 4 : 1) to afford a white solid (3.41 g, 97%). ^{1}H NMR (400 MHz, $CDCl_3$) δ 7.33 – 7.23 (m, 8H), 1.70 (s, 6H). ^{19}F NMR (376 MHz, $CDCl_3$) δ 36.5.

*4-(4-(((1H-imidazol-1-yl)sulfonyl)oxy)benzyl)phenyl fluorosulfate (**37**)*

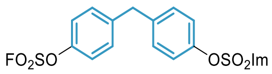

The title compound was prepared according to General Procedure D using SDI (1.19 g, 5.98 mmol, 2.5 equiv), potassium fluoride (877 mg, 15.1 mmol, 6.3 equiv) in TFA (10 mL) for Flask A and **S-5** (987 mg, 2.99 mmol, 1.0 equiv) and Et_3N (833 µL, 5.98 mmol, 2.5 equiv) in DCM (8 mL) for Flask B. After stirring for 16 h at rt, the mixture was purified by flash column chromatography (hexanes : ethyl acetate = 3 : 2) to afford a white solid (1.18 g, 96%). mp: 85 – 86 ˚C. ^{1}H NMR (400 MHz, CD_3CN) δ 7.79 (s, 1H), 7.46 – 7.42 (m, 1H), 7.41 – 7.32 (m, 4H), 7.30 – 7.23 (m, 2H), 7.16 – 7.12 (m, 1H), 6.97 – 6.91 (m, 2H), 4.03 (s, 2H). ^{13}C NMR (101 MHz, CD_3CN) δ 149.6, 148.6, 143.1, 142.6, 138.7, 132.3, 131.9, 131.7, 122.4, 122.0, 119.7, 40.6. ^{19}F NMR (376 MHz, CD_3CN) δ 36.0. HRMS (ESI): *m/z* $[M+Na]^+$ calcd for $C_{16}H_{13}FN_2NaO_6S_2$ 435.0091; found 435.0090.

*Bis(4-((tert-butyldimethylsilyl)oxy)phenyl)methane (**50**)*

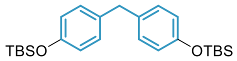

The title compound was prepared according to General Procedure B using 4,4′-dihydroxydiphenylmethane (801 mg, 4.00 mmol, 1.0 equiv), TBSCl (1.45 g, 9.60 mmol, 2.4 equiv), and imidazole (708 mg, 10.4 mmol, 2.6 equiv) in DCM (14 mL). After stirring for 16 h at rt, the mixture was purified by flash column chromatography (hexanes : ethyl acetate = 20 : 1) to afford a colorless oil (1.70 g, 99%). ^{1}H NMR (400 MHz, CD_3CN) δ 7.10 – 7.03 (m, 4H), 6.81 – 6.74 (m, 4H), 3.81 (s, 2H), 0.97 (s, 18H), 0.17 (s, 12H). ^{13}C NMR (101 MHz, CD_3CN) δ 154.8, 135.8, 130.6, 120.9, 40.7, 26.0, 18.8, -4.3. HRMS (ESI): *m/z* $[M+Na]^+$ calcd for $C_{25}H_{40}NaO_2Si_2$ 451.2459; found 451.2461.

*4,4'-(Propane-2,2-diyl)bis(4,1-phenylene))bis(oxy)bis(tert-butyldimethylsilane) (**51**)*[6]

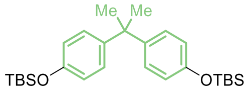

The title compound was prepared according to General Procedure B using 2,2-bis(4-hydroxyphenyl)propane (2.28 g, 10.0 mmol, 1.0 equiv), TBSCl (3.62 g, 24.0 mmol, 2.4 equiv), and imidazole (1.77 g, 26.0 mmol, 2.6 equiv) in DCM (33 mL). After stirring for 16 h at rt, the mixture was purified by flash column chromatography (hexanes : ethyl acetate = 20 : 1) to afford a white solid (4.49 g, 98%). ^{1}H NMR (400 MHz, $CDCl_3$) δ 7.10 – 7.01 (m, 4H), 6.75 – 6.68 (m, 4H), 1.61 (s, 6H), 0.97 (s, 18H), 0.19 (s, 12H).

*Methanetriyltris(benzene-4,1-diyl) tris(fluorosulfate) (**52**)*

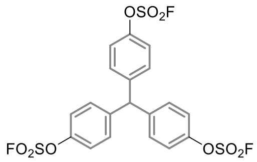

The title compound was prepared according to General Procedure D using SDI (8.92 g, 45.0 mmol, 7.5 equiv), potassium fluoride (6.57 g, 113 mmol, 18.9 equiv) in TFA (24 mL) for Flask A and 4,4′,4′′-trihydroxytriphenylmethane (1.75 g, 6.00 mmol, 1.0 equiv) and Et_3N (3.35 mL, 24.0 mmol, 4.0 equiv) in DCM (25

mL) for Flask B. After stirring for 16 h at rt, the mixture was purified by flash column chromatography (hexanes : ethyl acetate = 4 : 1) to afford a yellowish oil (3.18 g, 98%). mp: 121 – 124 ˚C. ^{1}H NMR (400 MHz, $CDCl_3$) *δ* 7.36 – 7.30 (m, 6H), 7.22 – 7.16 (m, 6H), 5.66 (s, 1H). ^{13}C NMR (101 MHz, $CDCl_3$) δ 149.1, 142.9, 131.3, 121.5, 54.9. ^{19}F NMR (376 MHz, $CDCl_3$) *δ* 36.8. HRMS (ESI): *m/z* $[M+Cl]^-$ calcd for $C_{19}H_{13}ClF_3O_9S_3$ 572.9368; found 572.9363.

*Tris(4-((tert-butyldimethylsilyl)oxy)phenyl)methane (**55**)*

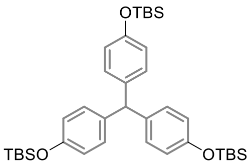

The title compound was prepared according to General Procedure B using 4,4′,4″-trihydroxytriphenylmethane (2.92 g, 10.0 mmol, 1.0 equiv), TBSCl (5.43 g, 36.0 mmol, 3.6 equiv), and imidazole (2.66 g, 39.0 mmol, 3.9 equiv) in DCM (35 mL). After stirring for 16 h at rt, the mixture was concentrated *in vacuo*, diluted with ethyl acetate, washed by $NaHCO_3$ and brine. The organic phase was dried over Na_2SO_4. Then, the product was dried overnight inside a vacuum oven at 90 ˚C to afford an apricot solid (6.13 g, 97%). mp: 109 – 111 ˚C. ^{1}H NMR (400 MHz, $CDCl_3$) *δ* 6.97 – 6.89 (m, 6H), 6.77 – 6.70 (m, 6H), 5.36 (s, 1H), 0.98 (s, 27H), 0.19 (s, 18H). ^{13}C NMR (101 MHz, $CDCl_3$) *δ* 153.9, 137.5, 130.3, 119.7, 54.7, 25.8, 18.3, -4.2. HRMS (ESI): *m/z* $[M+Na]^+$ calcd for $C_{37}H_{58}NaO_3Si_3$ 657.3586; found 657.3645.

4. Synthesis of Sequence-Regulated Oligosulfates

General Procedure E. A mixture of aryl fluorosulfate, *tert*-butyldimethylsilyl ether with imidazylate moiety, and 1,8-diazabicyclo[5.4.0]undec-7-ene (DBU) in anhydrous MeCN was stirred for 2 h at 80 ˚C in an Ace pressure tube. Then, the mixture was concentrated *in vacuo*, and purified by flash column chromatography to afford the corresponding oligosulfate bearing imidazylate functional group.

General Procedure F. A mixture of imidazylate and silver(I) fluoride (AgF) in anhydrous MeCN was stirred for 16 h at 80 ˚C in an Ace pressure tube. Then, the mixture was concentrated *in vacuo*, and purified by flash column chromatography to afford the corresponding oligosulfate bearing fluorosulfate functional group.

4.1. Uni-directional Synthesis

*4-(2-(4-(((3,5-Dimethylphenoxy)sulfonyl)oxy)phenyl)propan-2-yl)phenyl 1H-imidazole-1-sulfonate (**S-9**)*

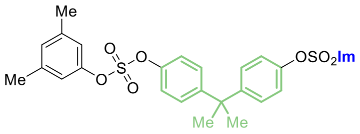

The title compound was prepared according to General Procedure E using **S-8** (457 mg, 2.24 mmol, 1.0 equiv), **8** (1.06 g, 2.24 mmol, 1.0 equiv), and DBU (84 μL, 0.56 mmol, 0.25 equiv) in anhydrous MeCN (7 mL). After stirring for 2 h at 80 ˚C, the mixture was purified by flash column chromatography (hexanes : ethyl acetate = 2 : 1) to afford

a colorless oil (1.15 g, 95%). ^{1}H NMR (400 MHz, CD_3CN) δ 7.82 – 7.78 (m, 1H), 7.45 – 7.41 (m, 1H), 7.33 – 7.24 (m, 6H), 7.14 (m, 1H), 7.08 – 7.04 (m, 1H), 6.99 – 6.96 (m, 2H), 6.95 – 6.89 (m, 2H), 2.31 (s, 6H), 1.65 (s, 6H). ^{13}C NMR (101 MHz, CD_3CN) δ 151.8, 151.3, 150.9, 149.4, 148.1, 141.6, 138.7, 132.2, 130.3, 129.7, 129.6, 121.9, 121.7, 119.7, 119.3, 43.6, 30.8, 21.2. HRMS (ESI): *m/z* $[M+H]^+$ calcd for $C_{26}H_{27}N_2O_7S_2$ 543.1254; found 543.1251.

*4-(2-(4-(((3,5-Dimethylphenoxy)sulfonyl)oxy)phenyl)propan-2-yl)phenyl fluorosulfate (**S-10**)*

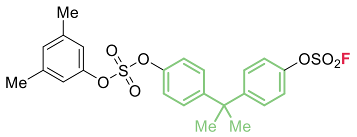

The title compound was prepared according to General Procedure F using **S-9** (217 mg, 0.40 mmol, 1.0 equiv) and AgF (81 mg, 0.65 mmol, 1.6 equiv) in anhydrous MeCN (1.3 mL). After stirring for 16 h at 80 ˚C, the mixture was purified by flash column chromatography (hexanes : ethyl acetate = 8 : 1) to afford a colorless oil (175 mg, 88%). ^{1}H NMR (400 MHz, CD_3CN) δ 7.45 – 7.26 (m, 8H), 7.06 (s, 1H), 6.97 (s, 2H), 2.31 (s, 6H), 1.70 (s, 6H). ^{13}C NMR (101 MHz, CD_3CN) δ 152.2, 151.3, 150.8, 149.5, 149.2, 141.6, 130.3, 130.0, 129.6, 121.8, 121.5, 119.3, 43.6, 30.8, 21.2. ^{19}F NMR (376 MHz, CD_3CN) δ 36.0. HRMS (ESI): *m/z* $[M+Na]^+$ calcd for $C_{23}H_{23}FNaO_7S_2$ 517.0761; found 517.0759.

*4-(1-(4-(((4-(2-(4-Methoxyphenyl)propan-2-yl)phenoxy)sulfonyl)oxy)phenyl)ethyl)phenyl 1H-imidazole-1-sulfonate (**3**)*

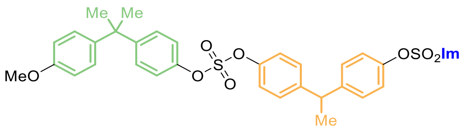

The title compound was prepared according to General Procedure E using **1** (700 mg, 2.16 mmol, 1.0 equiv), **2** (1.03 g, 2.25 mmol, 1.0 equiv), and DBU (81 µL, 0.55 mmol, 0.25 equiv) in anhydrous MeCN (7 mL). After stirring for 2 h at 80 ˚C, the mixture was purified by flash column chromatography (hexanes : ethyl acetate = 3 : 1 to 2 : 1) to afford a colorless oil (1.32 g, 94%). ^{1}H NMR (400 MHz, DMSO-d_6) δ 8.23 – 8.14 (m, 1H), 7.85 – 7.75 (m, 1H), 7.42 – 7.27 (m, 10H), 7.27 – 7.23 (m, 1H), 7.16 – 7.10 (m, 2H), 7.03 – 6.95 (m, 2H), 6.89 – 6.81 (m, 2H), 4.28 (q, J = 7.2 Hz, 1H), 3.71 (s, 3H), 1.62 (s, 6H), 1.55 (d, J = 7.2 Hz, 3H). ^{13}C NMR (101 MHz, DMSO-d_6) δ 157.2, 150.6, 148.2, 147.6, 146.9, 146.3, 145.6, 141.5, 138.1, 131.4, 129.4, 129.3, 128.4, 127.4, 121.1, 121.0, 120.4, 119.1, 113.5, 55.0, 42.7, 41.7, 30.4, 21.2. HRMS (ESI): *m/z* $[M+H]^+$ calcd for $C_{33}H_{33}N_2O_8S_2$ 649.1673; found 649.1671.

*4-(1-(4-(((4-(2-(4-Methoxyphenyl)propan-2-yl)phenoxy)sulfonyl)oxy)phenyl)ethyl)phenyl fluorosulfate (**4**)*

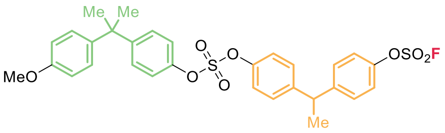

The title compound was prepared according to General Procedure F using **3** (1.18 g, 1.82 mmol, 1.0 equiv) and AgF (365 mg, 2.88 mmol, 1.6 equiv) in anhydrous MeCN (6 mL). After stirring for 16 h at 80 ˚C, the mixture was purified by flash column chromatography (hexanes : ethyl acetate = 3 : 1) to afford a colorless viscous oil (1.05 g,

96%). ^{1}H NMR (400 MHz, DMSO-d_6) δ 7.56 – 7.46 (m, 4H), 7.46 – 7.40 (m, 2H), 7.38 – 7.26 (m, 6H), 7.16 – 7.09 (m, 2H), 6.88 – 6.81 (m, 2H), 4.34 (q, J = 7.2 Hz, 1H), 3.71 (s, 3H), 1.64 – 1.56 (m, 9H). ^{13}C NMR (101 MHz, DMSO-d_6) δ 157.2, 150.6, 148.2, 148.0, 147.6, 146.9, 145.4, 141.5, 129.6, 129.3, 128.4, 127.4, 121.1, 121.1, 120.4, 113.5, 54.9, 42.7, 41.7, 30.4, 21.2. ^{19}F NMR (376 MHz, DMSO-d_6) δ 35.7. HRMS (ESI): m/z $[M+NH_4]^+$ calcd for $C_{30}H_{33}FNO_8S_2$ 618.1626; found 618.1626.

***{A-B-C}* Trimeric imidazylate (6)**

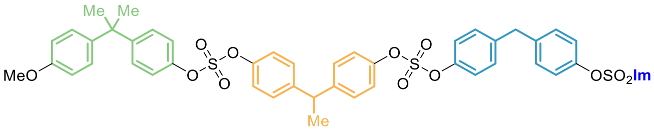

The title compound was prepared according to General Procedure E using **4** (913 mg, 1.52 mmol, 1.0 equiv), **5** (671 mg, 1.51 mmol, 1.0 equiv), and DBU (57 μL, 0.38 mmol, 0.25 equiv) in anhydrous MeCN (5 mL). After stirring for 2 h at 80 ˚C, the mixture was purified by flash column chromatography (hexanes : ethyl acetate = 3 : 1 to 2 : 1) to afford a colorless viscous oil (1.37 g, 99%). ^{1}H NMR (400 MHz, CD_3CN) δ 7.80 – 7.77 (m, 1H), 7.46 – 7.41 (m, 1H), 7.38 – 7.18 (m, 18H), 7.17 – 7.10 (m, 3H), 6.95 – 6.88 (m, 2H), 6.86 – 6.79 (m, 2H), 4.27 (q, J = 7.2 Hz, 1H), 3.98 (s, 2H), 3.73 (s, 3H), 1.67 – 1.56 (m, 9H). ^{13}C NMR (101 MHz, CD_3CN) δ 158.7, 152.1, 149.8, 149.8, 149.7, 149.7, 149.1, 148.5, 147.0, 146.9, 142.9, 142.8, 142.0, 141.8, 138.7, 132.3, 131.6, 131.5, 130.2, 130.2, 122.4, 122.2, 122.2, 122.1, 121.4, 119.7, 114.4, 55.8, 44.3, 42.9, 40.7, 31.0, 21.8. HRMS (ESI): m/z $[M+H]^+$ calcd for $C_{46}H_{43}N_2O_{12}S_3$ 911.1973; found 911.1970.

***{A-B-C}* Trimeric fluorosulfate (7)**

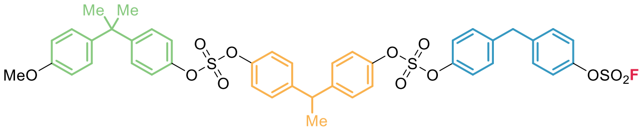

The title compound was prepared according to General Procedure F using **6** (1.10 g, 1.21 mmol, 1.0 equiv) and AgF (247 mg, 1.95 mmol, 1.6 equiv) in anhydrous MeCN (5 mL). After stirring for 16 h at 80 ˚C, the mixture was purified by flash column chromatography (hexanes : ethyl acetate = 3 : 1 to 2 : 1) to afford a colorless viscous oil (922 mg, 88%). ^{1}H NMR (400 MHz, CD_3CN) δ 7.41 – 7.18 (m, 20H), 7.18 – 7.08 (m, 2H), 6.88 – 6.77 (m, 2H), 4.26 (q, J = 7.0, 5.7 Hz, 1H), 4.03 (s, 2H), 3.73 (s, 3H), 1.68 – 1.55 (m, 9H). ^{13}C NMR (101 MHz, CD_3CN) δ 158.7, 152.8, 149.9, 149.7, 149.7, 149.6, 149.1, 147.0, 146.9, 143.2, 142.9, 141.8, 131.9, 131.5, 131.5, 130.2, 130.2, 129.5, 128.6, 122.2, 122.1, 122.0, 121.4, 114.5, 55.8, 44.3, 42.8, 40.7, 30.9, 21.8. ^{19}F NMR (376 MHz, CD_3CN) δ 36.0. HRMS (ESI): m/z $[M+Na]^+$ calcd for $C_{43}H_{39}FNaO_{12}S_3$ 885.1480; found 885.1480.

***{A-B-C-A}* Tetrameric imidazylate (9)**

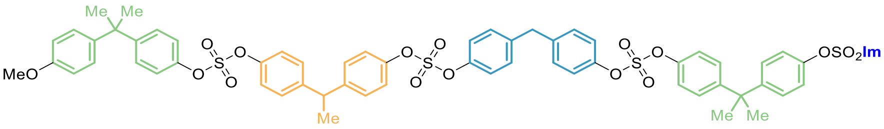

The title compound was prepared according to General Procedure E using **7** (819 mg, 0.95 mmol, 1.0 equiv), **8** (449 mg, 0.95 mmol, 1.0 equiv), and DBU (35 µL 0.24 mmol, 0.25 equiv) in anhydrous MeCN (3 mL). After stirring for 2 h at 80 ˚C, the mixture was purified by flash column chromatography (hexanes : ethyl acetate = 1 : 1) to afford a white solid (1.10 g, 96%). mp: 56 – 57 ˚C. ^{1}H NMR (400 MHz, CD_3CN) δ 7.80 – 7.76 (m, 1H), 7.45 – 7.39 (m, 1H), 7.37 – 7.17 (m, 26H), 7.16 – 7.09 (m, 3H), 6.93 – 6.86 (m, 2H), 6.85 – 6.78 (m, 2H), 4.25 (q, J = 7.1 Hz, 1H), 4.00 (s, 2H), 3.72 (s, 3H), 1.64 – 1.57 (m, 15H). ^{13}C NMR (101 MHz, CD_3CN) δ 158.7, 152.1, 151.8, 150.9, 149.8, 149.8, 149.7, 149.7, 149.4, 149.1, 148.1, 146.9, 142.9, 142.0, 142.0, 138.7, 132.2, 131.5, 130.2, 129.7, 129.6, 129.5, 128.6, 122.2, 122.1, 122.1, 121.8, 121.6, 121.4, 119.7, 114.4, 55.8, 44.3, 43.5, 42.8, 40.7, 31.0, 30.8, 21.8. HRMS (ESI): *m/z* $[M+H]^+$ calcd for $C_{61}H_{57}N_2O_{16}S_4$ 1201.2585; found 1201.2583.

***{A-B-C-A}* Tetrameric fluorosulfate (*10*)**

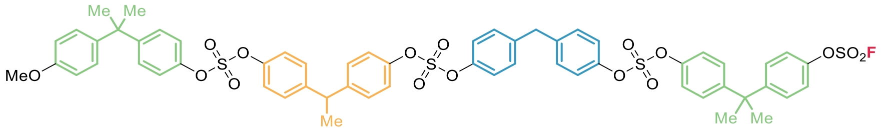

The title compound was prepared according to General Procedure F using **9** (995 mg, 0.83 mmol, 1.0 equiv) and AgF (167 mg, 1.32 mmol, 1.6 equiv) in anhydrous MeCN (3 mL). After stirring for 16 h at 80 ˚C, the mixture was purified by flash column chromatography (hexanes : ethyl acetate = 3 : 1 to 2 : 1) to afford a white solid (800 mg, 84%). mp: 60 – 62 ˚C. ^{1}H NMR (400 MHz, CD_3CN) δ 7.40 – 7.23 (m, 26H), 7.23 – 7.18 (m, 2H), 7.15 – 7.09 (m, 2H), 6.84 – 6.78 (m, 2H), 4.26 (q, J = 7.2 Hz, 1H), 4.01 (s, 2H), 3.72 (s, 3H), 1.68 – 1.55 (m, 15H). ^{13}C NMR (101 MHz, CD_3CN) δ 158.8, 152.2, 152.1, 150.8, 149.9, 149.8, 149.7, 149.7, 149.4, 149.2, 149.2, 149.2, 147.0, 146.9, 142.9, 142.0, 142.0, 131.5, 130.2, 130.2, 130.0, 129.6, 129.5, 128.6, 122.2, 122.1, 122.1, 121.7, 121.5, 121.5, 121.4, 114.4, 55.8, 44.3, 43.6, 42.9, 40.8, 31.0, 30.8, 21.8. ^{19}F NMR (376 MHz, CD_3CN) δ 36.0. HRMS (ESI): *m/z* $[M+Na]^+$ calcd for $C_{58}H_{53}FNaO_{16}S_4$ 1175.2093; found 1175.2098.

***{A-B-C-A-B}* Pentameric imidazylate (*11*)**

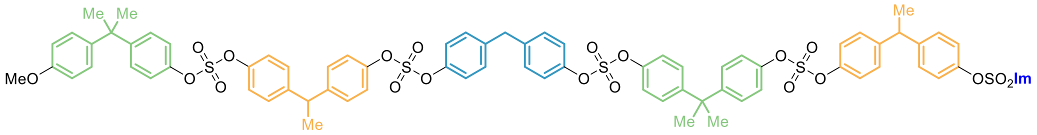

The title compound was prepared according to General Procedure E using **10** (619 mg, 0.54 mmol, 1.0 equiv), **2** (248 mg, 0.54 mmol, 1.0 equiv), and DBU (21 µL 0.14 mmol, 0.25 equiv) in anhydrous MeCN (2 mL). After stirring for 2 h at 80 ˚C, the mixture was purified by flash column chromatography (hexanes : ethyl acetate = 1 : 1) to afford a white solid (748 mg, 94%). mp: 61 – 62 ˚C. ^{1}H NMR (400 MHz, CD_3CN) δ 7.78 – 7.75 (m, 1H), 7.43 – 7.39 (m, 1H), 7.34 – 7.17 (m, 34H), 7.15 – 7.09 (m, 3H), 6.93 – 6.87 (m, 2H), 6.84 – 6.77 (m, 2H), 4.30 – 4.16 (m, 2H), 3.99 (d, J = 3.9 Hz, 2H), 3.72 (d, J = 1.8 Hz, 3H), 1.67 – 1.49 (m, 18H). ^{13}C NMR (101 MHz, CD_3CN) δ 158.7, 152.1, 151.0, 149.8, 149.8, 149.7, 149.7, 149.4, 149.1, 148.4, 147.7, 146.9, 146.9, 146.8, 142.9, 142.0, 142.0, 138.6, 132.3, 131.5, 130.3, 130.2, 130.2, 129.6, 129.5, 128.6, 122.3, 122.2, 122.1, 122.1, 122.1, 121.7, 121.6, 121.4, 119.7, 114.4, 55.8, 44.3, 44.3, 43.5, 42.8, 40.7, 31.0, 30.8, 21.8, 21.8. HRMS (ESI): *m/z* $[M+H]^+$ calcd for $C_{75}H_{69}N_2O_{20}S_5$ 1477.3042; found 1477.3047.

4-(4-(((4-(2-(4-Methoxyphenyl)propan-2-yl)phenoxy)sulfonyl)oxy)benzyl)phenyl 1H-imidazole-1-sulfonate (**12**)

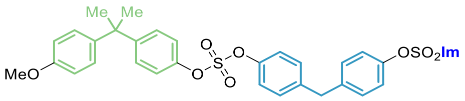

The title compound was prepared according to General Procedure E using **1** (649 mg, 2.00 mmol, 1.0 equiv), **5** (889 mg, 2.00 mmol, 1.0 equiv), and DBU (75 µL, 0.50 mmol, 0.25 equiv) in anhydrous MeCN (7 mL). After stirring for 2 h at 80 ˚C, the mixture was purified by flash column chromatography (hexanes : ethyl acetate = 2 : 1 to 1 : 1) to afford a colorless oil (1.21 g, 95%). ^{1}H NMR (400 MHz, CD_3CN) δ 7.81 – 7.76 (m, 1H), 7.46 – 7.40 (m, 1H), 7.36 – 7.20 (m, 10H), 7.19 – 7.10 (m, 3H), 6.98 – 6.89 (m, 2H), 6.88 – 6.80 (m, 2H), 4.00 (s, 2H), 3.75 (s, 3H), 1.64 (s, 6H). ^{13}C NMR (101 MHz, CD_3CN) δ 158.7, 152.2, 149.9, 149.1, 148.5, 142.9, 142.8, 141.8, 138.7, 132.3, 131.7, 131.5, 129.5, 128.6, 122.4, 122.2, 121.4, 119.7, 114.4, 55.8, 42.9, 40.7, 31.0. HRMS (EI): *m/z* $[M]^+$ calcd $C_{32}H_{30}N_2O_8S_2$ 634.1444; found 634.1433.

4-(4-(((4-(2-(4-Methoxyphenyl)propan-2-yl)phenoxy)sulfonyl)oxy)benzyl)phenyl fluorosulfate (**13**)

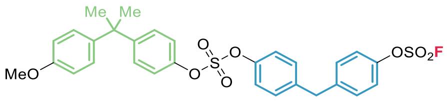

The title compound was prepared according to General Procedure F using **12** (1.07 g, 1.69 mmol, 1.0 equiv) and AgF (369 mg, 2.91 mmol, 1.6 equiv) in anhydrous MeCN (7 mL). After stirring for 16 h at 80 ˚C, the mixture was purified by flash column chromatography (hexanes : ethyl acetate = 8 : 1 to 2 : 1) to afford a colorless viscous oil (944 mg, 95%). ^{1}H NMR (400 MHz, CD_3CN) δ 7.41 – 7.20 (m, 6H), 7.17 – 7.12 (m, 1H), 6.87 – 6.81 (m, 1H), 4.06 (s, 1H), 3.75 (s, 1H), 1.64 (s, 3H). ^{13}C NMR (101 MHz, CD_3CN) δ 158.8, 152.2, 149.9, 149.6, 149.1, 143.2, 142.9, 141.8, 131.9, 131.6, 129.5, 128.6, 122.2, 122.1, 121.4, 114.4, 55.8, 42.9, 40.7, 31.0. ^{19}F NMR (376 MHz, CD_3CN) δ 36.0. HRMS (EI): *m/z* $[M]^+$ calcd for $C_{29}H_{27}FO_8S_2$ 586.1131; found 586.1131.

{A-C-C} *Trimeric imidazylate* (**14**)

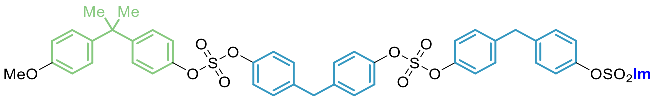

The title compound was prepared according to General Procedure E using **13** (784 mg, 1.34 mmol, 1.0 equiv), **5** (596 mg, 1.34 mmol, 1.0 equiv), and DBU (51 µL, 0.34 mmol, 0.25 equiv) in anhydrous MeCN (6 mL). After stirring for 2 h at 80 ˚C, the mixture was purified by flash column chromatography (hexanes : ethyl acetate = 2 : 1 to 1 : 1) to afford a colorless viscous oil (1.10 g, 92%). ^{1}H NMR (400 MHz, CD_3CN) δ 7.80 – 7.77 (m, 1H), 7.44 – 7.41 (m, 1H), 7.35 – 7.19 (m, 18H), 7.16 – 7.10 (m, 3H), 6.95 – 6.89 (m, 2H), 6.85 – 6.79 (m, 2H), 4.00 (d, J = 15.2 Hz, 4H), 3.73 (s, 3H), 1.63 (s, 6H). ^{13}C NMR (101 MHz, CD_3CN) δ 158.7, 152.1, 149.8, 149.8, 149.8, 149.1, 148.5, 142.9, 142.8, 142.0, 142.0, 141.8, 138.7, 132.3, 131.6, 131.5, 129.5, 128.6, 122.4, 122.2, 122.2, 121.4, 119.7, 114.4, 55.8, 42.9, 40.7, 40.7, 31.0. HRMS (ESI): *m/z* $[M+Cl]^-$ calcd for $C_{45}H_{40}ClN_2O_{12}S_3$ 931.1437; found 931.1432.

{A-C-C} *Trimeric fluorosulfate* (**15**)

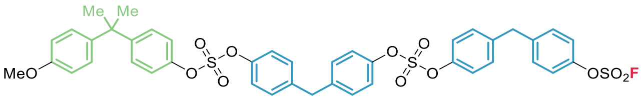

The title compound was prepared according to General Procedure F using **14** (945 mg, 1.05 mmol, 1.0 equiv) and AgF (214 mg, 1.69 mmol, 1.6 equiv) in anhydrous MeCN (6 mL). After stirring for 16 h at 80 ˚C, the mixture was purified by flash column chromatography (hexanes : ethyl acetate = 4 : 1 to 2 : 1) to afford a colorless viscous oil (842 mg, 94%). ^{1}H NMR (400 MHz, CD_3CN) δ 7.41 – 7.19 (m, 20H), 7.17 – 7.10 (m, 2H), 6.85 – 6.79 (m, 2H), 4.03 (d, J = 6.5 Hz, 4H), 3.74 (s, 3H), 1.63 (s, 6H). ^{13}C NMR (101 MHz, CD_3CN) δ 158.7, 152.1, 149.9, 149.9, 149.8, 149.6, 149.1, 143.2, 142.9, 142.1, 131.9, 131.6, 131.5, 131.5, 129.5, 128.6, 122.2, 122.2, 121.4, 114.4, 55.8, 42.9, 40.7, 40.7, 31.0. ^{19}F NMR (376 MHz, CD_3CN) δ 36.0. HRMS (ESI): *m/z* [M+Cl]$^-$ calcd for $C_{42}H_{37}ClFO_{12}S_3$ 883.1125; found 883.1128.

***{A-C-C-B}* Tetrameric imidazylate (16)**

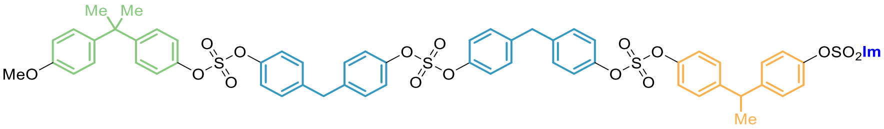

The title compound was prepared according to General Procedure E using **15** (721 mg, 0.85 mmol, 1.0 equiv), **2** (390 mg, 0.85 mmol, 1.0 equiv), and DBU (31 µL 0.21 mmol, 0.25 equiv) in anhydrous MeCN (5 mL). After stirring for 2 h at 80 ˚C, the mixture was purified by flash column chromatography (hexanes : ethyl acetate : DCM = 2 : 1 : 1) to afford a white solid (956 mg, 96%). mp: 54 – 55 ˚C. ^{1}H NMR (400 MHz, CD_3CN) δ 7.80 – 7.74 (m, 1H), 7.44 – 7.39 (m, 1H), 7.36 – 7.18 (m, 26H), 7.15 – 7.10 (m, 3H), 6.95 – 6.87 (m, 2H), 6.85 – 6.79 (m, 2H), 4.22 (q, J = 7.2 Hz, 1H), 4.01 (s, 4H), 3.73 (s, 3H), 1.62 (s, 6H), 1.55 (d, J = 7.2 Hz, 3H). ^{13}C NMR (101 MHz, CD_3CN) δ 158.7, 152.1, 149.8, 149.8, 149.7, 149.1, 148.4, 147.7, 146.8, 142.9, 142.0, 142.0, 138.7, 132.3, 131.5, 130.3, 130.2, 129.5, 128.6, 122.3, 122.2, 122.1, 121.4, 119.7, 114.4, 55.8, 44.3, 42.9, 40.7, 31.0, 21.8. HRMS (ESI): *m/z* [M+Cl]$^-$ calcd for $C_{59}H_{52}ClN_2O_{16}S_4$ 1207.1894; found 1207.1890.

***{A-C-C-B}* Tetrameric fluorosulfate (17)**

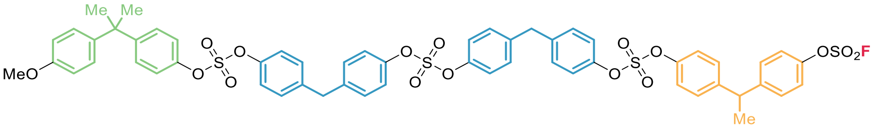

The title compound was prepared according to General Procedure F using **16** (815 mg, 0.69 mmol, 1.0 equiv) and AgF (140 mg, 1.10 mmol, 1.6 equiv) in anhydrous MeCN (4 mL). After stirring for 16 h at 80 ˚C, the mixture was purified by flash column chromatography (hexanes : ethyl acetate : DCM = 2 : 1 : 1) to afford a white solid (700 mg, 90%). mp: 45 – 52 ˚C. ^{1}H NMR (400 MHz, CD_3CN) δ 7.43 – 7.19 (m, 28H), 7.15 – 7.10 (m, 2H), 6.85 – 6.79 (m, 2H), 4.28 (q, J = 7.2 Hz, 1H), 4.01 (s, 4H), 3.73 (s, 3H), 1.66 – 1.55 (m, 9H). ^{13}C NMR (101 MHz, CD_3CN) δ 158.7, 152.1, 149.8, 149.7, 149.5, 149.1, 148.1, 146.7, 142.9, 142.0, 142.0, 131.5, 130.6, 130.2, 129.5, 128.6, 122.2, 122.1, 122.0, 122.0, 121.4, 114.4, 55.8, 44.3, 42.9, 40.7, 31.0, 21.7. ^{19}F NMR (376 MHz, CD_3CN) δ 36.0. HRMS (ESI): *m/z* [M+Na]$^+$ calcd for $C_{56}H_{49}FNaO_{16}S_4$ 1147.1780; found 1147.1778.

***{A-C-C-B-B}* Pentameric imidazylate (18)**

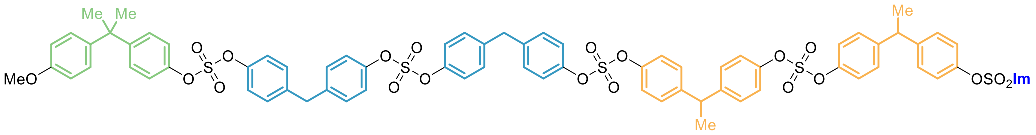

The title compound was prepared according to General Procedure E using **17** (571 mg, 0.51 mmol, 1.0 equiv), **2** (234 mg, 0.51 mmol, 1.0 equiv), and DBU (19 µL 0.13 mmol, 0.25 equiv) in anhydrous MeCN (3.5 mL). After stirring for 2 h at 80 ˚C, the mixture was purified by flash column chromatography (hexanes : ethyl acetate : DCM = 3 : 1 : 1 to 1 : 1 : 1) to afford a white solid (693 mg, 94%). mp: 58 – 59 ˚C. ^{1}H NMR (400 MHz, CD_3CN) δ 7.81 – 7.73 (m, 1H), 7.45 – 7.38 (m, 1H), 7.37 – 7.17 (m, 34H), 7.17 – 7.09 (m, 3H), 6.95 – 6.87 (m, 2H), 6.84 – 6.77 (m, 2H), 4.23 (dq, J = 14.3, 7.2 Hz, 2H), 4.00 (s, 4H), 3.72 (s, 3H), 1.66 – 1.49 (m, 12H). ^{13}C NMR (101 MHz, CD_3CN) δ 158.7, 152.1, 149.8, 149.8, 149.7, 149.7, 149.1, 148.4, 147.7, 147.0, 146.8, 142.9, 142.0, 142.0, 138.7, 132.3, 131.5, 130.3, 130.2, 130.2, 129.5, 128.6, 122.3, 122.2, 122.1, 122.1, 122.1, 121.4, 119.7, 114.4, 55.8, 44.3, 44.3, 42.9, 40.7, 31.0, 21.8, 21.8. HRMS (ESI): *m/z* $[M+Na]^+$ calcd for $C_{73}H_{64}N_2NaO_{20}S_5$ 1471.2548; found 1471.2546.

4-(2-(4-(((4-(2-(4-Methoxyphenyl)propan-2-yl)phenoxy)sulfonyl)oxy)phenyl)propan-2-yl)phenyl 1H-imidazole-1-sulfonate ***(19)***

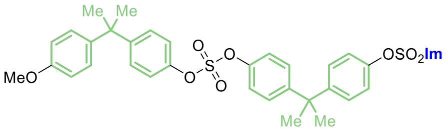

The title compound was prepared according to General Procedure E using **1** (811 mg, 2.50 mmol, 1.0 equiv), **8** (1.18 g, 2.50 mmol, 1.0 equiv), and DBU (94 µL, 0.63 mmol, 0.25 equiv) in anhydrous MeCN (7 mL). After stirring for 2 h at 80 ˚C, the mixture was purified by flash column chromatography (hexanes : ethyl acetate = 3 : 1 to 1 : 1) to afford a colorless oil (1.56 g, 94%). ^{1}H NMR (400 MHz, DMSO-d_6) δ 8.22 – 8.16 (m, 1H), 7.83 – 7.78 (m, 1H), 7.39 – 7.27 (m, 10H), 7.26 – 7.22 (m, 1H), 7.17 – 7.09 (m, 2H), 7.02 – 6.95 (m, 2H), 6.87 – 6.80 (m, 2H), 3.71 (s, 3H), 1.62 (d, J = 3.3 Hz, 12H). ^{13}C NMR (101 MHz, DMSO-d_6) δ 157.2, 150.6, 150.2, 149.5, 147.8, 147.6, 146.7, 141.5, 138.1, 131.4, 128.7, 128.5, 128.4, 127.4, 120.8, 120.6, 120.4, 119.1, 113.4, 54.9, 42.4, 41.7, 30.4, 30.2. HRMS (ESI): *m/z* $[M+H]^+$ calcd for $C_{34}H_{35}N_2O_8S_2$ 663.1829; found 663.1829.

4-(2-(4-(((4-(2-(4-Methoxyphenyl)propan-2-yl)phenoxy)sulfonyl)oxy)phenyl)propan-2-yl)phenyl fluorosulfate ***(20)***

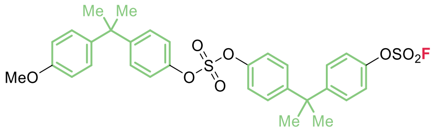

The title compound was prepared according to General Procedure F using **19** (1.57 g, 2.37 mmol, 1.0 equiv) and AgF (482 mg, 3.80 mmol, 1.6 equiv) in anhydrous MeCN (8 mL). After stirring for 16 h at 80 ˚C, the mixture was purified by flash column chromatography (hexanes : ethyl acetate = 5 : 1) to afford a colorless viscous oil (1.36 g, 93%). ^{1}H NMR (400 MHz, CD_3CN) δ 7.42 – 7.37 (m, 2H), 7.37 – 7.30 (m, 6H), 7.29 – 7.21 (m, 4H), 7.18 – 7.12 (m, 2H), 6.86 – 6.80 (m, 2H), 3.74 (s, 3H), 1.69 (s, 6H), 1.64 (s, 6H). ^{13}C NMR (101 MHz, CD_3CN) δ 158.7, 152.2,

152.2, 150.8, 149.5, 149.2, 149.2, 142.9, 130.0, 129.6, 129.5, 128.6, 121.7, 121.5, 121.4, 114.4, 55.8, 43.6, 42.9, 31.0, 30.8. ^{19}F NMR (376 MHz, CD_3CN) δ 36.0. HRMS (EI): *m/z* $[M]^+$ calcd for $C_{31}H_{36}O_8S_2$ 614.1444; found 614.1460.

***{A-A-C}** Trimeric imidazylate (**21**)*

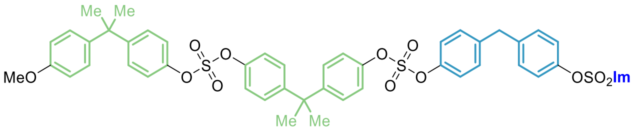

The title compound was prepared according to General Procedure E using **20** (963 mg, 1.57 mmol, 1.0 equiv), **5** (698 mg, 1.57 mmol, 1.0 equiv), and DBU (58 µL, 0.39 mmol, 0.25 equiv) in anhydrous MeCN (7 mL). After stirring for 2 h at 80 ˚C, the mixture was purified by flash column chromatography (hexanes : ethyl acetate = 3 : 2) to afford a colorless viscous oil (1.32 g, 91%). ^{1}H NMR (400 MHz, CD_3CN) δ 7.79 – 7.76 (m, 1H), 7.44 – 7.41 (m, 1H), 7.36 – 7.29 (m, 6H), 7.29 – 7.20 (m, 12H), 7.16 – 7.11 (m, 3H), 6.95 – 6.90 (m, 2H), 6.85 – 6.80 (m, 2H), 3.99 (s, 2H), 3.73 (s, 3H), 1.68 (s, 6H), 1.63 (s, 6H). ^{13}C NMR (101 MHz, CD_3CN) δ 158.7, 152.1, 151.0, 151.0, 149.9, 149.4, 149.4, 149.2, 148.5, 142.9, 142.8, 141.8, 138.7, 132.3, 131.7, 131.5, 129.6, 129.6, 129.5, 128.6, 122.4, 122.2, 121.7, 121.4, 119.7, 114.4, 55.8, 43.5, 42.9, 40.7, 31.0, 30.8. HRMS (ESI): *m/z* $[M+H]^+$ calcd for $C_{47}H_{45}N_2O_{12}S_3$ 925.2129; found 925.2136.

***{A-A-C}** Trimeric fluorosulfate (**22**)*

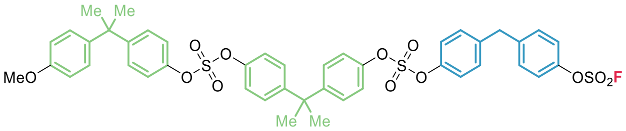

The title compound was prepared according to General Procedure F using **21** (1.18 g, 1.28 mmol, 1.0 equiv) and AgF (260 mg, 2.05 mmol, 1.6 equiv) in anhydrous MeCN (7 mL). After stirring for 16 h at 80 ˚C, the mixture was purified by flash column chromatography (hexanes : ethyl acetate = 2 : 1) to afford a colorless viscous oil (1.07 g, 95%). ^{1}H NMR (400 MHz, CD_3CN) δ 7.40 – 7.19 (m, 20H), 7.16 – 7.11 (m, 2H), 6.85 – 6.79 (m, 2H), 4.04 (s, 2H), 3.73 (s, 3H), 1.67 (s, 6H), 1.63 (s, 6H). ^{13}C NMR (101 MHz, CD_3CN) δ 158.7, 152.1, 151.0, 151.0, 149.9, 149.6, 149.4, 149.4, 149.2, 143.2, 142.9, 141.8, 131.9, 131.6, 129.6, 129.6, 129.5, 128.6, 122.2, 122.1, 122.0, 121.7, 121.4, 114.4, 55.8, 43.5, 42.9, 40.7, 31.0, 30.8. ^{19}F NMR (376 MHz, CD_3CN) δ 36.0. HRMS (ESI): *m/z* $[M+NH_4]^+$ calcd for $C_{44}H_{45}FNO_{12}S_3$ 894.2082; found 894.2093.

***{A-A-C-B}** Tetrameric imidazylate (**23**)*

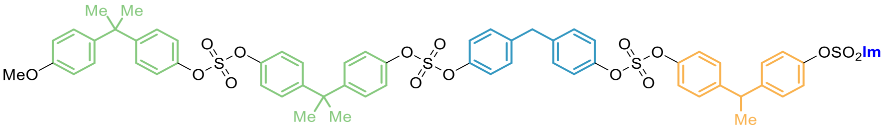

The title compound was prepared according to General Procedure E using **22** (940 mg, 1.07 mmol, 1.0 equiv), **2** (491 mg, 1.07 mmol, 1.0 equiv), and DBU (40 µL 0.27 mmol, 0.25 equiv) in anhydrous MeCN (6 mL). After stirring for 2 h at 80 ˚C, the mixture was purified by flash column chromatography (hexanes : ethyl acetate : DCM

= 2 : 1 : 1 to 1 : 1 : 1) to afford a white solid (1.24 g, 96%). mp: 55 – 58 ˚C. ^{1}H NMR (400 MHz, CD_3CN) δ 7.80 – 7.75 (m, 1H), 7.44 – 7.40 (m, 1H), 7.37 – 7.17 (m, 26H), 7.16 – 7.09 (m, 3H), 6.94 – 6.87 (m, 2H), 6.84 – 6.77 (m, 2H), 4.22 (q, J = 7.2 Hz, 1H), 4.01 (s, 2H), 3.72 (s, 3H), 1.72 – 1.49 (m, 15H). ^{13}C NMR (101 MHz, CD_3CN) δ 158.7, 152.1, 151.0, 151.0, 149.9, 149.8, 149.7, 149.4, 149.4, 149.2, 148.4, 147.7, 146.8, 142.9, 142.0, 138.7, 132.3, 131.5, 130.4, 130.2, 129.6, 129.6, 129.5, 128.6, 122.3, 122.2, 122.1, 121.7, 121.4, 119.7, 114.4, 55.8, 44.3, 43.5, 42.9, 40.7, 31.0, 30.8, 21.9. HRMS (ESI): *m/z* $[M+H]^+$ calcd for $C_{61}H_{57}N_2O_{16}S_4$ 1201.2585; found 1201.2583.

***{A-A-C-B}** Tetrameric fluorosulfate (**24**)*

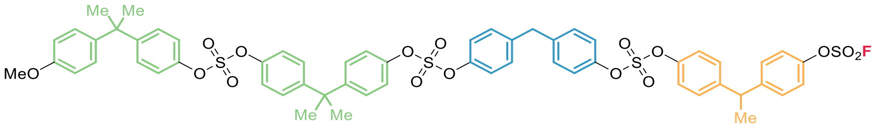

The title compound was prepared according to General Procedure F using **23** (1.06 g, 0.88 mmol, 1.0 equiv) and AgF (179 mg, 1.41 mmol, 1.6 equiv) in anhydrous MeCN (4 mL). After stirring for 16 h at 80 ˚C, the mixture was purified by flash column chromatography (hexanes : ethyl acetate : DCM = 6 : 1 : 1 to 2 : 1 : 1) to afford a white solid (938 mg, 92%). mp: 55 – 57 ˚C. ^{1}H NMR (400 MHz, CD_3CN) δ 7.42 – 7.18 (m, 28H), 7.12 (m, 2H), 6.84 – 6.78 (m, 2H), 4.28 (q, J = 7.2 Hz, 1H), 4.01 (s, 2H), 3.72 (s, 3H), 1.69 – 1.57 (m, 15H). ^{13}C NMR (101 MHz, CD_3CN) δ 158.8, 152.1, 151.0, 151.0, 149.9, 149.8, 149.8, 149.5, 149.5, 149.4, 149.4, 149.2, 148.1, 146.7, 142.9, 142.0, 131.5, 130.6, 130.2, 129.6, 129.6, 129.5, 128.6, 122.2, 122.2, 122.0, 122.0, 121.7, 121.4, 114.4, 55.8, 44.4, 43.5, 42.9, 40.8, 31.0, 30.8, 21.8. ^{19}F NMR (376 MHz, CD_3CN) δ 36.0. HRMS (ESI): *m/z* $[M+Na]^+$ calcd for $C_{58}H_{53}FNaO_{16}S_4$ 1175.2093; found 1175.2083.

***{A-A-C-B-C}** Pentameric imidazylate (**25**)*

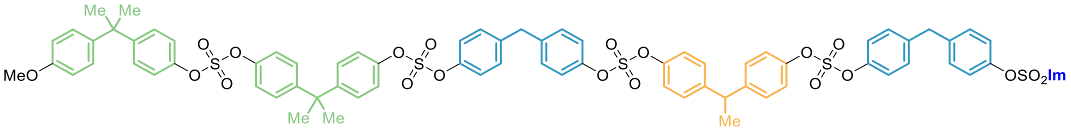

The title compound was prepared according to General Procedure E using **24** (763 mg, 0.66 mmol, 1.0 equiv), **5** (293 mg, 0.66 mmol, 1.0 equiv), and DBU (25 µL 0.17 mmol, 0.25 equiv) in anhydrous MeCN (4 mL). After stirring for 2 h at 80 ˚C, the mixture was purified by flash column chromatography (hexanes : ethyl acetate : DCM = 2 : 1 : 1 to 1 : 1 : 1) to afford a white solid (952 mg, 99%). mp: 59 – 62 ˚C. ^{1}H NMR (400 MHz, CD_3CN) δ 7.79 – 7.75 (m, 1H), 7.44 – 7.39 (m, 1H), 7.35 – 7.15 (m, 34H), 7.15 – 7.06 (m, 3H), 6.95 – 6.87 (m, 2H), 6.84 – 6.76 (m, 2H), 4.24 (q, J = 7.1 Hz, 1H), 3.97 (d, J = 9.8 Hz, 4H), 3.71 (s, 3H), 1.68 – 1.51 (m, 15H). ^{13}C NMR (101 MHz, CD_3CN) δ 158.7, 152.1, 151.0, 150.9, 149.8, 149.8, 149.7, 149.6, 149.4, 149.3, 149.1, 148.5, 146.9, 142.9, 142.7, 142.0, 141.8, 138.7, 132.3, 131.6, 131.5, 130.2, 129.5, 129.5, 129.5, 128.6, 122.4, 122.2, 122.2, 122.1, 121.6, 121.4, 119.7, 114.4, 55.8, 44.3, 43.5, 42.8, 40.7, 40.7, 31.0, 30.8, 21.8. HRMS (ESI): *m/z* $[M+H]^+$ calcd for $C_{74}H_{67}N_2O_{20}S_5$ 1463.2885; found 1463.2889.

***{A-B-A}** Trimeric imidazylate (**26**)*

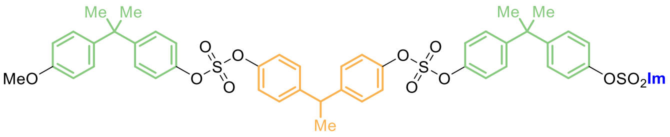

The title compound was prepared according to General Procedure E using **4** (1.04 g, 1.73 mmol, 1.0 equiv), **8** (818 mg, 1.73 mmol, 1.0 equiv), and DBU (66 µL 0.43 mmol, 0.25 equiv) in anhydrous MeCN (5 mL). After stirring for 2 h at 80 ˚C, the mixture was purified by flash column chromatography (hexanes : ethyl acetate = 3 : 1 to 2 : 1) to afford a colorless viscous oil (1.62 g, 100%). ^{1}H NMR (400 MHz, DMSO-d_6) δ 8.22 – 8.16 (m, 1H), 7.82 – 7.77 (m, 1H), 7.47 – 7.38 (m, 4H), 7.38 – 7.26 (m, 14H), 7.26 – 7.22 (m, 1H), 7.16 – 7.08 (m, 2H), 7.02 – 6.94 (m, 2H), 6.88 – 6.79 (m, 2H), 4.32 (q, J = 7.1 Hz, 1H), 3.71 (s, 3H), 1.70 – 1.53 (m, 15H). ^{13}C NMR (101 MHz, DMSO-d_6) δ 157.2, 150.6, 150.2, 149.5, 148.2, 148.2, 147.8, 147.6, 146.7, 145.7, 145.6, 141.5, 138.1, 131.4, 129.3, 129.3, 128.7, 128.5, 128.4, 127.4, 121.0, 121.0, 120.8, 120.6, 120.4, 119.1, 113.4, 54.9, 42.7, 42.4, 41.7, 30.4, 30.2, 21.2. HRMS (ESI): *m/z* $[M+H]^+$ calcd for $C_{48}H_{47}N_2O_{12}S_3$ 939.2286; found 939.2285.

***{A-B-A}** Trimeric fluorosulfate (**27**)*

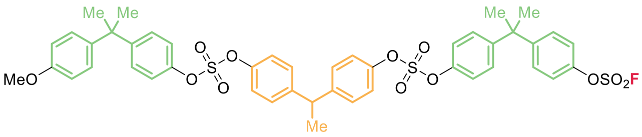

The title compound was prepared according to General Procedure F using **26** (1.20 g, 1.28 mmol, 1.0 equiv) and AgF (254 mg, 2.00 mmol, 1.6 equiv) in anhydrous MeCN (5 mL). After stirring for 16 h at 80 ˚C, the mixture was purified by flash column chromatography (hexanes : ethyl acetate = 3 : 1 to 2 : 1) to afford a colorless viscous oil (1.10 g, 96%). ^{1}H NMR (400 MHz, DMSO-d_6) δ 7.50 (m, 2H), 7.46 – 7.38 (m, 6H), 7.38 – 7.27 (m, 12H), 7.17 – 7.07 (m, 2H), 6.90 – 6.78 (m, 2H), 4.31 (q, J = 7.1 Hz, 1H), 3.70 (s, 3H), 1.66 (s, 6H), 1.63 – 1.54 (m, 9H). ^{13}C NMR (101 MHz, DMSO-d_6) δ 157.2, 150.8, 150.6, 149.3, 148.2, 147.9, 147.7, 147.6, 145.7, 145.6, 141.5, 129.3, 129.2, 128.9, 128.6, 128.4, 127.4, 121.0, 120.7, 120.6, 120.4, 113.4, 54.9, 42.7, 42.4, 41.7, 30.4, 30.1, 21.2. ^{19}F NMR (376 MHz, DMSO-d_6) δ 35.7. HRMS (ESI): *m/z* $[M+Na]^+$ calcd for $C_{45}H_{43}FNaO_{12}S_3$ 913.1793; found 913.1790.

***{A-B-A-C}** Tetrameric imidazylate (**28**)*

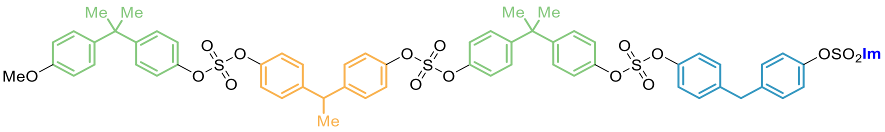

The title compound was prepared according to General Procedure E using **27** (1.00 g, 1.12 mmol, 1.0 equiv), **5** (496 mg, 1.12 mmol, 1.0 equiv), and DBU (42 µL 0.28 mmol, 0.25 equiv) in anhydrous MeCN (4 mL). After stirring for 2 h at 80 ˚C, the mixture was purified by flash column chromatography (hexanes : ethyl acetate = 1 : 1) to afford a white solid (1.31 g, 97%). mp: 54 – 55 ˚C. ^{1}H NMR (400 MHz, CD_3CN) δ 7.77 (m, 1H), 7.43 – 7.39 (m, 1H), 7.36 – 7.17 (m, 26H), 7.15 – 7.08 (m, 3H), 6.94 – 6.87 (m, 2H), 6.84 – 6.77 (m, 2H), 4.25 (q, J = 7.1 Hz, 1H), 3.97 (s, 2H), 3.72 (s, 3H), 1.77 – 1.46 (m, 15H). ^{13}C NMR (101 MHz, CD_3CN) δ 158.7, 152.1, 151.0, 149.9, 149.8, 149.8, 149.7, 149.4, 149.4, 149.1, 148.5, 147.0, 146.9, 142.9, 142.7, 142.0, 141.8, 138.7, 132.3, 131.7, 131.5,

130.2, 130.2, 129.6, 129.6, 129.5, 128.6, 122.4, 122.2, 122.2, 122.1, 121.7, 121.4, 119.7, 114.4, 55.8, 44.3, 43.5, 42.9, 40.7, 31.0, 30.8, 21.8. HRMS (ESI): *m/z* $[M+H]^+$ calcd for $C_{61}H_{57}N_2O_{16}S_4$ 1201.2585; found 1201.2587.

***{A-B-A-C}* Tetrameric fluorosulfate (*29*)**

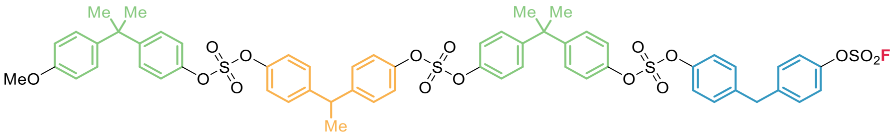

The title compound was prepared according to General Procedure F using **28** (961 mg, 0.80 mmol, 1.0 equiv) and AgF (162 mg, 1.28 mmol, 1.6 equiv) in anhydrous MeCN (4 mL). After stirring for 16 h at 80 ˚C, the mixture was purified by flash column chromatography (hexanes : ethyl acetate = 3 : 1 to 2 : 1) to afford a white solid (820 mg, 85%). mp: 59 – 60 ˚C. ^{1}H NMR (400 MHz, CD_3CN) *δ* 7.39 – 7.17 (m, 28H), 7.15 – 7.09 (m, 2H), 6.85 – 6.78 (m, 2H), 4.25 (q, *J* = 7.2 Hz, 1H), 4.03 (s, 2H), 3.72 (s, 3H), 1.68 – 1.56 (m, 15H). ^{13}C NMR (101 MHz, CD_3CN) *δ* 158.8, 152.1, 151.0, 151.0, 149.9, 149.7, 149.7, 149.6, 149.6, 149.4, 149.4, 149.2, 147.0, 146.9, 143.2, 142.9, 141.8, 131.9, 131.6, 130.2, 130.2, 129.6, 129.6, 129.5, 128.6, 122.2, 122.1, 122.0, 122.0, 121.7, 121.4, 114.4, 55.8, 44.3, 43.5, 42.9, 40.7, 31.0, 30.8, 21.8. ^{19}F NMR (376 MHz, CD_3CN) δ 36.0. HRMS (ESI): *m/z* $[M+Na]^+$ calcd for $C_{58}H_{53}FNaO_{16}S_4$ 1175.2093; found 1175.2099.

***{A-B-A-C-B}* Pentameric imidazylate (*30*)**

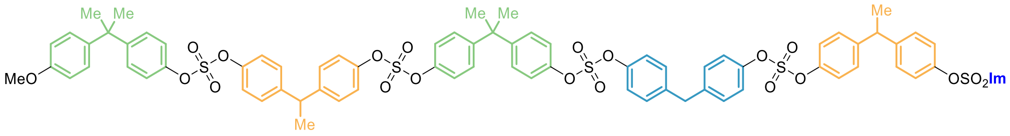

The title compound was prepared according to General Procedure E using **29** (678 mg, 0.59 mmol, 1.0 equiv), **2** (275 mg, 0.60 mmol, 1.0 equiv), and DBU (22 μL 0.15 mmol, 0.25 equiv) in anhydrous MeCN (2 mL). After stirring for 2 h at 80 ˚C, the mixture was purified by flash column chromatography (hexanes : ethyl acetate = 1 : 1) to afford a white solid (796 mg, 91%). mp: 61 – 64 ˚C. ^{1}H NMR (400 MHz, CD_3CN) *δ* 7.79 – 7.74 (m, 1H), 7.41 (m, 1H), 7.36 – 7.16 (m, 34H), 7.16 – 7.08 (m, 3H), 6.95 – 6.88 (m, 2H), 6.84 – 6.77 (m, 2H), 4.23 (dq, *J* = 11.5, 7.2 Hz, 2H), 3.99 (d, *J* = 5.8 Hz, 2H), 3.72 (d, *J* = 1.7 Hz, 3H), 1.68 – 1.50 (m, 18H). ^{13}C NMR (101 MHz, CD_3CN) *δ* 158.7, 152.1, 151.0, 149.8, 149.8, 149.7, 149.7, 149.4, 149.1, 148.4, 147.7, 146.9, 146.9, 146.8, 142.9, 142.0, 138.7, 132.3, 131.5, 130.3, 130.2, 130.2, 130.2, 129.6, 129.5, 128.6, 122.3, 122.2, 122.1, 122.1, 121.7, 121.4, 119.7, 114.4, 55.8, 44.3, 44.3, 43.5, 42.9, 40.7, 31.0, 30.8, 21.8, 21.8. HRMS (ESI): *m/z* $[M+H]^+$ calcd for $C_{75}H_{69}N_2O_{20}S_5$ 1477.3042; found 1477.3042.

***{A-C-B}* Trimeric imidazylate (*31*)**

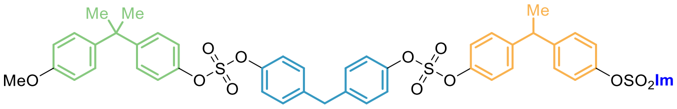

The title compound was prepared according to General Procedure E using **13** (803 mg, 1.37 mmol, 1.0 equiv), **2** (628 mg, 1.37 mmol, 1.0 equiv), and DBU (51 μL 0.34 mmol, 0.25 equiv) in anhydrous MeCN (7 mL). After stirring for 2 h at 80 ˚C, the mixture was purified by flash column chromatography (hexanes : ethyl acetate = 2 : 1

to 1 : 2) to afford a colorless viscous oil (1.16 g, 93%). ^{1}H NMR (400 MHz, CD_3CN) δ 7.80 – 7.75 (m, 1H), 7.45 – 7.39 (m, 1H), 7.37 – 7.18 (m, 18H), 7.17 – 7.09 (m, 3H), 6.96 – 6.88 (m, 2H), 6.86 – 6.79 (m, 2H), 4.24 (q, *J* = 7.2 Hz, 1H), 4.03 (s, 2H), 3.74 (s, 3H), 1.63 (s, 6H), 1.57 (d, *J* = 7.2 Hz, 3H). ^{13}C NMR (101 MHz, CD_3CN) δ 158.7, 152.1, 149.9, 149.8, 149.7, 149.1, 148.4, 147.7, 146.8, 142.9, 142.0, 142.0, 138.7, 132.3, 131.5, 131.5, 130.3, 130.2, 129.5, 128.6, 122.3, 122.2, 122.2, 122.1, 121.4, 119.7, 114.4, 55.8, 44.3, 42.9, 40.7, 31.0, 21.8. HRMS (ESI): *m/z* $[M+H]^+$ calcd for $C_{46}H_{43}N_2O_{12}S_3$ 911.1973; found 911.1971.

***{A-C-B}** Trimeric fluorosulfate (**32**)*

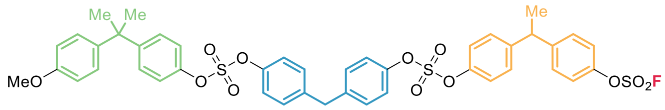

The title compound was prepared according to General Procedure F using **31** (1.01 g, 1.11 mmol, 1.0 equiv) and AgF (225 mg, 1.77 mmol, 1.6 equiv) in anhydrous MeCN (4 mL). After stirring for 16 h at 80 ˚C, the mixture was purified by flash column chromatography (hexanes : ethyl acetate = 3 : 1 to 2 : 1) to afford a colorless viscous oil (878 mg, 92%). ^{1}H NMR (400 MHz, CD_3CN) δ 7.45 – 7.18 (m, 20H), 7.17 – 7.10 (m, 2H), 6.87 – 6.79 (m, 2H), 4.29 (q, *J* = 7.2 Hz, 1H), 4.03 (s, 2H), 3.74 (s, 3H), 1.65 – 1.59 (m, 9H). ^{13}C NMR (101 MHz, CD_3CN) δ 158.7, 152.1, 149.9, 149.8, 149.7, 149.5, 149.1, 148.1, 146.7, 142.9, 142.1, 142.0, 131.5, 131.5, 130.6, 130.2, 129.5, 128.6, 122.2, 122.2, 122.0, 122.0, 121.4, 114.4, 55.8, 44.3, 42.9, 40.7, 31.0, 21.7. ^{19}F NMR (376 MHz, CD_3CN) δ 36.0. HRMS (ESI): *m/z* $[M+Na]^+$ calcd for $C_{43}H_{39}FNaO_{12}S_3$ 885.1480; found 885.1484.

***{A-C-B-A}** Tetrameric imidazylate (**33**)*

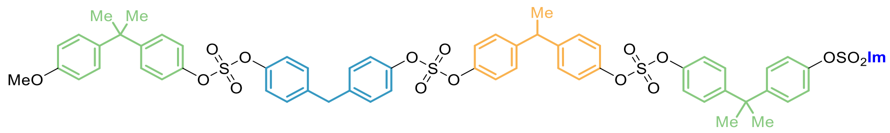

The title compound was prepared according to General Procedure E using **32** (737 mg, 0.85 mmol, 1.0 equiv), **8** (402 mg, 0.85 mmol, 1.0 equiv), and DBU (31 µL 0.21 mmol, 0.25 equiv) in anhydrous MeCN (4 mL). After stirring for 2 h at 80 ˚C, the mixture was purified by flash column chromatography (hexanes : ethyl acetate : DCM = 2 : 1 : 1 to 1 : 1 : 1) to afford a white solid (947 mg, 93%). mp: 61 – 65 ˚C. ^{1}H NMR (400 MHz, CD_3CN) δ 7.80 – 7.76 (m, 1H), 7.45 – 7.39 (m, 1H), 7.37 – 7.17 (m, 26H), 7.16 – 7.09 (m, 3H), 6.93 – 6.86 (m, 2H), 6.85 – 6.78 (m, 2H), 4.25 (q, *J* = 7.1 Hz, 1H), 4.00 (s, 2H), 3.72 (s, 3H), 1.64 – 1.57 (m, 15H). ^{13}C NMR (101 MHz, CD_3CN) δ 158.7, 152.1, 151.8, 150.9, 149.8, 149.8, 149.7, 149.7, 149.4, 149.1, 148.1, 146.9, 142.9, 142.0, 142.0, 138.7, 132.2, 131.5, 130.2, 129.7, 129.6, 129.5, 128.6, 122.2, 122.1, 122.1, 121.8, 121.6, 121.4, 119.7, 114.4, 55.8, 44.3, 43.5, 42.8, 40.7, 31.0, 30.8, 21.8. HRMS (ESI): *m/z* $[M+H]^+$ calcd for $C_{61}H_{57}N_2O_{16}S_4$ 1201.2585; found 1201.2586.

***{A-C-B-A}** Tetrameric fluorosulfate (**34**)*

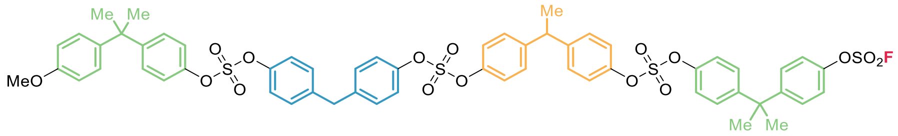

The title compound was prepared according to General Procedure F using **33** (811 mg, 0.68 mmol, 1.0 equiv) and AgF (138 mg, 1.09 mmol, 1.6 equiv) in anhydrous MeCN (3 mL). After stirring for 16 h at 80 ˚C, the mixture was purified by flash column chromatography (hexanes : ethyl acetate : DCM = 8 : 1 : 1 to 3 : 1 : 1) to afford a white solid (687 mg, 88%). mp: 52 – 54 ˚C. ^{1}H NMR (400 MHz, CD_3CN) δ 7.41 – 7.18 (m, 28H), 7.16 – 7.08 (m, 2H), 6.85 – 6.78 (m, 2H), 4.25 (q, J = 7.2 Hz, 1H), 4.00 (s, 2H), 3.72 (s, 3H), 1.66 (s, 6H), 1.63 – 1.55 (m, 9H). ^{13}C NMR (101 MHz, CD_3CN) δ 158.7, 152.1, 152.1, 150.8, 149.8, 149.8, 149.7, 149.7, 149.4, 149.2, 149.2, 149.1, 146.9, 146.9, 142.9, 142.0, 142.0, 131.5, 130.2, 130.2, 130.0, 129.6, 129.5, 128.6, 122.2, 122.1, 122.1, 121.7, 121.5, 121.5, 121.4, 114.4, 55.8, 44.3, 43.6, 42.8, 40.7, 31.0, 30.7, 21.8. ^{19}F NMR (376 MHz, CD_3CN) δ 36.0. HRMS (ESI): m/z $[M+Na]^+$ calcd for $C_{58}H_{53}FNaO_{16}S_4$ 1175.2093; found 1175.2101.

***{A-C-B-A-B}** Pentameric imidazylate (**35**)*

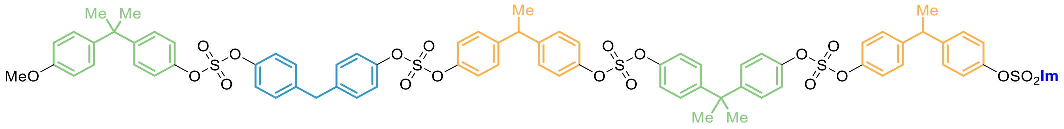

The title compound was prepared according to General Procedure E using **34** (587 mg, 0.51 mmol, 1.0 equiv), **2** (234 mg, 0.51 mmol, 1.0 equiv), and DBU (19 µL 0.13 mmol, 0.25 equiv) in anhydrous MeCN (3 mL). After stirring for 2 h at 80 ˚C, the mixture was purified by flash column chromatography (hexanes : ethyl acetate : DCM = 3 : 1 : 1 to 1 : 1 : 1) to afford a viscous solid (651 mg, 86%). mp: 62 – 63 ˚C. ^{1}H NMR (400 MHz, CD_3CN) δ 7.79 – 7.75 (m, 1H), 7.43 – 7.39 (m, 1H), 7.36 – 7.18 (m, 34H), 7.15 – 7.09 (m, 3H), 6.93 – 6.88 (m, 2H), 6.84 – 6.78 (m, 2H), 4.24 (dq, J = 14.4, 7.2 Hz, 2H), 4.00 (s, 2H), 3.72 (s, 3H), 1.67 – 1.60 (m, 12H), 1.58 (dd, J = 7.3 Hz, 9H), 1.55 (dd, J = 7.2, 1.8 Hz, 3H). ^{13}C NMR (101 MHz, CD_3CN) δ 158.7, 152.1, 151.0, 149.8, 149.8, 149.7, 149.7, 149.4, 149.1, 148.4, 147.7, 147.0, 147.0, 146.8, 142.9, 142.0, 142.0, 138.7, 132.3, 131.5, 130.3, 130.2, 130.2, 129.6, 129.5, 128.6, 122.3, 122.2, 122.1, 122.1, 122.1, 121.7, 121.4, 119.7, 114.4, 55.8, 44.3, 44.3, 43.5, 42.9, 40.7, 31.0, 30.8, 21.8, 21.8. HRMS (ESI): m/z $[M+H]^+$ calcd for $C_{75}H_{69}N_2O_{20}S_5$ 1477.3042; found 1477.3044.

4.2. Bi-directional Synthesis

***{B-A-B}** Trimeric bisimidazylate (**38**)*

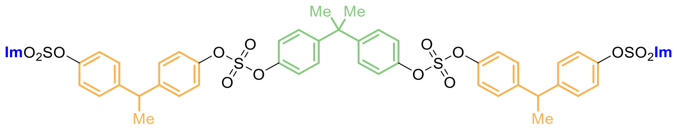

The title compound was prepared according to General Procedure E using **2** (1.38 g, 3.00 mmol, 2.0 equiv), **36** (589 mg, 1.50 mmol, 1.0 equiv), and DBU (57 µL 0.38 mmol, 0.25 equiv) in anhydrous MeCN (7 mL). After stirring for 2 h at 80 ˚C, the mixture was purified by flash column chromatography (hexanes : ethyl acetate = 1 : 2) to afford a white solid (1.52 g, 97%). mp: 50 – 52 ˚C. ^{1}H NMR (400 MHz, DMSO-d_6) δ 8.20 – 8.16 (m, 2H), 7.83 – 7.77 (m, 2H), 7.45 – 7.30 (m, 20H), 7.26 – 7.22 (m, 2H), 7.02 – 6.95 (m, 4H), 4.27 (q, J = 7.1 Hz, 2H), 1.66 (s, 6H), 1.55 (d, J = 7.2 Hz, 6H). ^{13}C NMR (101 MHz, DMSO-d_6) δ 149.6, 148.2, 147.8, 146.9, 146.3, 145.6, 138.1, 131.4, 129.4, 129.3, 128.6, 121.1, 121.0, 120.6, 119.1, 42.7, 42.3, 30.2, 21.2. HRMS (ESI): m/z $[M+H]^+$ calcd for $C_{49}H_{45}N_4O_{14}S_4$ 1041.1810; found 1041.1811.

*{**B-A-B**} Trimeric bisfluorosulfate (**39**)*

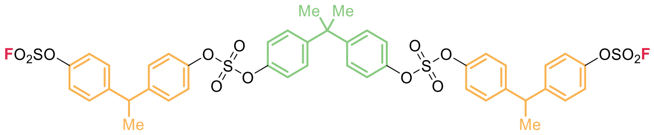

The title compound was prepared according to General Procedure F using **38** (1.24 g, 1.19 mmol, 1.0 equiv) and AgF (449 mg, 3.54 mmol, 3.0 equiv) in anhydrous MeCN (5 mL). After stirring for 16 h at 80 ˚C, the mixture was purified by flash column chromatography (hexanes : ethyl acetate = 3 : 1 to 2 : 1) to afford a white solid (986 mg, 88%). mp: 51 – 52 ˚C. ^{1}H NMR (400 MHz, DMSO-d_6) δ 7.54 – 7.46 (m, 8H), 7.46 – 7.40 (m, 4H), 7.39 – 7.31 (m, 12H), 4.33 (q, J = 7.2 Hz, 2H), 1.66 (s, 6H), 1.59 (d, J = 7.2 Hz, 6H). ^{13}C NMR (101 MHz, DMSO-d_6) δ 149.6, 148.2, 148.0, 147.8, 146.9, 145.5, 129.6, 129.3, 128.6, 121.1, 121.1, 120.7, 42.7, 42.3, 30.2, 21.2. ^{19}F NMR (376 MHz, DMSO-d_6) δ 35.7. HRMS (ESI): m/z [M+Na]$^+$ calcd for $C_{43}H_{38}F_2NaO_{14}S_4$ 967.1005; found 967.1008.

*{**C-B-A-B-C**} Pentameric bisimidazylate (**40**)*

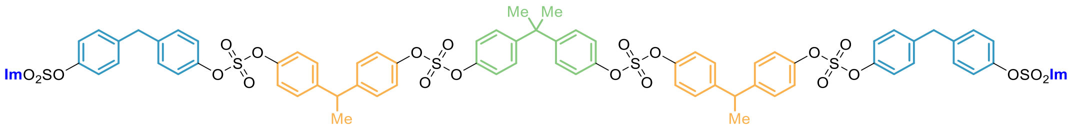

The title compound was prepared according to General Procedure E using **39** (863 mg, 0.91 mmol, 1.0 equiv), **5** (809 mg, 1.82 mmol, 2.0 equiv), and DBU (34 µL 0.23 mmol, 0.25 equiv) in anhydrous MeCN (5 mL). After stirring for 2 h at 80 ˚C, the mixture was purified by flash column chromatography (hexanes : ethyl acetate : DCM = 1 : 1 : 1 to 1 : 2 : 1) to afford a white solid (1.32 g, 93%). mp: 63 – 65 ˚C. ^{1}H NMR (400 MHz, CD_3CN) δ 7.78 – 7.76 (m, 2H), 7.43 – 7.39 (m, 2H), 7.35 – 7.18 (m, 36H), 7.13 – 7.11 (m, 2H), 6.95 – 6.86 (m, 4H), 4.25 (q, J = 7.2 Hz, 2H), 3.97 (s, 4H), 1.65 (s, 6H), 1.58 (d, J = 7.2 Hz, 6H). ^{13}C NMR (101 MHz, CD_3CN) δ 151.0, 149.8, 149.7, 149.7, 149.4, 148.5, 146.9, 142.7, 141.8, 138.7, 132.3, 131.6, 131.5, 130.2, 129.6, 122.4, 122.2, 122.1, 121.6, 119.7, 44.3, 43.5, 40.7, 30.8, 21.8. HRMS (ESI): m/z [M+H]$^+$ calcd for $C_{75}H_{65}N_4O_{22}S_6$ 1565.2409; found 1565.2412.

*{**C-B-A-B-C**} Pentameric bisfluorosulfate (**41**)*

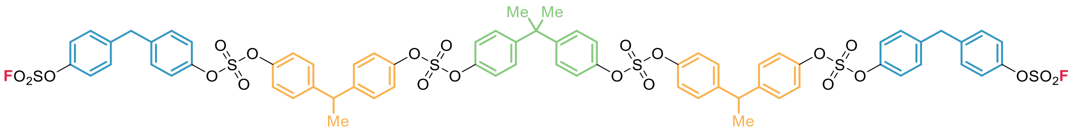

The title compound was prepared according to General Procedure F using **40** (1.14 g, 0.73 mmol, 1.0 equiv) and AgF (278 mg, 2.19 mmol, 3.0 equiv) in anhydrous MeCN (3.5 mL). After stirring for 16 h at 80 ˚C, the mixture was purified by flash column chromatography (hexanes : ethyl acetate : DCM = 4 : 1 : 1) to afford a white solid (1.07 g, 99%). mp: 50 – 51 ˚C. ^{1}H NMR (400 MHz, CD_3CN) δ 7.43 – 7.15 (m, 40H), 4.25 (q, J = 6.7 Hz, 2H), 4.03 (s, 4H), 1.65 (s, 6H), 1.58 (d, J = 7.1 Hz, 6H). ^{13}C NMR (101 MHz, CD_3CN) δ 151.0, 149.9, 149.7, 149.7, 149.6, 149.6, 149.4, 147.0, 143.2, 141.8, 131.9, 131.5, 130.2, 129.6, 122.2, 122.1, 122.1, 122.1, 122.0, 121.7, 44.3, 43.5, 40.7, 30.7, 21.8. ^{19}F NMR (376 MHz, CD_3CN) δ 36.0. HRMS (ESI): m/z [M+Na]$^+$ calcd for $C_{69}H_{58}F_2NaO_{22}S_6$ 1491.1604; found 1491.1591.

*4-(4-(((4-(4-(((1H-Imidazol-1-yl)sulfonyl)oxy)benzyl)phenoxy)sulfonyl)oxy)benzyl)phenyl 1H-imidazole-1-sulfonate (**42**)*

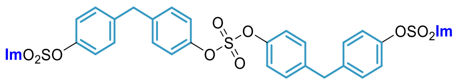

The title compound was prepared according to General Procedure E using **5** (889 mg, 2.00 mmol, 1.0 equiv), **37** (825 mg, 2.00 mmol, 1.0 equiv), and DBU (75 µL, 0.50 mmol, 0.25 equiv) in anhydrous MeCN (7 mL). After stirring for 2 h at 80 ˚C, the mixture was purified by flash column chromatography (hexanes : ethyl acetate = 1 : 2) to afford a colorless viscous oil (1.41 g, 98%). ^{1}H NMR (400 MHz, CD_3CN) δ 7.80 – 7.78 (m, 2H), 7.45 – 7.42 (m, 2H), 7.33 – 7.21 (m, 12H), 7.15 – 7.12 (m, 2H), 6.96 – 6.90 (m, 4H), 4.01 (s, 4H). ^{13}C NMR (101 MHz, CD_3CN) δ 149.8, 148.5, 142.8, 141.9, 138.7, 132.3, 131.7, 131.5, 122.4, 122.2, 119.7, 40.7. HRMS (ESI): *m/z* [M+Cl]$^-$ calcd for $C_{32}H_{26}ClN_4O_{10}S_3$ 757.0505; found 757.0507.

*(((Sulfonylbis(oxy))bis(4,1-phenylene))bis(methylene))bis(4,1-phenylene) bis(fluorosulfate) (**43**)*

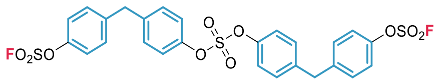

The title compound was prepared according to General Procedure F using **42** (1.28 g, 1.77 mmol, 1.0 equiv) and AgF (674 mg, 5.31 mmol, 3.0 equiv) in anhydrous MeCN (7 mL). After stirring for 16 h at 80 ˚C, the mixture was purified by flash column chromatography (hexanes : ethyl acetate = 4 : 1) to afford a white solid (918 mg, 83%). mp: 93 – 94 ˚C. ^{1}H NMR (400 MHz, CD_3CN) δ 7.38 (s, 8H), 7.35 – 7.26 (m, 8H), 4.05 (s, 4H). ^{13}C NMR (101 MHz, CD_3CN) δ 149.9, 149.6, 143.2, 141.8, 131.9, 131.6, 122.3, 122.1, 40.7. ^{19}F NMR (376 MHz, CD_3CN) δ 36.0. HRMS (ESI): *m/z* [M+Cl]$^-$ calcd for $C_{26}H_{20}ClF_2O_{10}S_3$ 660.9881; found 660.9880.

***{A-C-C-A}** Tetrameric bisimidazylate (**44**)*

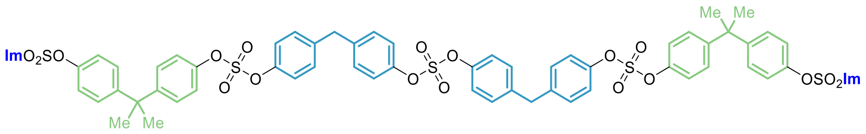

The title compound was prepared according to General Procedure E using **43** (825 mg, 1.32 mmol, 1.0 equiv), **8** (1.25 g, 2.64 mmol, 2.0 equiv), and DBU (49 µL 0.33 mmol, 0.25 equiv) in anhydrous MeCN (4 mL). After stirring for 2 h at 80 ˚C, the mixture was purified by flash column chromatography (hexanes : ethyl acetate : DCM = 2 : 1 : 1 to 1 : 1 : 1) to afford a white solid (1.71 g, 99%). mp: 55 – 56 ˚C. ^{1}H NMR (400 MHz, CD_3CN) δ 7.81 – 7.76 (m, 2H), 7.43 – 7.39 (m, 2H), 7.35 – 7.20 (m, 28H), 7.14 – 7.10 (m, 2H), 6.93 – 6.86 (m, 4H), 4.02 (s, 4H), 1.63 (s, 12H). ^{13}C NMR (101 MHz, CD_3CN) δ 151.8, 151.0, 149.8, 149.8, 149.4, 148.1, 142.0, 138.7, 132.2, 131.5, 129.7, 129.6, 122.2, 122.2, 121.9, 121.6, 119.7, 43.5, 40.7, 30.8. HRMS (ESI): *m/z* [M+Cl]$^-$ calcd for $C_{62}H_{54}ClN_4O_{18}S_5$ 1337.1731; found 1337.1732.

***{A-C-C-A}** Tetrameric bisfluorosulfate (**45**)*

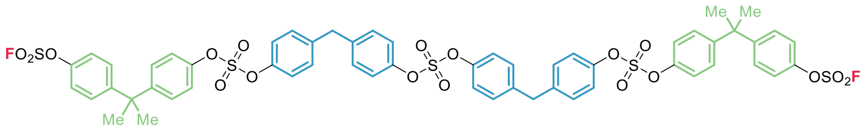

The title compound was prepared according to General Procedure F using **44** (1.70 g, 1.30 mmol, 1.0 equiv) and AgF (495 mg, 3.90 mmol, 3.0 equiv) in anhydrous MeCN (4 mL). After stirring for 16 h at 80 ˚C, the mixture was purified by flash column chromatography (hexanes : ethyl acetate : DCM = 6 : 1 : 1) to afford a white solid (1.51 g, 96%). mp: 46 – 50 ˚C. ^{1}H NMR (400 MHz, CD_3CN) δ 7.46 – 7.14 (m, 32H), 4.02 (s, 4H), 1.67 (s, 12H). ^{13}C NMR (101 MHz, CD_3CN) δ 152.2, 150.8, 149.8, 149.8, 149.4, 149.2, 149.2, 142.0, 142.0, 131.5, 130.0, 129.6, 122.2, 121.7, 121.5, 121.5, 43.6, 40.7, 30.7. ^{19}F NMR (376 MHz, CD_3CN) δ 36.0. HRMS (ESI): *m/z* $[M+Cl]^-$ calcd $C_{56}H_{48}ClF_2O_{18}S_5$ 1241.1106; found 1241.1108.

*4-(2-(4-(((4-(2-(4-(((1H-Imidazol-1-yl)sulfonyl)oxy)phenyl)propan-2-yl)phenoxy)sulfonyl)oxy)phenyl)propan-2-yl)phenyl 1H-imidazole-1-sulfonate (**46**)*

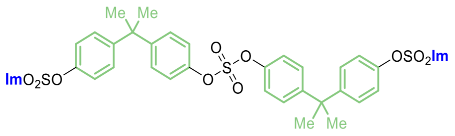

The title compound was prepared according to General Procedure E using **8** (945 mg, 2.00 mmol, 1.0 equiv), **S-7** (881 mg, 2.00 mmol, 1.0 equiv), and DBU (75 µL, 0.50 mmol, 0.25 equiv) in anhydrous MeCN (7 mL). After stirring for 2 h at 80 ˚C, the mixture was purified by flash column chromatography (hexanes : ethyl acetate = 2 : 1 to 1 : 1) to afford a colorless viscous oil (1.53 g, 98%). ^{1}H NMR (400 MHz, CD_3CN) δ 7.84 – 7.72 (m, 2H), 7.49 – 7.38 (m, 2H), 7.38 – 7.17 (m, 13H), 7.17 – 7.05 (m, 2H), 6.95 – 6.87 (m, 3H), 1.65 (s, 12H). ^{13}C NMR (101 MHz, CD_3CN) δ 151.8, 151.0, 149.4, 148.1, 138.7, 132.2, 129.7, 129.6, 121.9, 121.6, 119.7, 43.6, 30.8. HRMS (ESI): *m/z* $[M+H]^+$ calcd for $C_{36}H_{35}N_4O_{10}S_3$ 779.1510; found 779.1510.

*(((Sulfonylbis(oxy))bis(4,1-phenylene))bis(propane-2,2-diyl))bis(4,1-phenylene) bis(fluorosulfate) (**47**)*

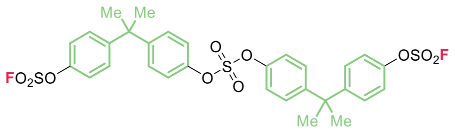

The title compound was prepared according to General Procedure F using **46** (1.39 g, 1.78 mmol, 1.0 equiv) and AgF (677 mg, 5.34 mmol, 3.0 equiv) in anhydrous MeCN (7 mL). After stirring for 16 h at 80 ˚C, the mixture was purified by flash column chromatography (hexanes : ethyl acetate = 8 : 1) to afford a white solid (884 mg, 73%). mp: 108 – 110 ˚C. ^{1}H NMR (400 MHz, CD_3CN) δ 7.43 – 7.38 (m, 4H), 7.38 – 7.31 (m, 8H), 7.31 – 7.25 (m, 4H), 1.69 (s, 12H). ^{13}C NMR (101 MHz, CD_3CN) δ 152.2, 150.8, 149.4, 149.2, 130.0, 129.6, 121.7, 121.5, 43.6, 30.7. ^{19}F NMR (376 MHz, CD_3CN) δ 36.0. HRMS (ESI): *m/z* $[M+Cl]^-$ calcd for $C_{30}H_{28}ClF_2O_{10}S_3$ 717.0507; found 717.0507.

***{A-A-A}** Trimeric bisimidazylate (**48**)*

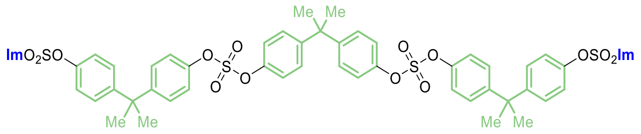

The title compound was prepared according to General Procedure E using **8** (1.89 g, 4.00 mmol, 2.0 equiv), **36** (785 mg, 2.00 mmol, 1.0 equiv), and DBU (75 µL 0.50 mmol, 0.25 equiv) in anhydrous MeCN (8 mL). After stirring for 2 h at 80 ˚C, the mixture was purified by flash column chromatography (hexanes : ethyl acetate : DCM = 1 : 1 : 1) to afford a white solid (2.00 g, 94%). mp: 58 – 60 ˚C. ^{1}H NMR (400 MHz, DMSO-d_6) δ 8.22 – 8.16 (m, 2H), 7.82 – 7.75 (m, 2H), 7.41 – 7.26 (m, 20H), 7.26 – 7.22 (m, 2H), 7.03 – 6.94 (m, 4H), 1.67 (s, 6H), 1.62 (s, 12H). ^{13}C NMR (101 MHz, DMSO-d_6) δ 150.2, 149.6, 149.5, 147.8, 146.7, 138.1, 131.4, 128.7, 128.6, 128.5, 120.8, 120.6, 120.6, 119.1, 42.4, 42.4, 30.2, 30.2. HRMS (ESI): *m/z* [M+Cl]$^-$ calcd for $C_{51}H_{48}ClN_4O_{14}S_4$ 1103.1744; found 1103.1747.

***{A-A-A}** Trimeric bisfluorosulfate (**49**)*

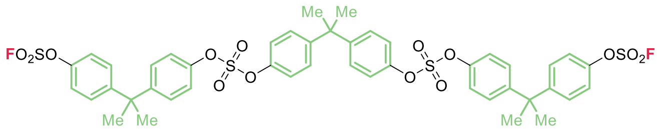

The title compound was prepared according to General Procedure F using **48** (2.00 g, 1.87 mmol, 1.0 equiv) and AgF (712 mg, 5.61 mmol, 3.0 equiv) in anhydrous MeCN (7 mL). After stirring for 16 h at 80 ˚C, the mixture was purified by flash column chromatography (hexanes : ethyl acetate : DCM = 1 : 1 : 1) to afford a white solid (1.66 g, 91%). mp: 124 – 125 ˚C. ^{1}H NMR (400 MHz, DMSO-d_6) δ 7.59 – 7.52 (m, 4H), 7.52 – 7.44 (m, 4H), 7.44 – 7.34 (m, 16H), 1.72 (s, 18H). ^{13}C NMR (101 MHz, DMSO-d_6) δ 150.8, 149.6, 149.4, 147.9, 147.8, 147.7, 128.9, 128.6, 128.6, 120.7, 120.6, 42.4, 42.4, 30.2, 30.1.^{19}F NMR (376 MHz, CD_3CN) δ 35.7. HRMS (ESI): *m/z* [M+Cl]$^-$ calcd for $C_{45}H_{42}ClF_2O_{14}S_4$ 1007.1119; found 1007.1116.

4.3. Tri-directional Synthesis

*Tetrameric trisimidazylate (**53**)*

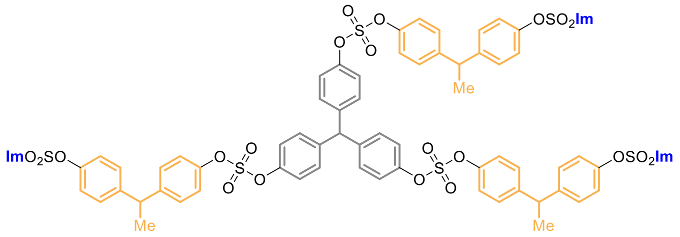

The title compound was prepared according to General Procedure E using **52** (1.08 g, 2.00 mmol, 1.0 equiv), **2** (3.03 g, 6.60 mmol, 3.3 equiv), and DBU (104 µL, 0.70 mmol, 0.35 equiv) in anhydrous MeCN (7 mL). After stirring for 2 h at 80 ˚C, the mixture was purified by flash column chromatography (hexanes : ethyl acetate : DCM = 1 : 1 : 1) to afford a white solid (2.94 g, 97%). mp: 57 – 61 ˚C. ^{1}H NMR (400 MHz, DMSO-d_6) δ 8.22 – 8.13 (m, 3H), 7.84 – 7.74 (m, 3H), 7.50 – 7.17 (m, 33H), 7.05 – 6.89 (m, 6H), 5.91 (s, 1H), 4.27 (q, J = 7.1 Hz, 3H), 1.54 (d, J = 7.2 Hz, 9H). ^{13}C NMR (101 MHz, DMSO-d_6) δ 148.4, 148.2, 147.0, 146.3, 145.6, 142.7, 138.1, 131.4, 130.9,

129.4, 129.3, 121.3, 121.1, 121.1, 119.1, 53.3, 42.7, 21.3. HRMS (ESI): *m/z* $[M+Na]^+$ calcd for $C_{70}H_{58}N_6NaO_{21}S_6$ 1533.1871; found 1533.1869.

*Tetrameric trisfluorosulfate (**54**)*

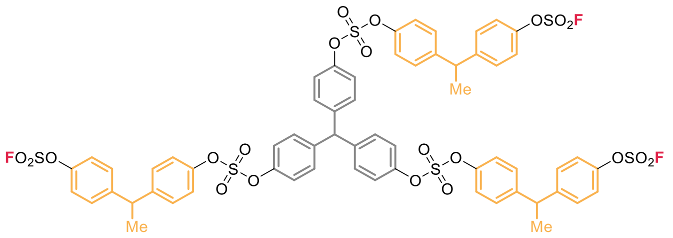

The title compound was prepared according to General Procedure F using **53** (2.92 g, 1.93 mmol, 1.0 equiv) and AgF (1.10 g, 8.69 mmol, 4.5 equiv) in anhydrous MeCN (6.5 mL). After stirring for 16 h at 80 ˚C, the mixture was purified by flash column chromatography (hexanes : DCM = 1 : 1) to afford a white solid (2.08 g, 79%). mp: 65 – 67 ˚C. ^{1}H NMR (400 MHz, DMSO-d_6) δ 7.53 – 7.45 (m, 12H), 7.45 – 7.39 (m, 12H), 7.39 – 7.32 (m, 6H), 7.32 – 7.25 (m, 6H), 5.90 (s, 1H), 4.33 (q, J = 7.2 Hz, 3H), 1.58 (d, J = 7.2 Hz, 9H). ^{13}C NMR (101 MHz, DMSO-d_6) δ 148.4, 148.2, 148.0, 146.9, 145.5, 142.7, 130.9, 129.6, 129.3, 121.3, 121.1, 121.0, 53.3, 42.7, 21.2. ^{19}F NMR (376 MHz, DMSO-d_6) δ 35.7. HRMS (ESI): *m/z* $[M+Na]^+$ calcd for $C_{61}H_{49}F_3NaO_{21}S_6$ 1389.0935; found 1389.0935.

5. Sequencing of Oligosulfates After Base Hydrolysis

5.1. Degradation Test on Oligosulfate

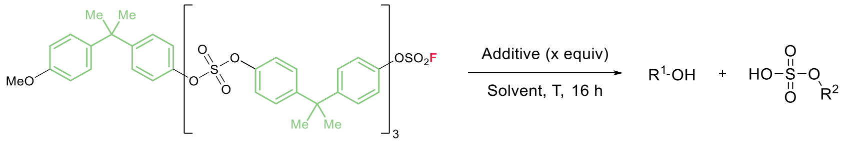

According to the reported research on sulfate linkage,[7-9] the degradation tests were performed under various conditions. The corresponding reagent (see Table S3) was added to a mixture of the presented tetramer in specific solvent. After stirring overnight, the crude mixture (0.1 mL) was added to HPLC-grade methanol (0.9 mL), the mixture was filtered by syringe filter, and analyzed by HRMS (ESI) to detect the expected fragments.

Supplementary Table 3. Degradation Tests with Different Reagents

Entry	Reagent	Solvent	Temperature	Result
1	NaI (15 mg, 0.10 mmol)	DMF	80 ˚C	n/d^a
2	NaN_3 (21 mg, 0.30 mmol)	DMF	80 ˚C	n/d^a
3	AgF (13 mg, 0.10 mmol)	DMF	80 ˚C	n/d^a
4	NaOH (12 mg, 0.30 mmol)	DCM/MeOH(9/1)	rt	Detected^b
5	NaOH (12 mg, 0.30 mmol)	DMF	80 ˚C	Detected^b
6	H_2SO_4 (16 µL, 0.30 mmol)	DMF	80 ˚C	n/d^a
7	TFA (23 µL, 0.30 mmol)	DMF	80 ˚C	n/d^a

* Standard conditions: the presented tetramer (36 mg, 0.03 mmol), reagent, solvent (0.2 mL).
n/d^a: Not detected. Detected^b: Observed fragments

Positive

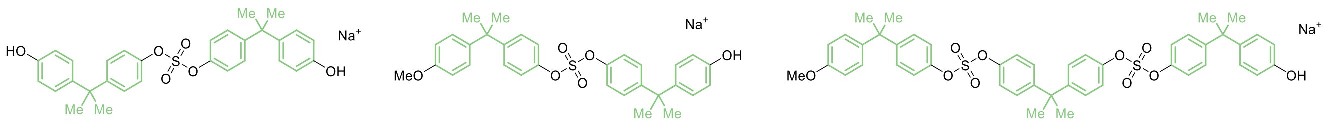

Negative

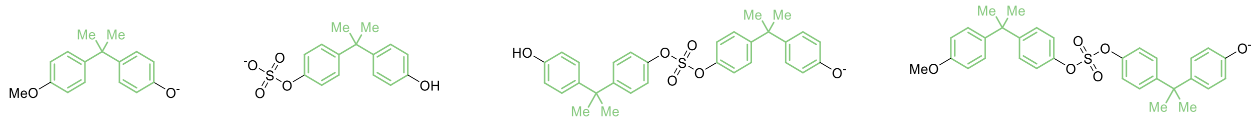

5.2. HRMS (ESI) Analysis for the Fragments of Sequence-Regulated Oligomers

5.2.1. Fragments from 11

*MeO–**{A}**–O*$^-$

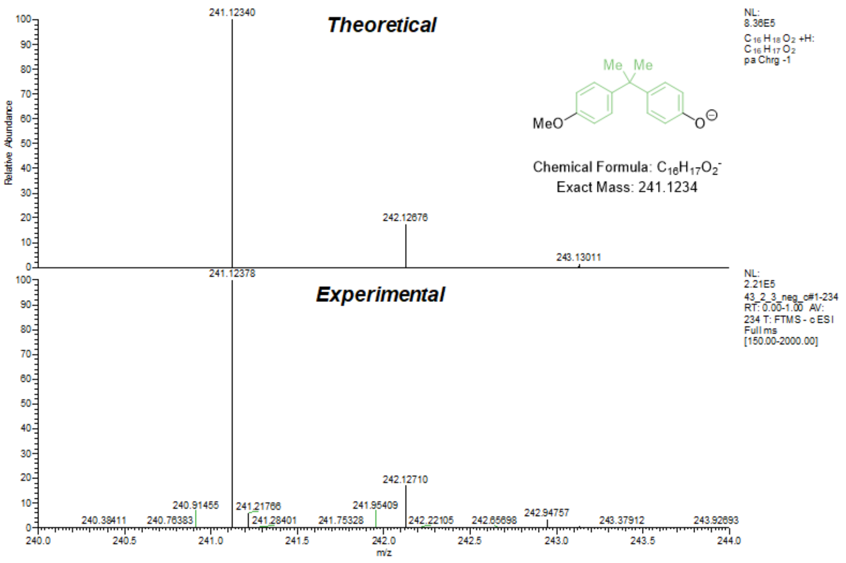

*MeO–**{A-B}**–O*$^-$

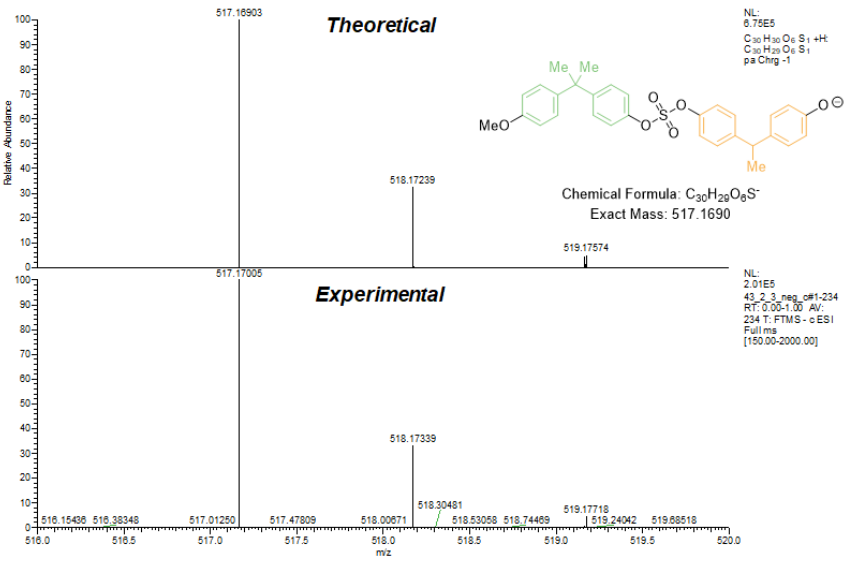

MeO–**{A-B-C}**–*O*$^-$

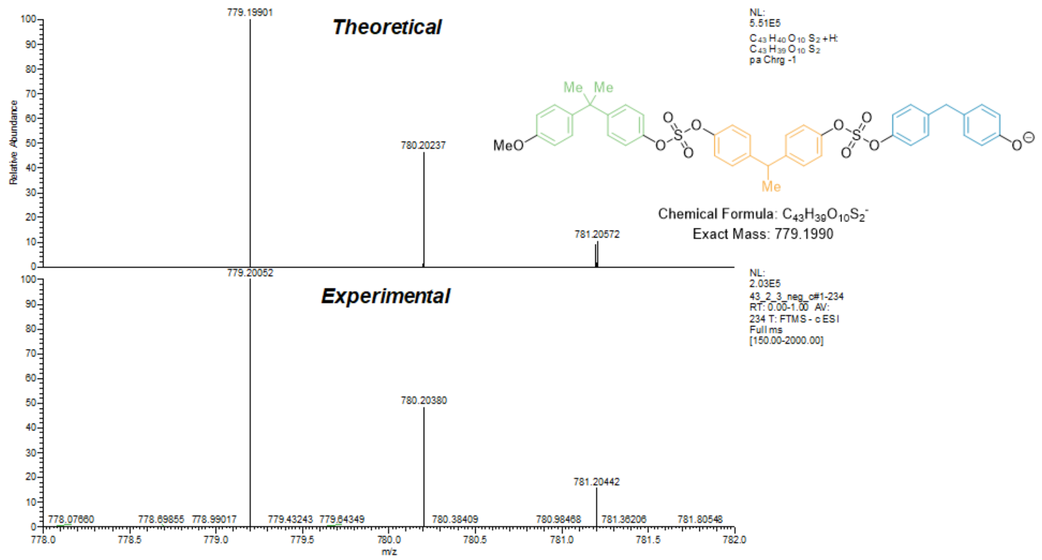

MeO–**{A-B-C-A}**–*O*$^-$

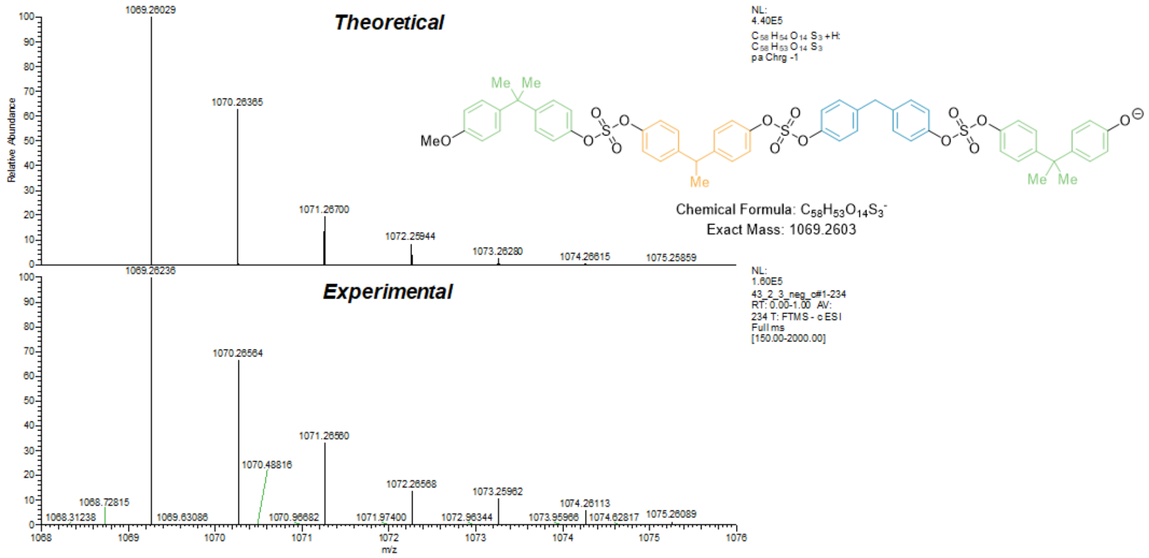

MeO–**{A-B-C-A-B}**–*OSO*$_2$*O*$^-$

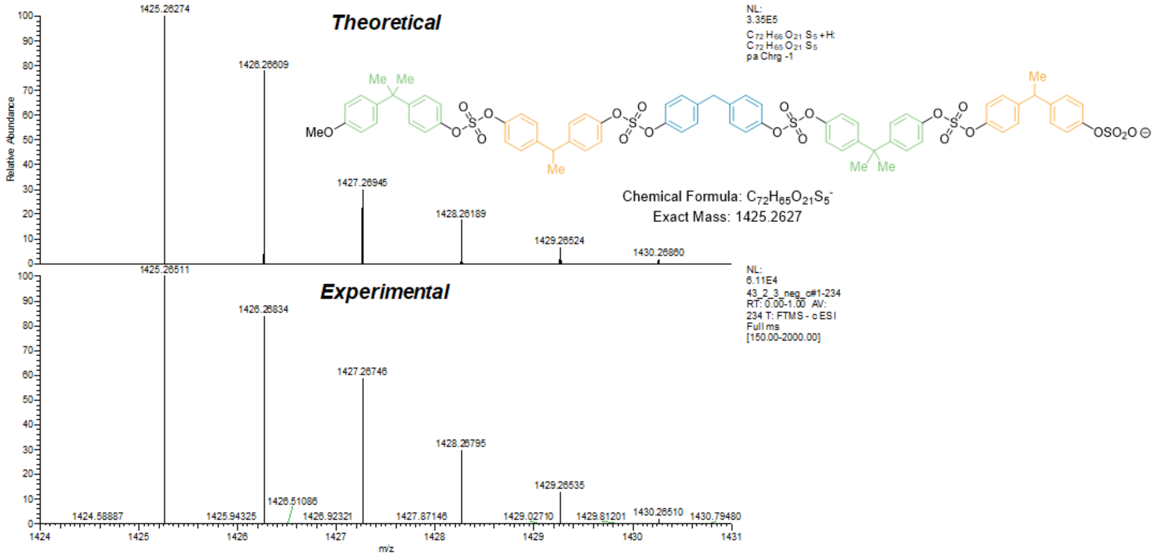

5.2.2. Fragments from 30

MeO–{A}-O⁻

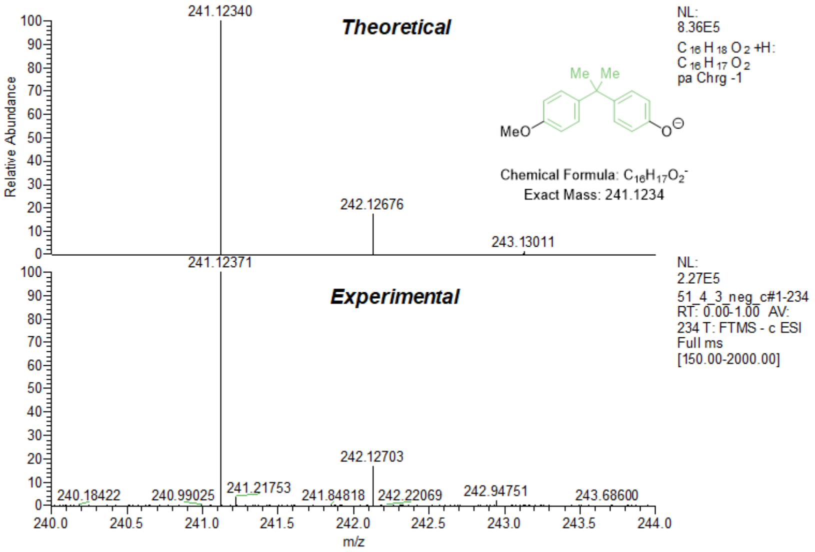

MeO–{A-B}–OH⁻

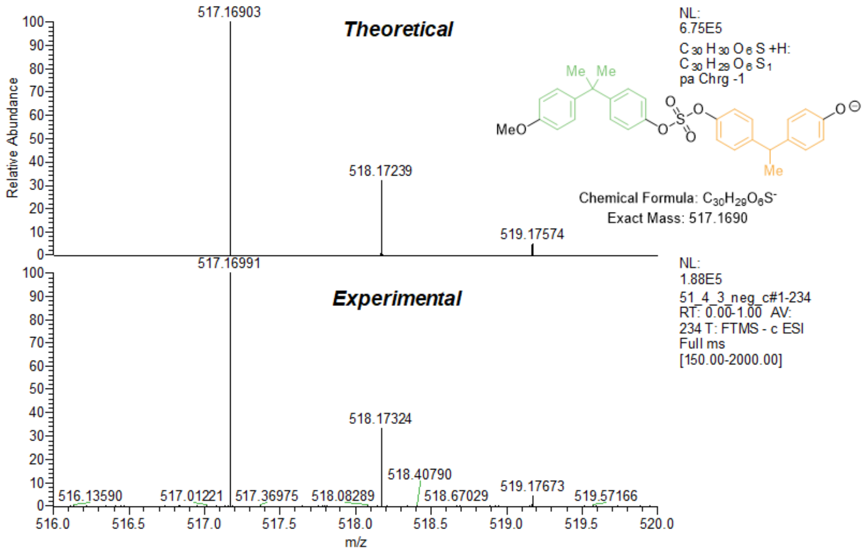

MeO–{A-B-A}–O⁻

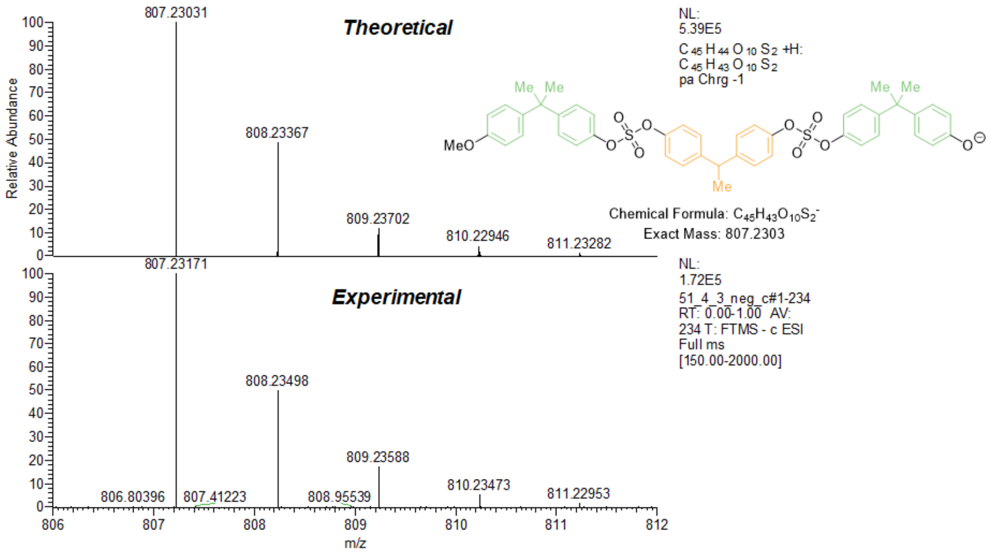

*MeO–{**A-B-A-C**}-OSO$_2$O$^-$*

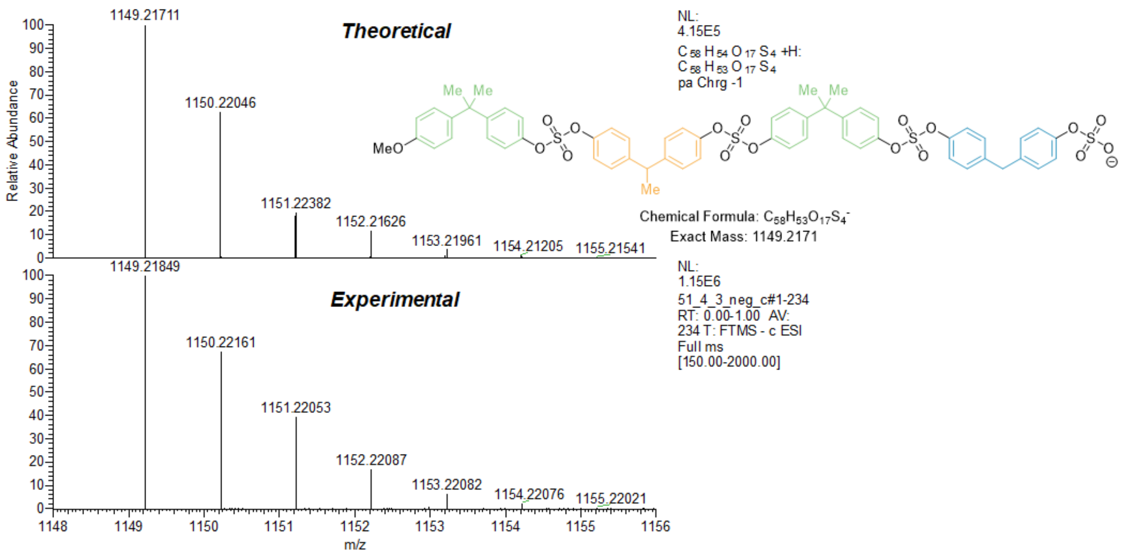

*MeO–{**A-B-A-C-B**}–OSO$_2$O$^-$*

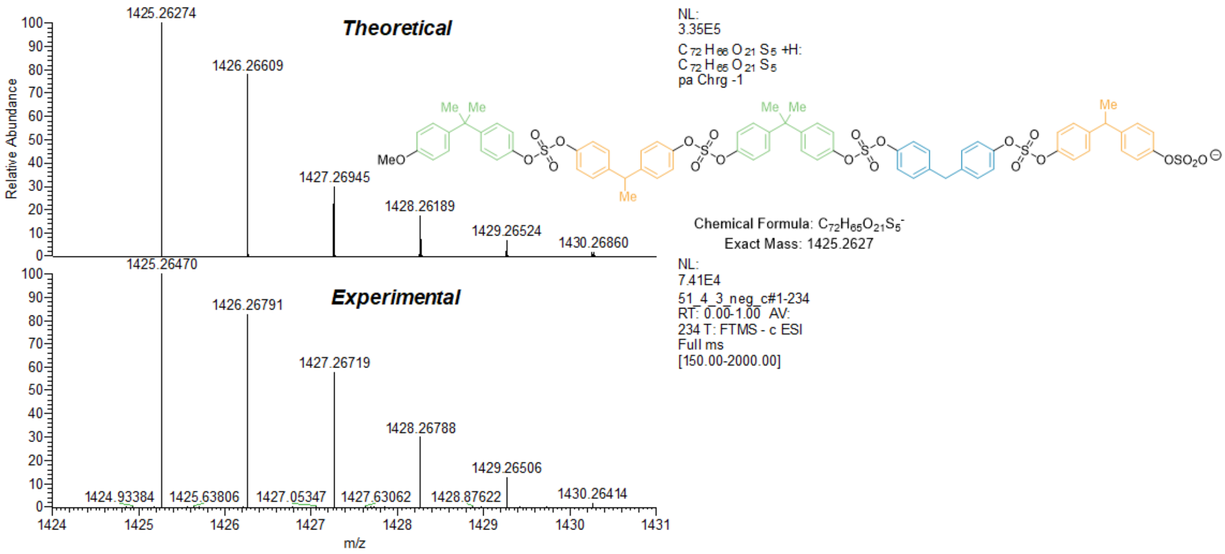

6. Synthesis of Polysulfate Periodic Copolymers

General Polymerization Procedure Polysulfates were prepared with the modified polycondensation procedure based on the previously reported procedure.[6] To a flame-dried 10 mL Schlenk tube with a magnetic stir bar, sequence-regulated bisfluorosulfate monomer (1.0 equiv) and bis(*tert*-butyldimethylsilyl) bisphenol monomer were added. After degassing for 30 min, nitrogen gas was purged, and anhydrous *N*-methyl-2-pyrrolidone (NMP) was added to the mixture and the mixture was pre-heated at 130 ˚C. Then, DBU (20 mol%) as a Lewis base catalyst was added. After stirring for 1 h at 130 ˚C, the mixture was diluted with DMF and precipitated by the addition of methanol. The precipitate was collected by suction filtration, purified by Soxhlet extraction with methanol, and dried overnight inside a vacuum oven at 80 ˚C to give the corresponding polysulfate periodic copolymer.

Polysulfate periodic copolymer **-(A-A-C)- (*P-1*)**

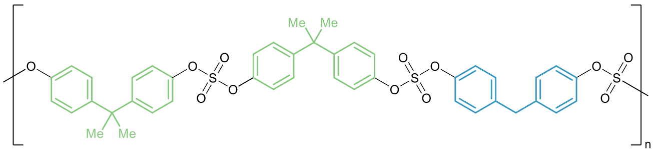

The title polymer was prepared according to General Polymerization Procedure using **47** (683 g, 1.00 mmol, 1.0 equiv) and **50** (429 mg, 1.00 mmol, 1.0 equiv) in anhydrous NMP (1.0 mL). A white solid (718 mg, 85%). ^{1}H NMR (400 MHz, CD_2Cl_2) δ 7.33 – 7.16 (m, 24H), 4.02 (s, 2H), 1.69 (s, 12H). ^{13}C NMR (101 MHz, CD_2Cl_2) δ 150.1, 149.3, 148.9, 140.7, 130.8, 128.9, 121.5, 121.0, 43.1, 40.8, 30.9. M_n = 19 kDa $Đ$ = 1.5.

Polysulfate periodic copolymer **-(A-A-A-C)- (*P-2*)**

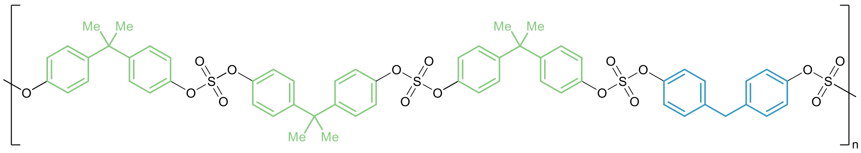

The title polymer was prepared according to General Polymerization Procedure using **49** (973 mg, 1.00 mmol, 1.0 equiv) and **50** (429 mg, 1.00 mmol, 1.0 equiv) in anhydrous NMP (1.0 mL). A white solid (837 mg, 74%). ^{1}H NMR (400 MHz CD_2Cl_2) δ 7.49 – 7.13 (m, 32H), 4.02 (s, 2H), 1.69 (s, 18H). ^{13}C NMR (101 MHz, CD_2Cl_2) δ 150.1, 149.3, 148.9, 140.7, 130.8, 128.9, 121.5, 121.0, 43.1, 40.8, 30.9. M_n = 24 kDa. $Đ$ = 1.7.

Polysulfate periodic copolymer **-(A-A-A-C-C)- (*P-3*)**

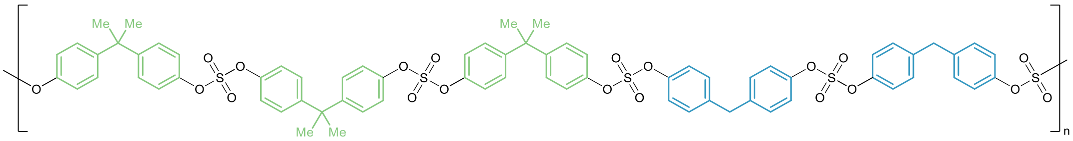

The title polymer was prepared according to General Polymerization Procedure using **45** (1.21 g, 1.00 mmol, 1.0 equiv) and **51** (457 mg, 1.00 mmol, 1.0 equiv) in anhydrous NMP (1.0 mL). A white solid (1.15 g, 82%). ^{1}H NMR (400 MHz, CD_2Cl_2) δ 7.45 – 7.08 (m, 40H), 4.02 (s, 4H), 1.68 (s, 18H). ^{13}C NMR (101 MHz, CD_2Cl_2) δ 150.1, 149.3, 148.9, 140.7, 130.8, 128.9, 121.5, 121.0, 43.1, 40.8, 30.9. M_n = 48 kDa. $Đ$ = 2.0.

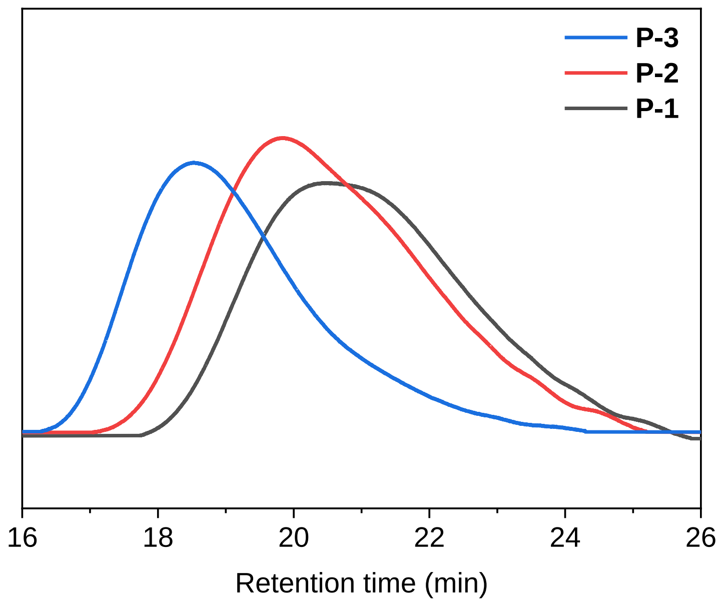

Supplementary Figure 1. GPC Chromatograms of P-1, P-2, and P-3.

7. Hydrolysis of Polysulfates Periodic Copolymer and Analysis of Fragments

7.1. GPC Chromatograms of Hydrolysed Polysulfates Compared to Pristine Polymer

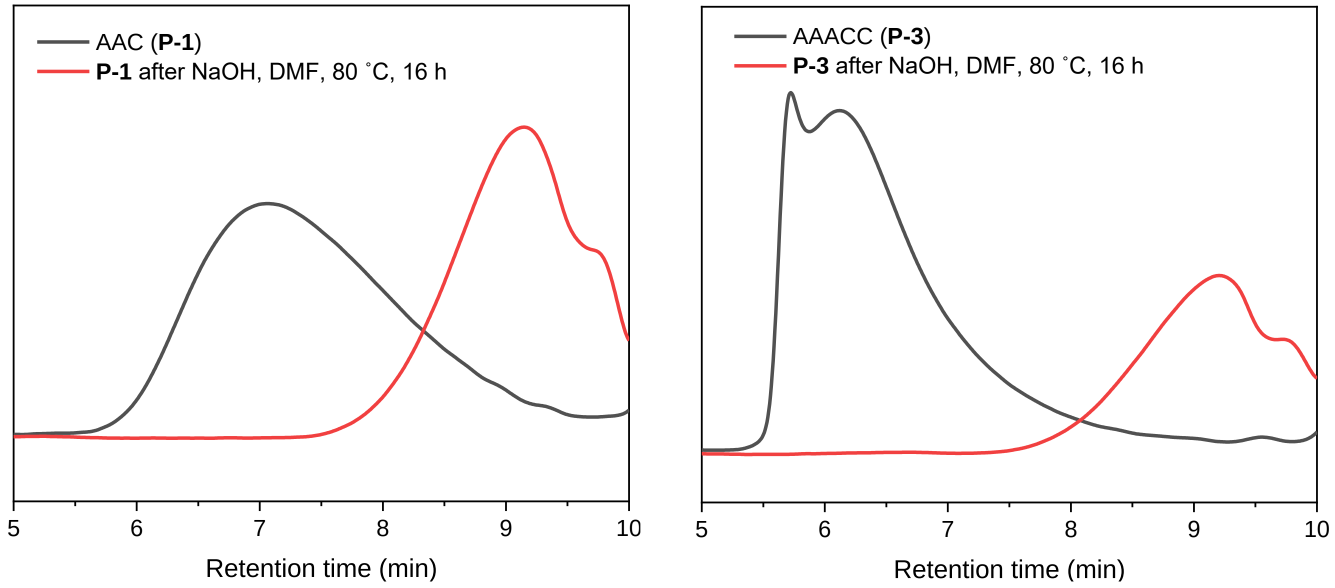

7.2. HRMS (ESI) Analysis for the Fragments from P-2

HO–{C}–OSO$_2$O$^-$

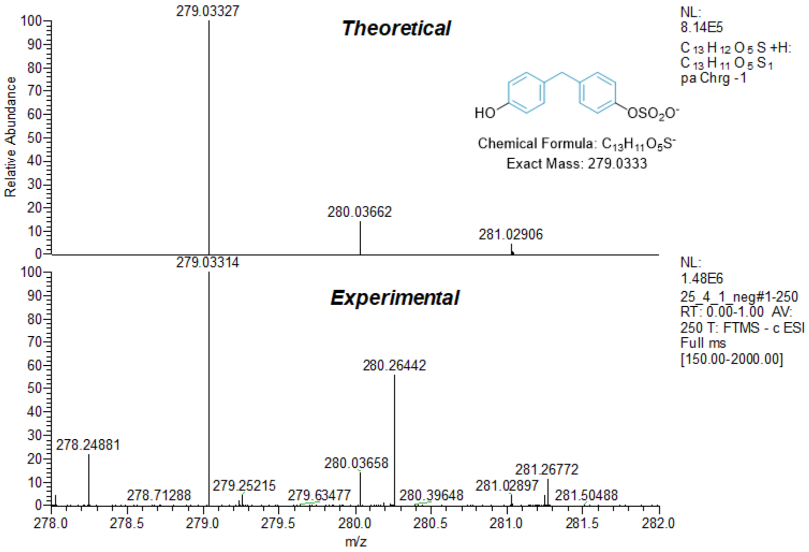

HO–{A}–OSO$_2$O$^-$

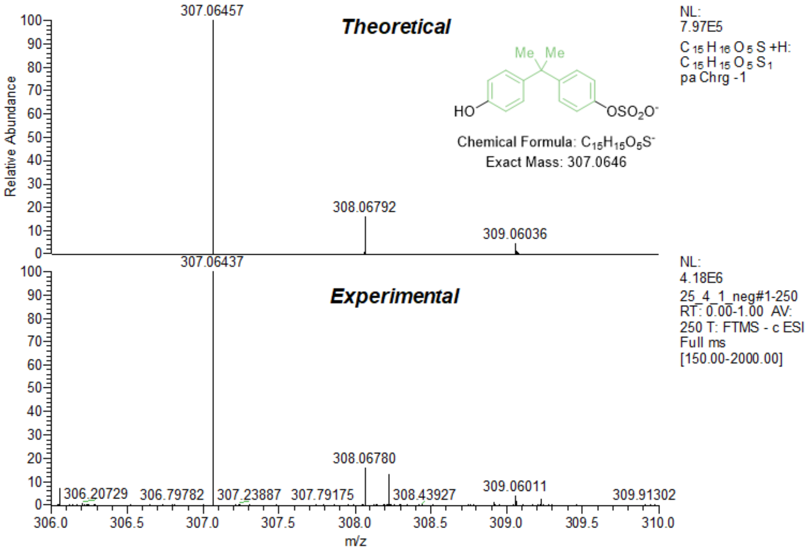

HO–{C-A}–O$^-$

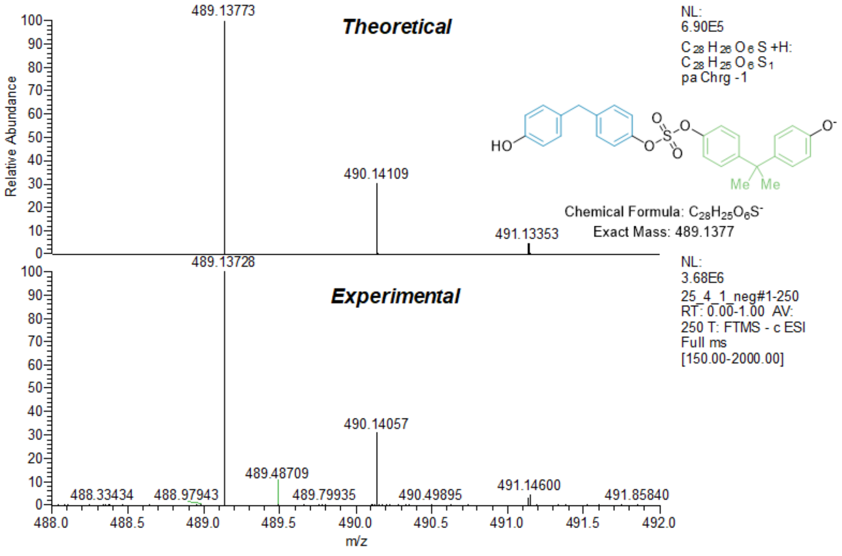

*HO–***{A-A}***–O*$^-$

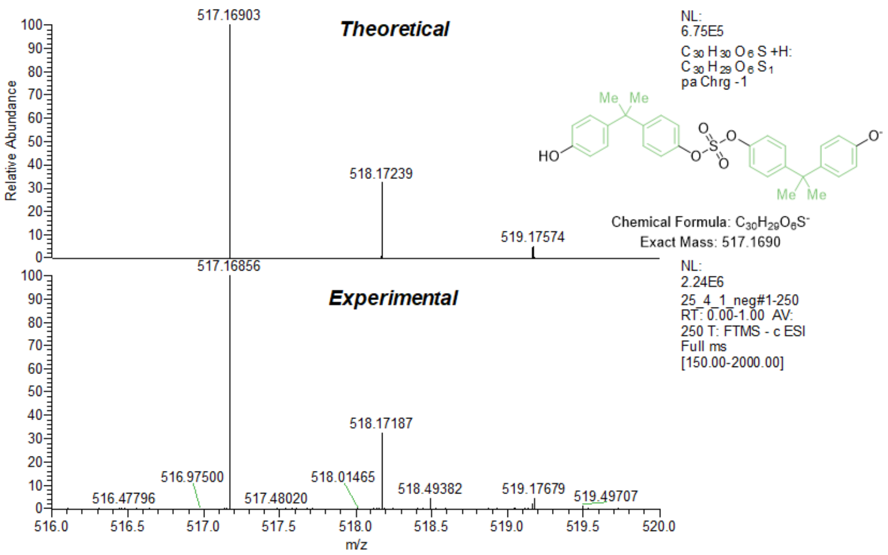

*HO–***{A-A-A}***–O*$^-$

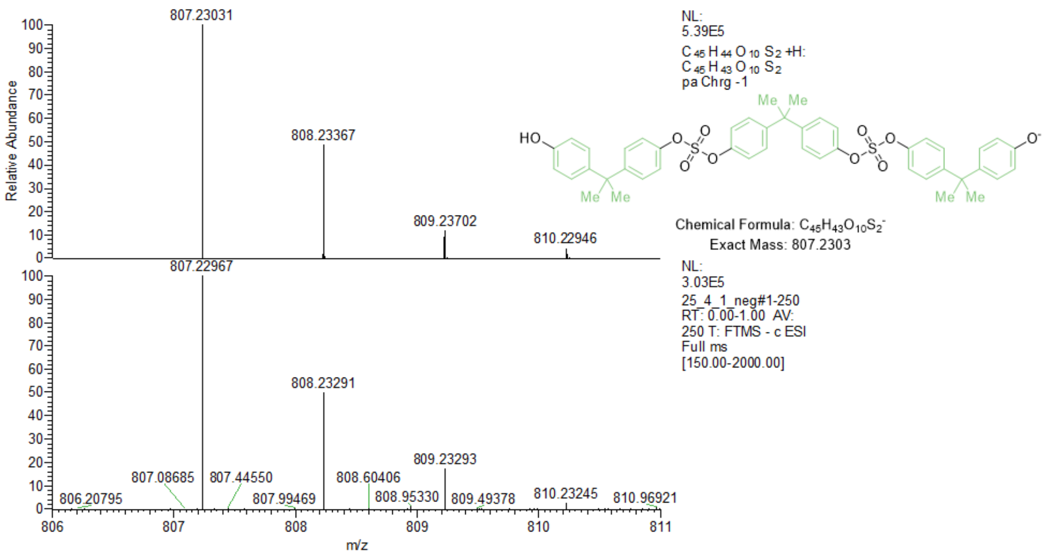

7.3. Degradation Test on P-2

Supplementary Table 4. Degradation Tests Using NaOH, NaI, or PPh_3 on P-2

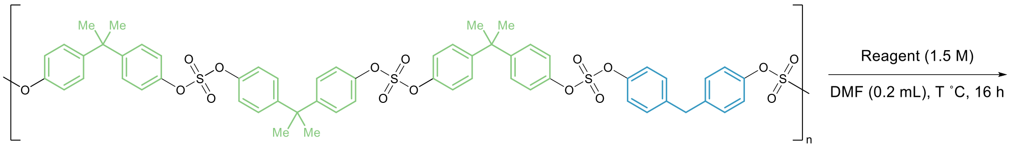

Entry	Reagent	Temperature	Result
1	NaOH (12 mg, 0.30 mmol)	rt	Minor change
2	NaI (45 mg, 0.30 mmol)	rt	No significant change
3	PPh_3 (79 mg, 0.30 mmol)	rt	No significant change
4	NaOH (12 mg, 0.30 mmol)	80 ˚C	Full degradation

* Standard conditions: **P-2** (35 mg, 0.03 mmol), DMF (0.2 mL), 16 h.

Referring to the previous reports on degradation of SuFEx-based polymers,[10] the degradation tests were performed under various conditions. A volume of 6 μL of each mixture was taken and diluted in 1.0 mL of DMF/LiBr solution. Following this, filtration was performed using a syringe filter, and the samples were subjected to analysed via GPC.

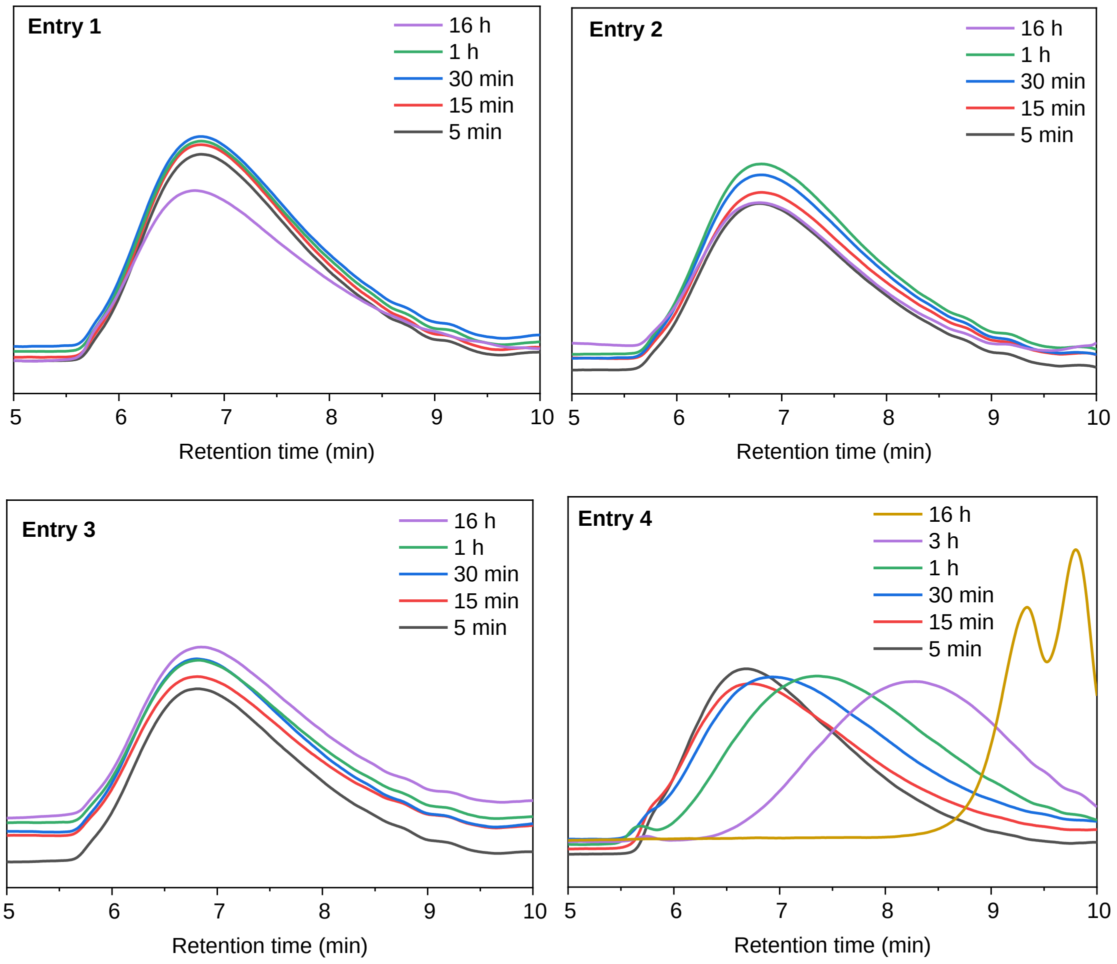

Supplementary Figure 2. GPC Traces.

8. Preliminary Adhesion Test

The adhesive resins were applied to a steel substrate (type: CR340, length: 100 mm, width: 25 mm, thickness: 1.6 mm) in an adhesion area of 25.4 mm × 25.4 mm, followed by curing. The thickness of the adhesive layer was approximately maintained 250 micron by using glass beads as spacer. The single lap shear strength represented the maximum stress required to destroy the bonded specimen. Stress (MPa) was calculated by dividing the applied force by the adhesion area (25.4 mm × 25.4 mm) and was evaluated using Instron 5982 universal testing machine (UTM) with 10 kN load cell. Lap shear strength was repeated three times, and all experiments were conducted at rt. Lap shear strength was calculated according to the following equation: $\tau = \frac{F}{A}$ (eq. 1), where τ is lap-shear strength (MPa), F is the maximum loading force to break the joint (N), and A is the specimen area (645.16 mm^2).

8.1. Adhesion Test of Linear Polysulfates

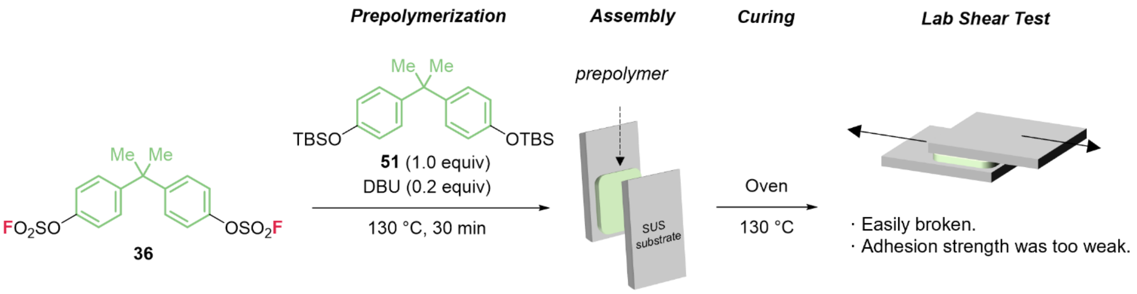

A mixture of **36** (392 mg, 1.00 mmol), **51** (457 mg, 1.00 mmol) and DBU (30 µL, 0.20 mmol) was stirred for 30 min at 130 ˚C for prepolymerization. Then, the resulting prepolymer resin was evenly applied to SUS specimens, and adhesive samples were fabricated by curing for 2 h at 130 ˚C. The adhesive samples with linear polysulfates were broken instantly when lab shear test using UTM was initiated.

8.2. Adhesion Test of P-4 & P-5

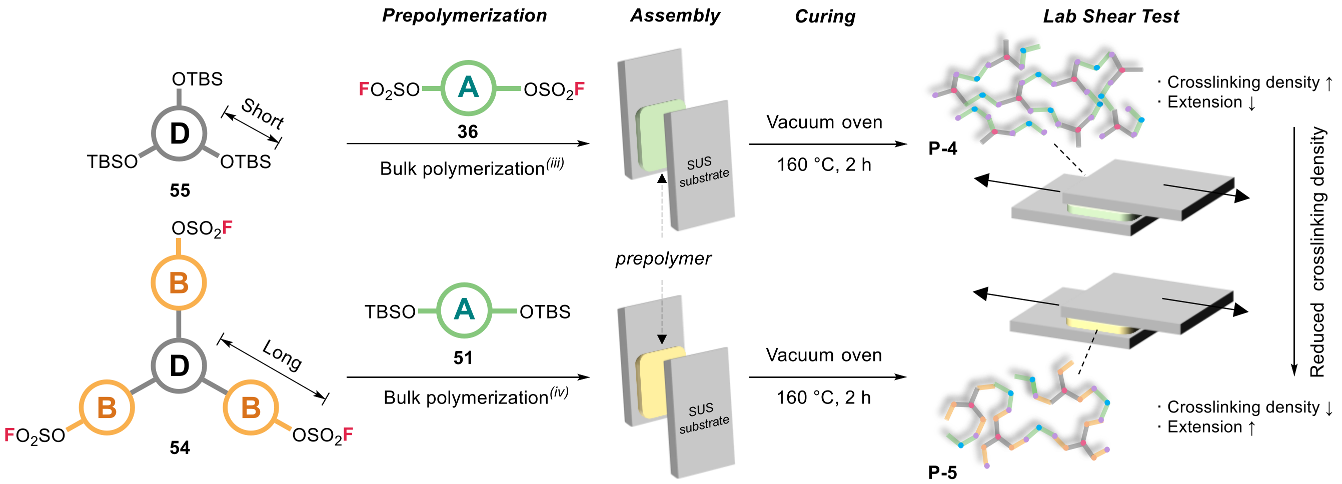

Supplementary Table 5. Experimental Data of Adhesion Test on P-4

Entry	Force (N)	Lab Shear Strength (MPa)
1	1556.32	2.41
2	1968.61	3.05
3	1237.97	1.92
Average	-	2.46
Standard deviation (95% CI)	-	0.52

Supplementary Table 6. Experimental Data of Adhesion Test on P-5

Entry	Force (N)	Lab Shear Strength (MPa)
1	1706.13	2.64
2	1928.44	2.99
3	1505.04	2.33
Average	-	2.66
Standard Deviation (95% CI)	-	0.30

9. Iterative Synthesis through Protecting Group Approach

After searching mild acid and basic conditions in the literature,[11,12] iterative synthesis was conducted to obtain **{A-A-C}** trimeric fluorosulfate **22** using the acetyl protecting group. Thus, the building block **5'** and **8'** were utilized. The fluorosulfate **1** was reacted with silyl ether **8'** through a SuFEx coupling reaction to afford **19'** in 94% yield. Subsequently, the acetyl group was deprotected under acidic- or basic conditions to yield **19''** in 50% or 79% yields, respectively. Then, SO_2F moiety was installed on **19''** using SuFEx-IT to give **{A-A}** dimeric fluorosulfate **20**.[13] By repeating these processes, **{A-A-C}** trimeric fluorosulfate **22** was obtained in 28% overall yield from acidic deprotection-associated path and 31% overall yield from basic deprotection-associated path over 6 steps.

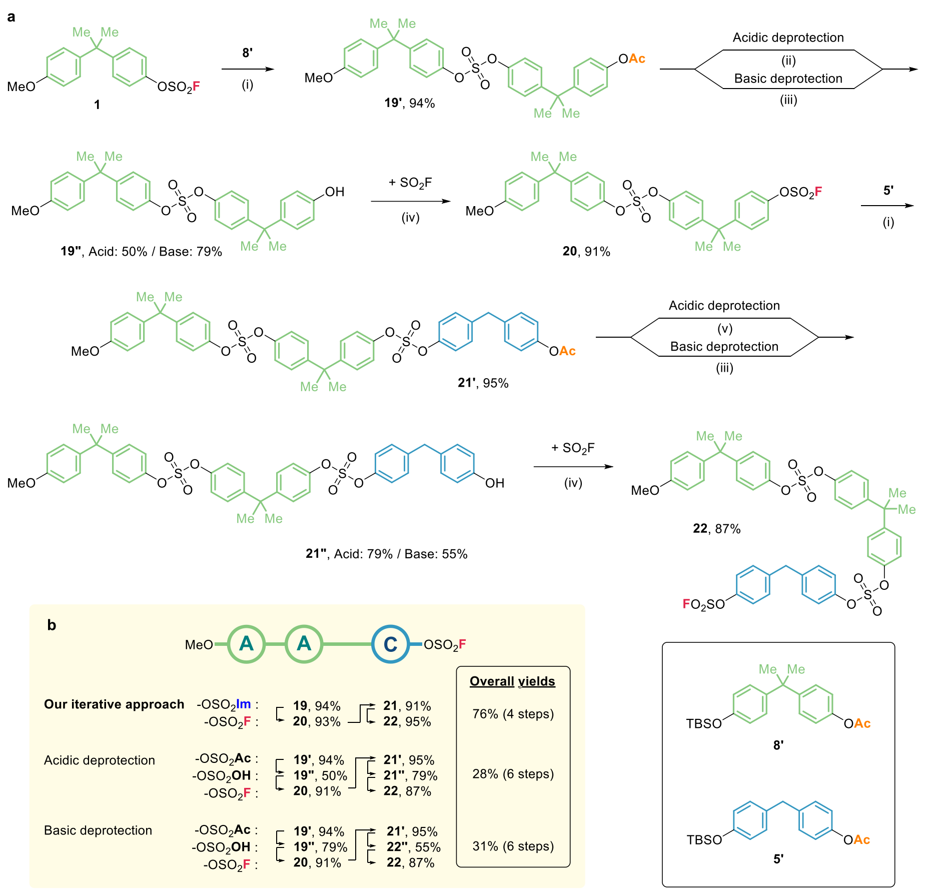

Supplementary Figure 3. Iterative Synthesis of Sequence-regulated Oligosulfates through Protection and Deprotection Using Acetyl Group. a Synthetic scheme. **b** Overall comparison. Reaction conditions: (i) fluorosulfate (1.0 equiv), building block (1.0 equiv), DBU (25 mol %), MeCN, 80 ˚C, 2 h; (ii) AcCl (15 mol %), MeOH (0.9 M), rt , 3 h; (iii) K_2CO_3 (2.5 equiv), MeOH, rt, 4 h; (iv) 1-(fluorosulfonyl)-2,3-dimethyl-1*H*-imidazol-3-ium trifluoromethanesulfonate (SuFEx-IT) (1.3 equiv), Et_3N (1.6 equiv), MeCN, 0 ˚C to rt, 16 h; (v) AcCl (15 mol %), MeOH (0.5 M), rt, 16 h.

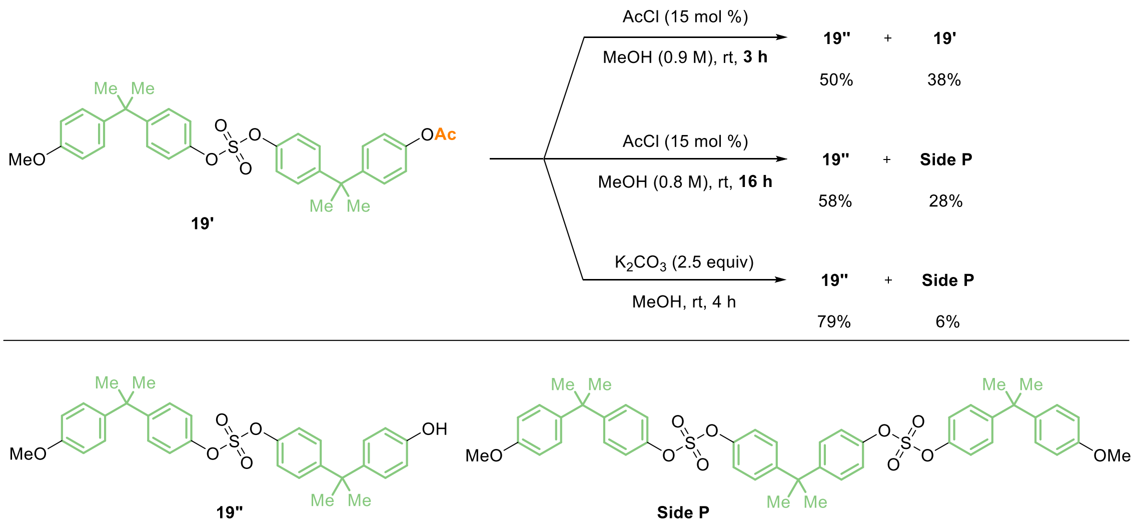

Supplementary Figure 4. Formation of Side P by Acidic and Basic Deprotection Steps.

We also investigated to side reaction during the deprotection step, prompted by the low yields depicted in Supplementary Figure 3. When dimer **19'** was deprotected under acidic conditions,[11] the substrate remained unacted in 38%. Extending reaction time to 16 h increased the yield of deacetylated product **19''**, however, a side product (**Side P**) was obtained in 28% yield. Deacetylation under basic conditions also gave **Side P** in 6% yield. The formation of undesired **Side P** is likely due to the sulfate cleave of substrate **19'** followed by the condensation between the cleaved one with the substrate **19'**. The formation of the side product is detrimental for the synthesis of sequence-regulated structures.

General Procedure G. Acetyl chloride in DCM was added dropwise to a mixture of monosubstituted bisphenol silyl ether and K_2CO_3 in DCM at 0 ˚C. After stirring for 16 h at rt, the mixture was concentrated *in vacuo* and purified by flash column chromatography to afford the corresponding bisphenol derivatives.

General Procedure H. Acetyl chloride was added to a mixture of acetate in methanol. After stirring for 3 h at rt, the mixture was concentrated *in vacuo* and purified by flash column chromatography to afford the corresponding oligomeric alcohol.

General Procedure I. K_2CO_3 was added to a mixture of acetate in methanol. After stirring for 4 h at rt, the mixture was filtered by vacuum filtration. The filtrate was concentrated *in vacuo* and purified by flash column chromatography to afford the corresponding oligomeric alcohol.

*4-(4-((Tert-butyldimethylsilyl)oxy)benzyl)phenyl acetate (**5'**)*

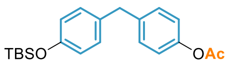

The title compound was prepared according to General Procedure G using **S-3** (6.25 g, 20.0 mmol, 1.0 equiv), AcCl (1.43 mL, 20.0 mmol, 1.0 equiv), and K_2CO_3 (4.15 g, 30.0 mmol, 1.5 equiv) in DCM (100 mL). After stirring for 16 h at rt, the mixture was concentrated via rotary evaporator and purified by flash column chromatography (hexanes : ethyl acetate = 7 : 1) to afford a colorless oil (1.26 g, 18%). ^{1}H NMR (400 MHz, $CDCl_3$) δ 7.21 – 7.12 (m, 2H), 7.08 – 6.93 (m, 4H), 6.81 – 6.71 (m, 2H), 3.90 (s, 2H), 2.29 (s, 3H), 0.98 (s, 9H), 0.19 (s, 6H). ^{13}C NMR (101 MHz, $CDCl_3$) δ 169.7, 154.1, 149.0, 139.3, 133.5, 130.0, 129.8, 121.5, 120.1, 40.6, 25.8, 21.3, 18.3, -4.3. HRMS (ESI): *m/z* $[M+Na]^+$ calcd for $C_{21}H_{28}NaO_3Si$ 379.1700; found 379.1708.

*4-(2-(4-((Tert-butyldimethylsilyl)oxy)phenyl)propan-2-yl)phenyl acetate (**8'**)*

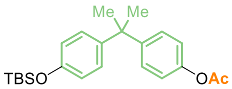

The title compound was prepared according to General Procedure G using **S-4** (3.70 g, 11.0 mmol, 1.0 equiv), AcCl (0.79 mL, 11.0 mmol, 1.0 equiv), and K_2CO_3 (2.28 g, 16.5 mmol, 1.5 equiv) in DCM (55 mL). After stirring for 16 h at rt, the mixture was concentrated via rotary evaporator and purified by flash column chromatography (hexanes : ethyl acetate = 9 : 1) to afford a colorless oil (1.80 g, 43%). ^{1}H NMR (400 MHz, $CDCl_3$) δ 7.25 – 7.19 (m, 2H), 7.11 – 7.05 (m, 2H), 7.01 – 6.94 (m, 2H), 6.78 – 6.70 (m, 2H), 2.28 (s, 3H), 1.65 (s, 6H), 0.99 (s, 9H),

0.20 (s, 6H). ^{13}C NMR (101 MHz, $CDCl_3$) δ 169.7, 153.6, 148.7, 148.5, 143.1, 127.9, 127.8, 120.9, 119.4, 42.2, 31.1, 25.8, 21.3, 18.1, -4.3. HRMS (ESI): *m/z* $[M+Na]^+$ calcd for $C_{23}H_{32}NaO_3Si$ 407.2013; found 407.2019.

*{A-A} Dimeric acetate (**19'**)*

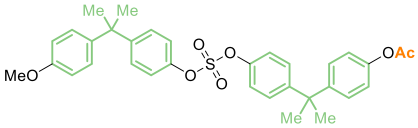

The title compound was prepared according to General Procedure E using **1** (649 mg, 2.00 mmol, 1.0 equiv), **8'** (769 mg, 2.00 mmol, 1.0 equiv), and DBU (75 µL, 0.50 mmol, 0.25 equiv) in anhydrous MeCN (7 mL). After stirring for 2 h at 80 ˚C, the mixture was purified by flash column chromatography (hexanes : ethyl acetate = 5 : 1) to afford a colorless viscous oil (1.08 g, 94%). ^{1}H NMR (400 MHz, Acetone-d_6) δ 7.44 – 7.34 (m, 4H), 7.34 – 7.22 (m, 6H), 7.22 – 7.12 (m, 2H), 7.08 – 6.98 (m, 2H), 6.90 – 6.79 (m, 2H), 3.76 (s, 3H), 2.24 (s, 2H), 1.70 – 1.67 (m, 12H). ^{13}C NMR (101 MHz, Acetone-d_6) δ 169.6, 158.7, 151.8, 151.2, 145.0, 149.3, 149.2, 148.1, 142.7, 129.4, 128.5, 122.2, 122.0, 121.5, 121.4, 121.3, 114.3, 55.4, 43.2, 42.8, 31.1, 31.0, 21.0. HRMS (ESI): *m/z* $[M+Na]^+$ calcd for $C_{33}H_{34}NaO_7S$ 597.1917; found 597.1912.

*{A-A} Dimeric alcohol (**19''**)*

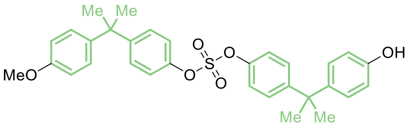

The title compound was prepared according to General Procedure H using **19'** (1.01 g, 1.75 mmol, 1.0 equiv) and acetyl chloride (19 µL, 0.26 mmol, 0.15 equiv) in methanol (2 mL). After stirring for 3 h at rt, the mixture was purified by flash column chromatography (hexanes : ethyl acetate = 6 : 1 to 4 : 1) to afford a colorless viscous oil (470 mg, 50%). ^{1}H NMR (400 MHz, Acetone-d_6) δ 8.17 (s, 1H), 7.45 – 7.33 (m, 4H), 7.33 – 7.22 (m, 4H), 7.22 – 7.12 (m, 2H), 7.12 – 7.02 (m, 2H), 6.91 – 6.79 (m, 2H), 6.79 – 6.71 (m, 2H), 3.76 (s, 3H), 1.67 – 1.65 (m, 12H). ^{13}C NMR (101 MHz, Acetone-d_6) δ 158.7, 156.2, 152.0, 151.8, 149.2, 142.7, 141.5, 129.3, 129.3, 128.5, 128.5, 121.3, 121.2, 115.7, 114.3, 55.4, 42.8, 42.7, 31.2, 31.1. HRMS (ESI): *m/z* $[M-H]^-$ calcd for $C_{31}H_{31}O_6S$ 531.1847; found 531.1843.

*{A-A-C} Trimeric acetate (**21'**)*

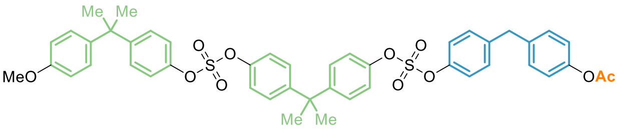

The title compound was prepared according to General Procedure E using **20** (687 mg, 1.12 mmol, 1.0 equiv), **5'** (399 mg, 1.12 mmol, 1.0 equiv), and DBU (42 µL, 0.28 mmol, 0.25 equiv) in anhydrous MeCN (4 mL). After stirring for 2 h at 80 ˚C, the mixture was purified by flash column chromatography (hexanes : ethyl acetate = dichloromethane = 6 : 1 : 1) to afford a colorless viscous oil (886 mg, 95%). ^{1}H NMR (400 MHz, Acetone-d_6) δ 7.44 – 7.24 (m, 18H), 7.19 – 7.13 (m, 2H), 7.07 – 7.00 (m, 2H), 6.87 – 6.80 (m, 2H), 4.04 (s, 2H), 3.75 (s, 3H), 2.23 (s, 3H), 1.72 – 1.66 (m, 12H). ^{13}C NMR (101 MHz, Acetone-d_6) δ 169.7, 158.7, 151.8, 150.7, 150.4, 149.9,

149.8, 149.4, 149.4, 149.2, 142.7, 142.3, 139.1, 131.5, 131.4, 130.5, 129.5, 129.4, 128.5, 122.7, 122.1, 122.0, 121.6, 121.3, 114.3, 55.4, 43.4, 42.8, 40.9, 31.1, 30.9, 21.0. HRMS (ESI): *m/z* $[M+Na]^+$ calcd for $C_{46}H_{44}NaO_{11}S_2$ 859.2217; found 859.2233.

{A-A-C} *Trimeric alcohol (**21''**)*

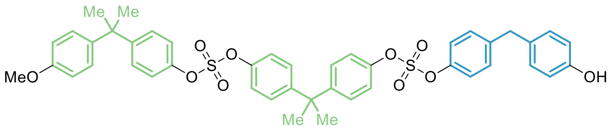

The title compound was prepared according to General Procedure I using **21'** (899 mg, 1.07 mmol, 1.0 equiv) and K_2CO_3 (370 mg, 2.68 mmol, 2.5 equiv) in methanol (11 mL). After stirring for 4 h at rt, the mixture was purified by flash column chromatography (hexanes : ethyl acetate = 6 : 1 : 1 to 4 : 1 : 1) to afford a colorless viscous oil (438 mg, 55%). ^{1}H NMR (400 MHz, Acetone-d_6) δ 8.17 (s, 1H), 7.44 – 7.24 (m, 16H), 7.20 – 7.11 (m, 2H), 7.10 – 7.02 (m, 2H), 6.88 – 6.81 (m, 2H), 6.81 – 6.73 (m, 2H), 3.92 (s, 2H), 3.75 (s, 3H), 1.72 (s, 6H), 1.66 (s, 6H). ^{13}C NMR (101 MHz, Acetone-d_6) δ 206.1, 158.7, 156.8, 151.9, 150.7, 150.7, 149.6, 149.4, 149.4, 149.2, 143.2, 142.7, 132.3, 131.2, 130.7, 129.4, 129.4, 128.5, 121.8, 121.5, 121.3, 116.2, 114.3, 55.4, 43.4, 42.8, 40.8, 31.1, 30.9. HRMS (ESI): *m/z* $[M-H]^-$ calcd for $C_{44}H_{41}O_{10}S_2$ 793.2147; found 793.2227.

MeO-{A-A-A}-OMe *(**Side P**)*

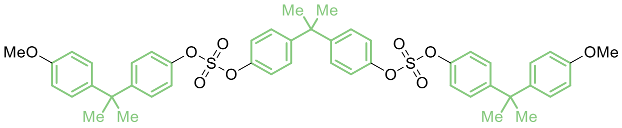

A white solid. mp: 130 – 133 ˚C. ^{1}H NMR (400 MHz, DMSO-d_6) δ 7.43 – 7.24 (m, 16H), 7.17 – 7.06 (m, 4H), 6.91 – 6.76 (m, 4H), 3.70 (s, 6H), 1.66 – 1.61 (m, 18H). ^{13}C NMR (101 MHz, DMSO-d_6) δ 157.2, 150.6, 149.6, 147.9, 147.6, 141.5, 128.6, 128.4, 127.5, 120.7, 120.4, 113.4, 54.9, 42.4, 41.7, 30.4, 30.2. HRMS (ESI): *m/z* $[M+Na]^+$ calcd for $C_{47}H_{48}NaO_{10}S_2$ 859.2581; found 859.2571.

10. NMR Spectra

^{1}H NMR (400 MHz, $CDCl_3$) of **S-1**

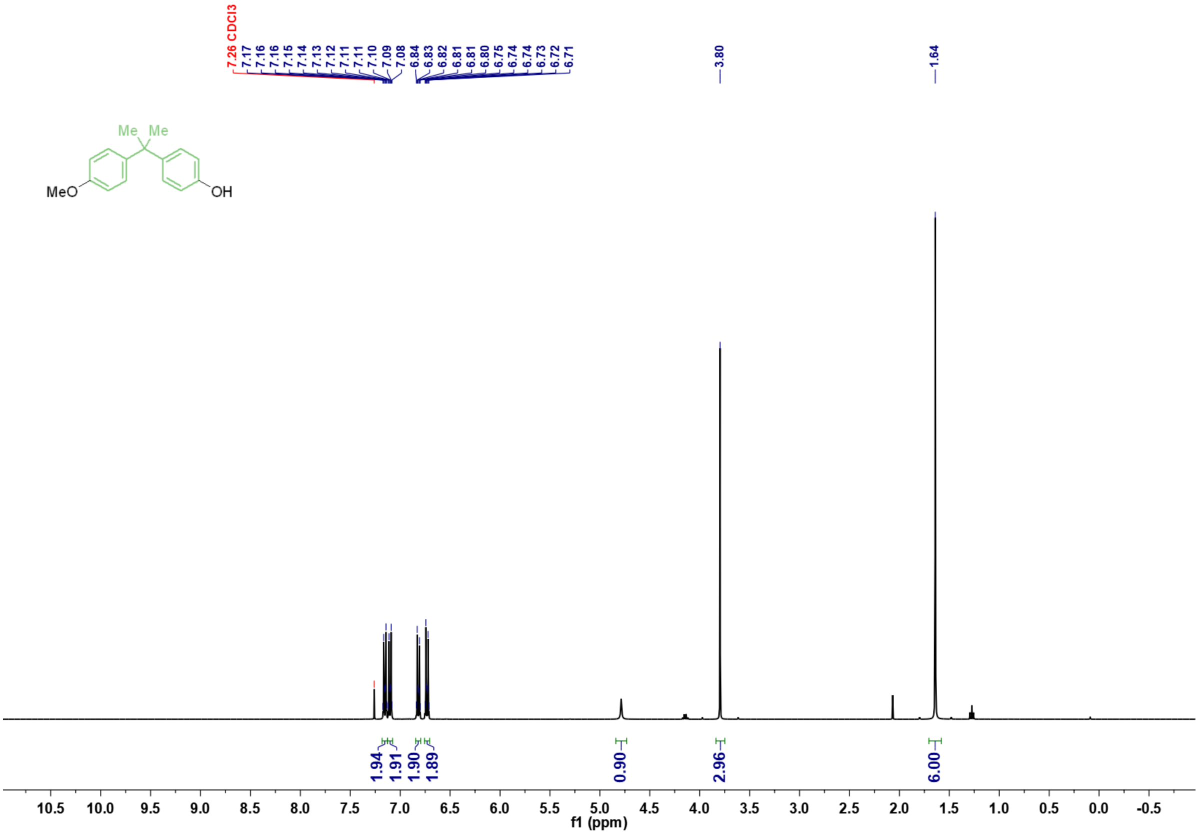

^{1}H NMR (400 MHz, DMSO-d_6) of **S-2**

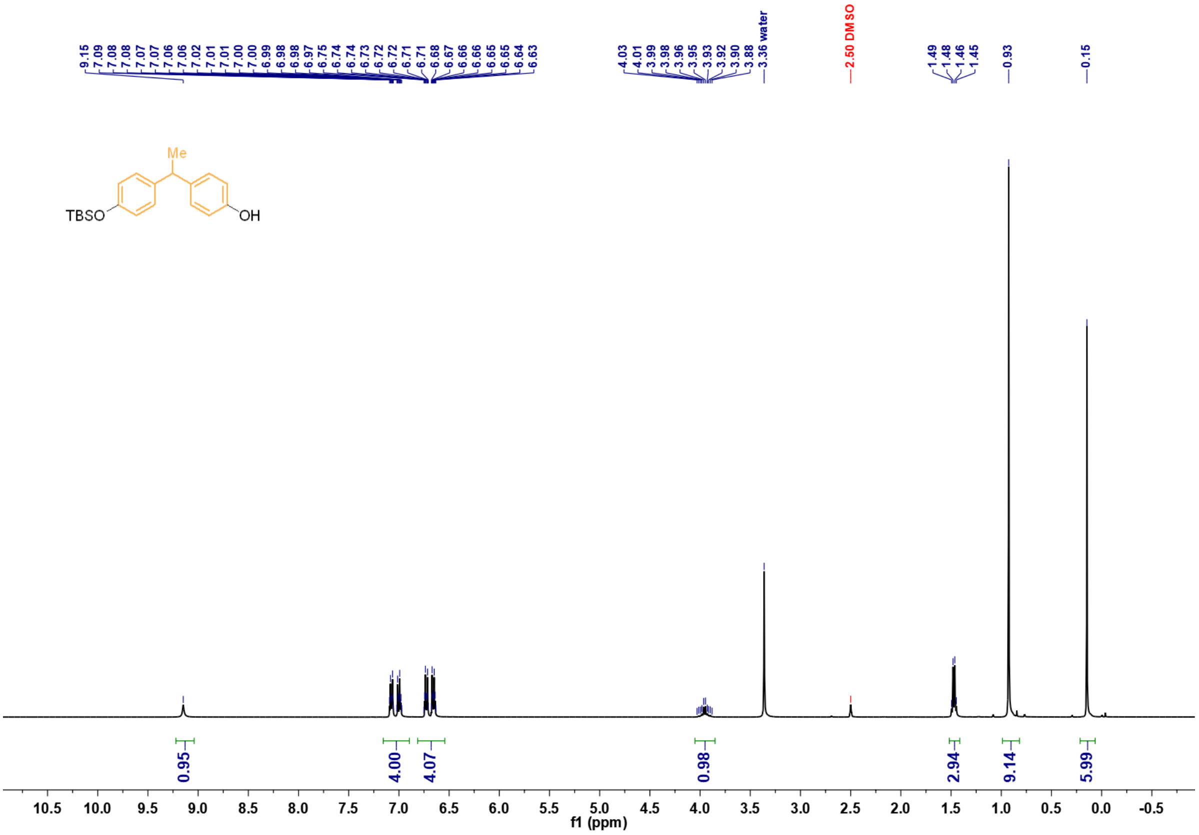

^{1}H NMR (400 MHz, $CDCl_3$) of **S-3**

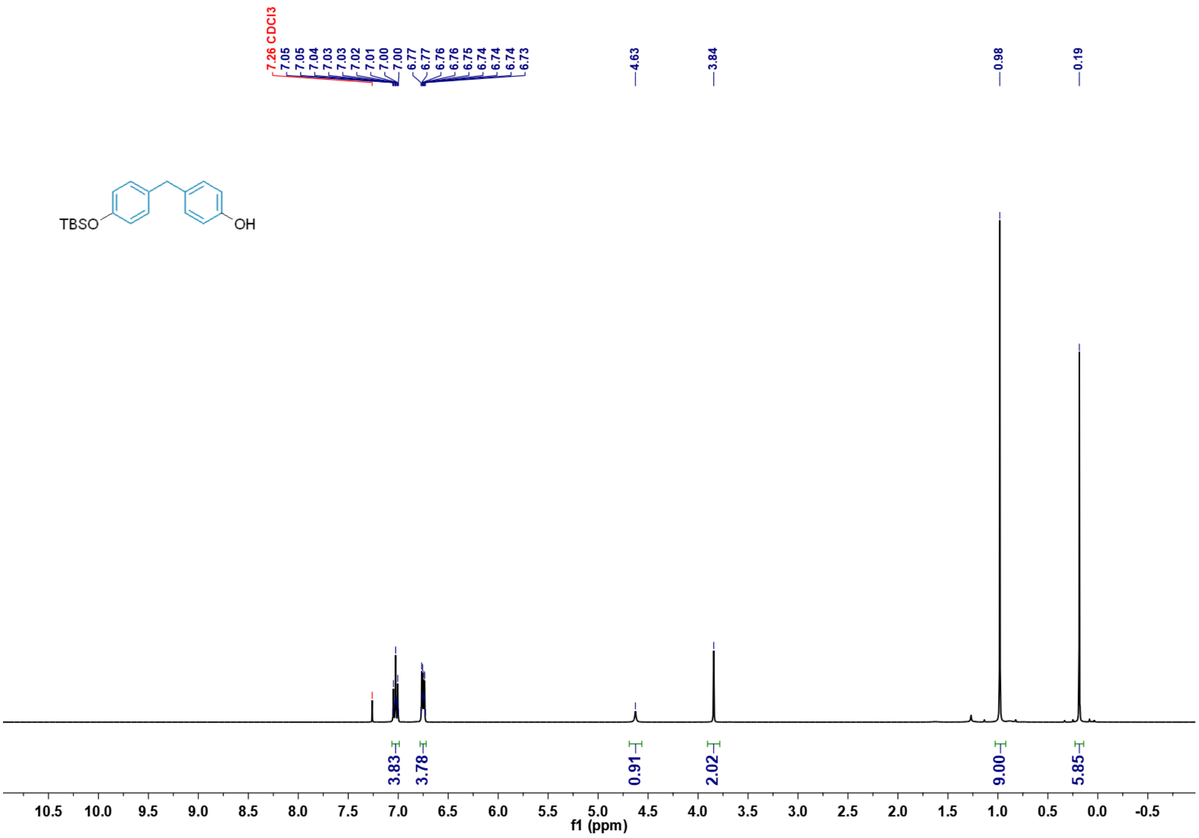

^{1}H NMR (400 MHz, $CDCl_3$) of **S-4**

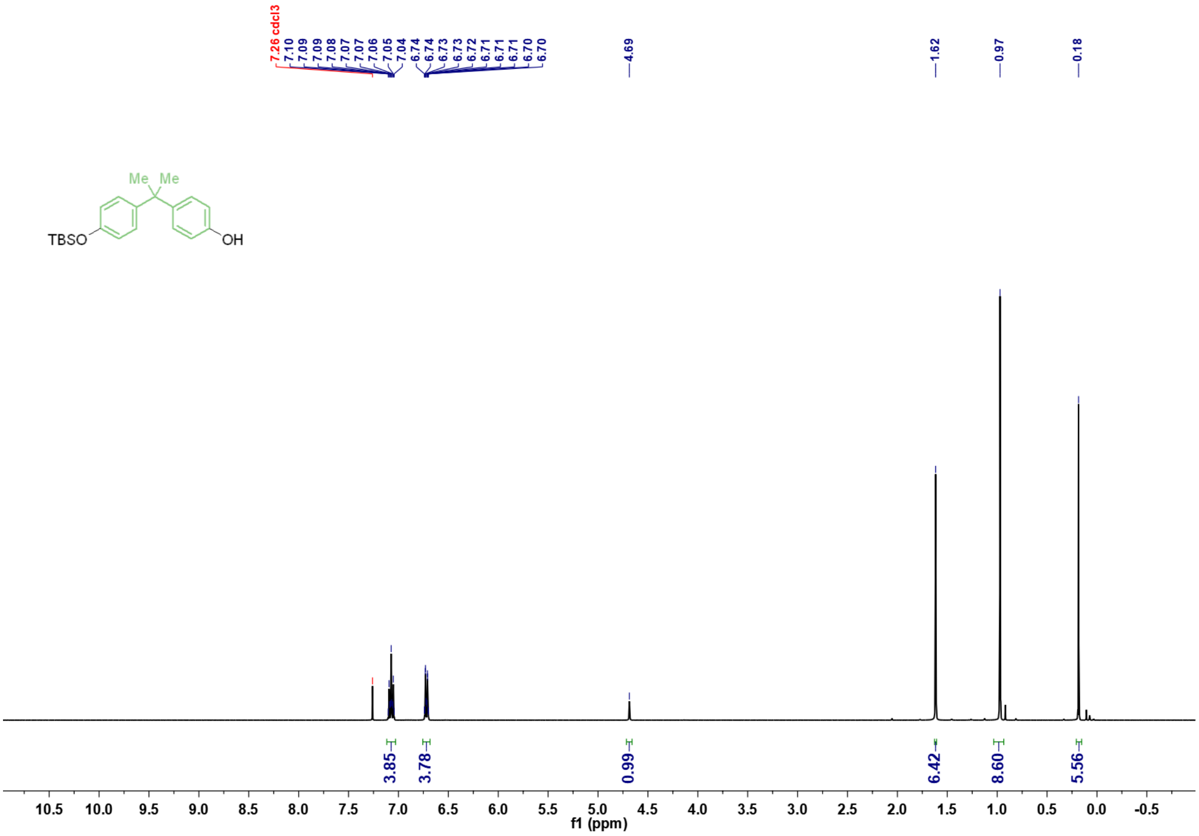

^{1}H NMR (400 MHz, CD_3CN) of **S-5**

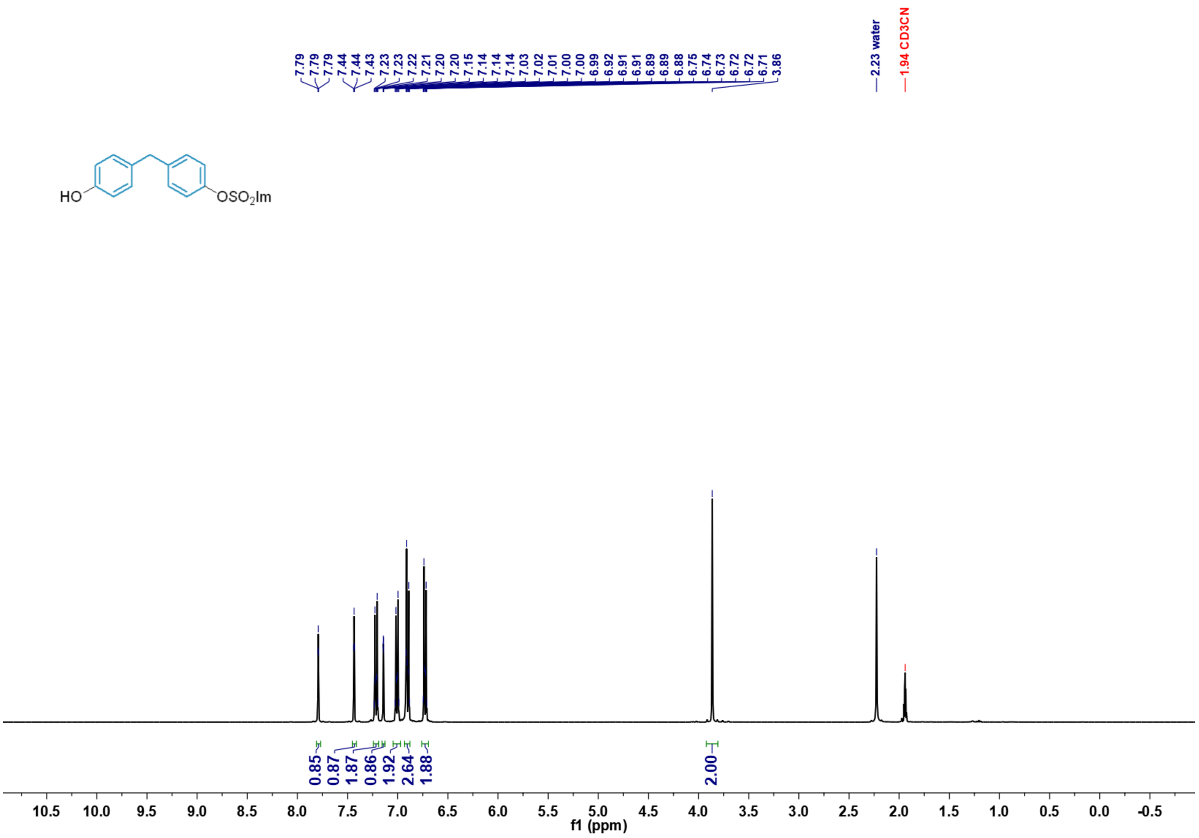

^{13}C NMR (101 MHz, CD_3CN) of **S-5**

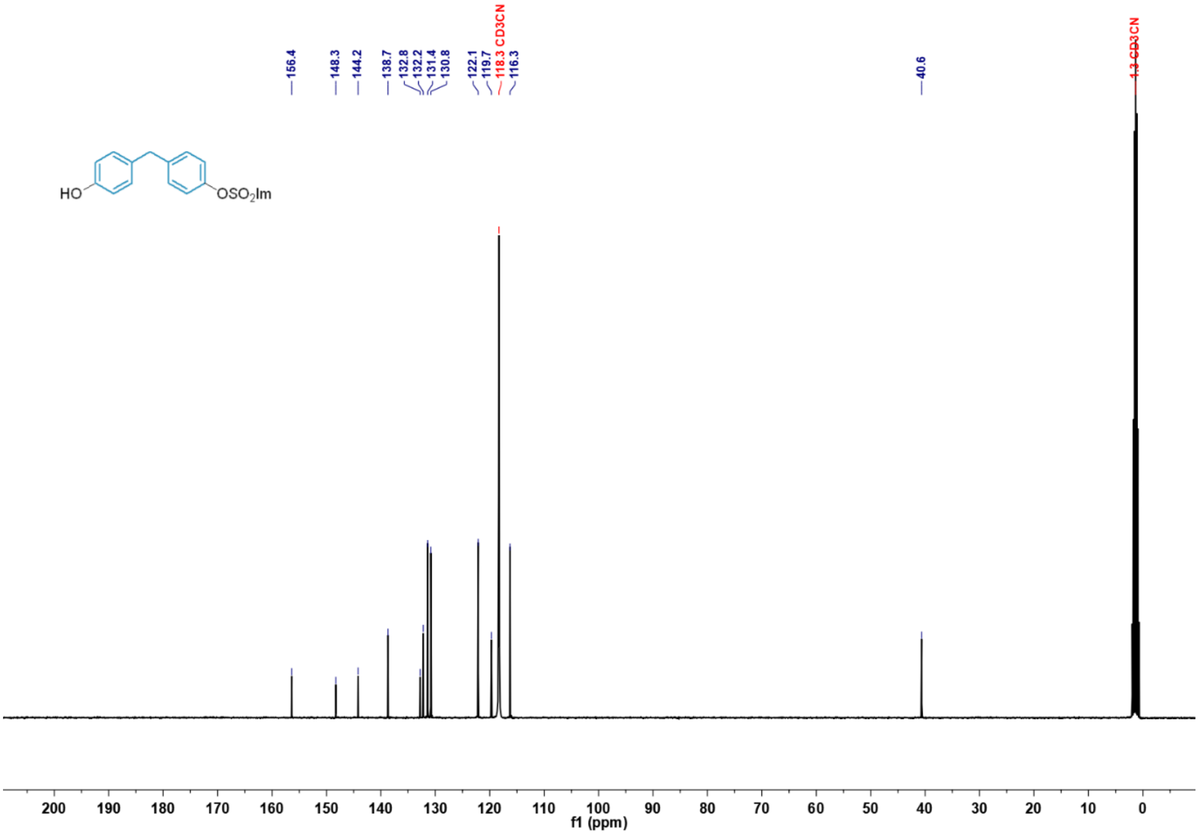

^{1}H NMR (400 MHz, DMSO-d_6) of **S-6**

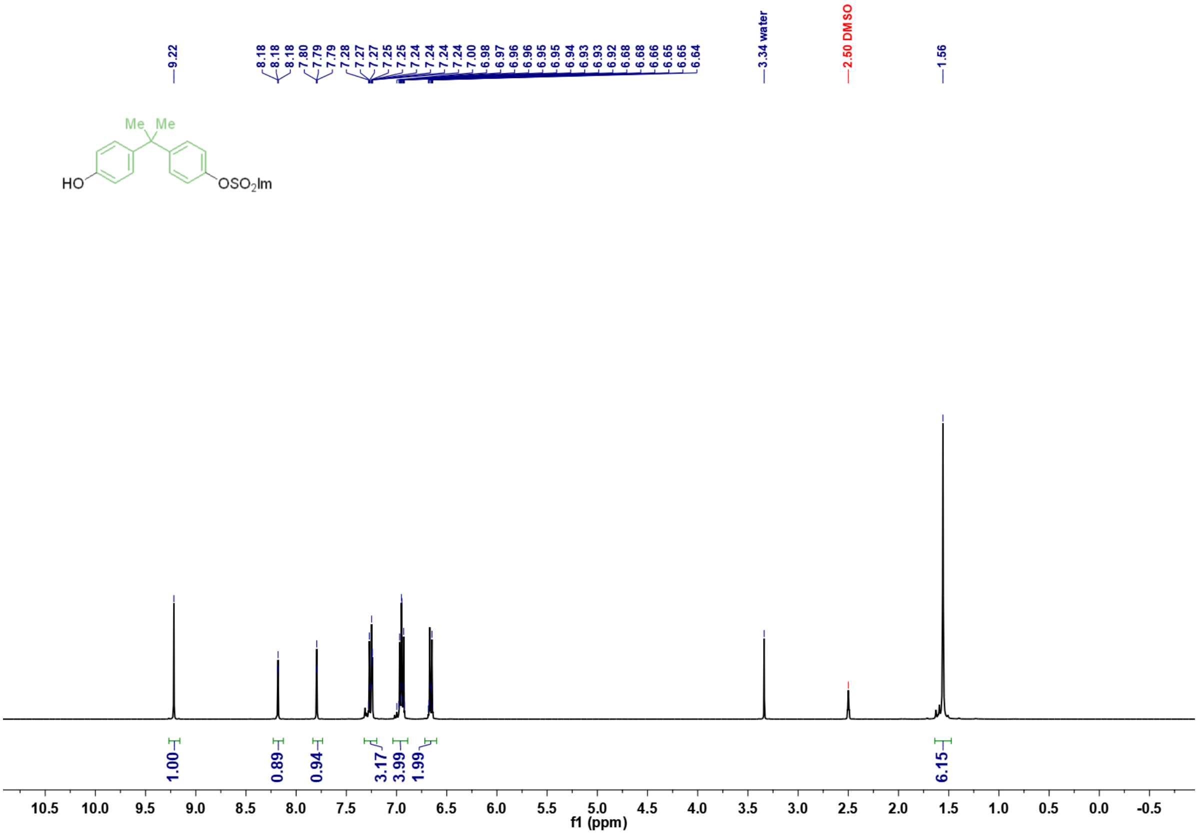

^{13}C NMR (101 MHz, DMSO-d_6) of **S-6**

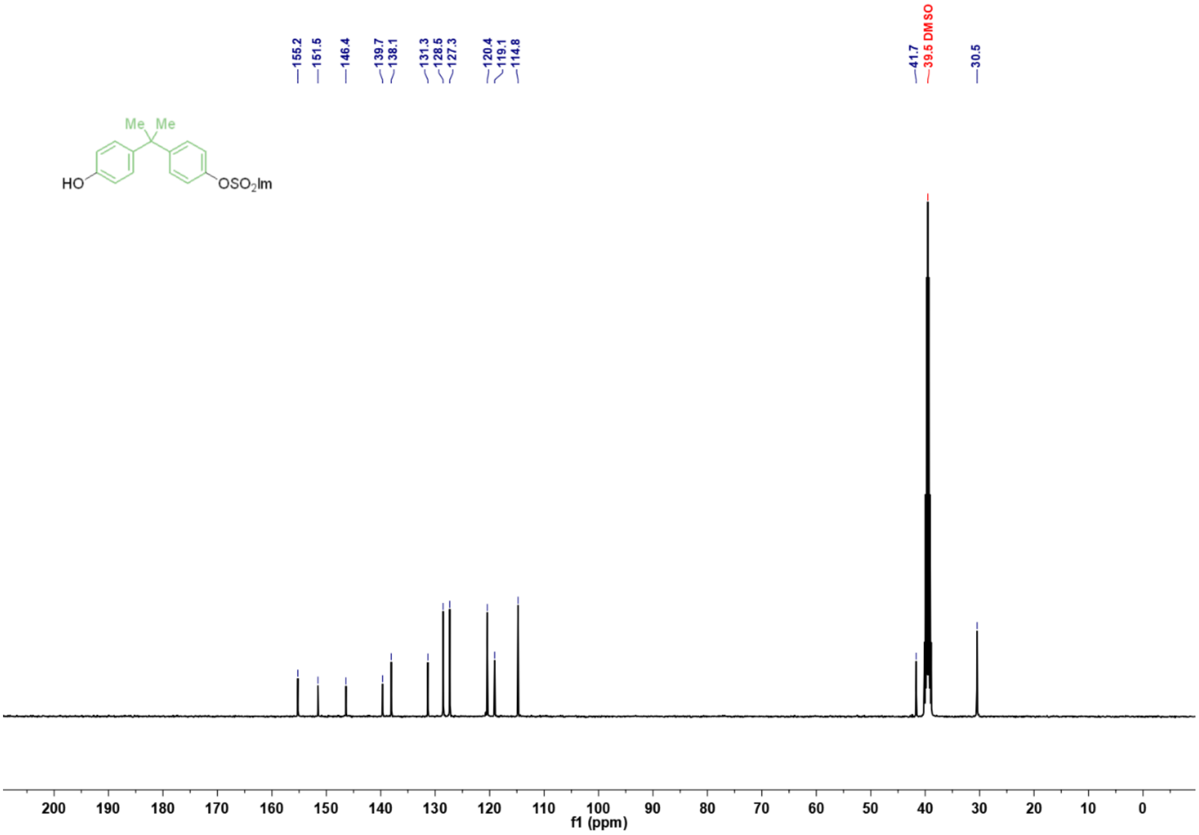

^{1}H NMR (400 MHz, CD_3CN) of **S-7**

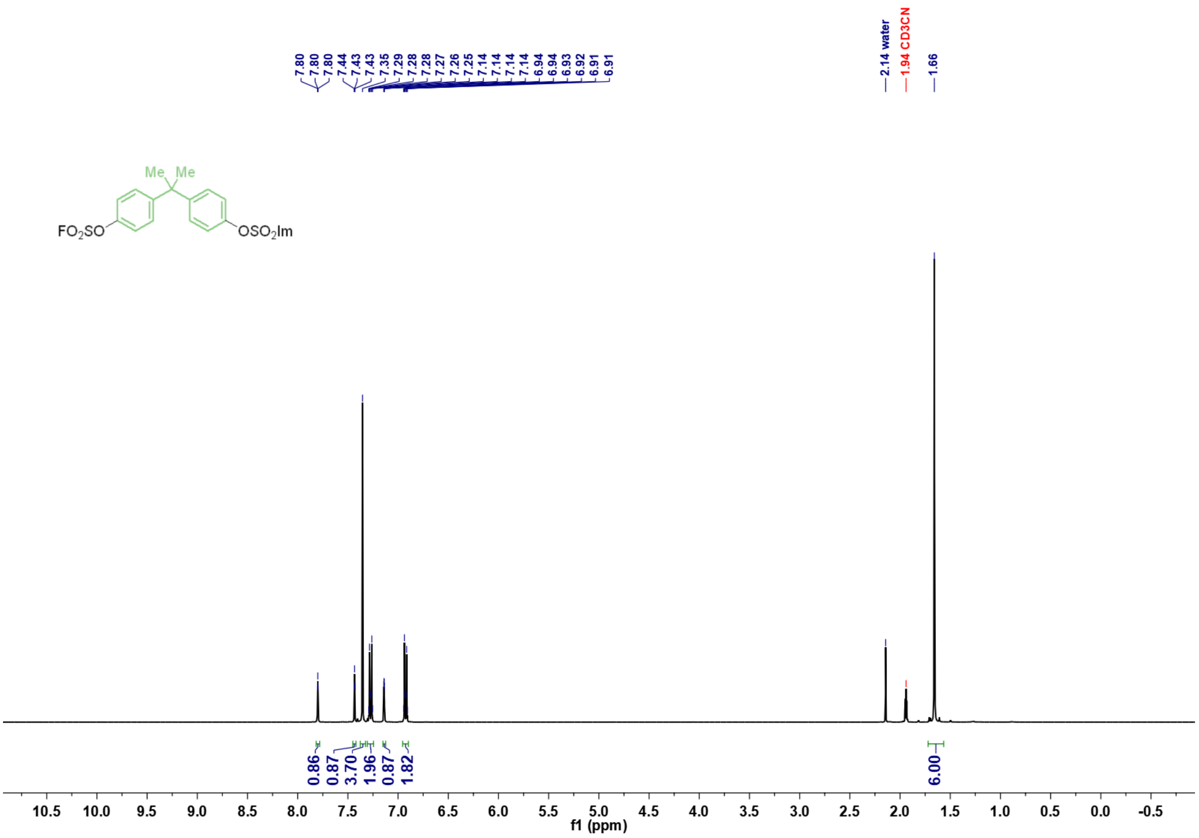

^{13}C NMR (101 MHz, CD_3CN) of **S-7**

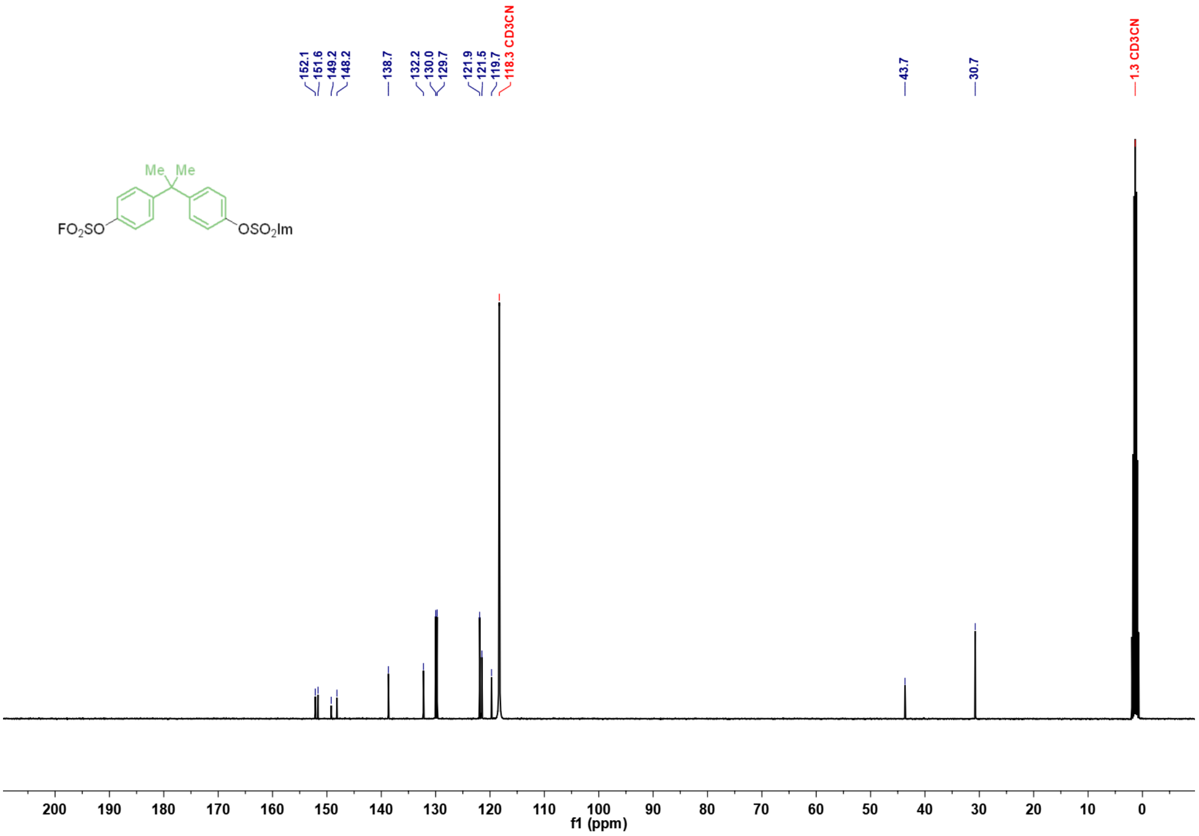

^{19}F NMR (376 MHz, CD_3CN) of **S-7**

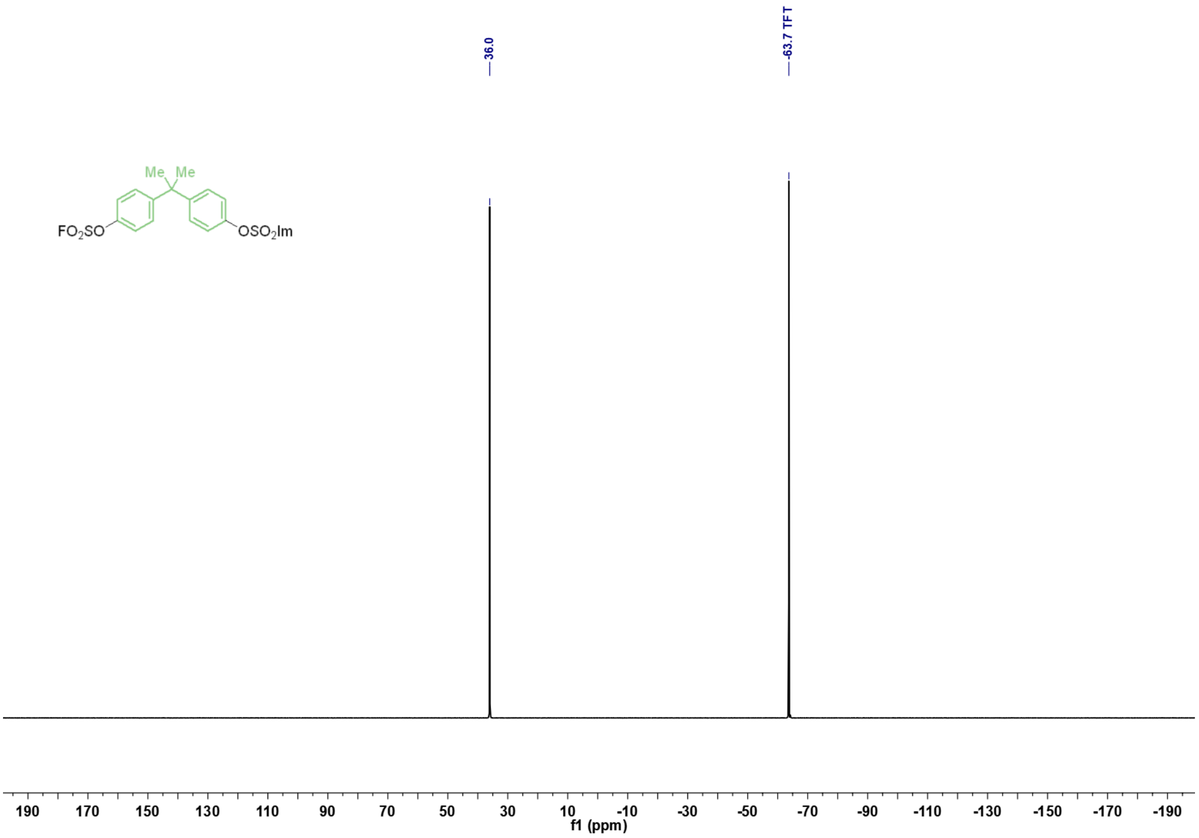

^{1}H NMR (400 MHz, $CDCl_3$) of **S-8**

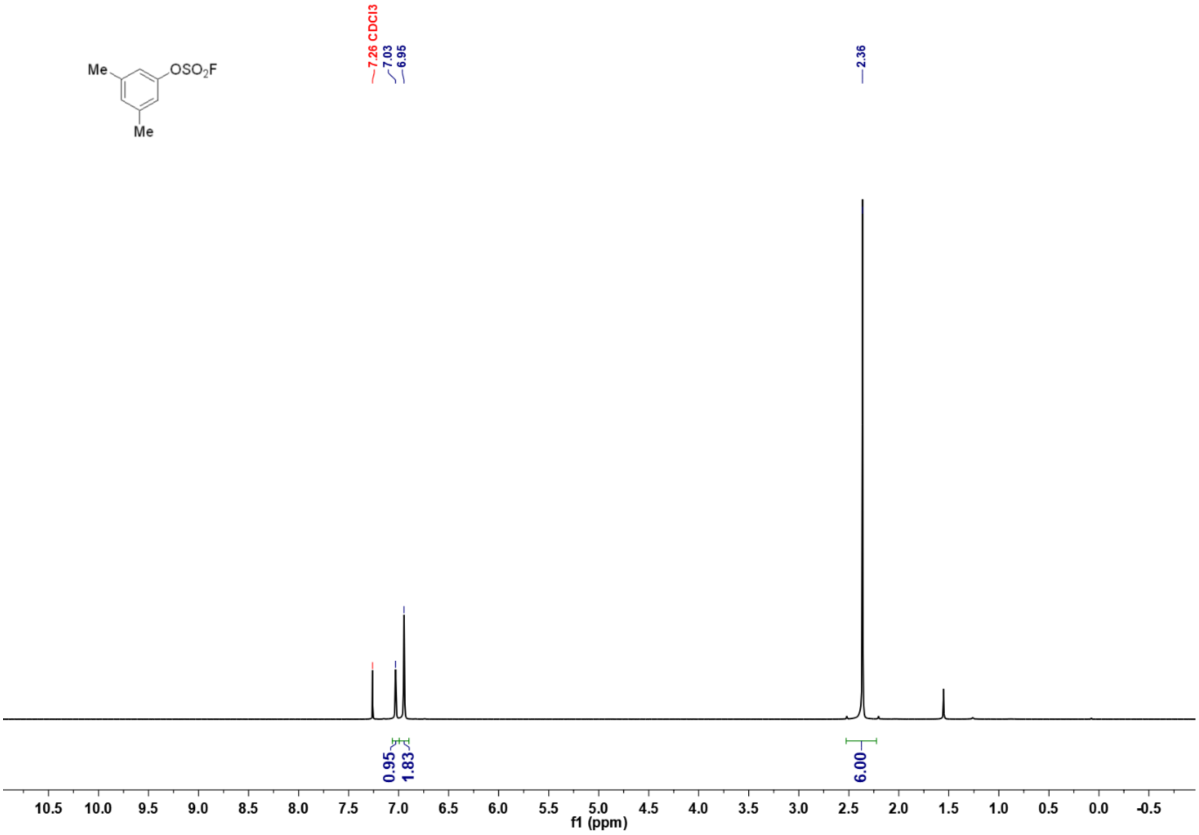

^{19}F NMR (376 MHz, $CDCl_3$) of **S-8**

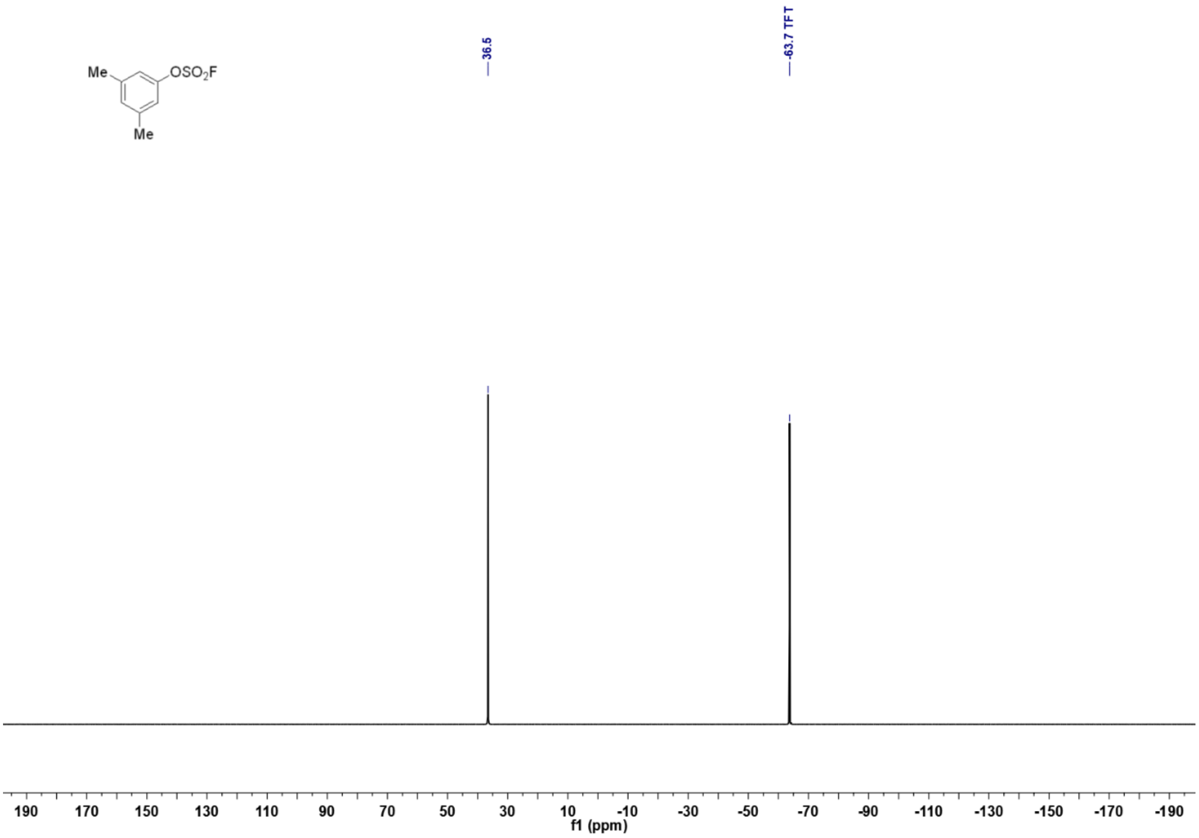

^{1}H NMR (400 MHz, CD_3CN) of **S-9**

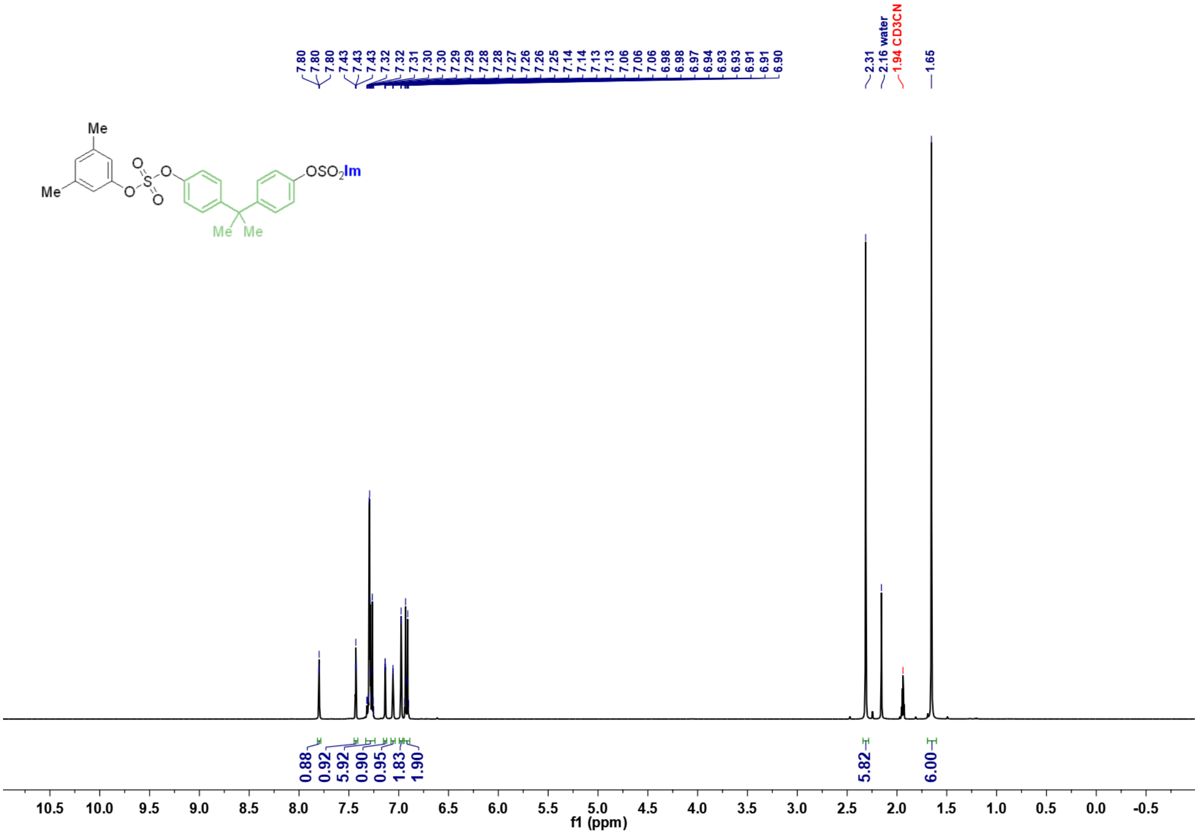

^{13}C NMR (101 MHz, CD_3CN) of **S-9**

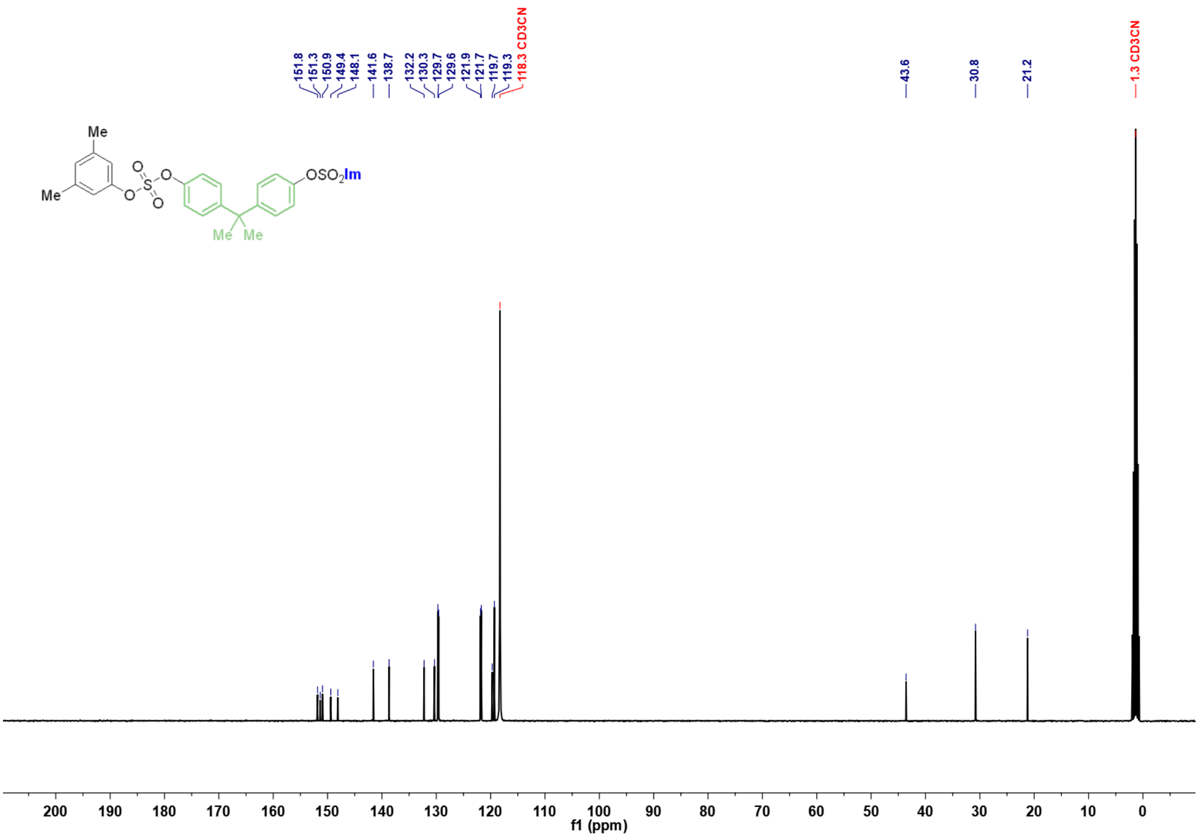

^{1}H NMR (400 MHz, CD_3CN) of **S-10**

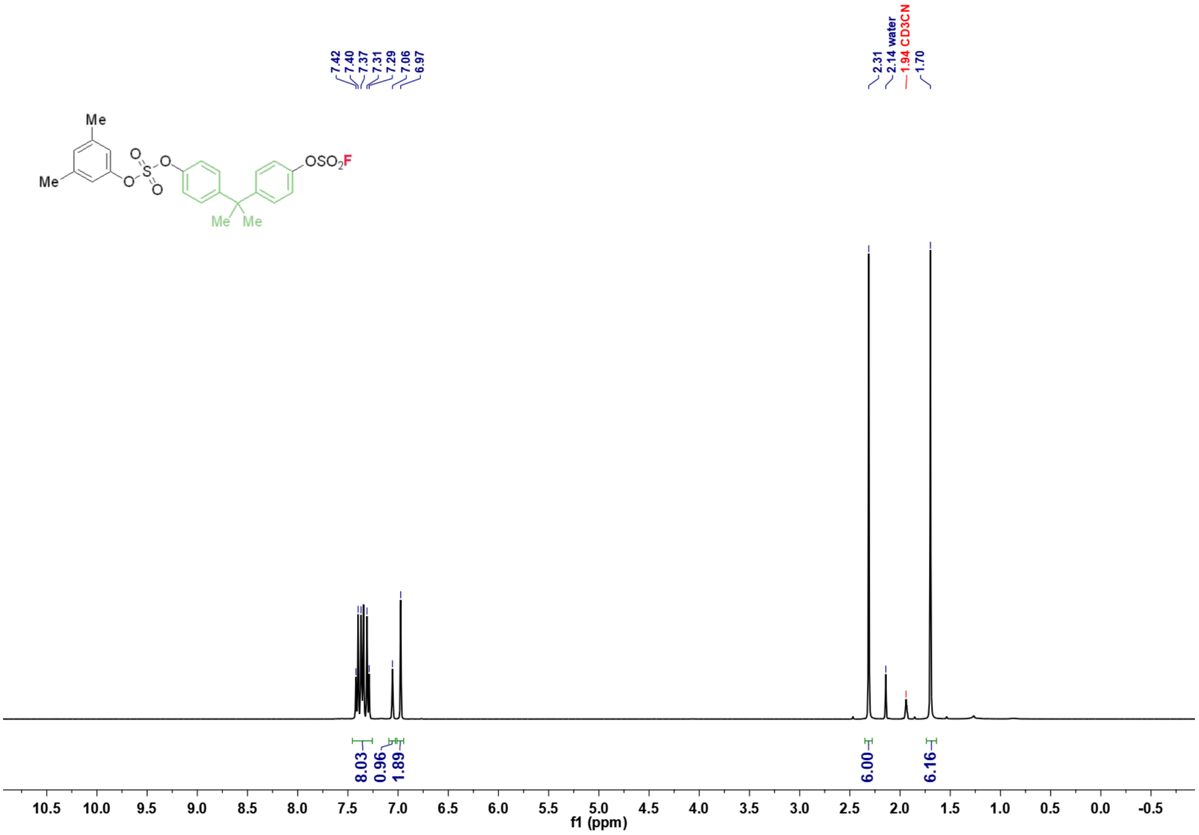

^{13}C NMR (101 MHz, CD_3CN) of **S-10**

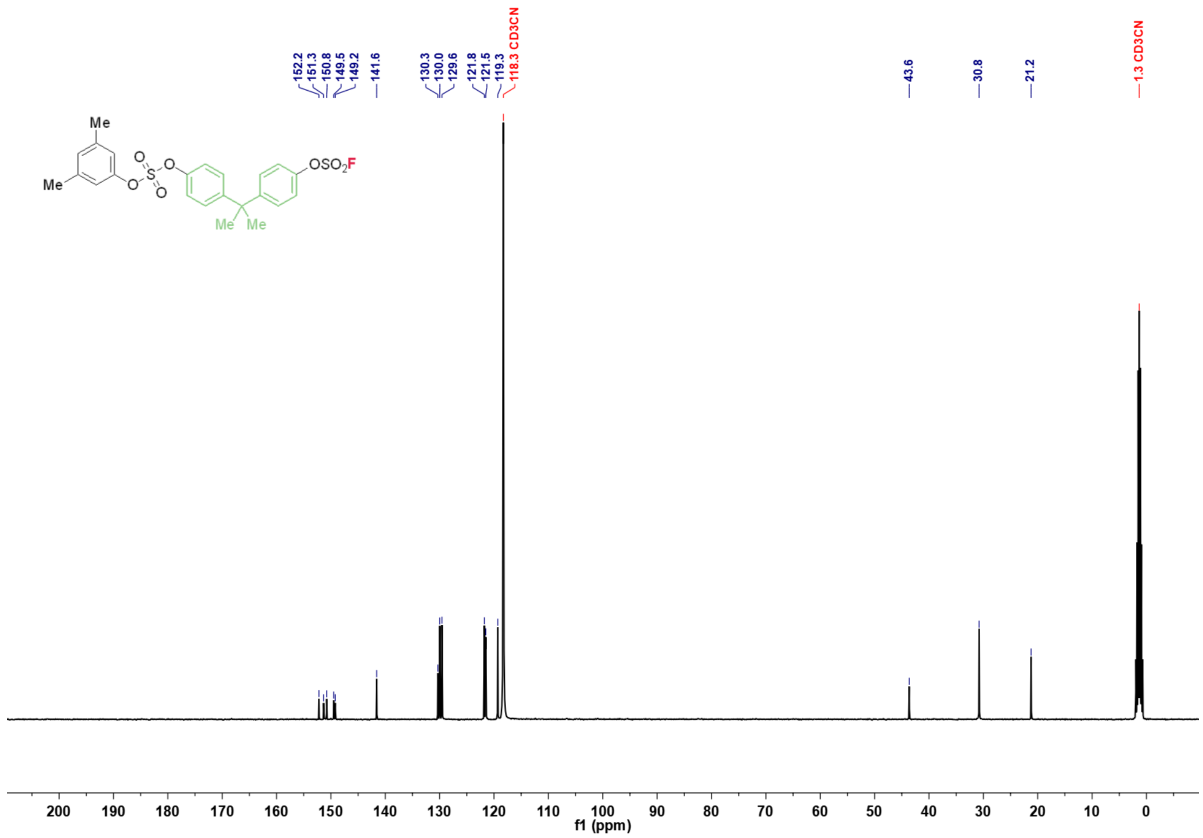

^{19}F NMR (376 MHz, CD_3CN) of **S-10**

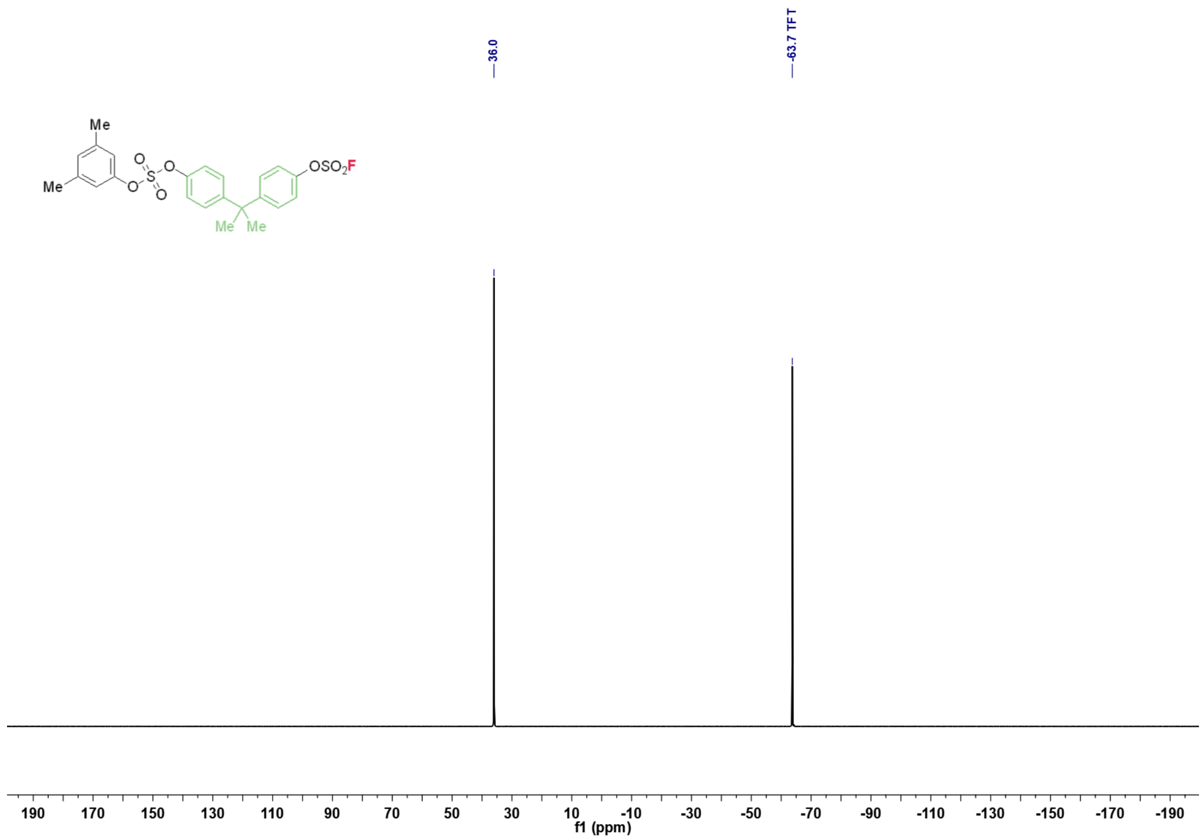

^{1}H NMR (400 MHz, $CDCl_3$) of **1**

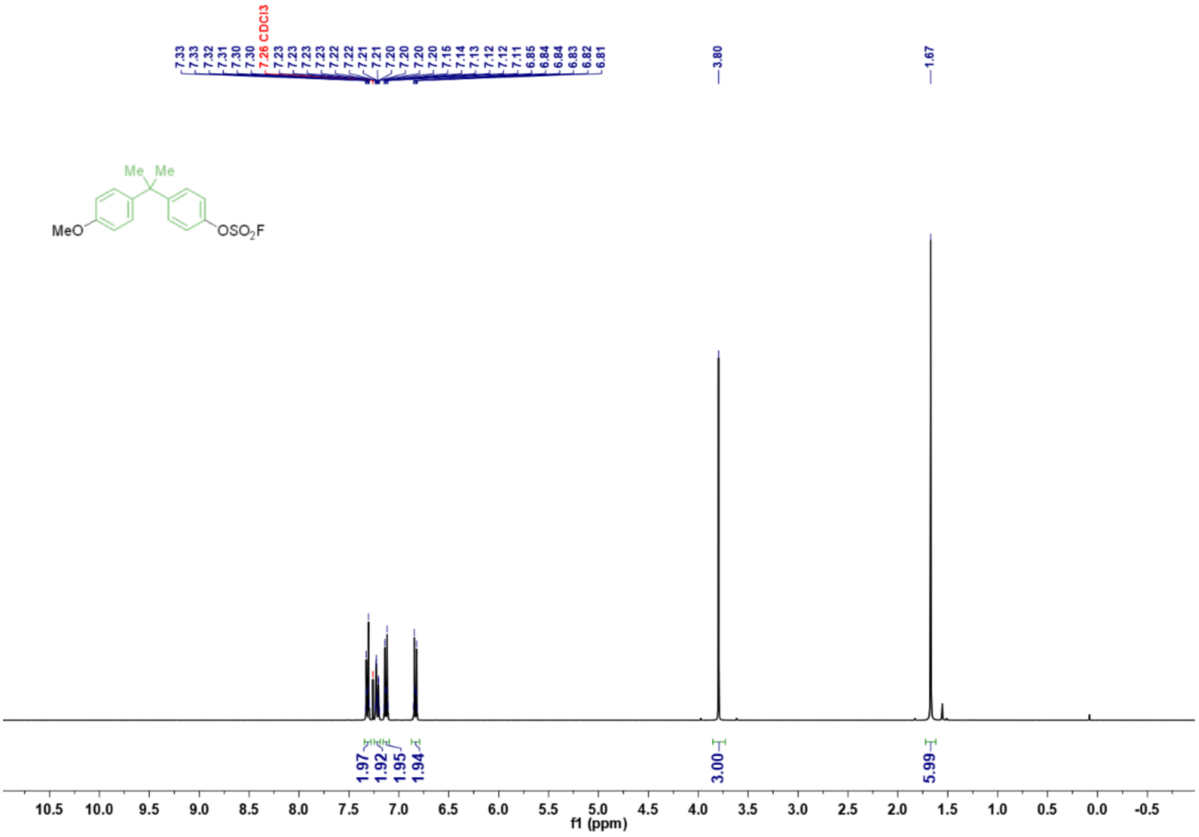

^{13}C NMR (101 MHz, $CDCl_3$) of **1**

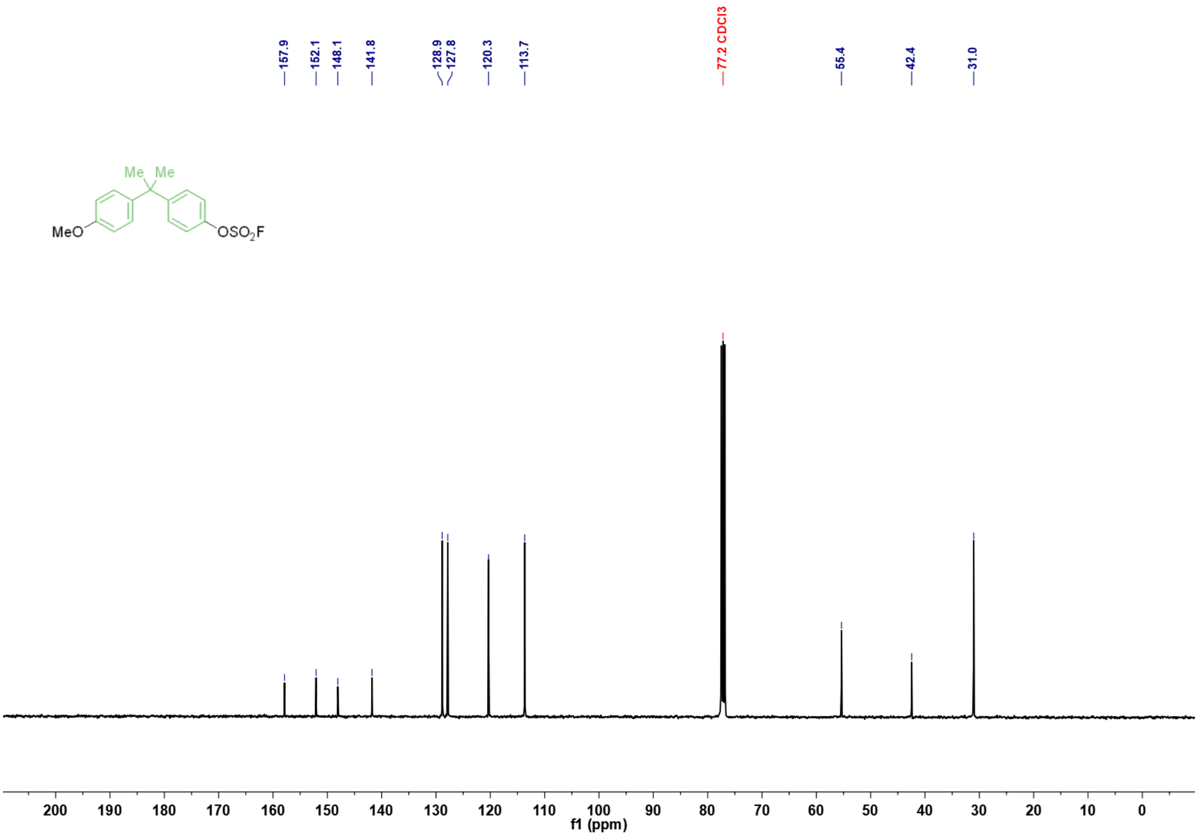

^{19}F NMR (376 MHz, $CDCl_3$) of **1**

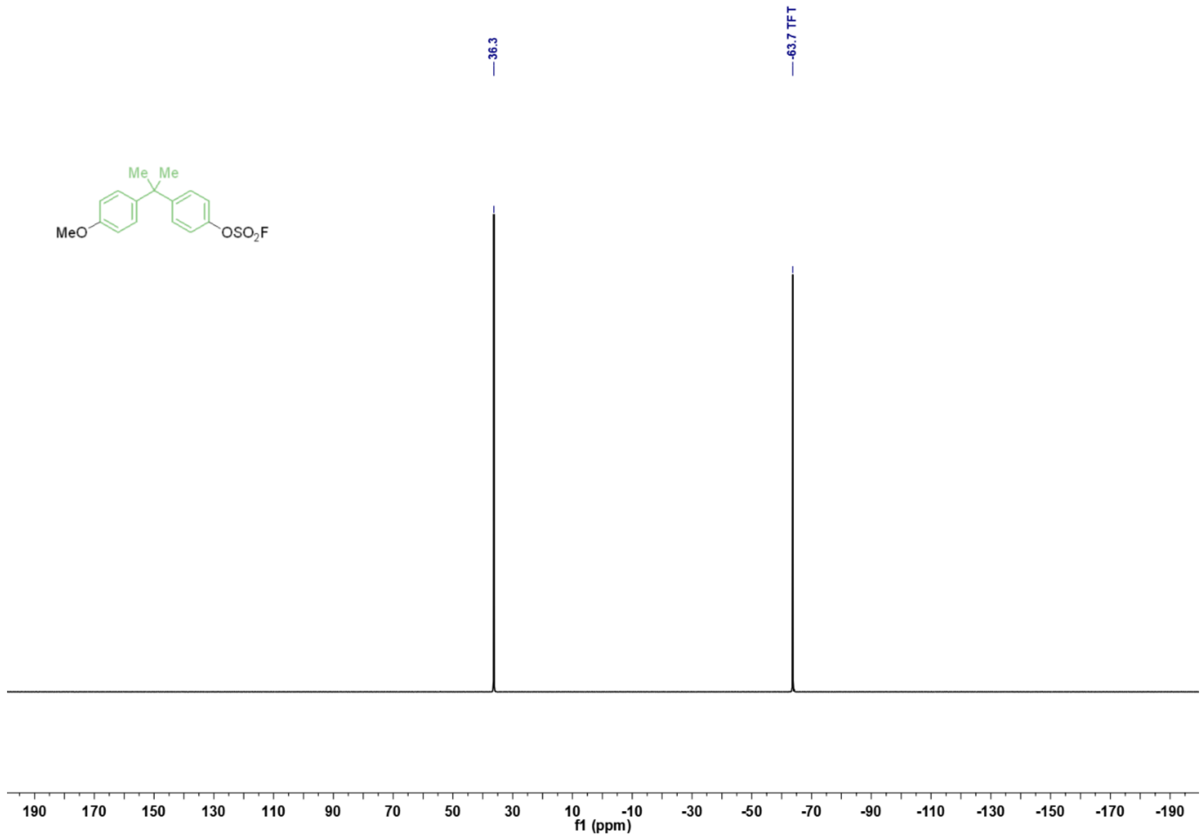

^{1}H NMR (400 MHz, $CDCl_3$) of **2**

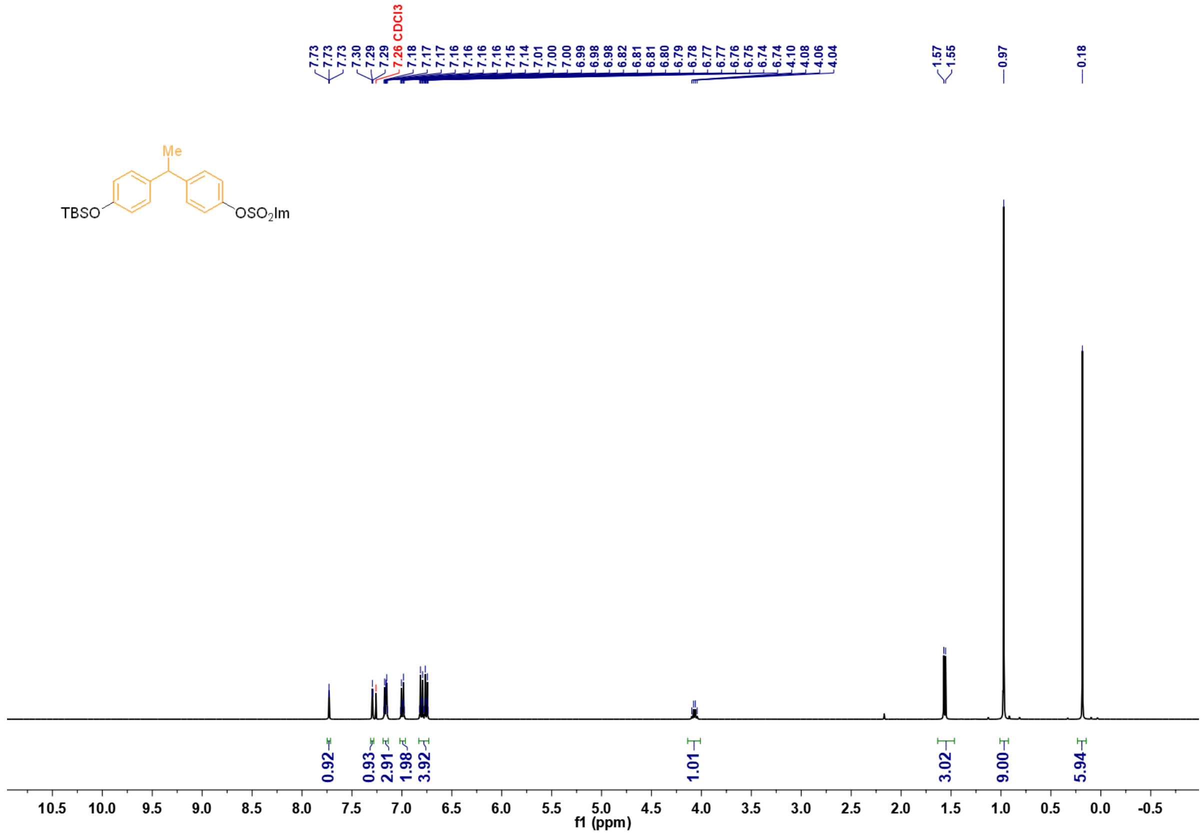

^{13}C NMR (101 MHz, $CDCl_3$) of **2**

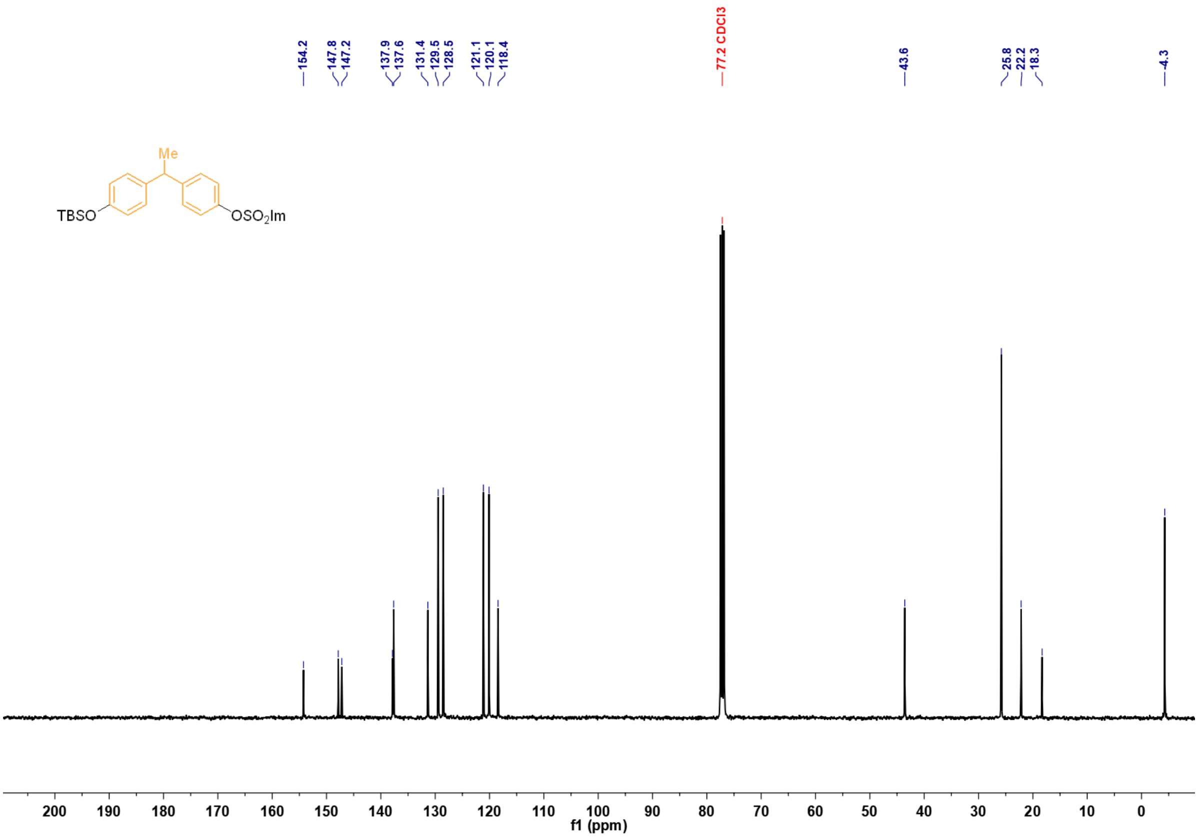

^{1}H NMR (400 MHz, DMSO-d_6) of **3**

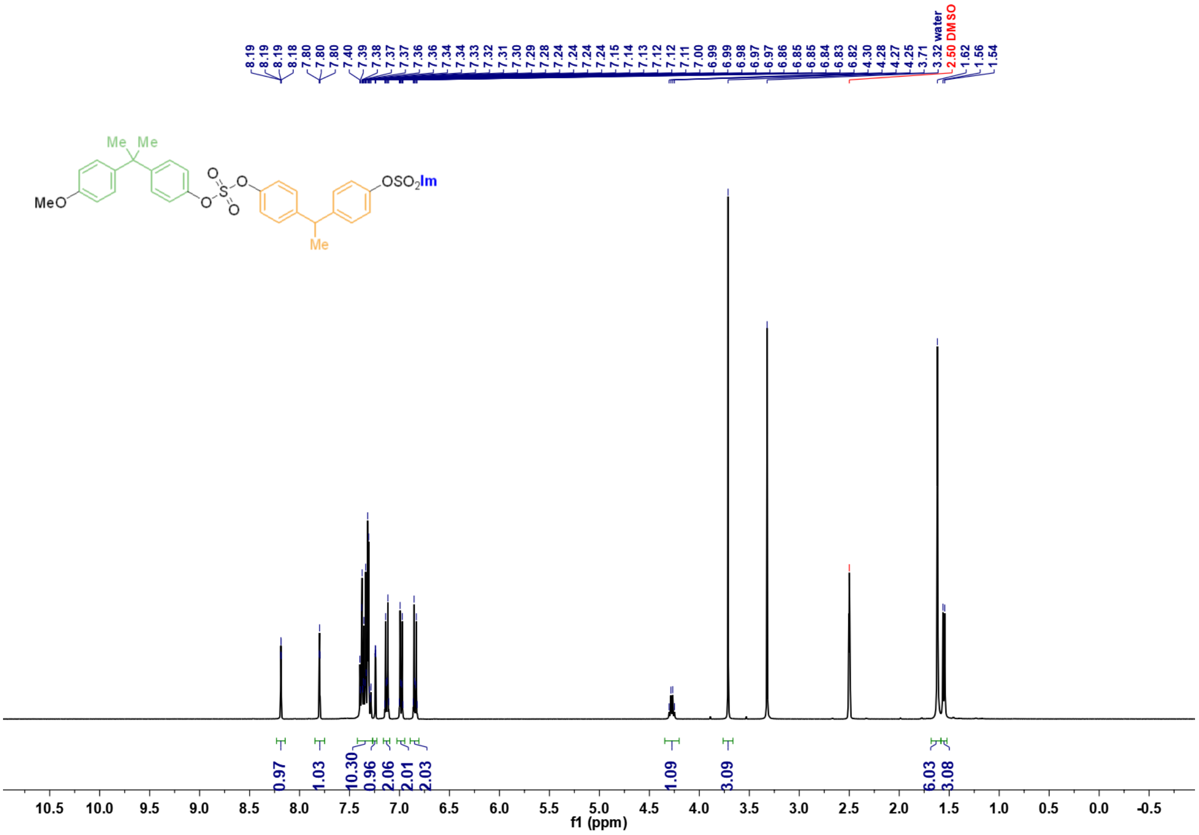

^{13}C NMR (101 MHz, DMSO-d_6) of **3**

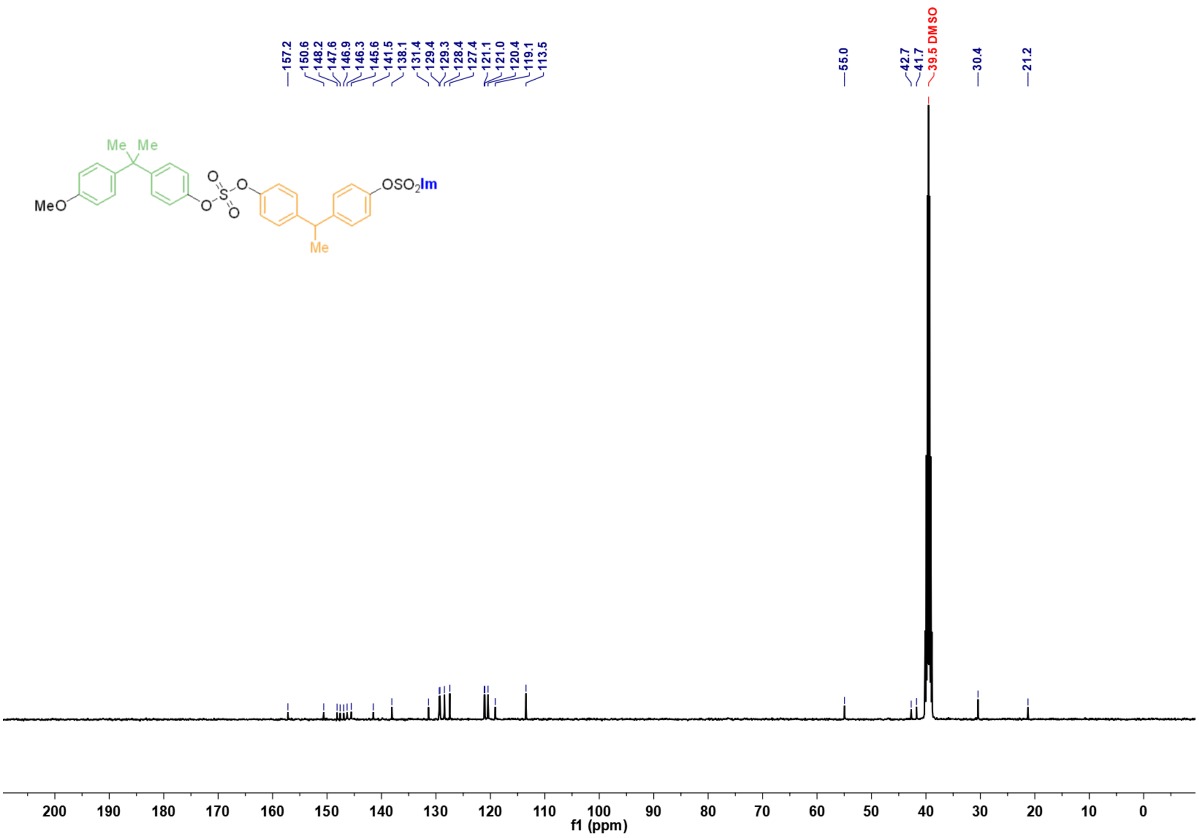

^{1}H NMR (400 MHz, DMSO-d_6) of **4**

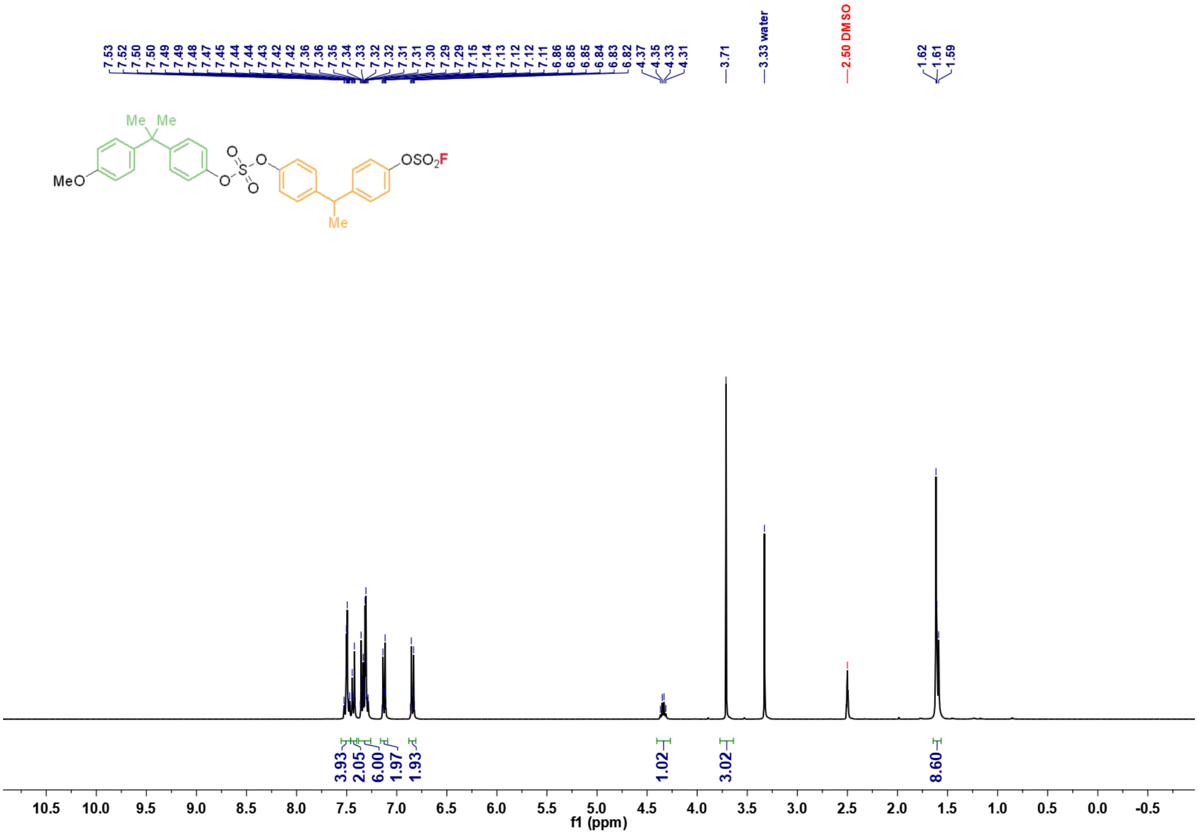

^{13}C NMR (101 MHz, DMSO-d_6) of **4**

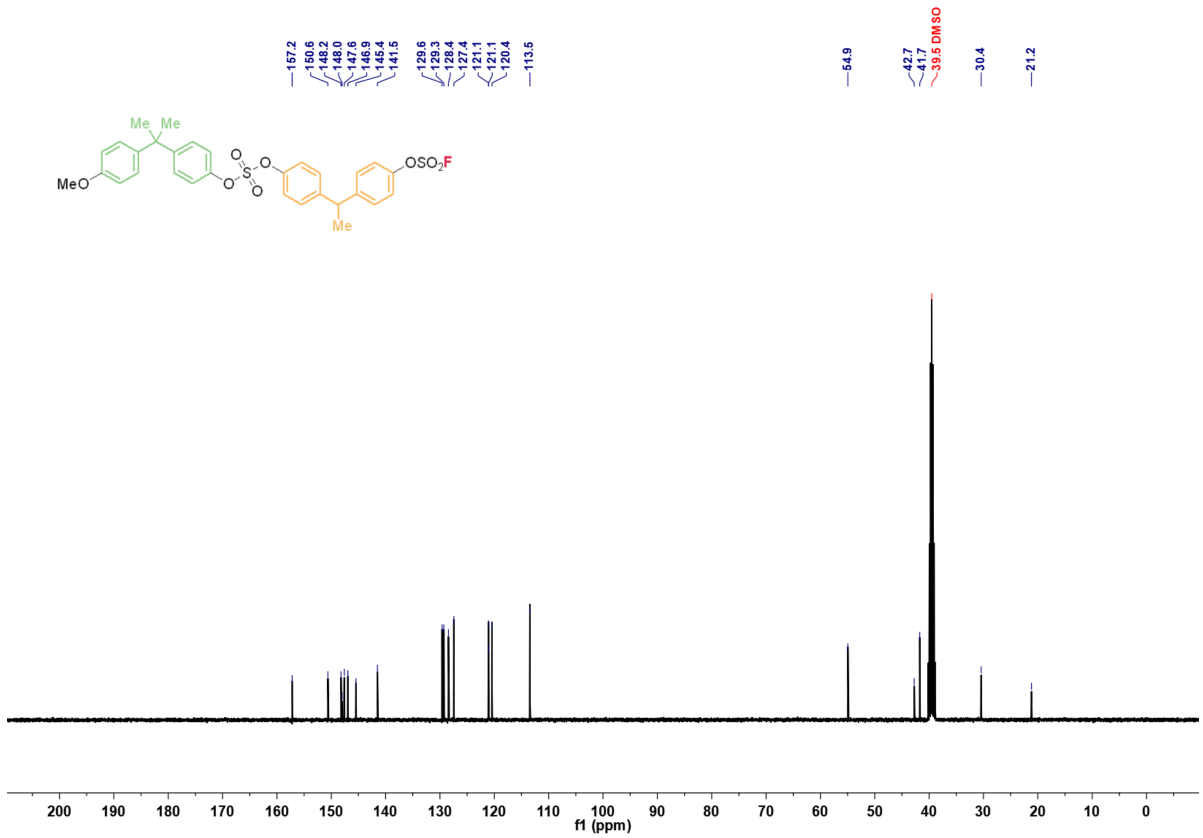

^{19}F NMR (376 MHz, DMSO-d_6) of **4**

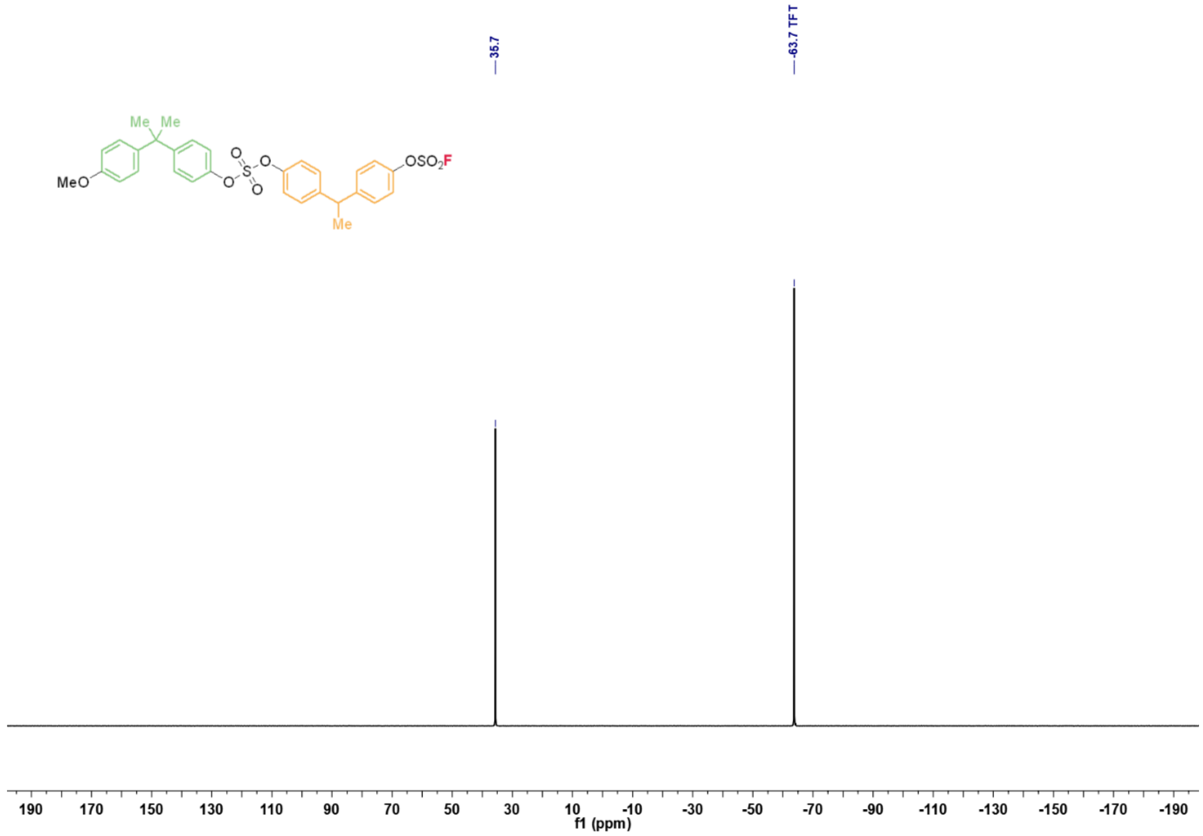

^1H NMR (400 MHz, $CDCl_3$) of **5**

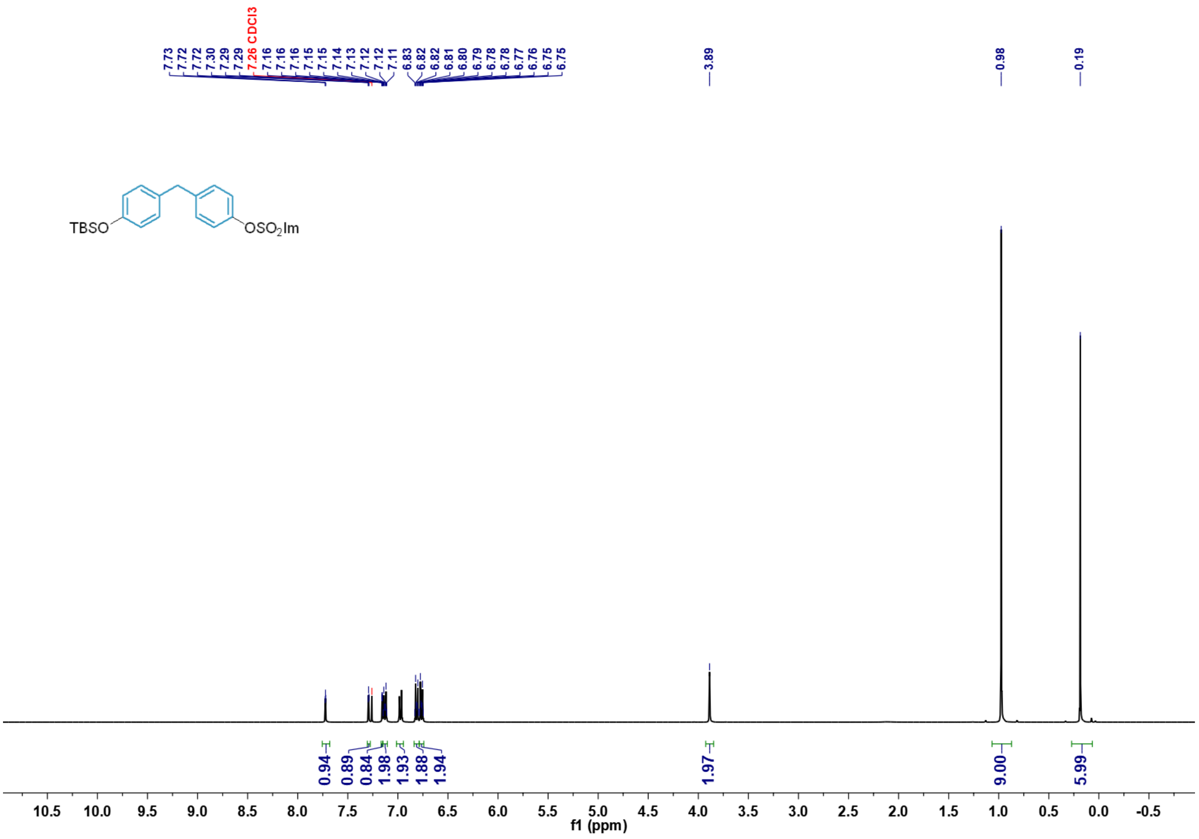

^{13}C NMR (101 MHz, $CDCl_3$) of **5**

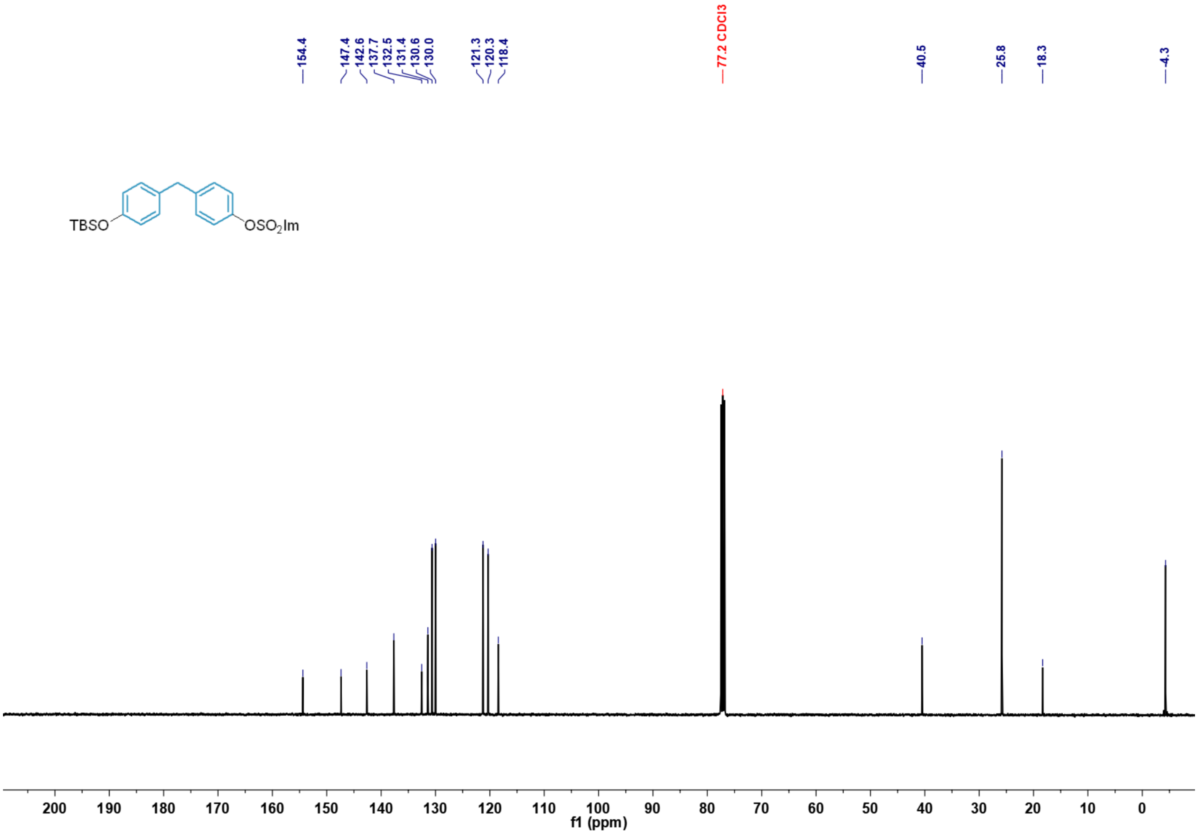

^{1}H NMR (400 MHz, CD_3CN) of **6**

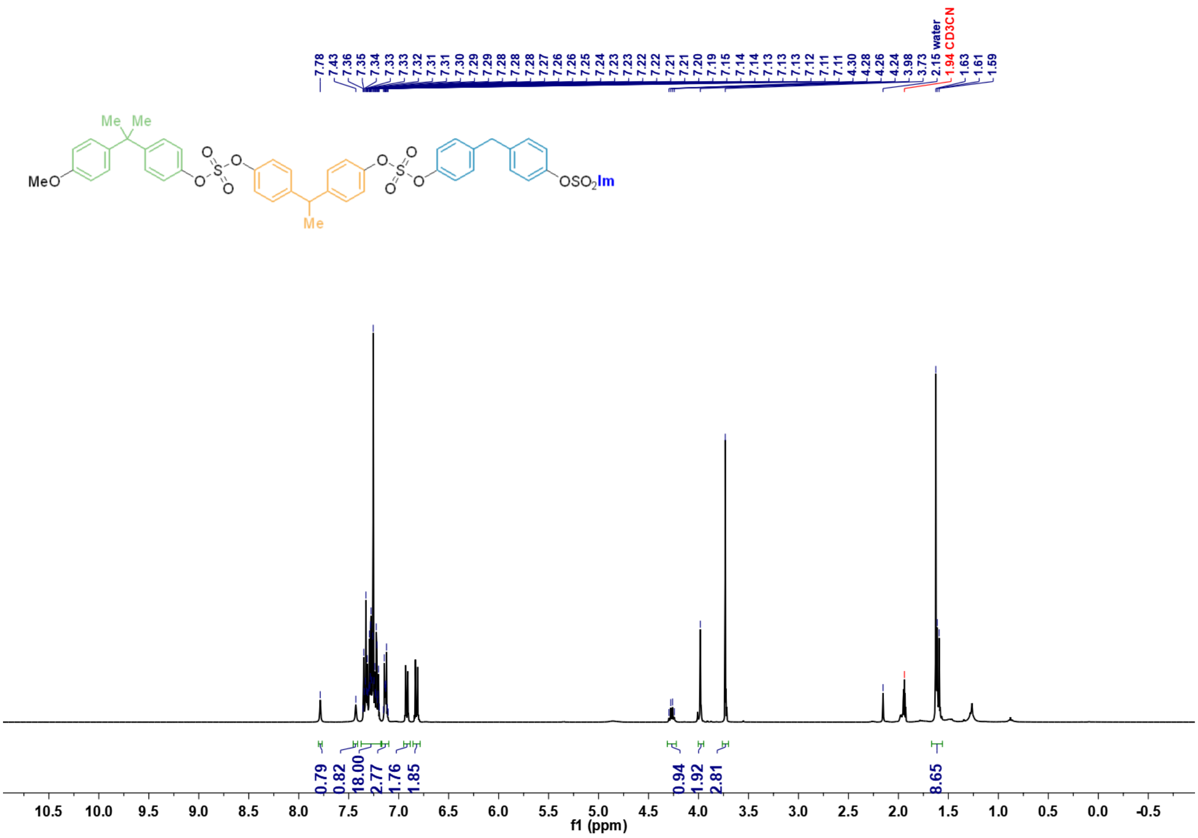

^{13}C NMR (101 MHz, CD_3CN) of **6**

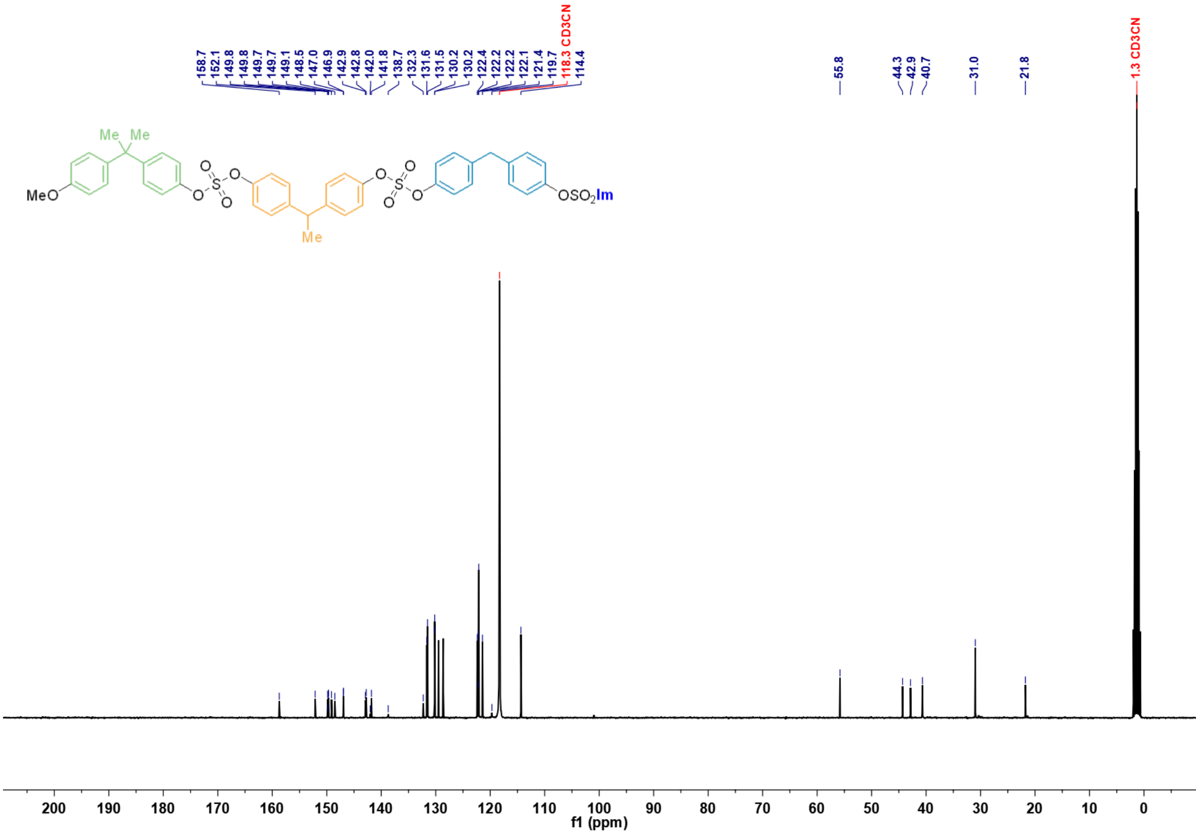

^{1}H NMR (400 MHz, CD_3CN) of **7**

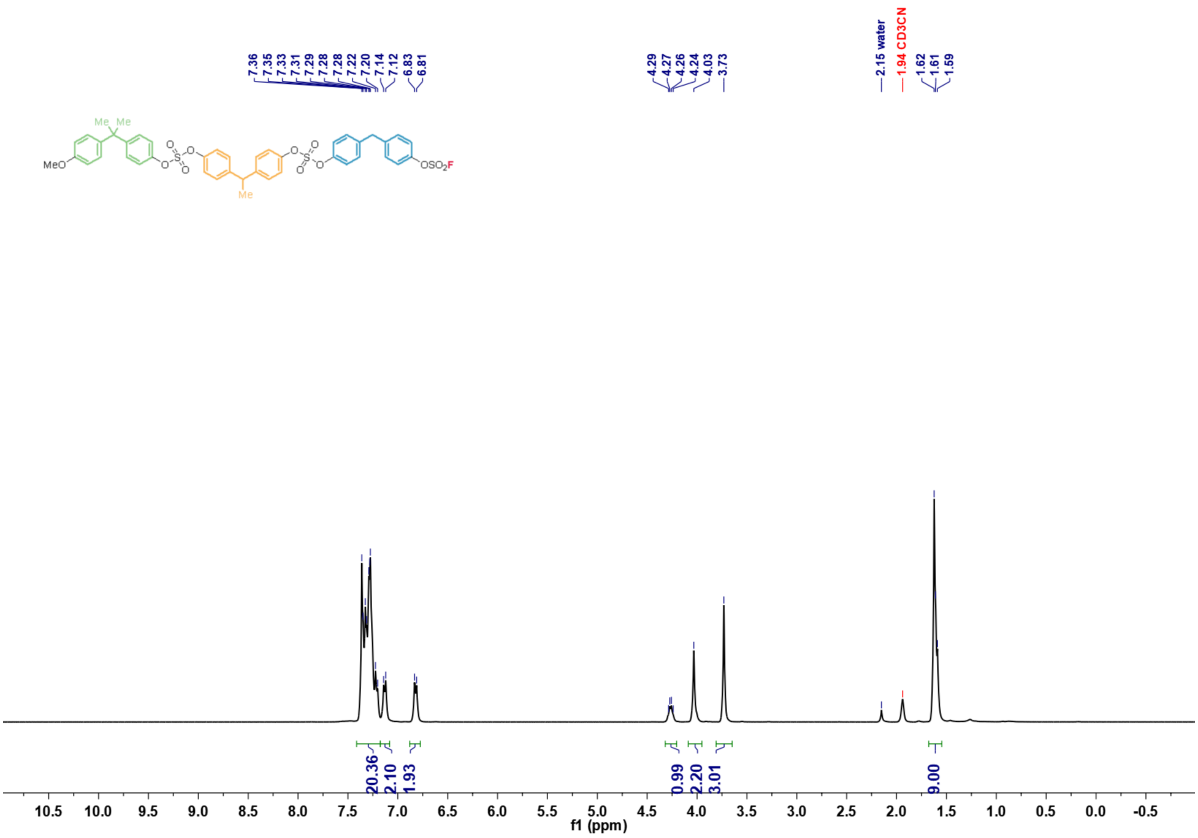

^{13}C NMR (101 MHz, CD_3CN) of **7**

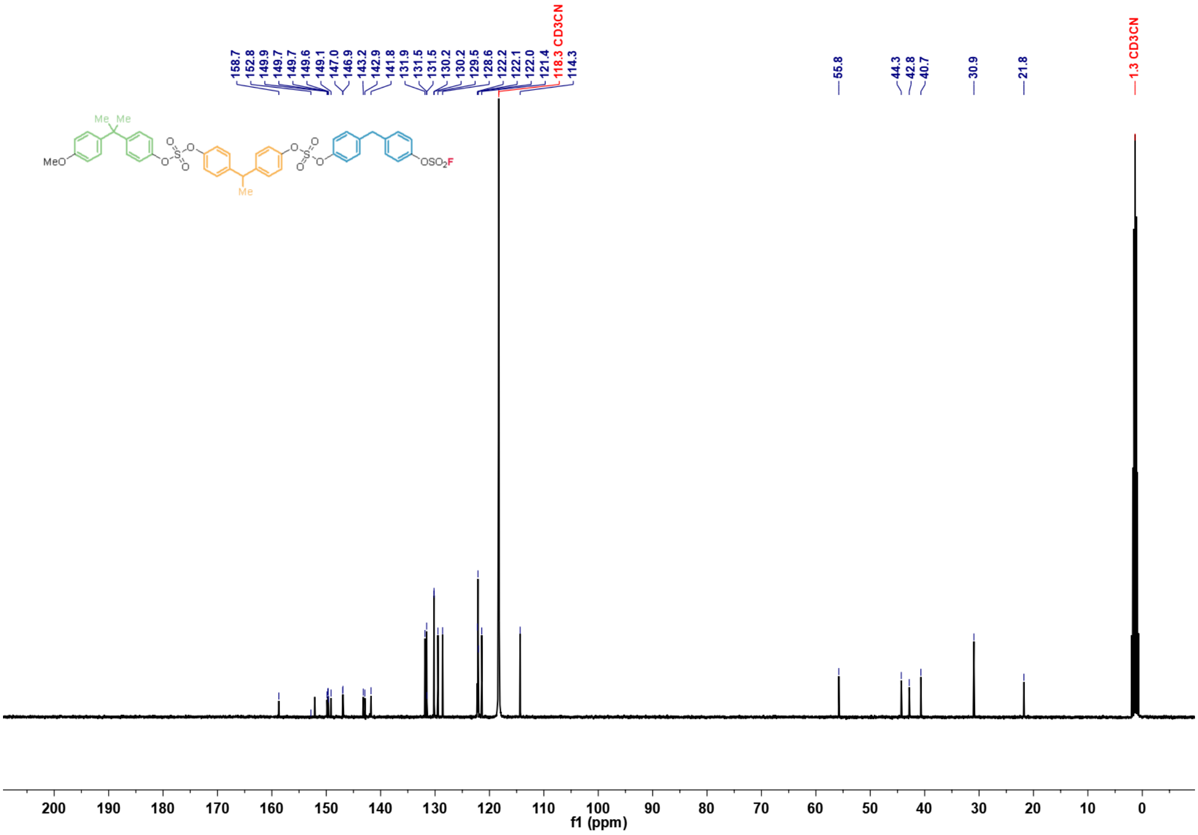

^{19}F NMR (376 MHz, CD_3CN) of **7**

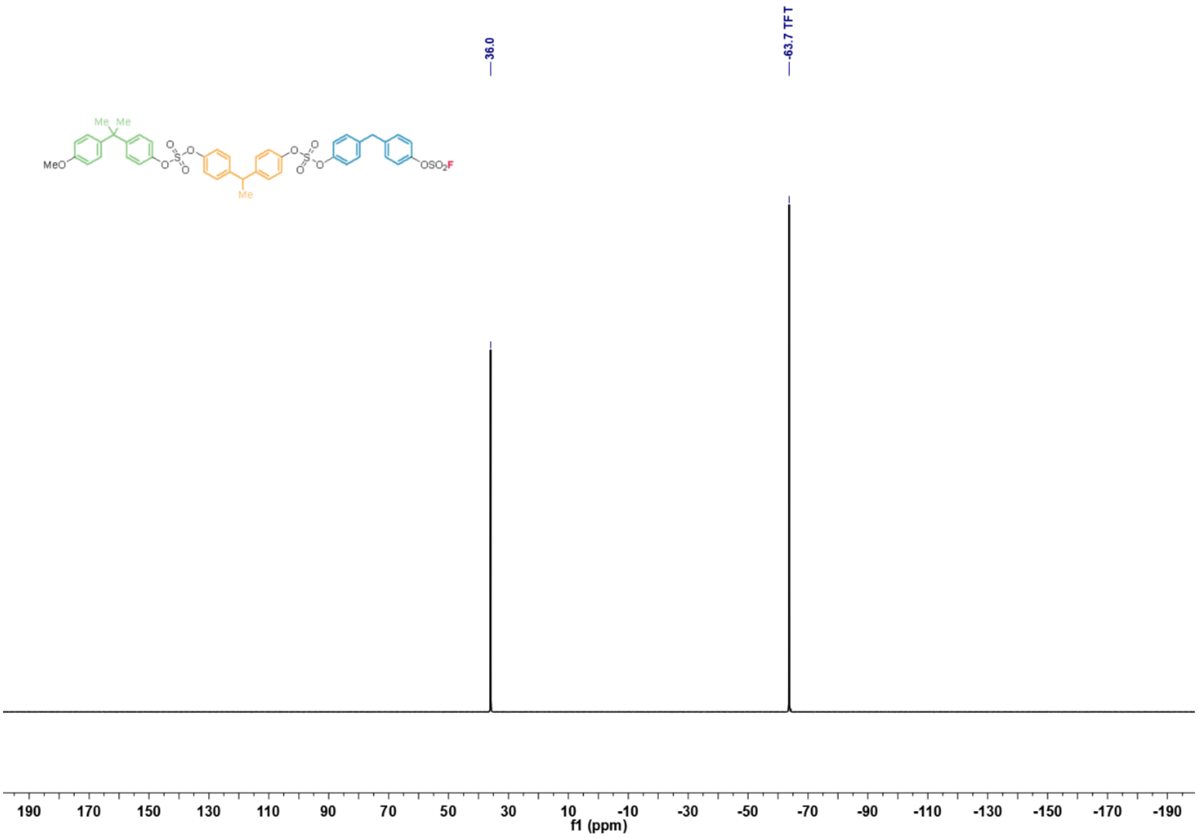

^{1}H NMR (400 MHz, $CDCl_3$) of **8**

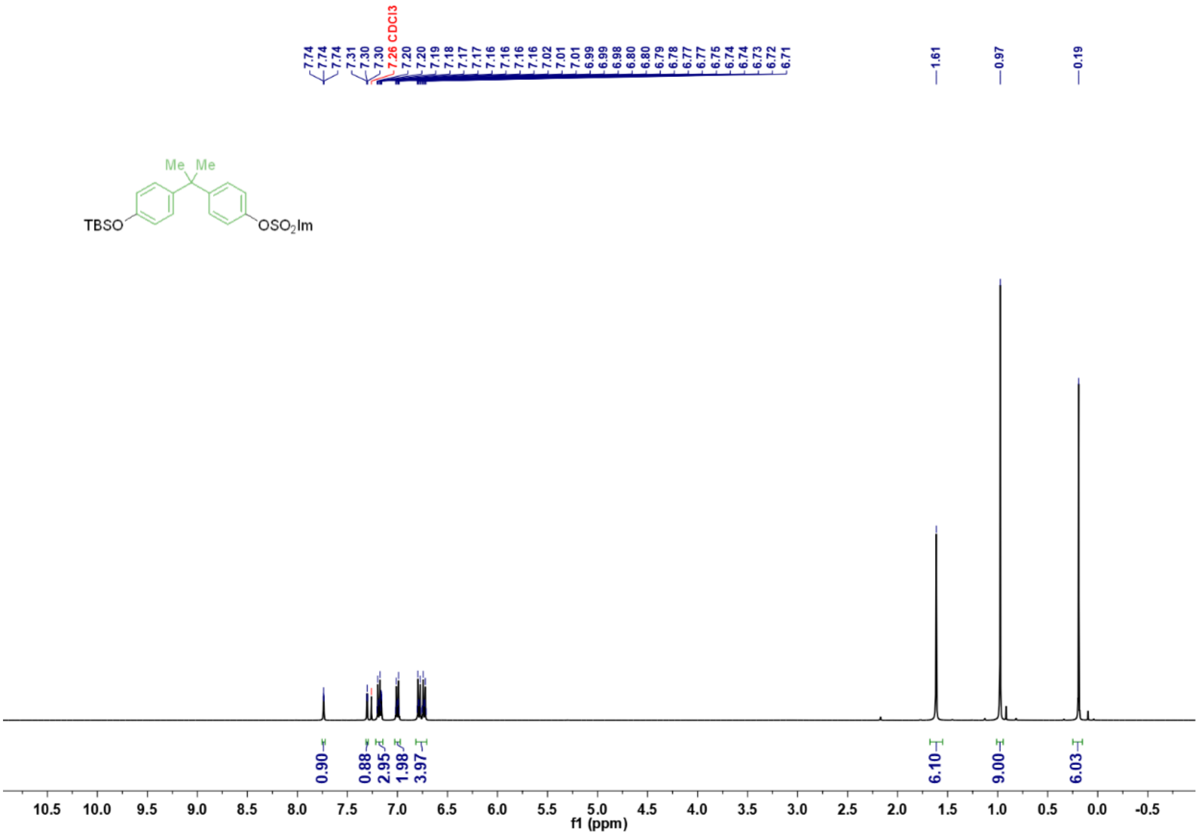

^{13}C NMR (101 MHz, $CDCl_3$) of **8**

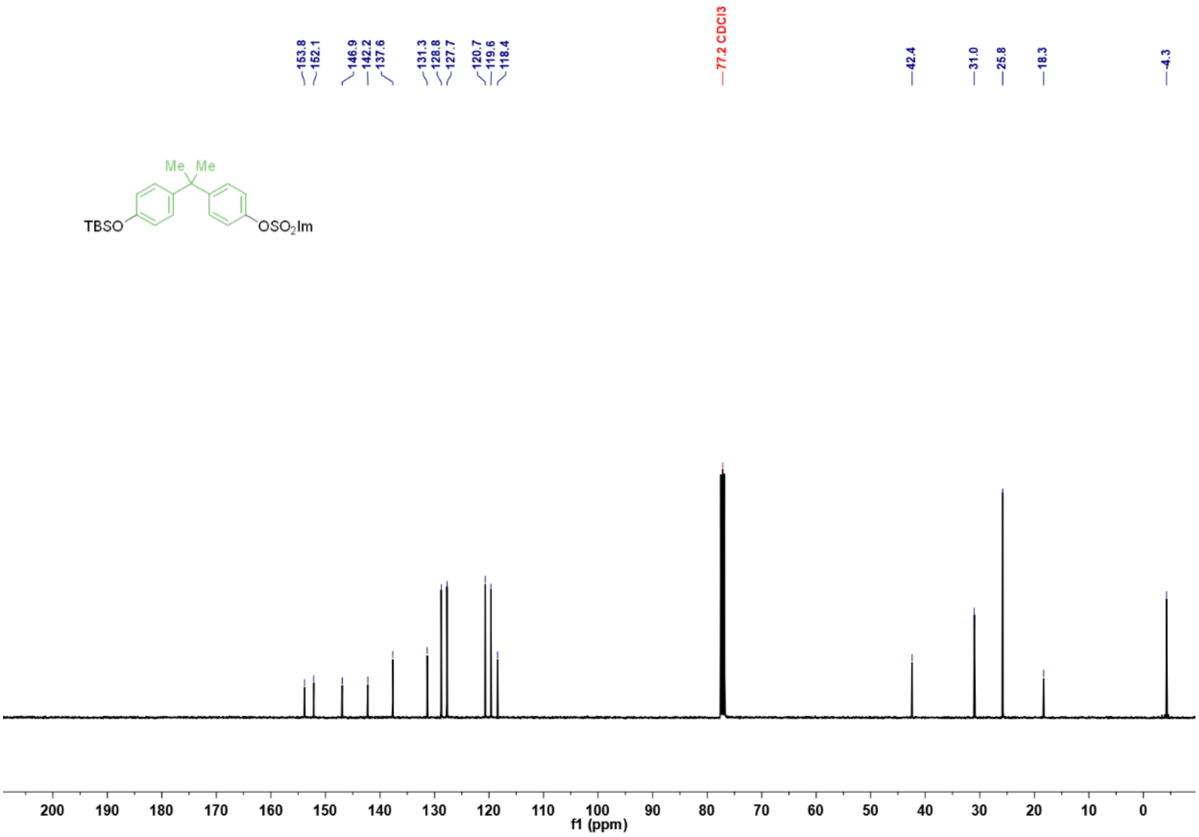

^{1}H NMR (400 MHz, CD_3CN) of **9**

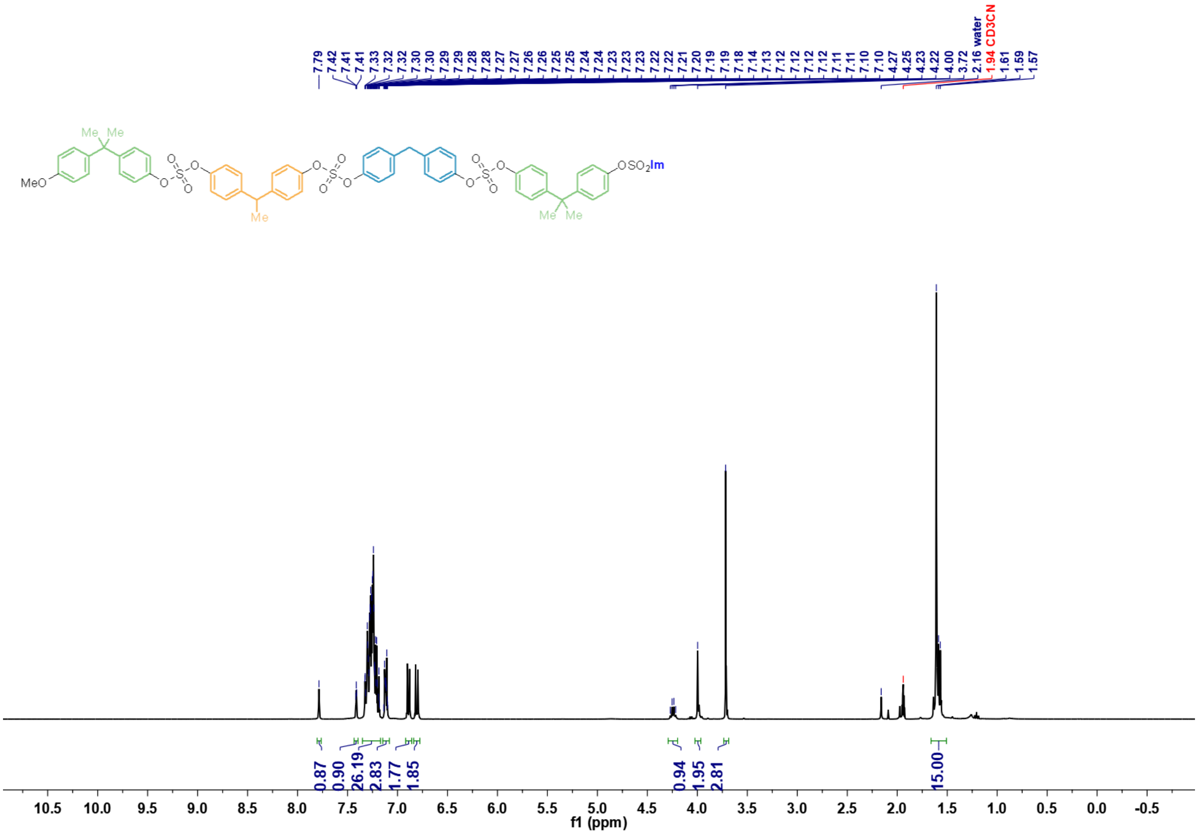

^{13}C NMR (101 MHz, CD_3CN) of **9**

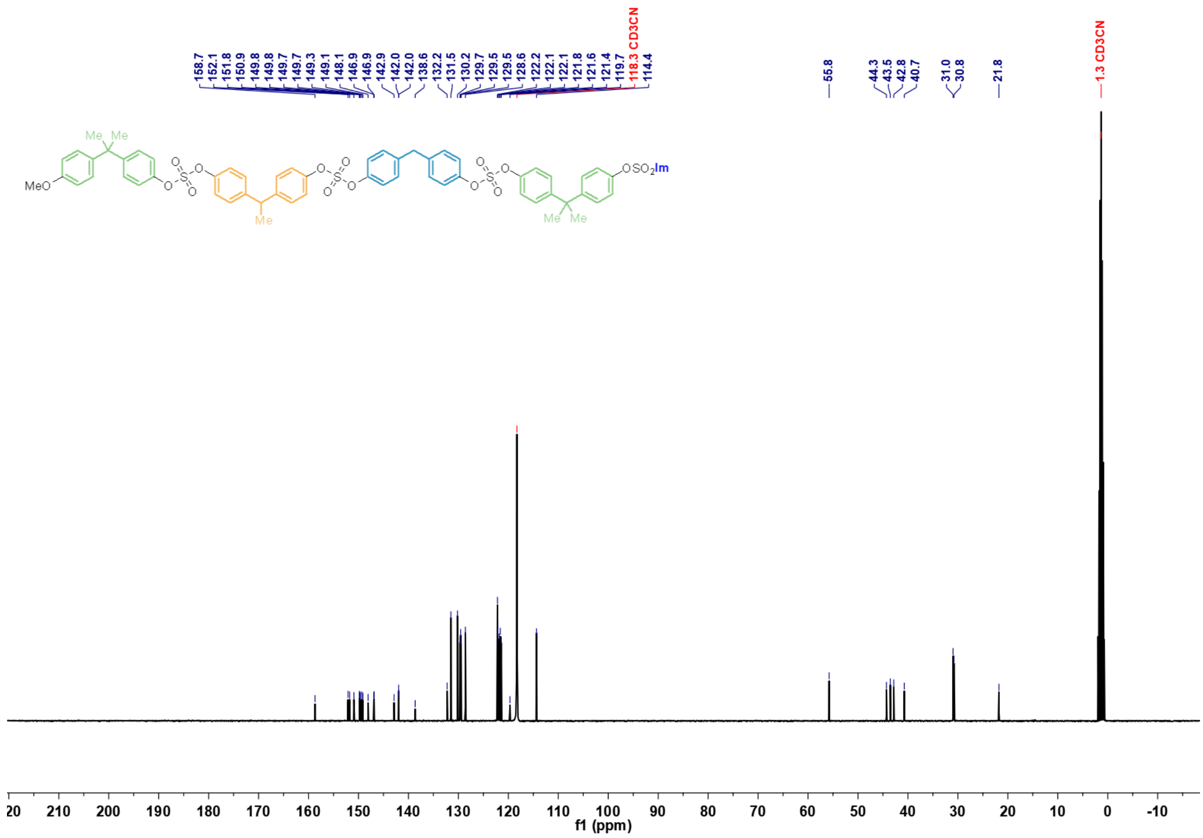

^{1}H NMR (400 MHz, CD_3CN) of **10**

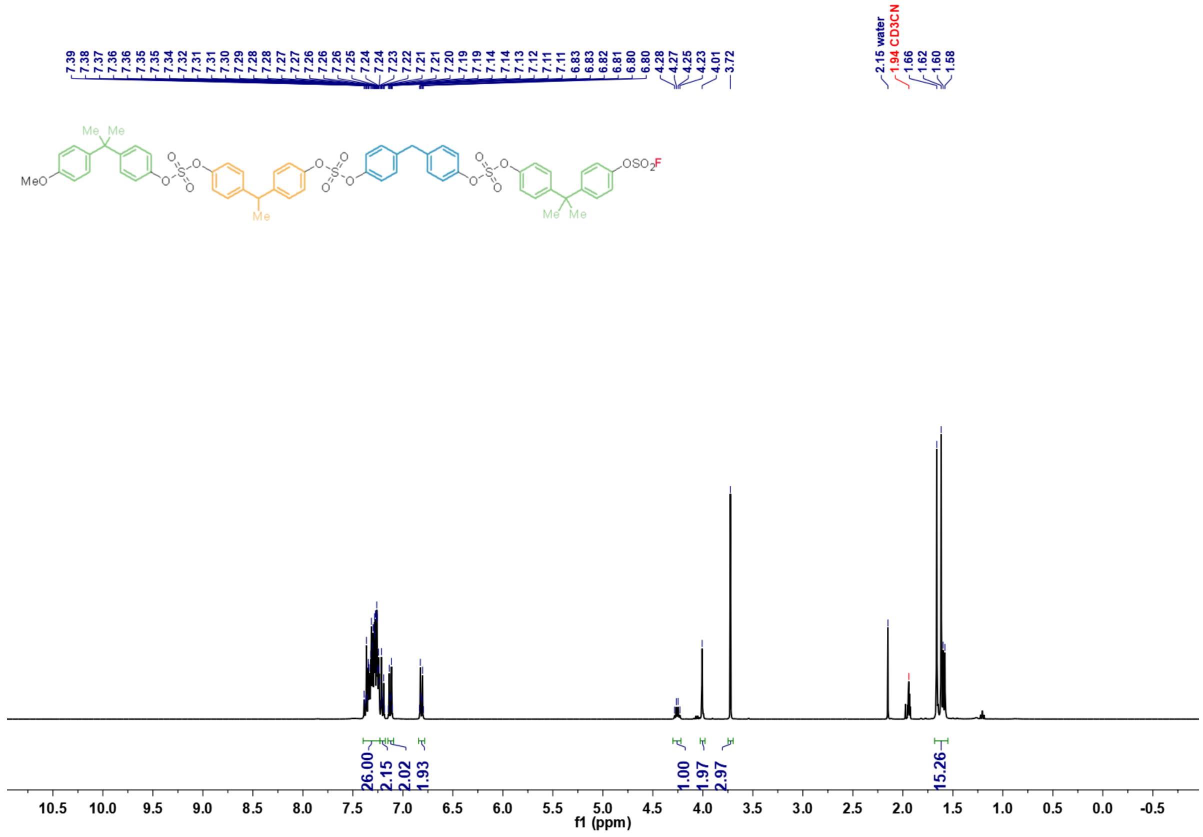

^{13}C NMR (101 MHz, CD_3CN) of **10**

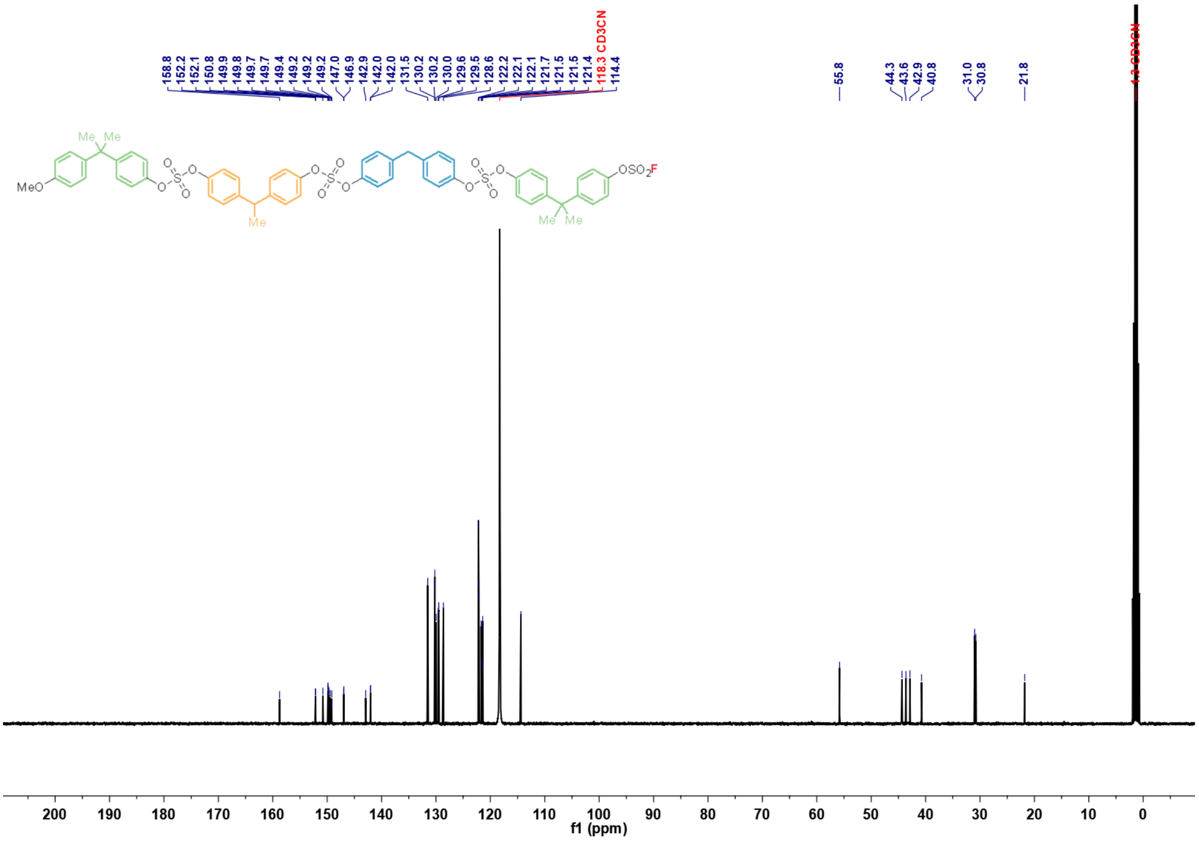

^{19}F NMR (376 MHz, CD_3CN) of **10**

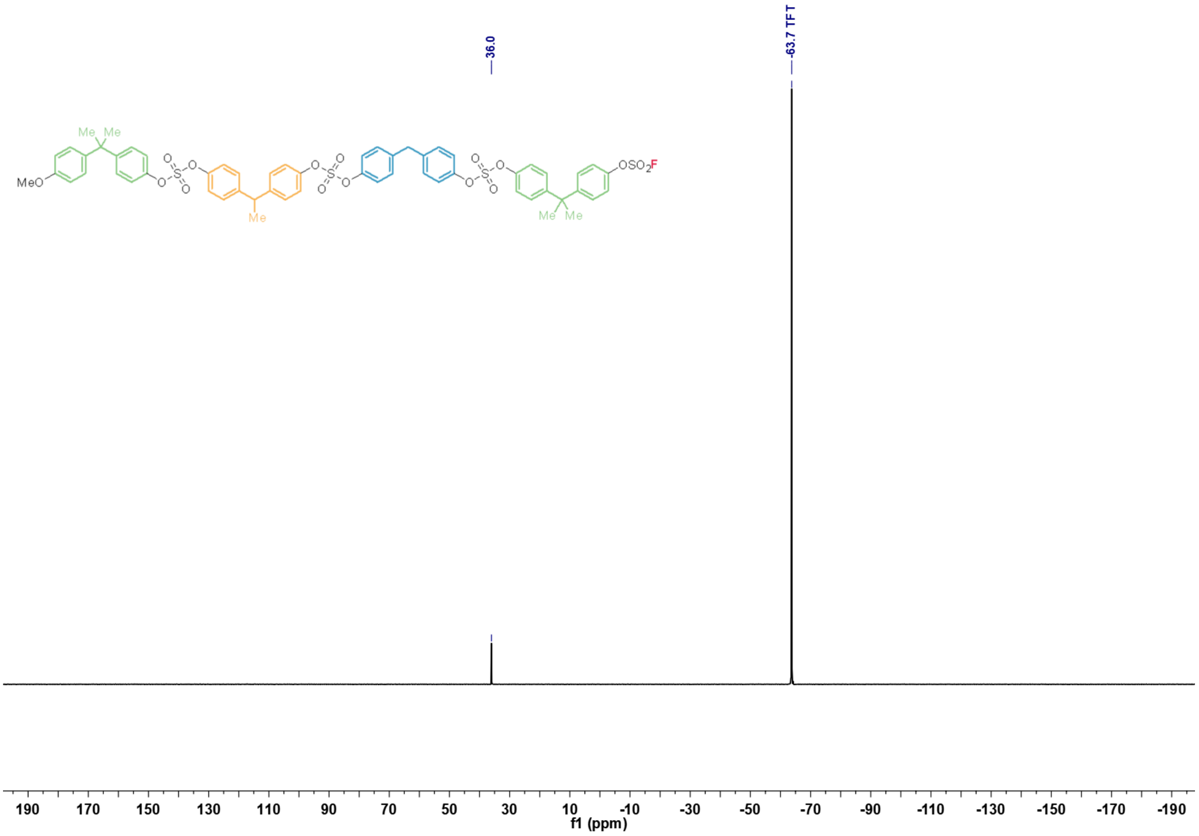

^{1}H NMR (400 MHz, CD_3CN) of **11**

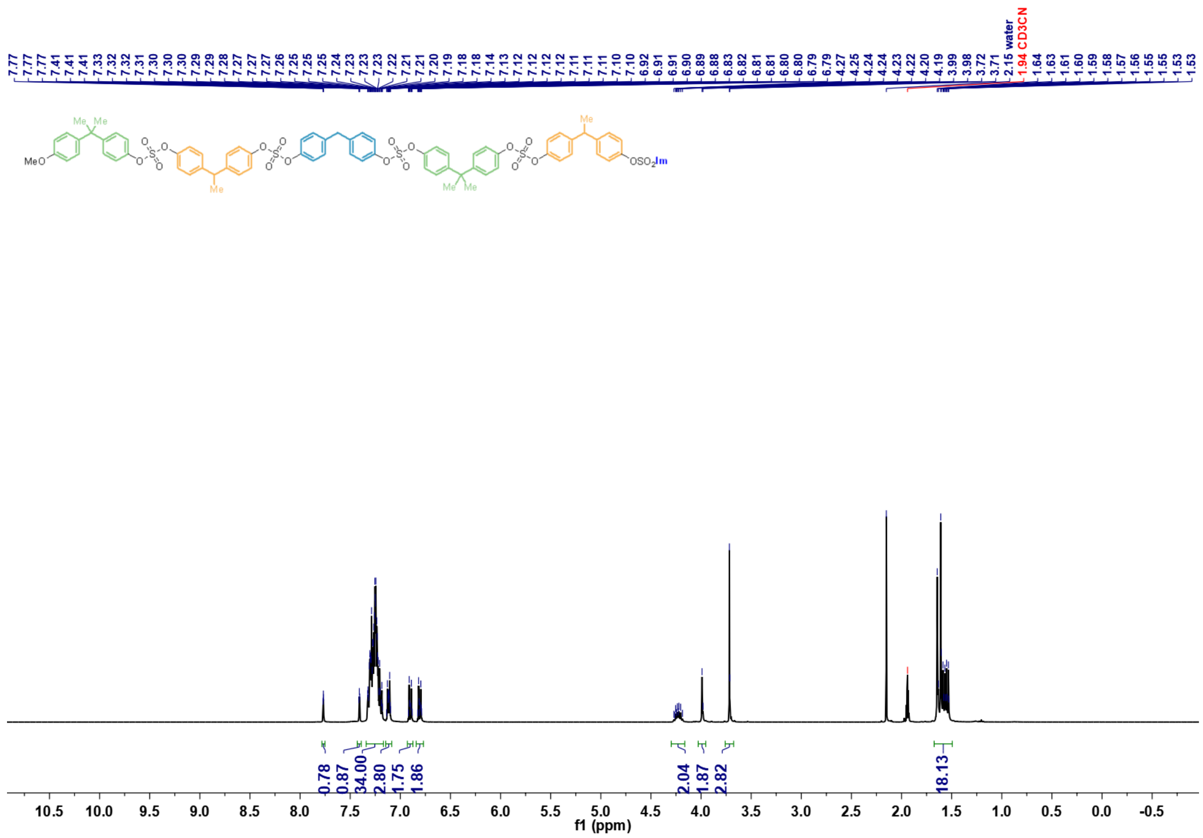

^{13}C NMR (101 MHz, CD_3CN) of **11**

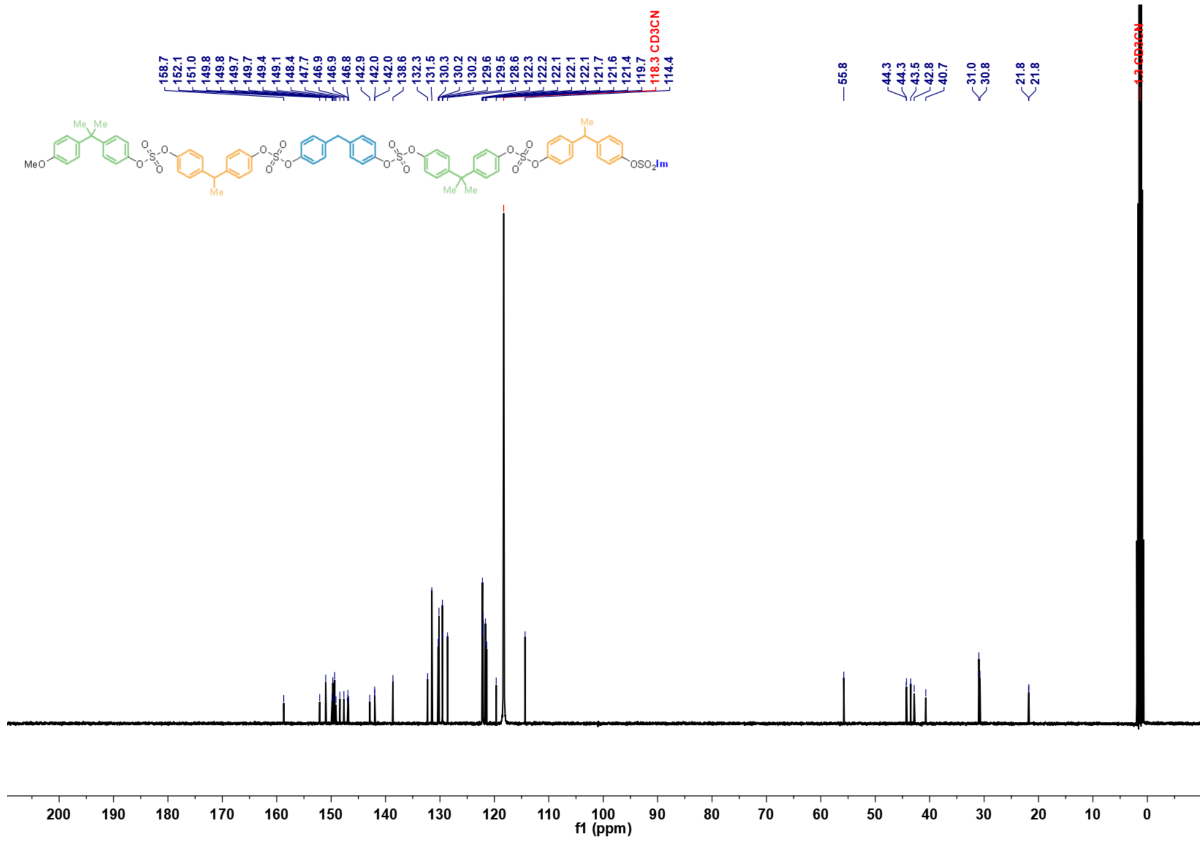

^{1}H NMR (400 MHz, CD_3CN) of **12**

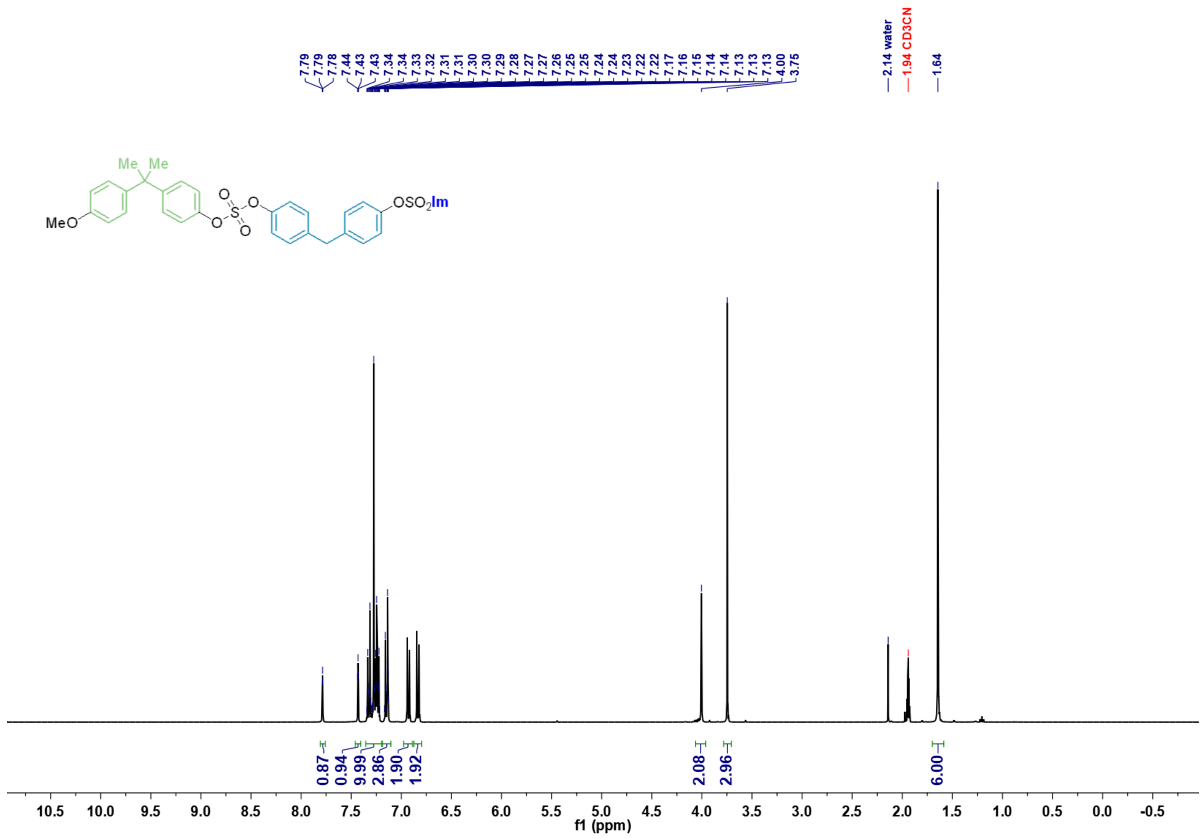

^{13}C NMR (101 MHz, CD_3CN) of **12**

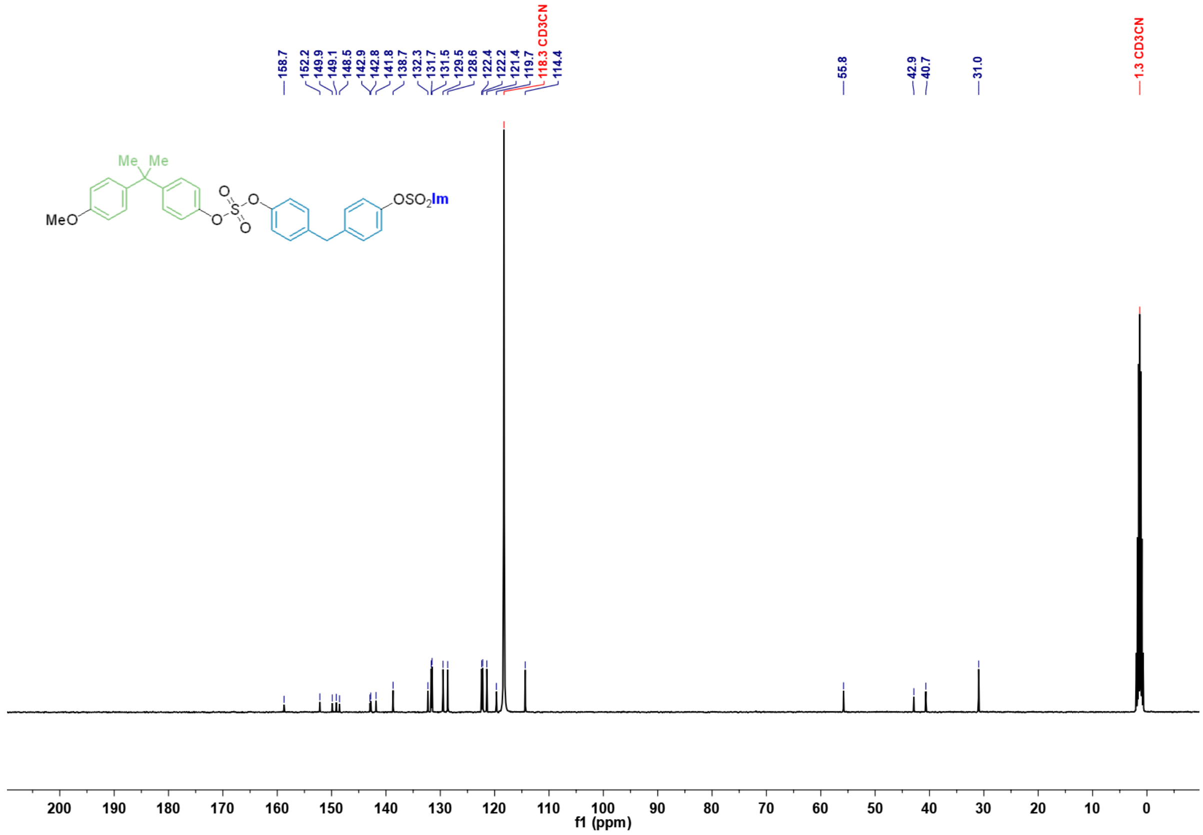

^{1}H NMR (400 MHz, CD_3CN) of **13**

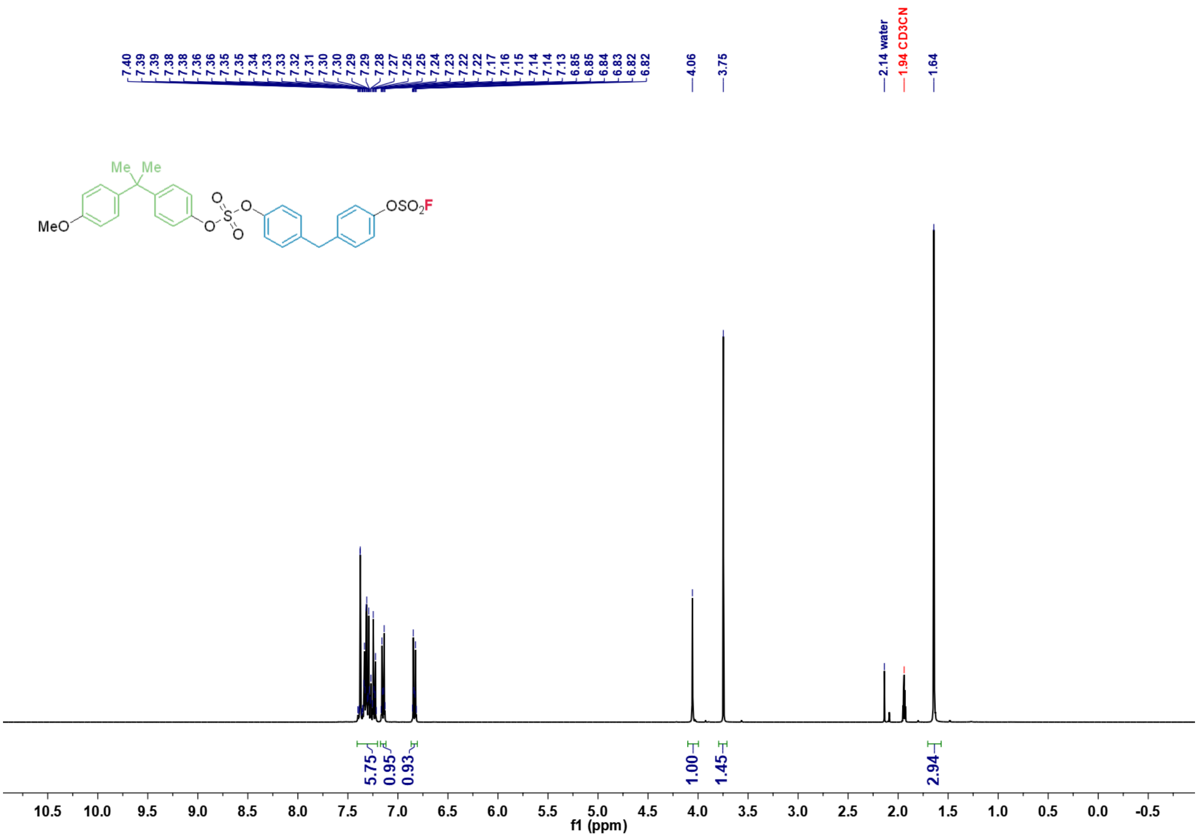

^{13}C NMR (101 MHz, CD_3CN) of **13**

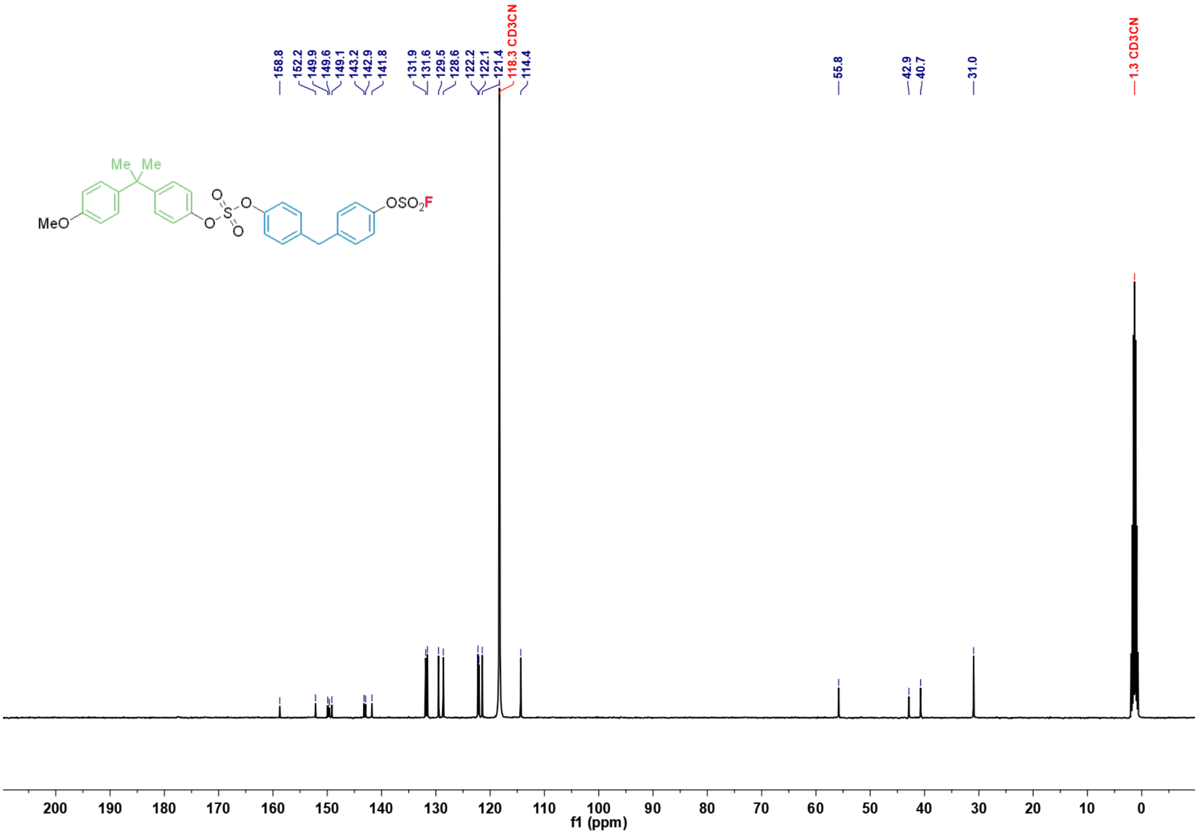

^{19}F NMR (376 MHz, CD_3CN) of **13**

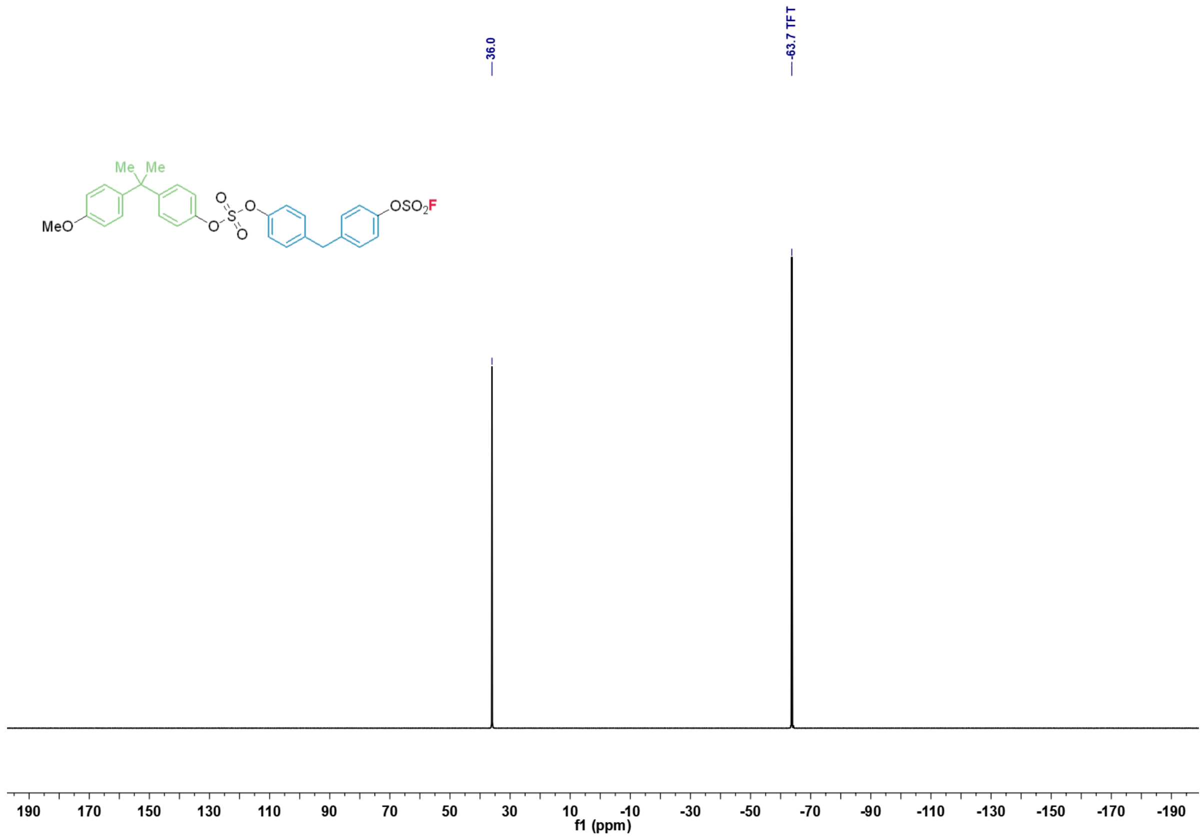

^{1}H NMR (400 MHz, CD_3CN) of **14**

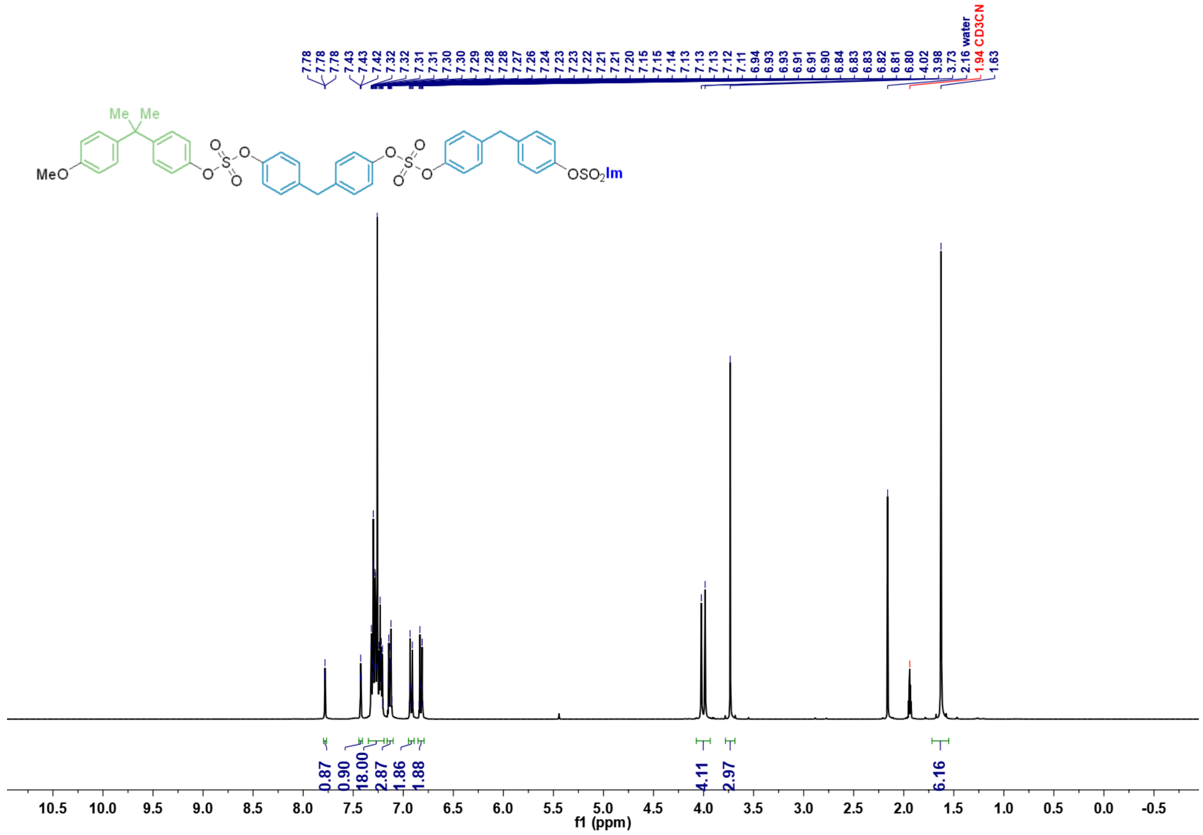

^{13}C NMR (101 MHz, CD_3CN) of **14**

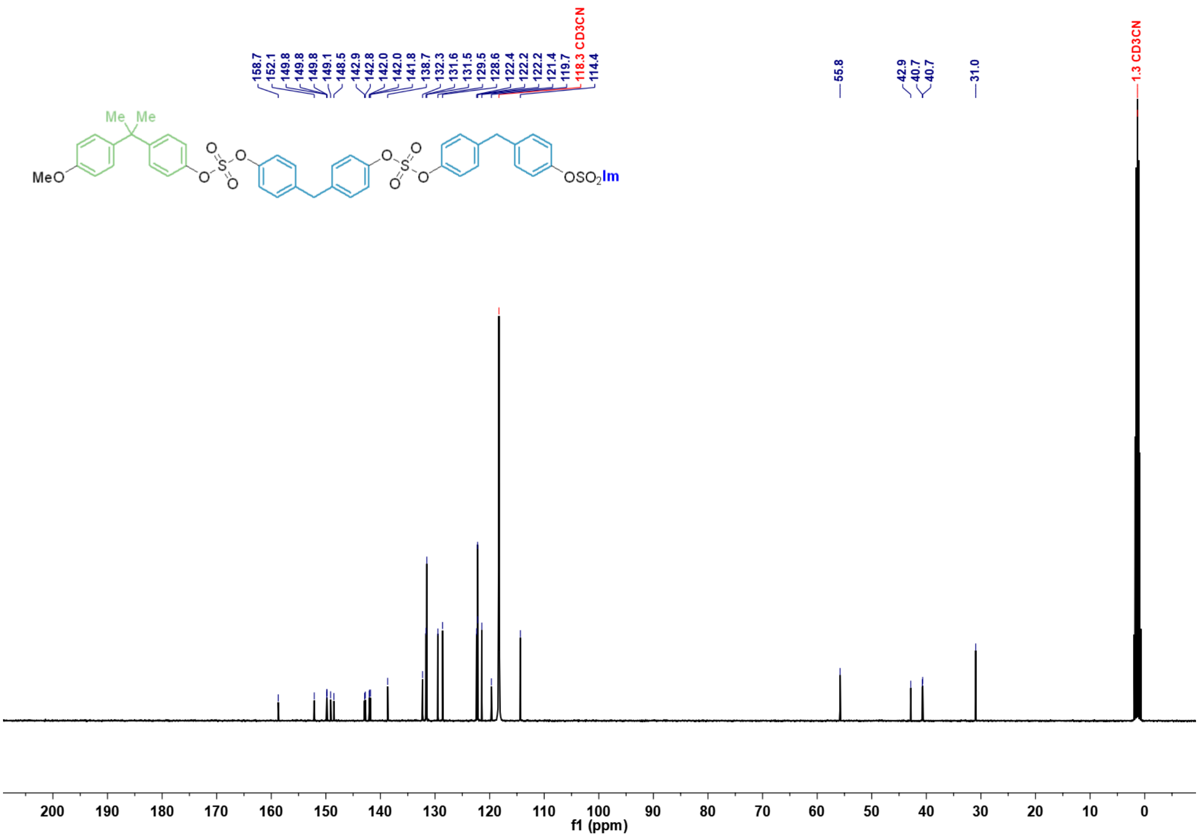

^{1}H NMR (400 MHz, CD_3CN) of **15**

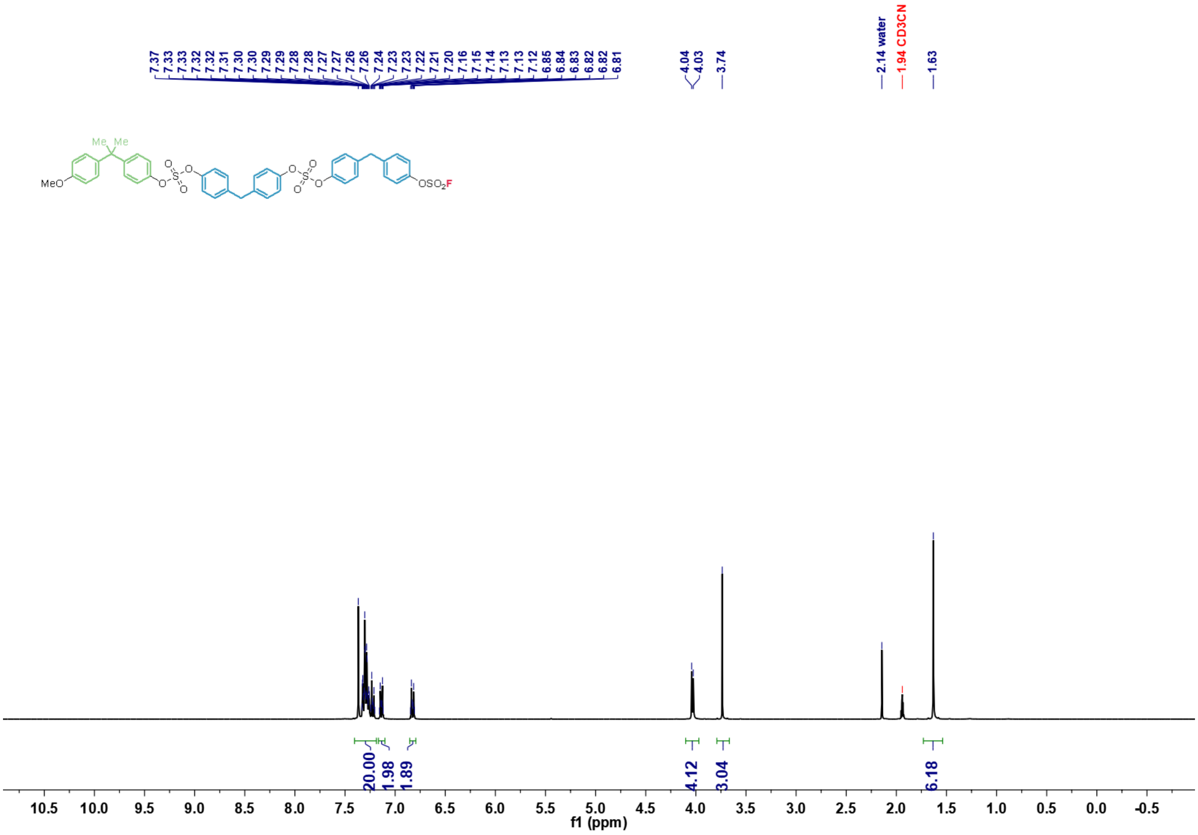

^{13}C NMR (101 MHz, CD_3CN) of **15**

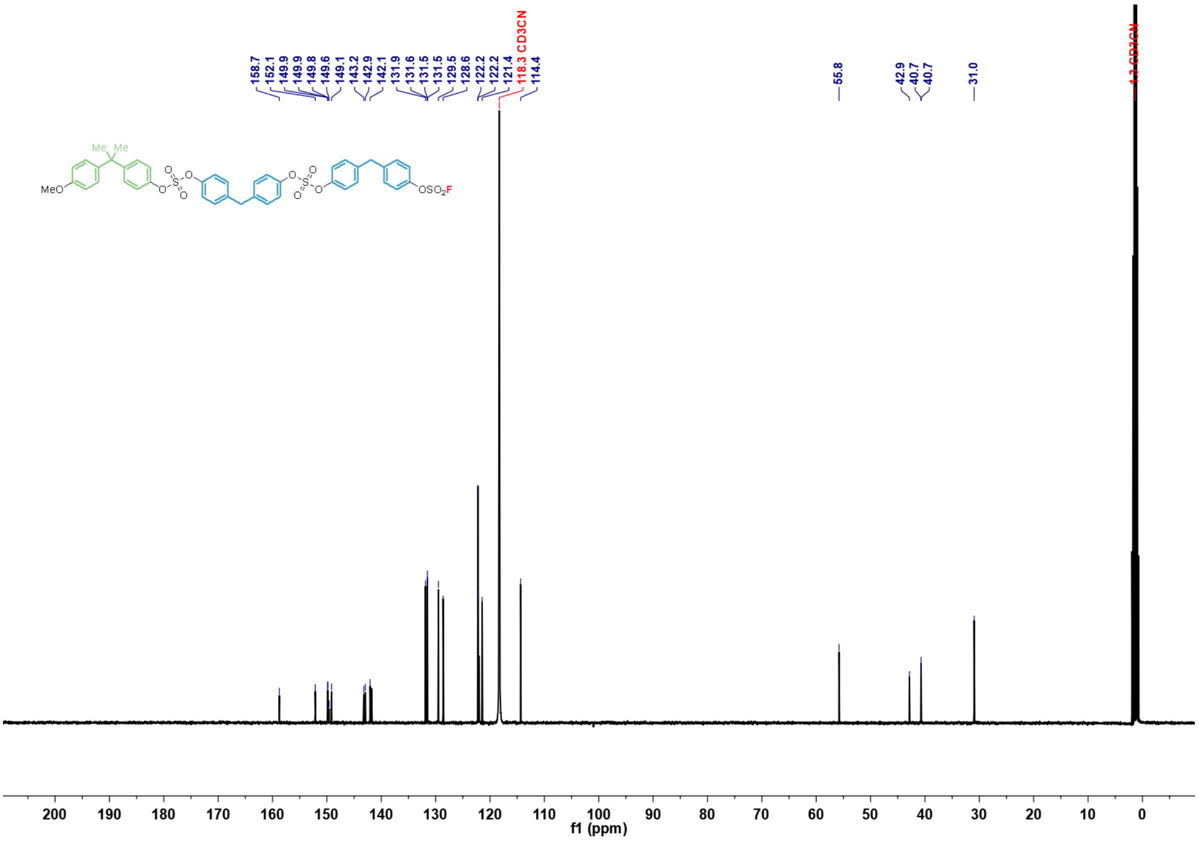

^{19}F NMR (376 MHz, CD_3CN) of **15**

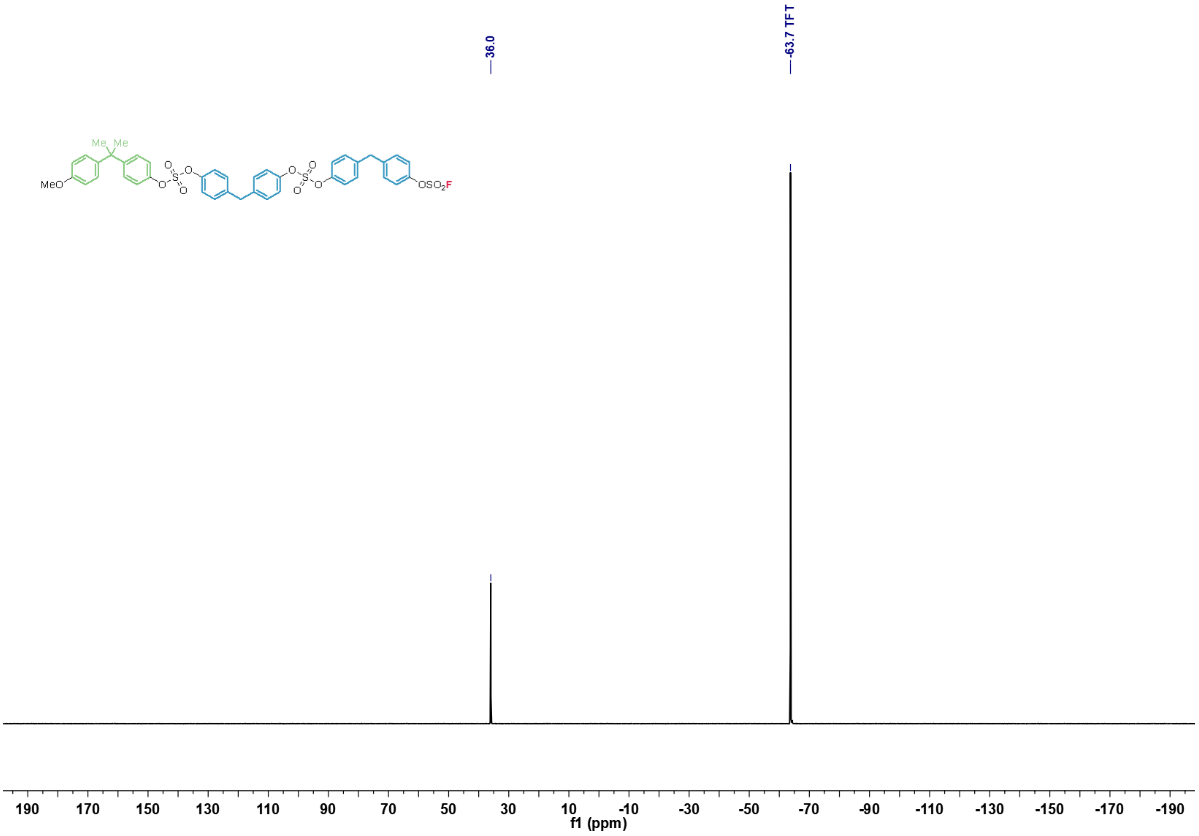

^{1}H NMR (400 MHz, CD_3CN) of **16**

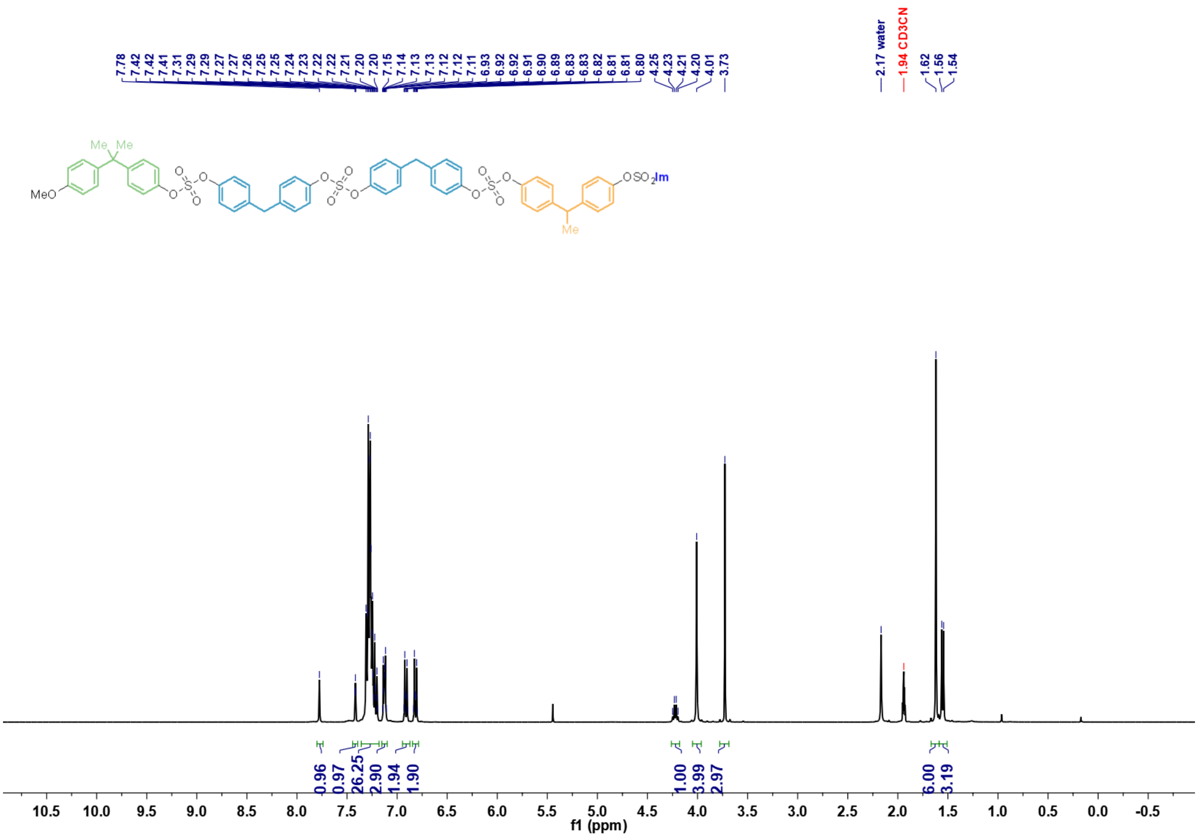

^{13}C NMR (101 MHz, CD_3CN) of **16**

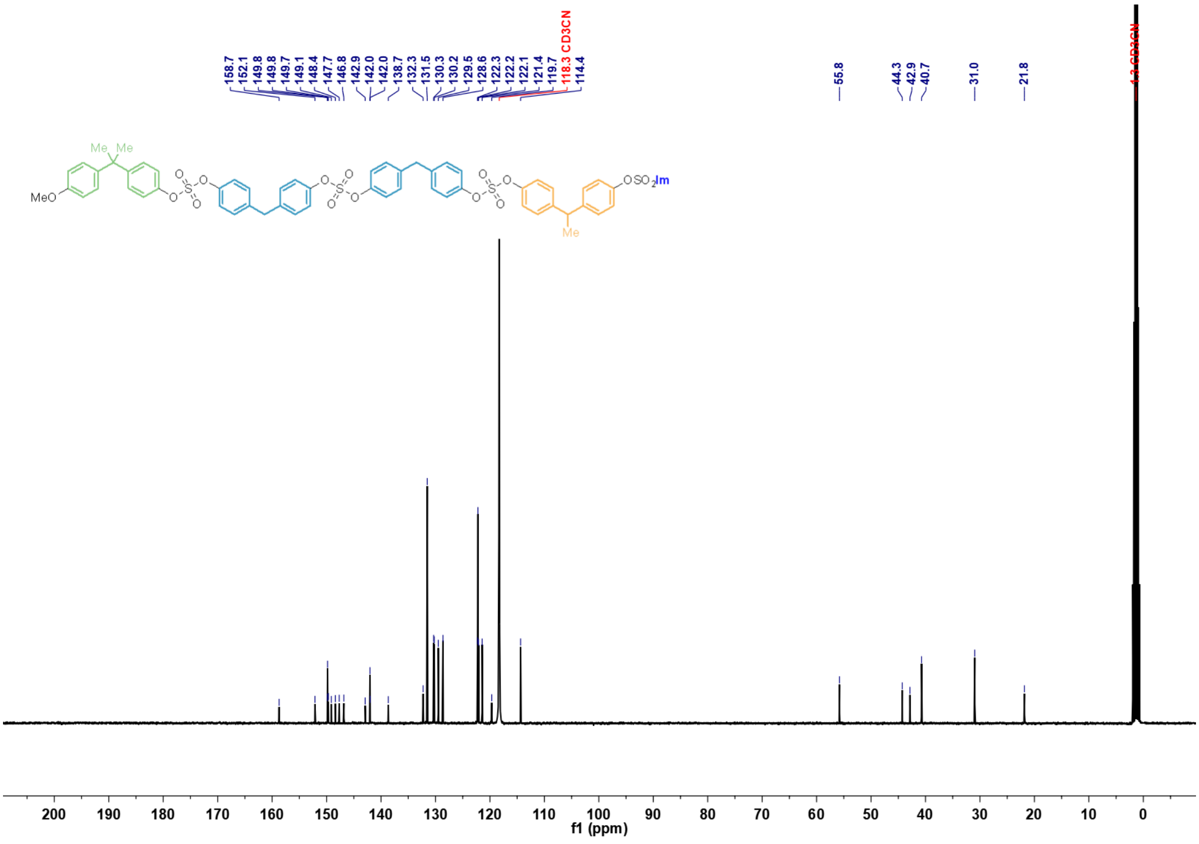

^{1}H NMR (400 MHz, CD_3CN) of **17**

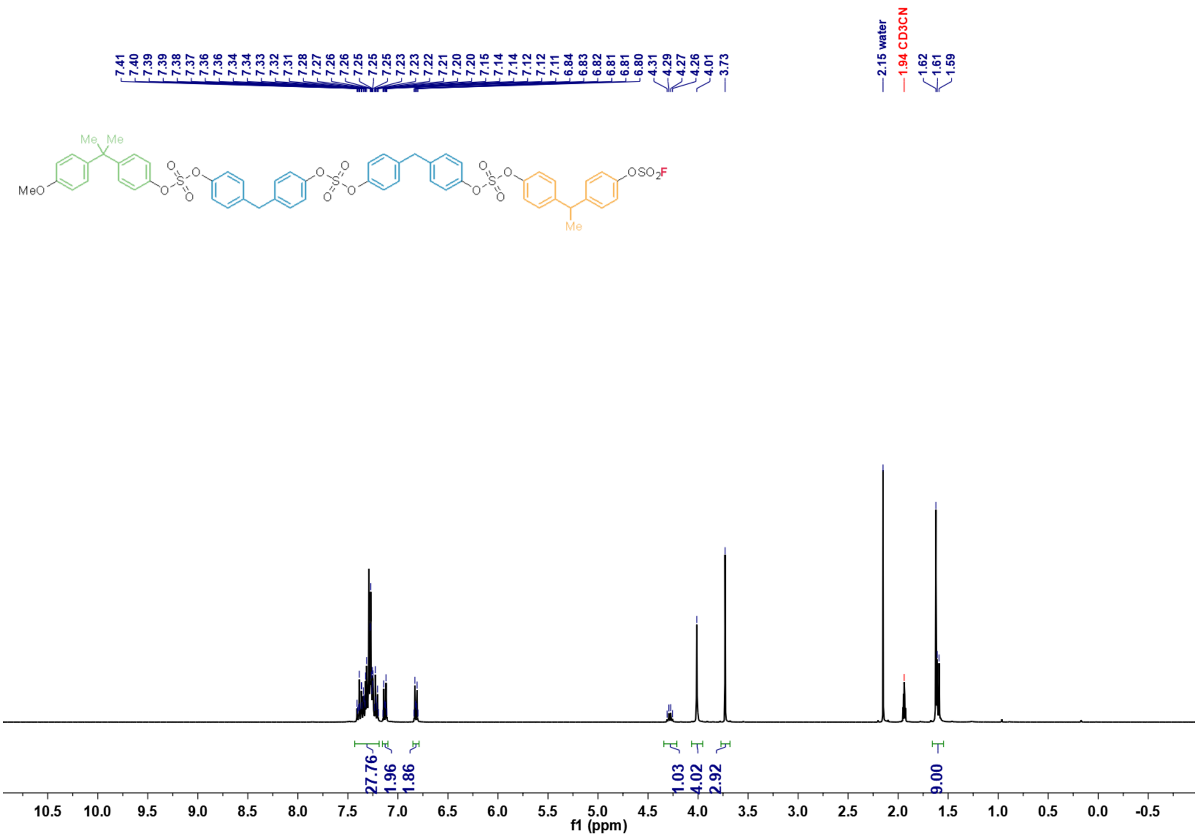

^{13}C NMR (101 MHz, CD_3CN) of **17**

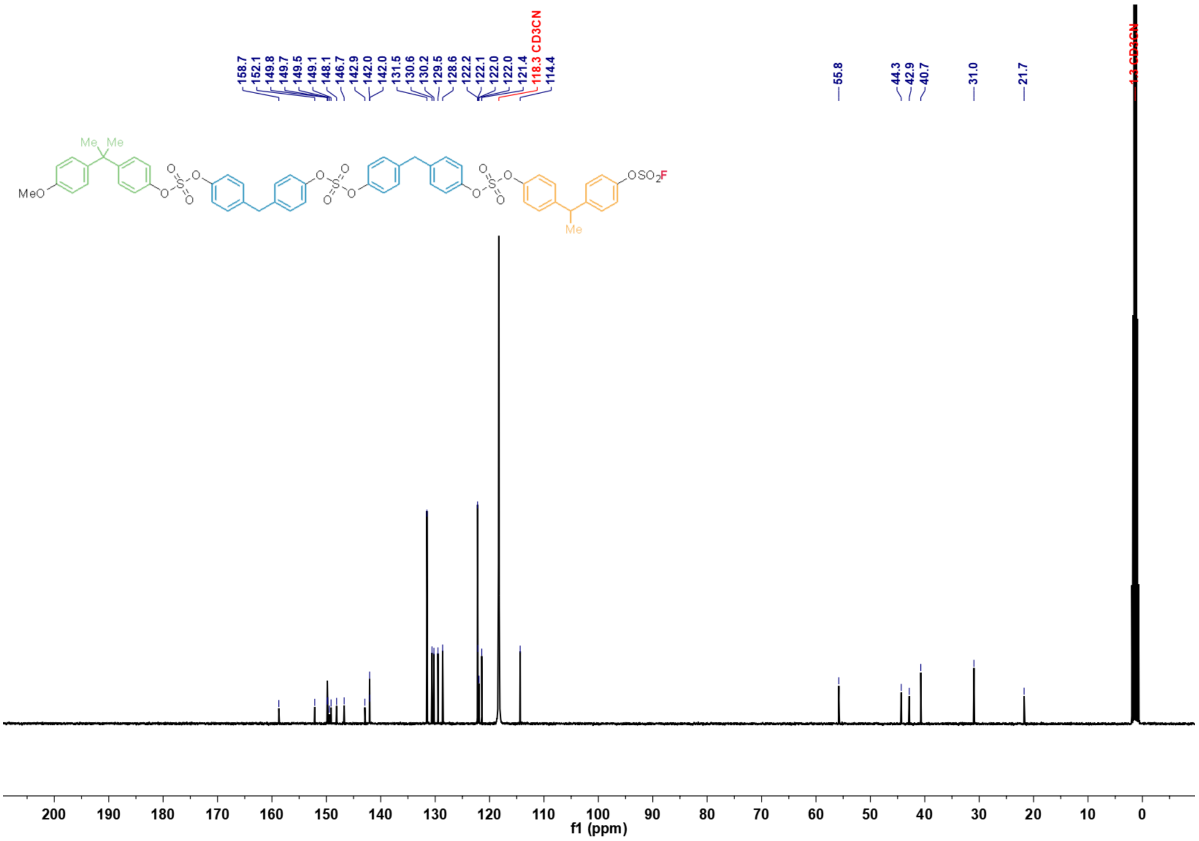

^{19}F NMR (376 MHz, CD_3CN) of **17**

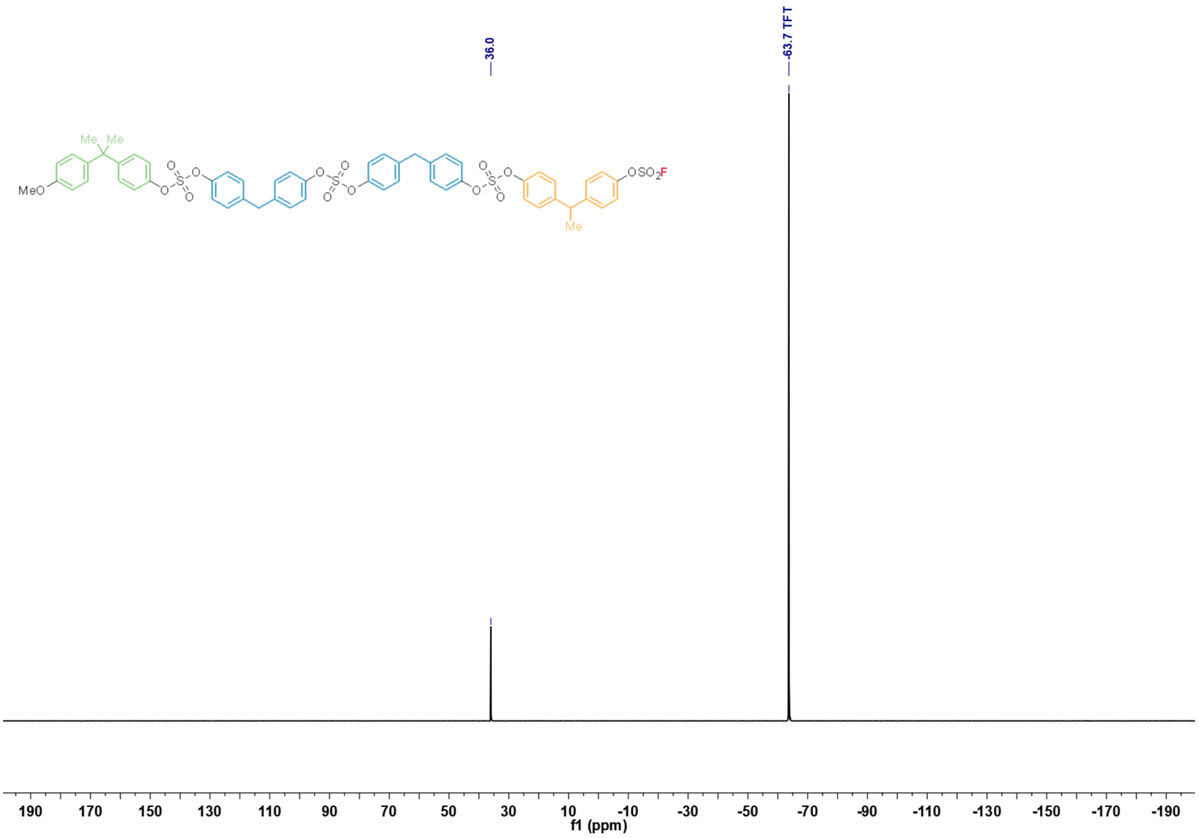

^{1}H NMR (400 MHz, CD_3CN) of **18**

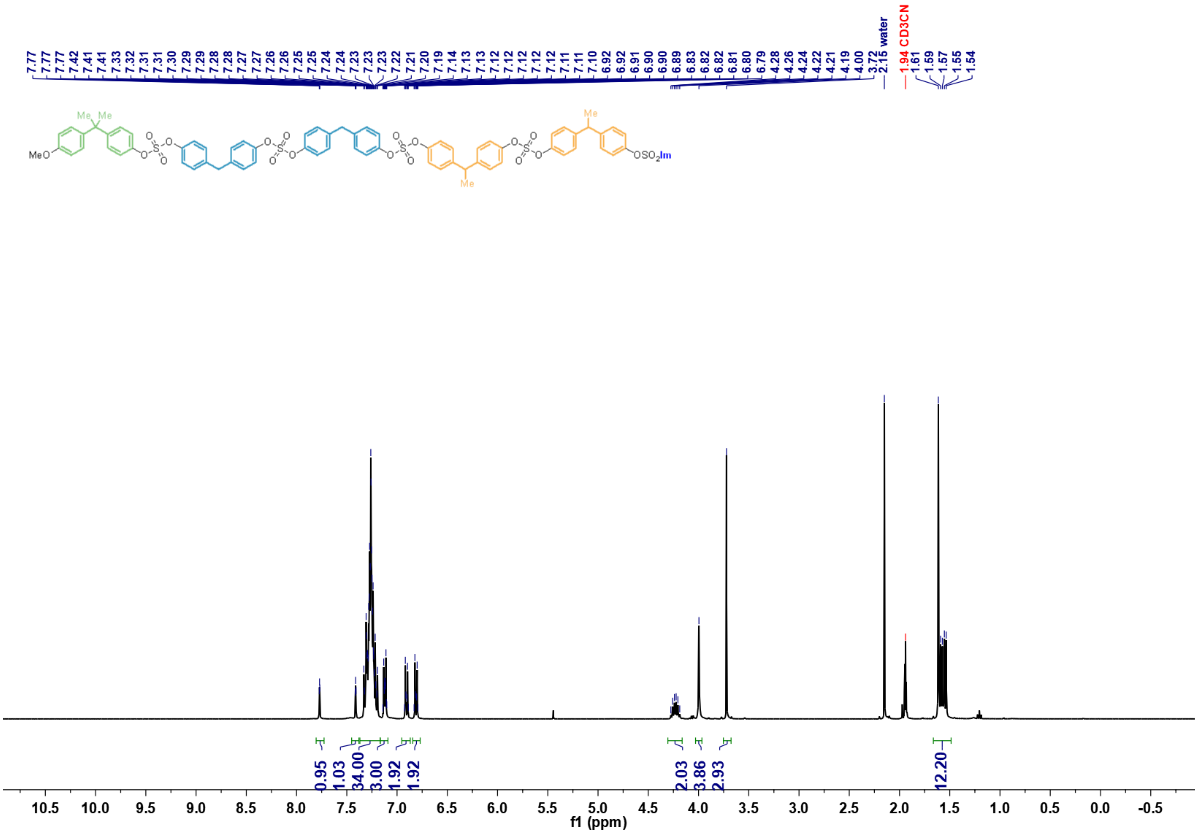

^{13}C NMR (101 MHz, CD_3CN) of **18**

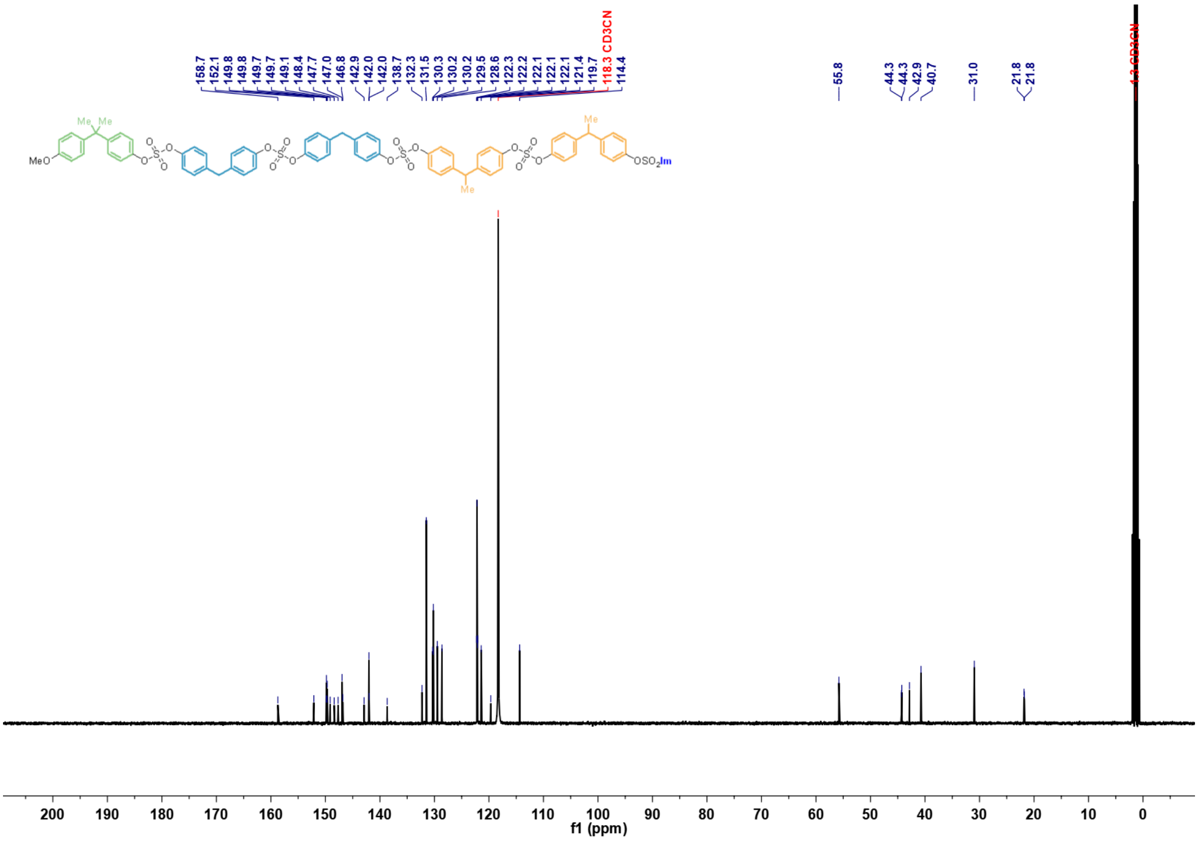

^{1}H NMR (400 MHz, DMSO-d_6) of **19**

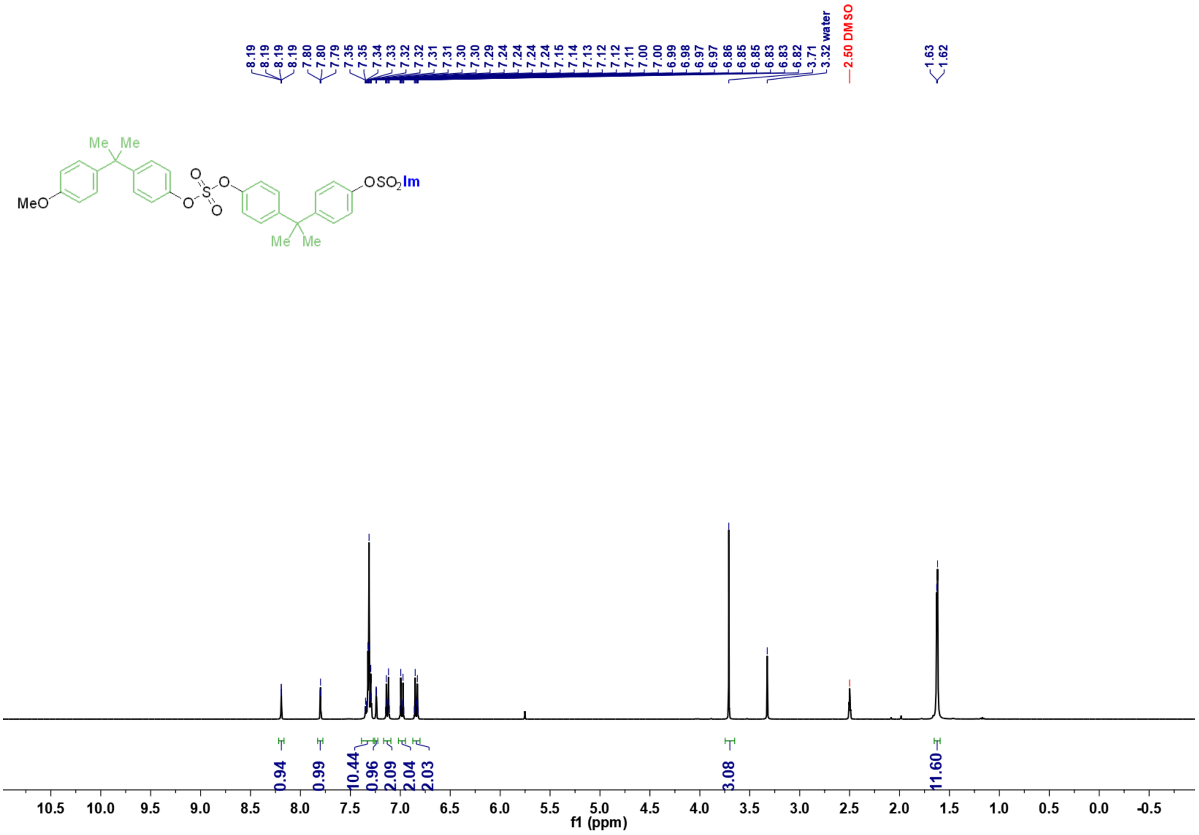

^{13}C NMR (101 MHz, DMSO-d_6) of **19**

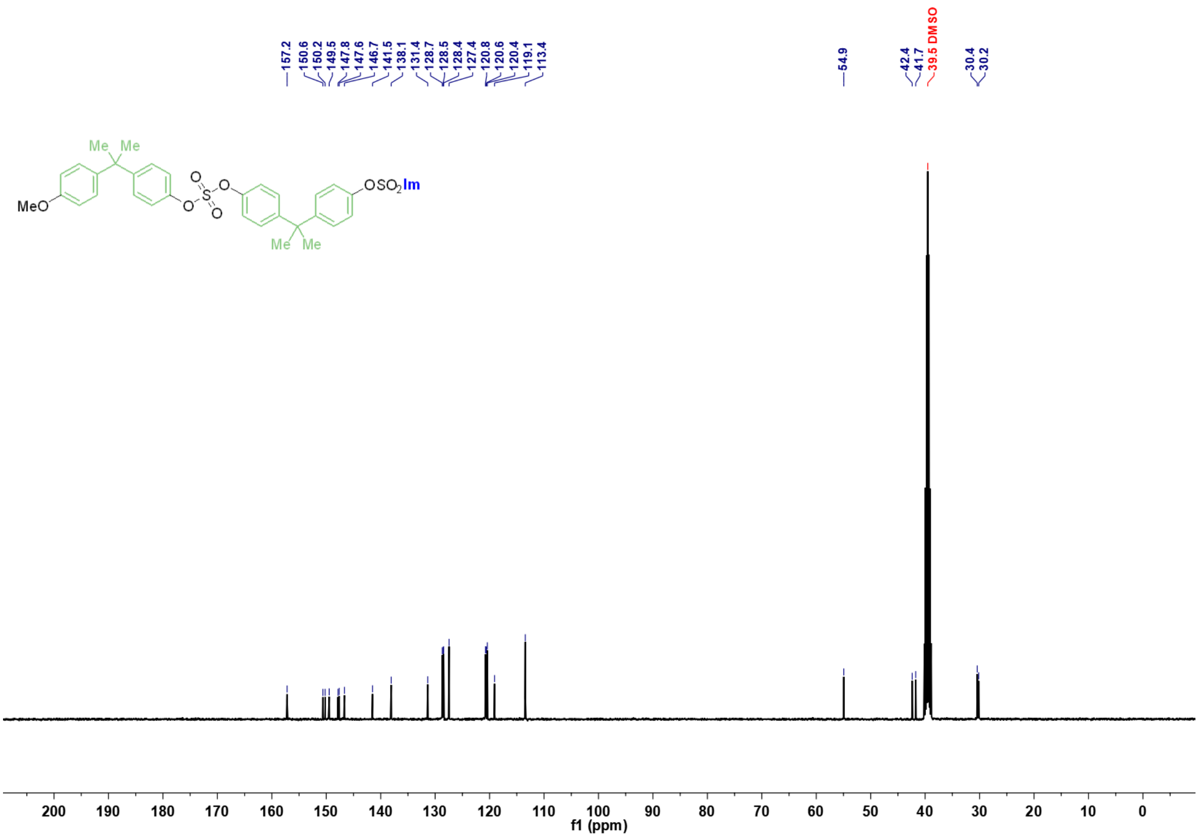

^{1}H NMR (400 MHz, CD_3CN) of **20**

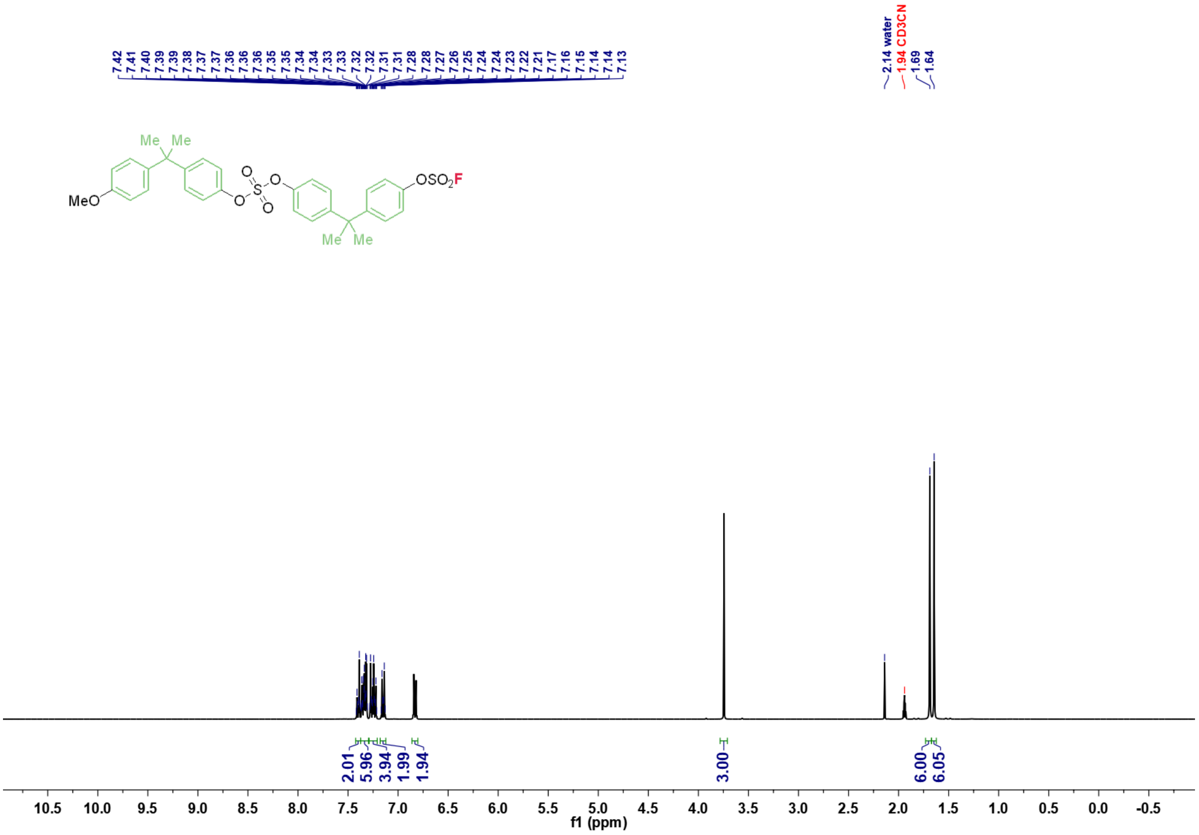

^{13}C NMR (101 MHz, CD_3CN) of **20**

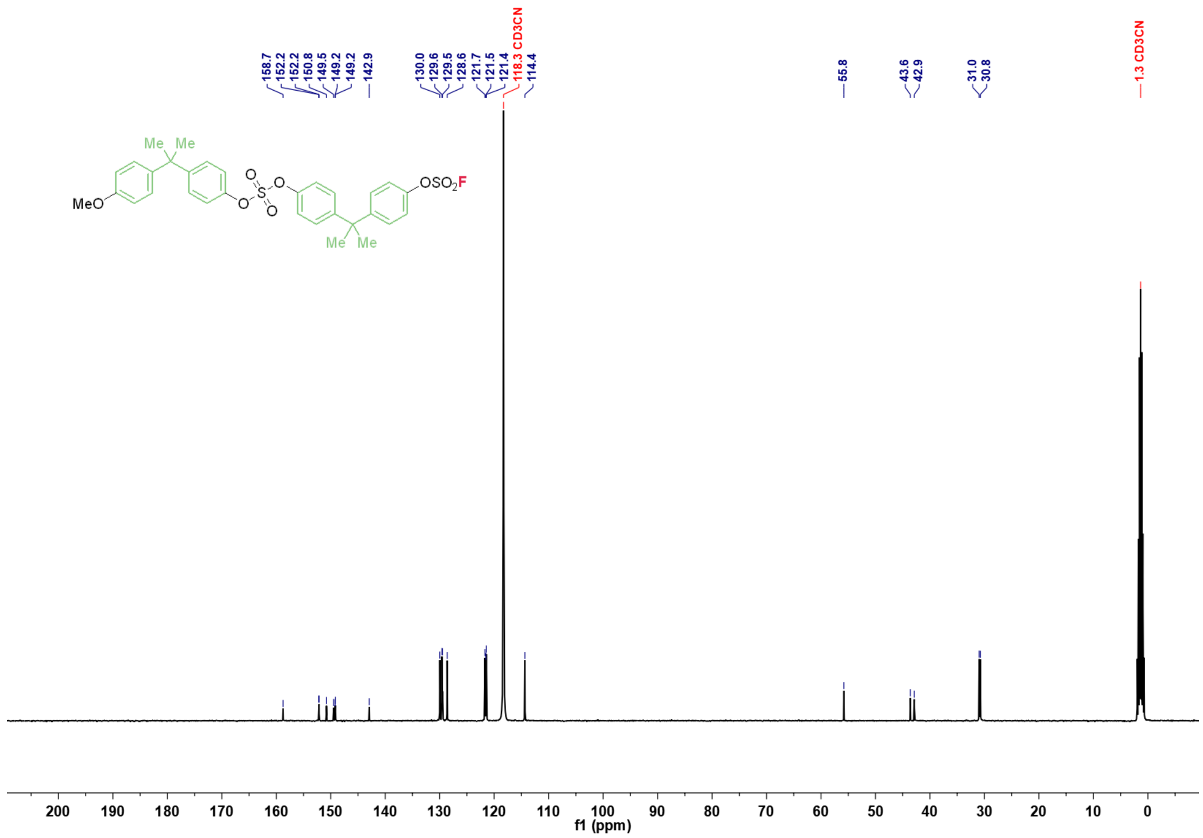

^{19}F NMR (376 MHz, CD_3CN) of **20**

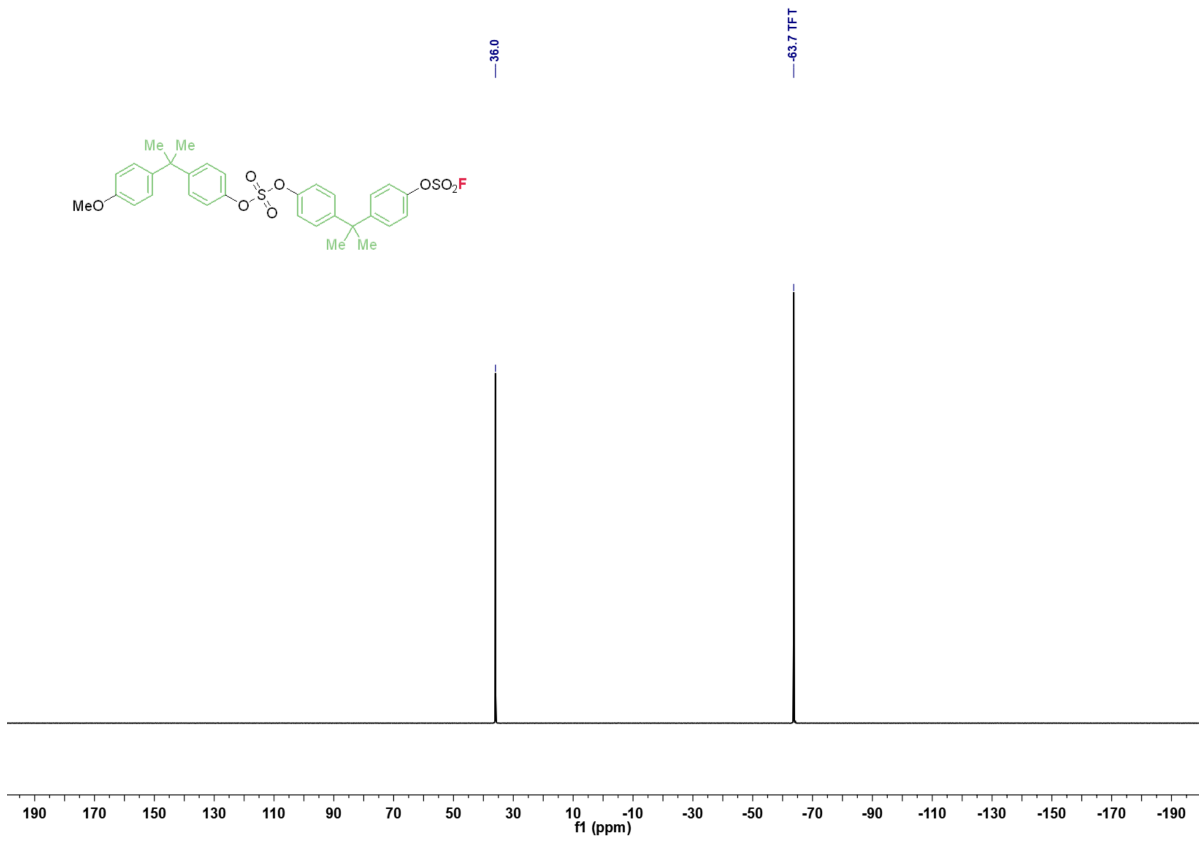

^{1}H NMR (400 MHz, CD_3CN) of **21**

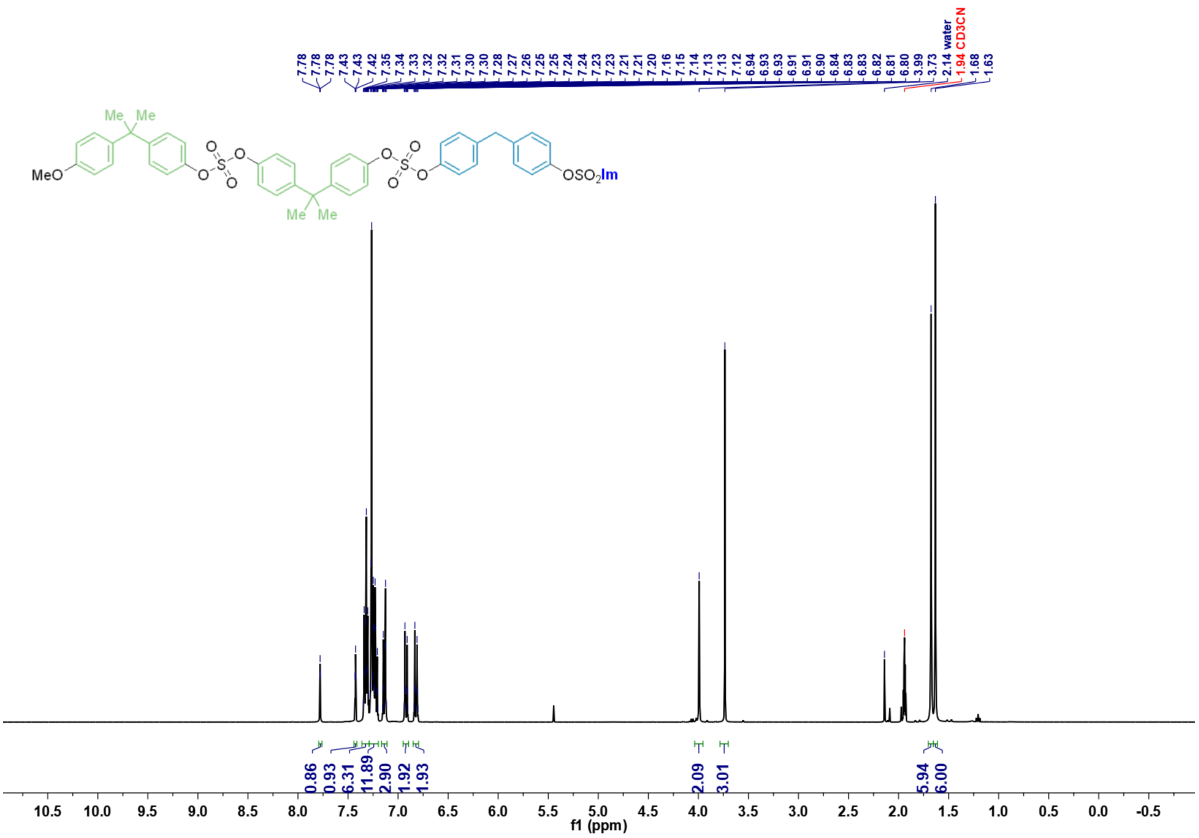

^{13}C NMR (101 MHz, CD_3CN) of **21**

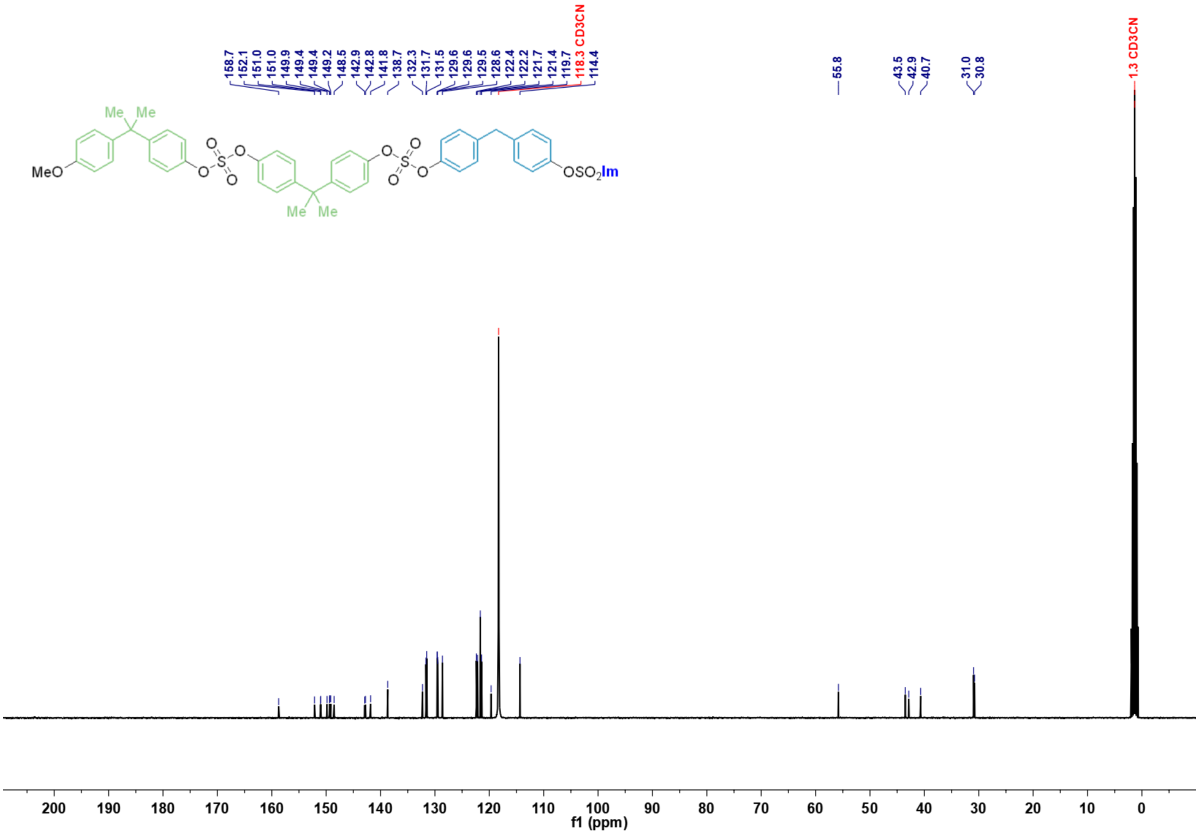

^{1}H NMR (400 MHz, CD_3CN) of **22**

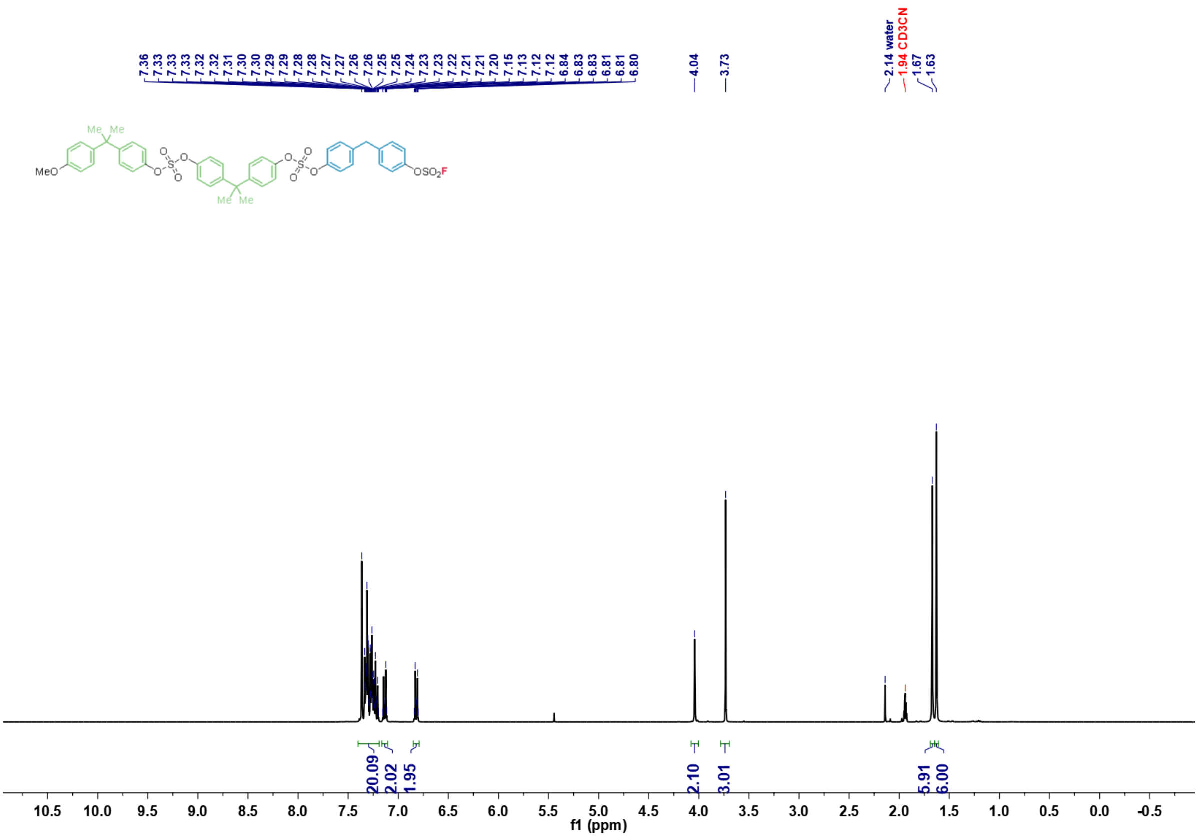

^{13}C NMR (101 MHz, CD_3CN) of **22**

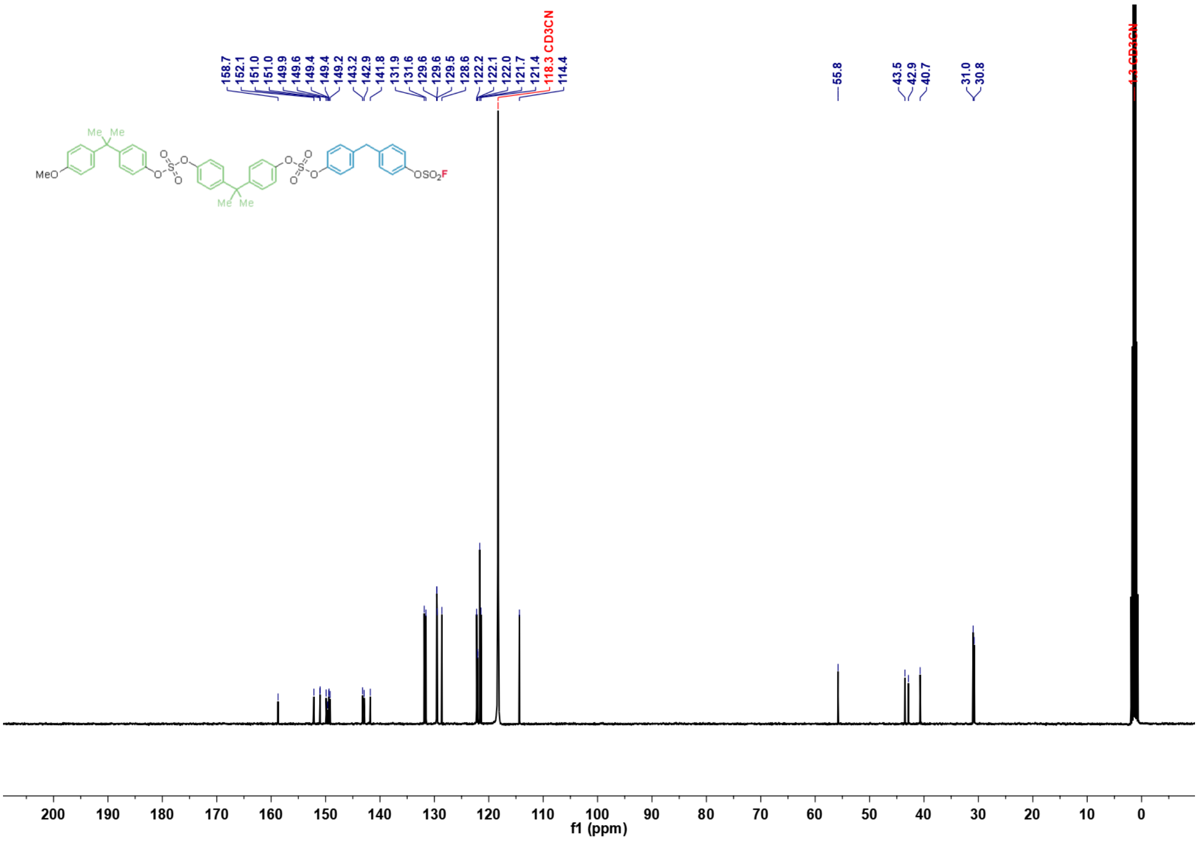

^{19}F NMR (376 MHz, CD_3CN) of **22**

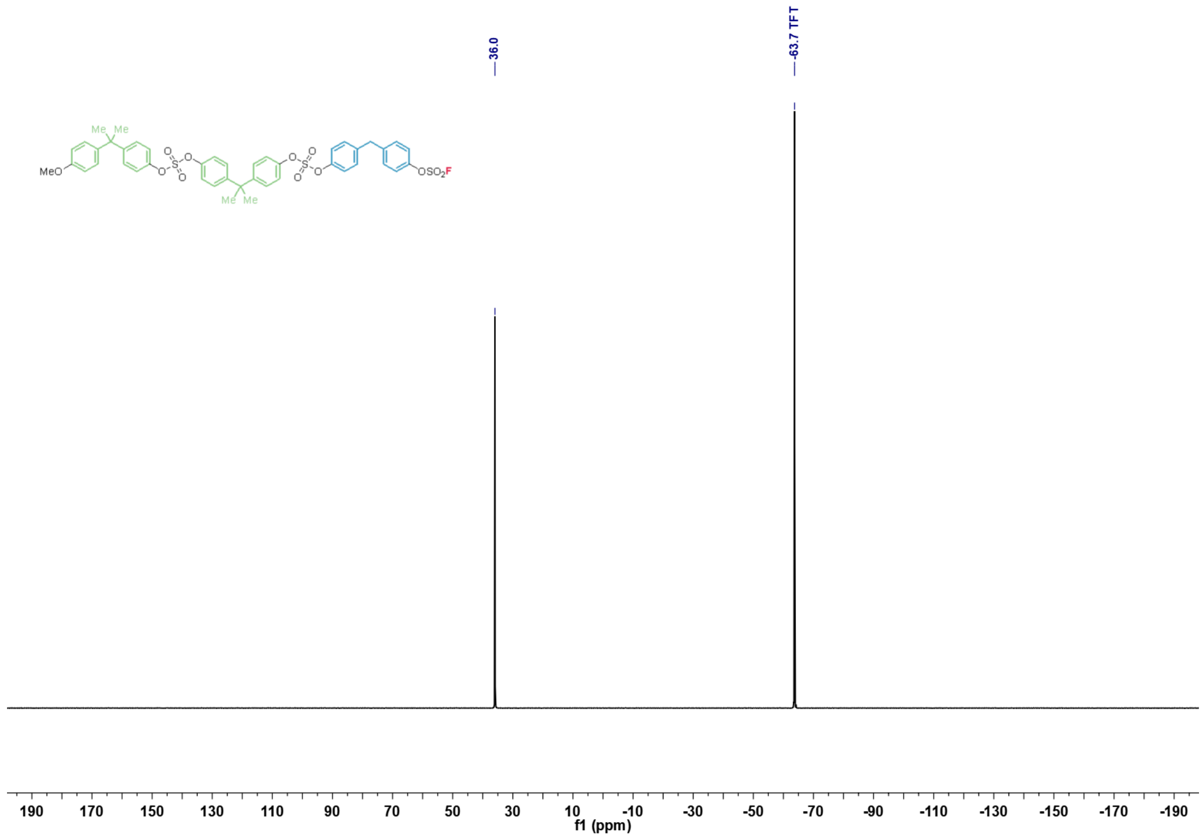

^{1}H NMR (400 MHz, CD_3CN) of **23**

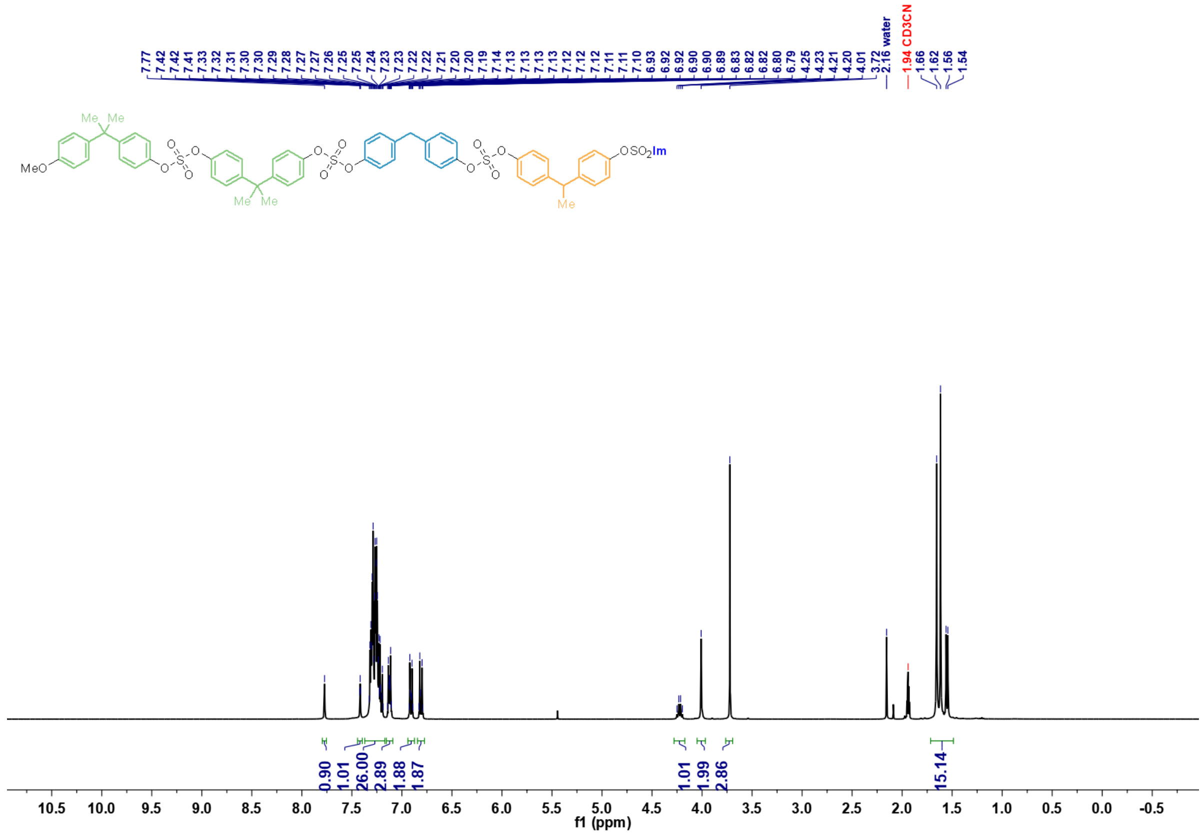

^{13}C NMR (101 MHz, CD_3CN) of **23**

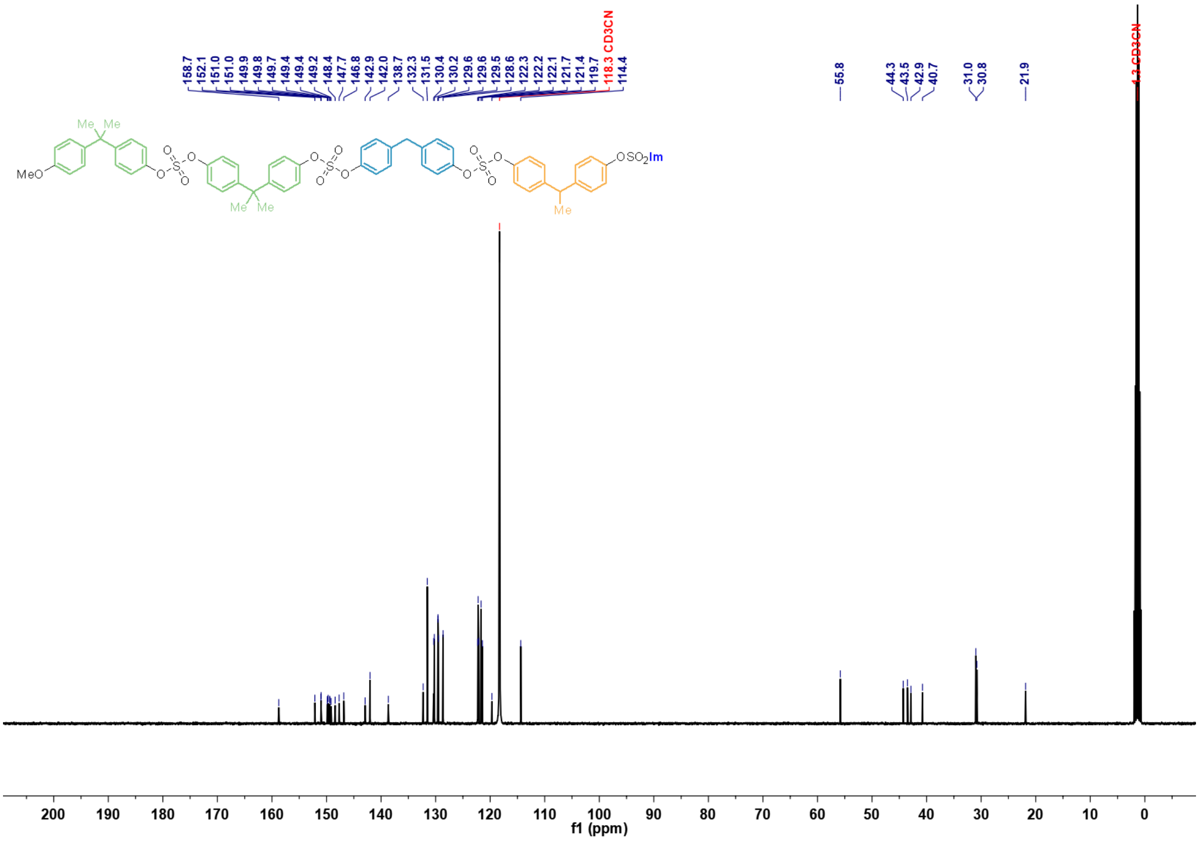

^{1}H NMR (400 MHz, CD_3CN) of **24**

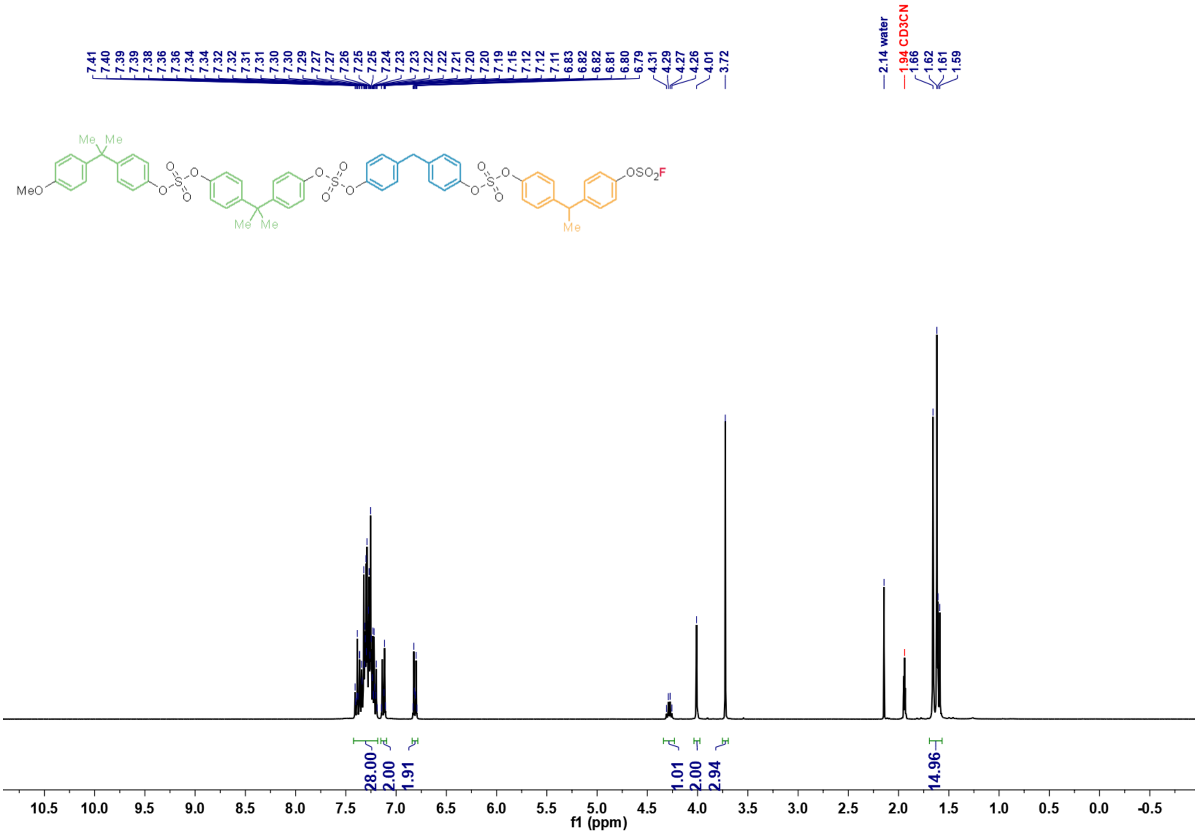

^{13}C NMR (101 MHz, CD_3CN) of **24**

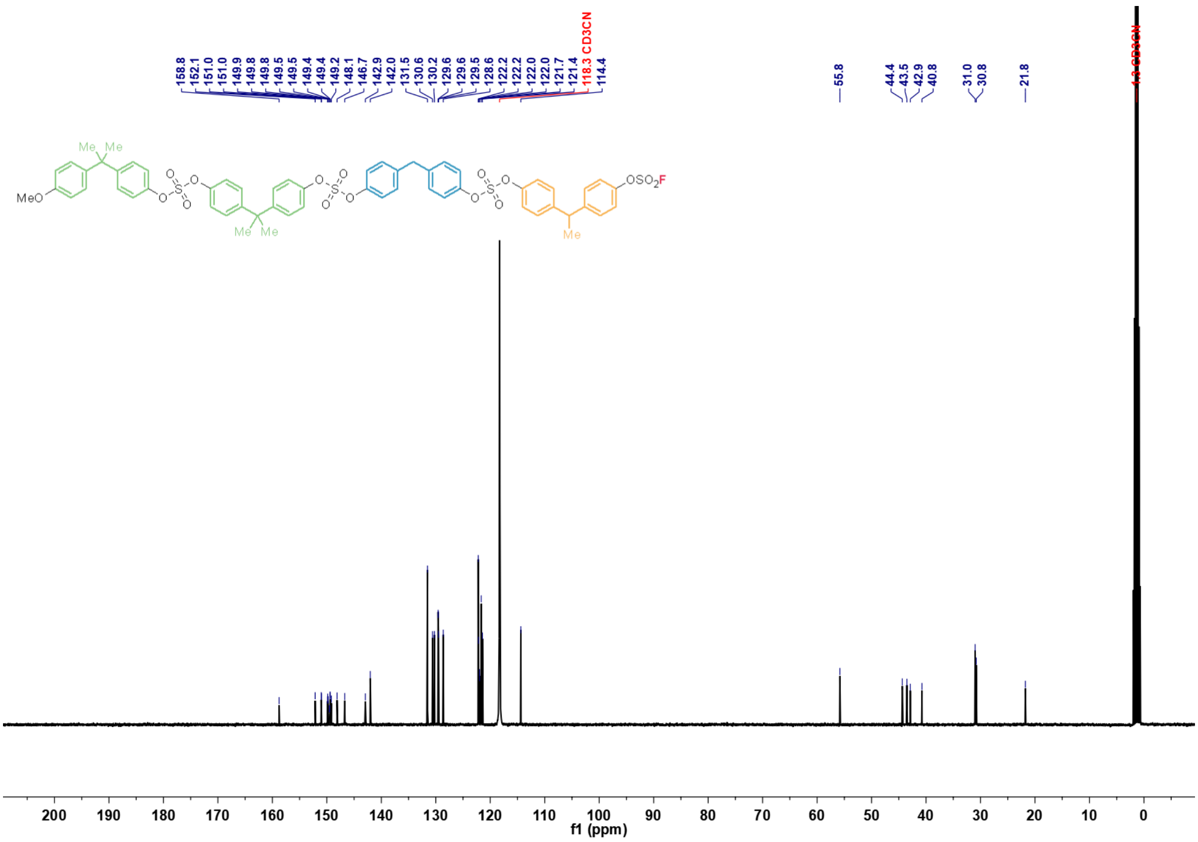

^{19}F NMR (376 MHz, CD_3CN) of **24**

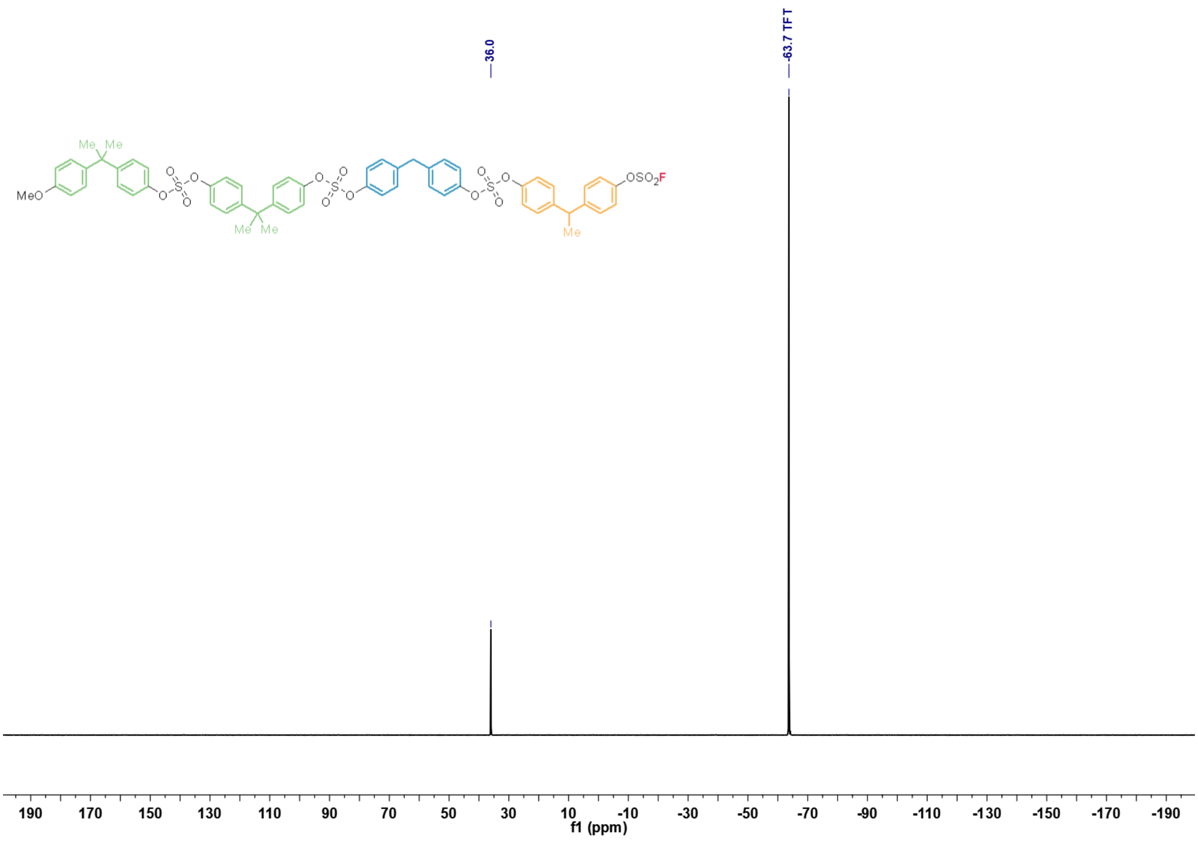

^{1}H NMR (400 MHz, CD_3CN) of **25**

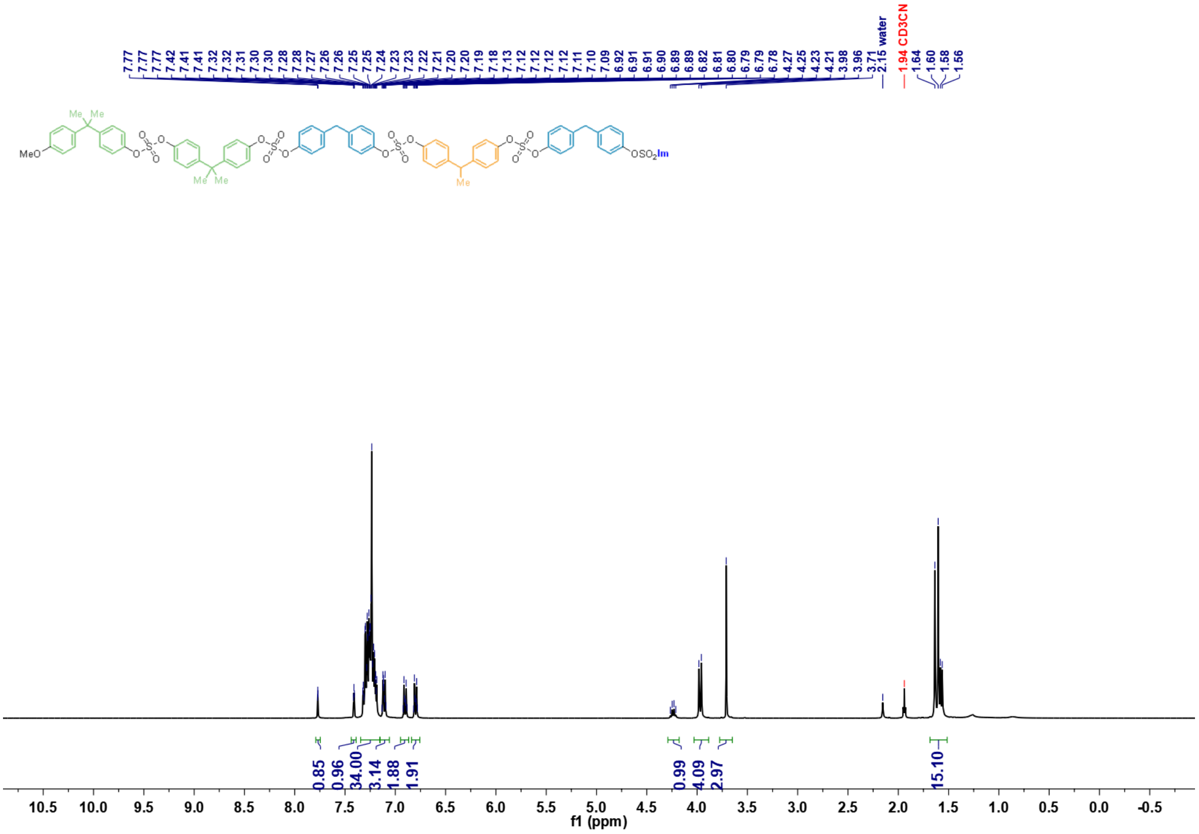

^{13}C NMR (101 MHz, CD_3CN) of **25**

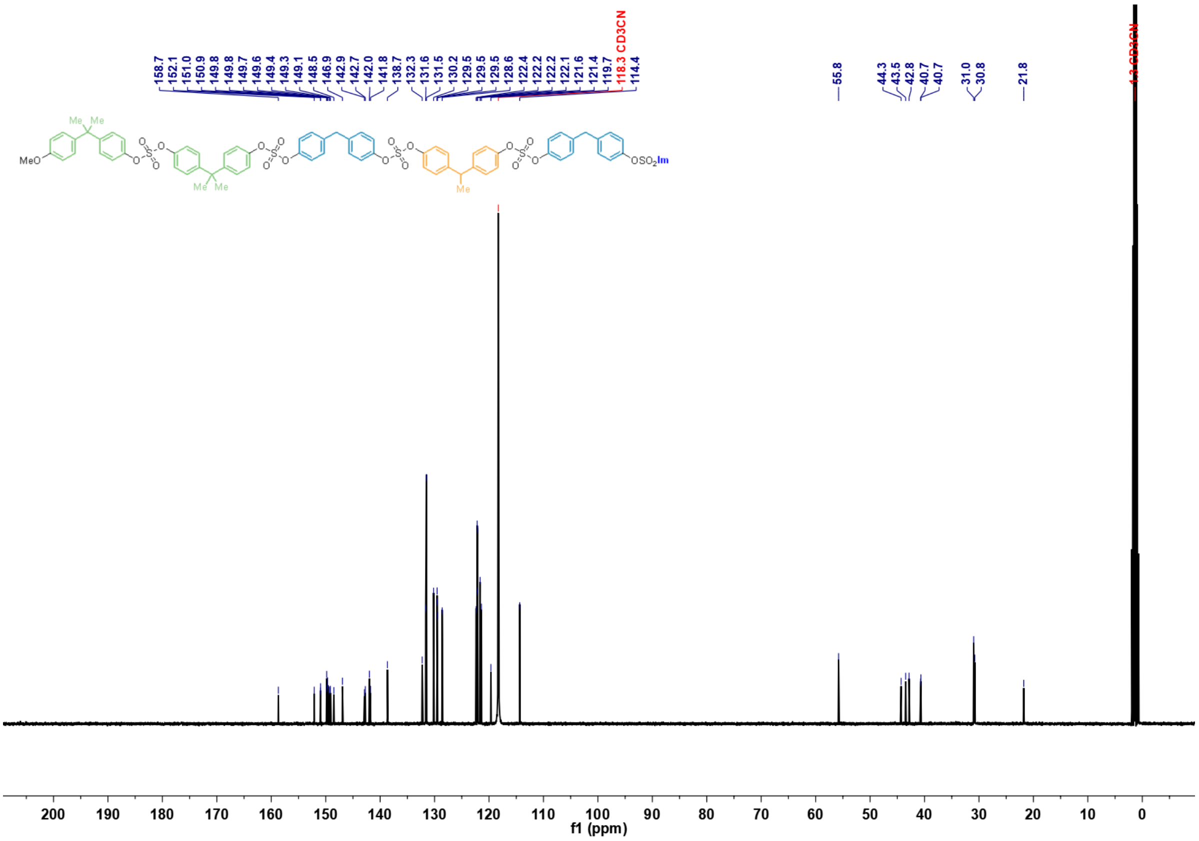

^{1}H NMR (400 MHz, DMSO-d_6) of **26**

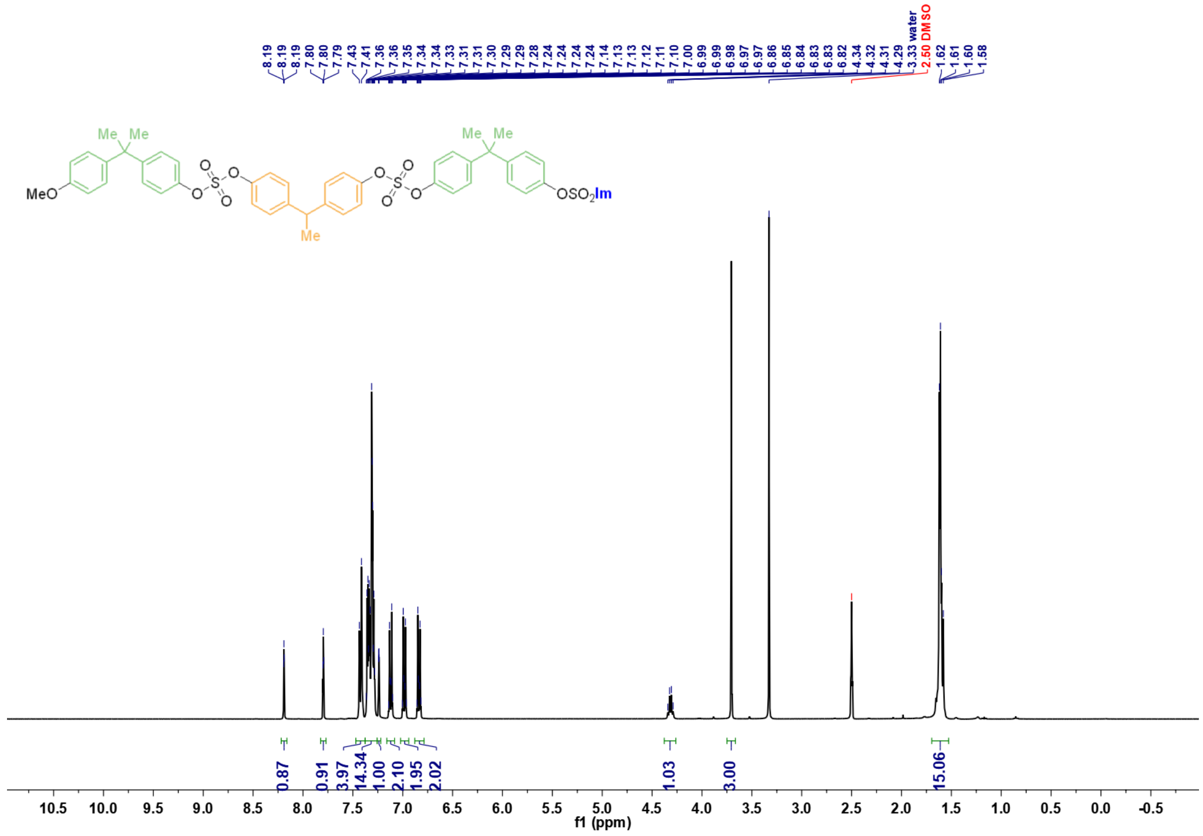

^{13}C NMR (101 MHz, DMSO-d_6) of **26**

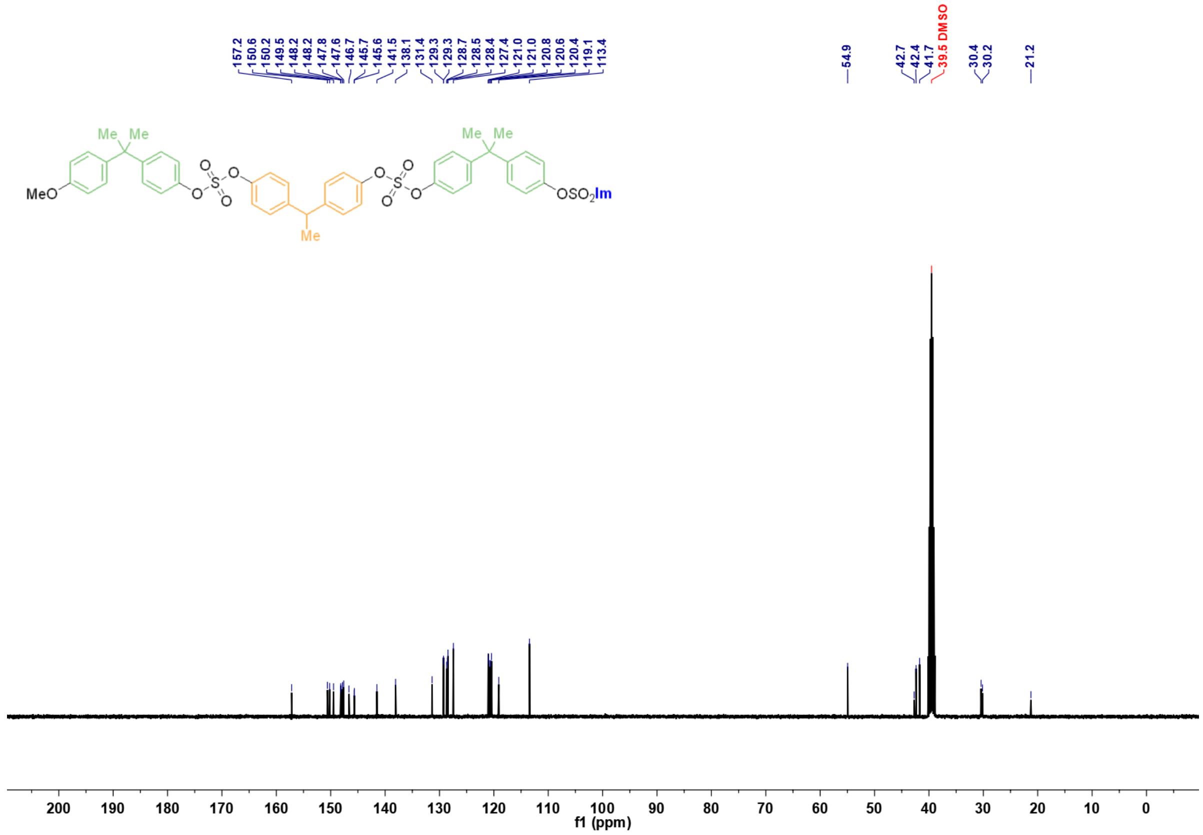

^{1}H NMR (400 MHz, DMSO-d_6) of **27**

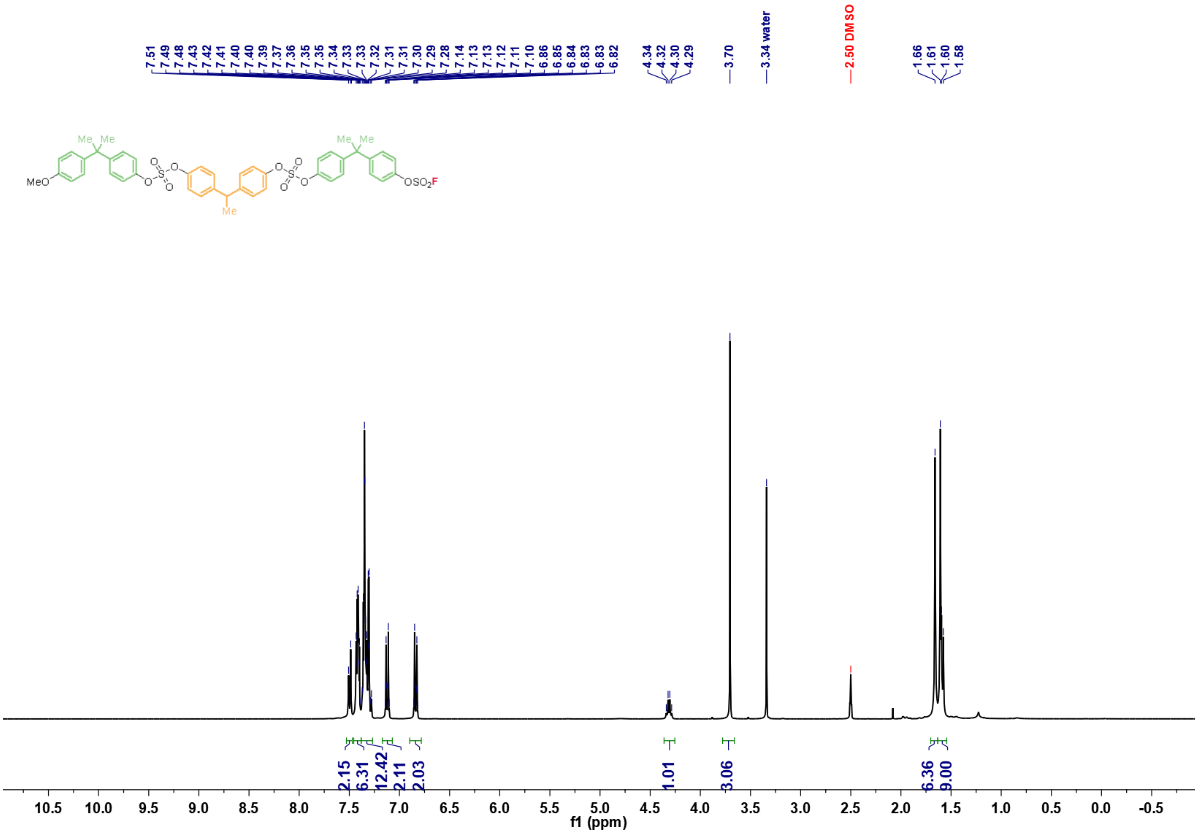

^{13}C NMR (101 MHz, DMSO-d_6) of **27**

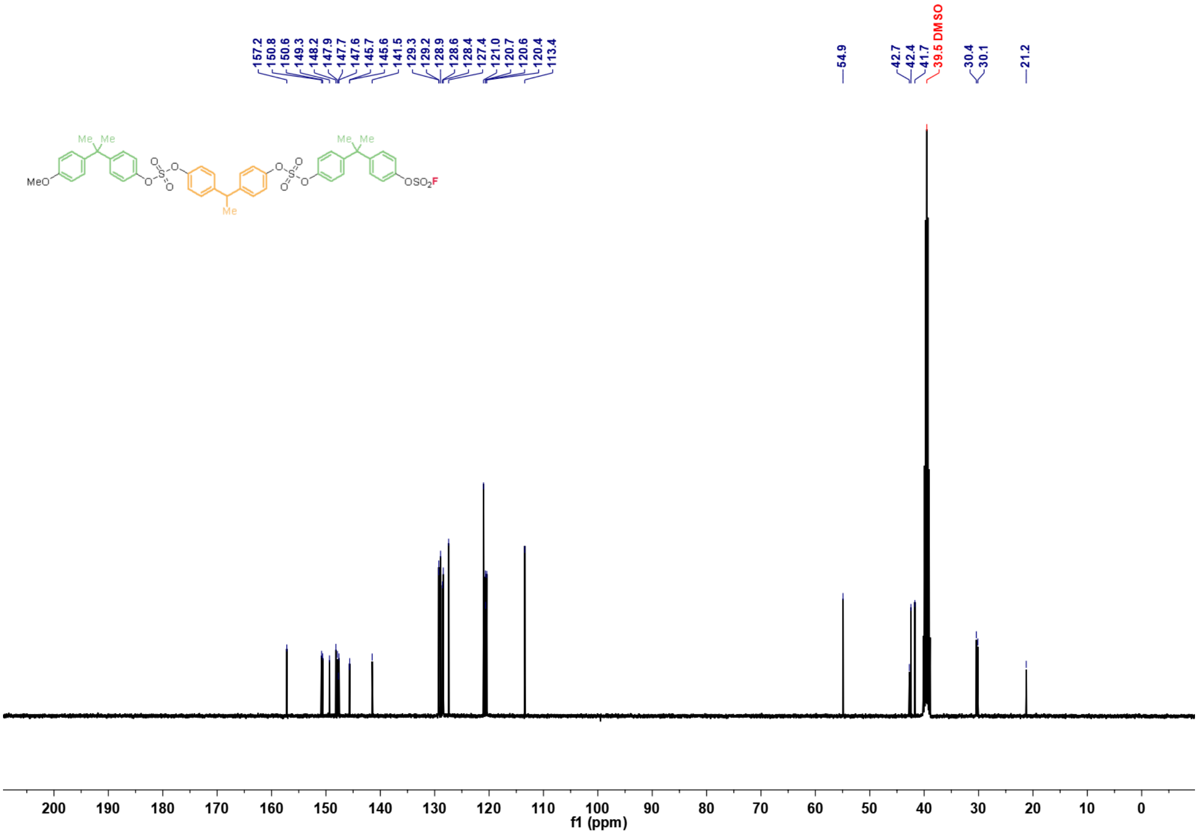

^{19}F NMR (376 MHz, DMSO-d_6) of **27**

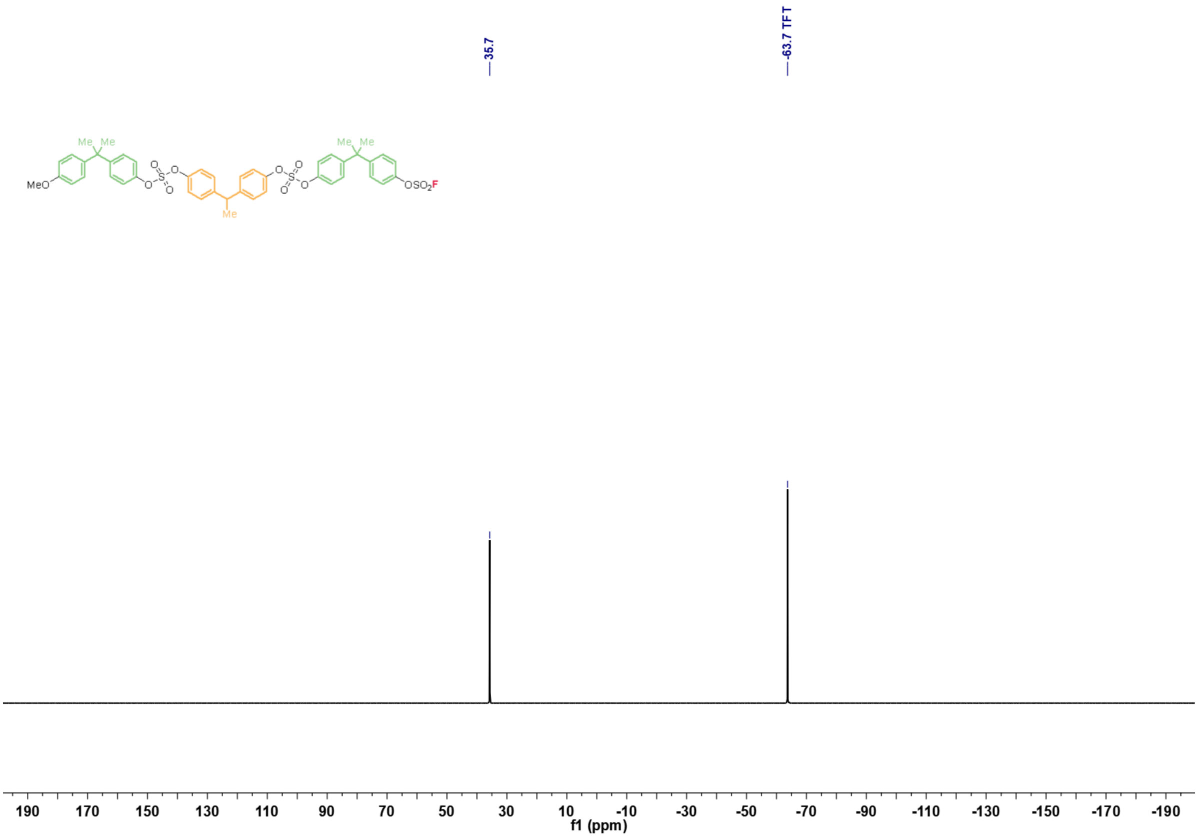

^{1}H NMR (400 MHz, CD_3CN) of **28**

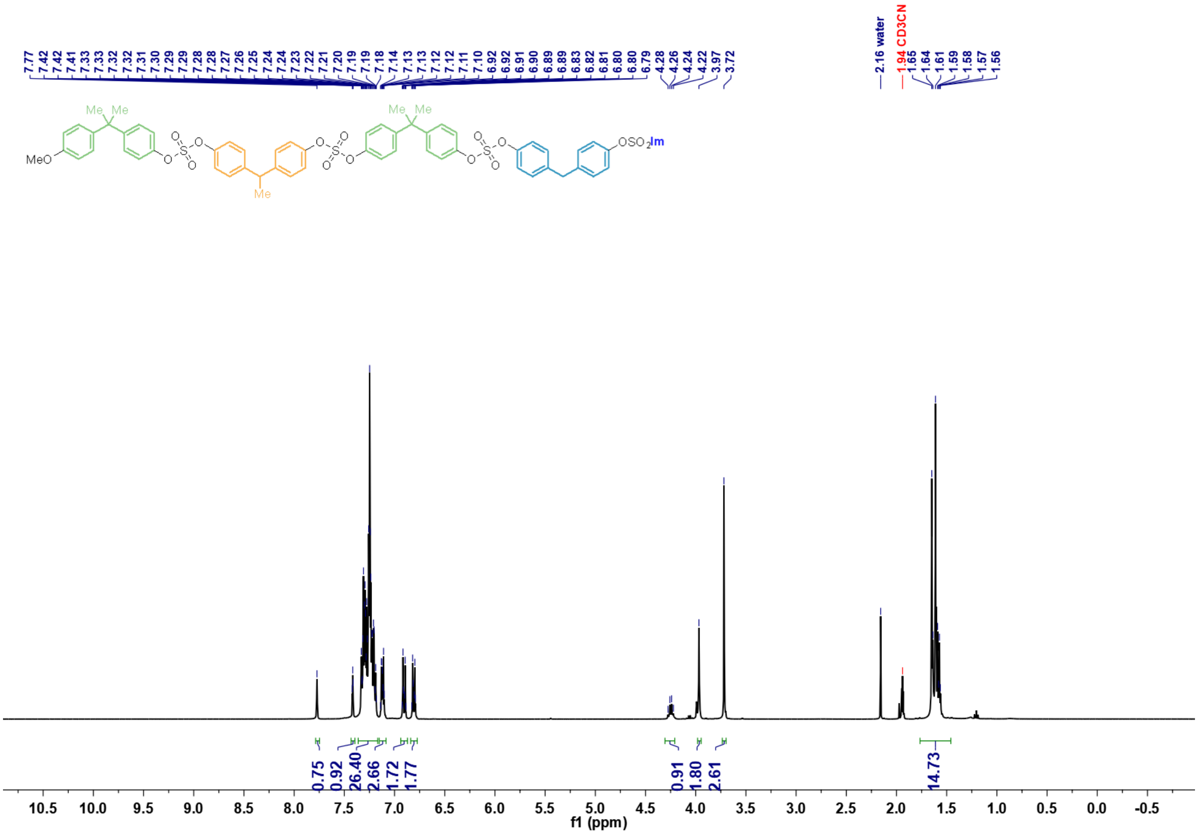

^{13}C NMR (101 MHz, CD_3CN) of **28**

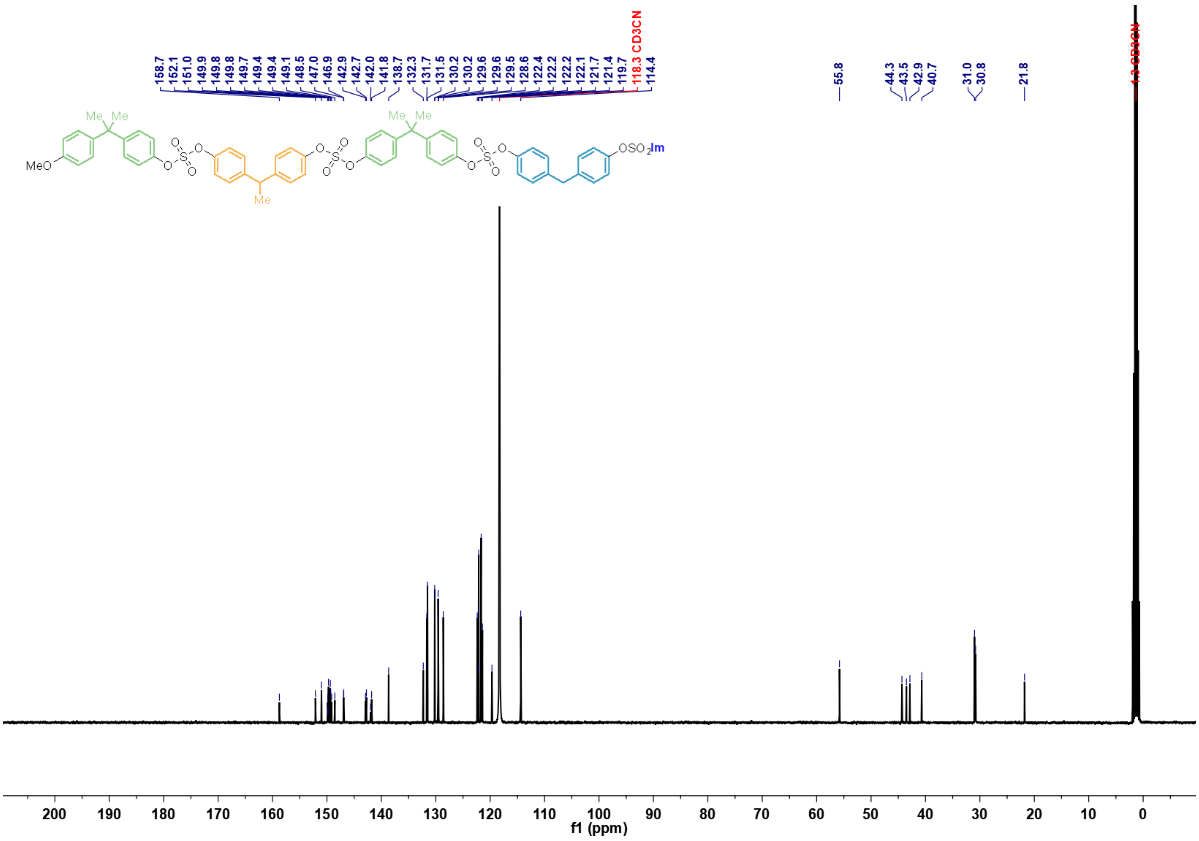

^{1}H NMR (400 MHz, CD_3CN) of **29**

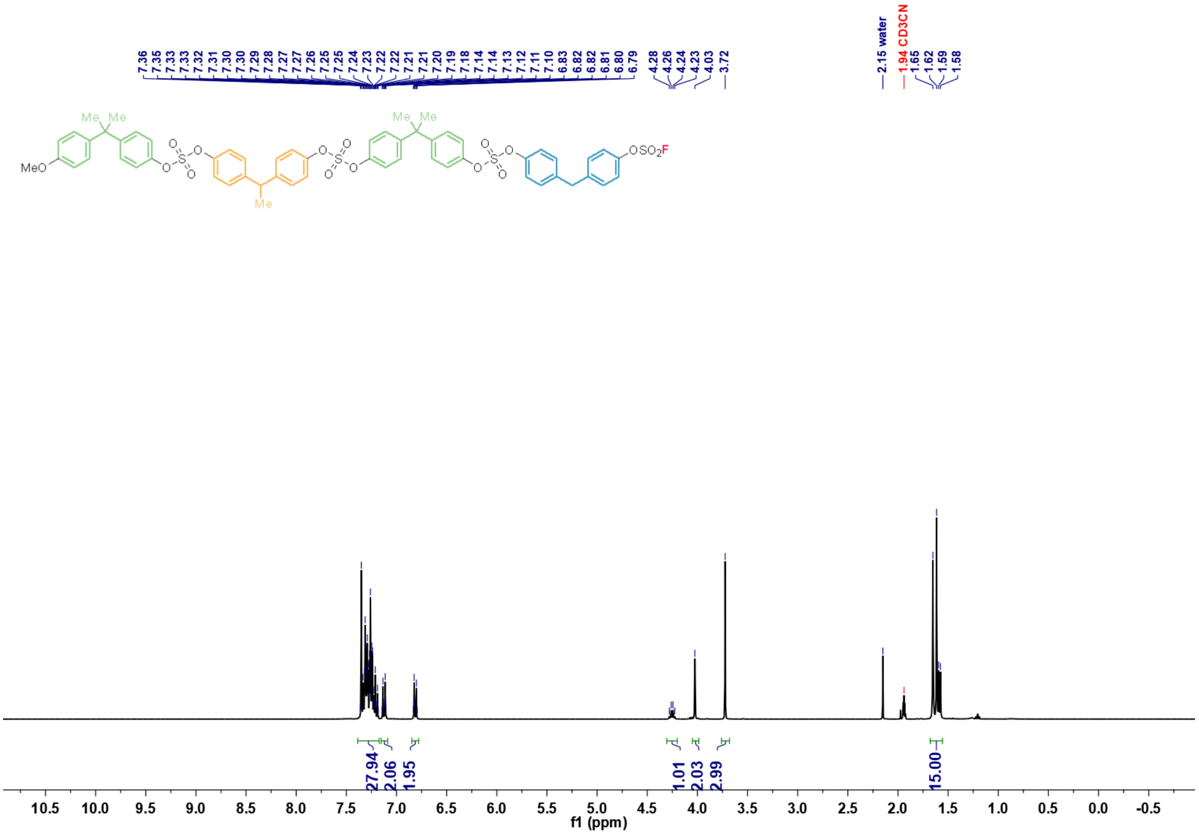

^{13}C NMR (101 MHz, CD_3CN) of **29**

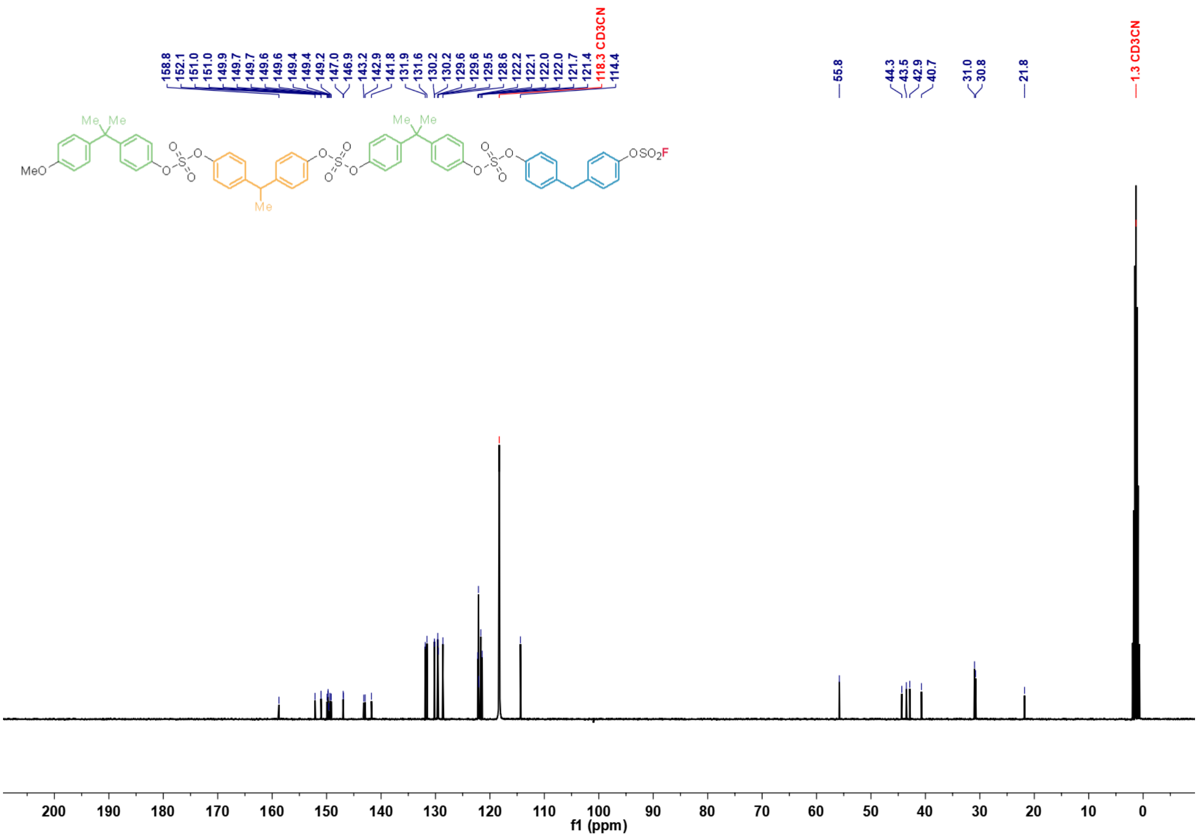

^{19}F NMR (376 MHz, CD_3CN) of **29**

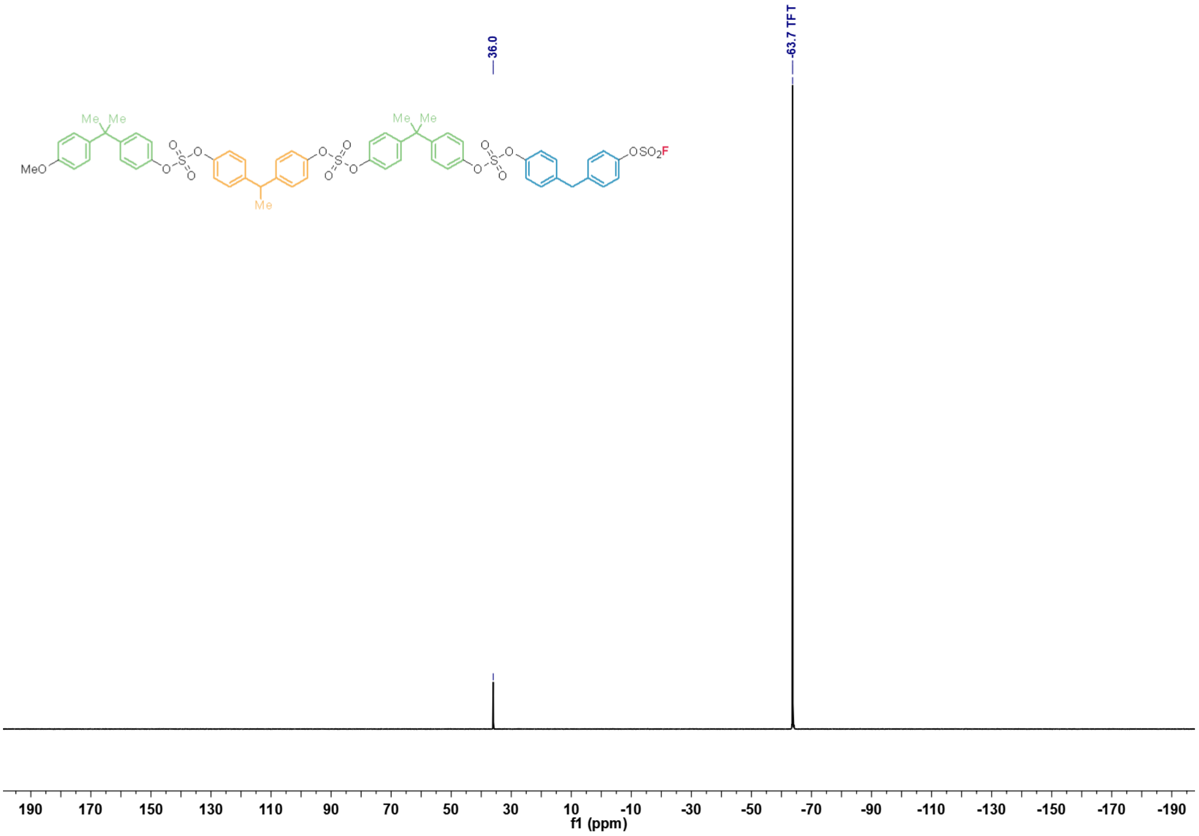

^{1}H NMR (400 MHz, CD_3CN) of **30**

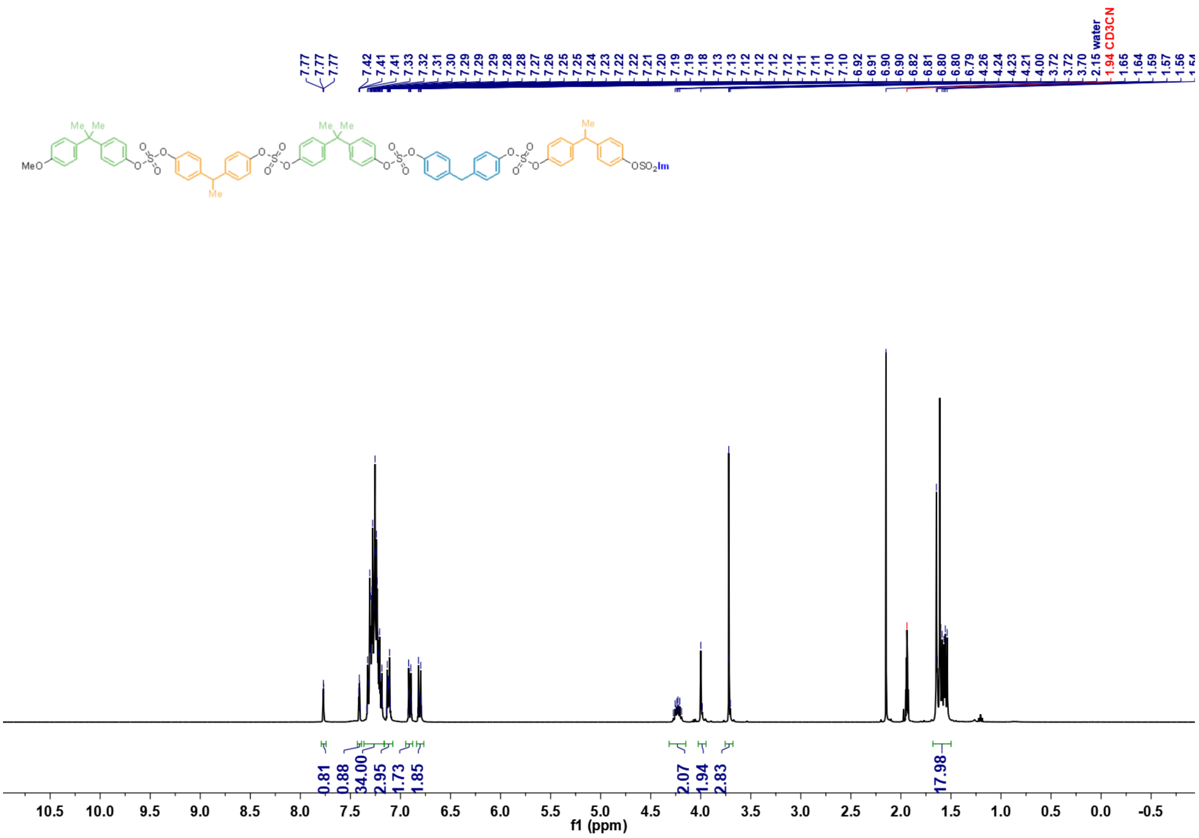

^{13}C NMR (101 MHz, CD_3CN) of **30**

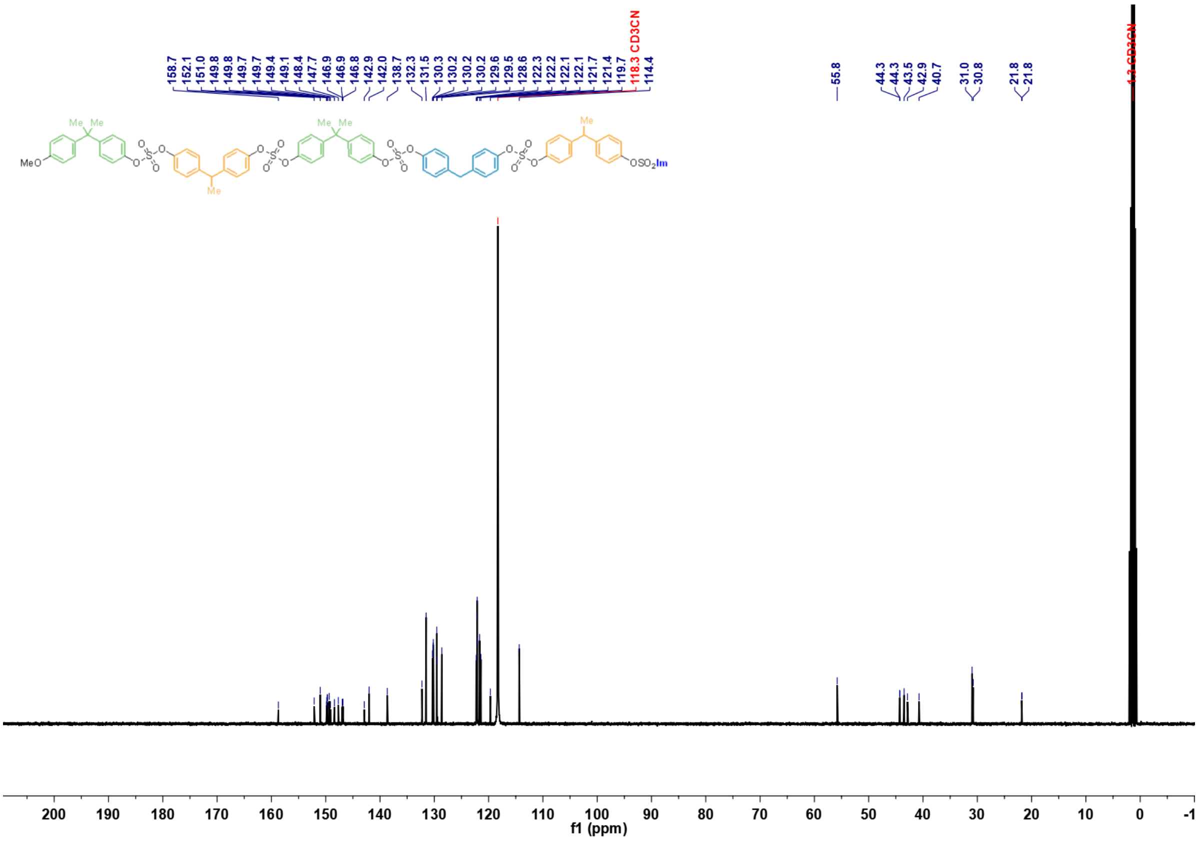

^{1}H NMR (400 MHz, CD_3CN) of **31**

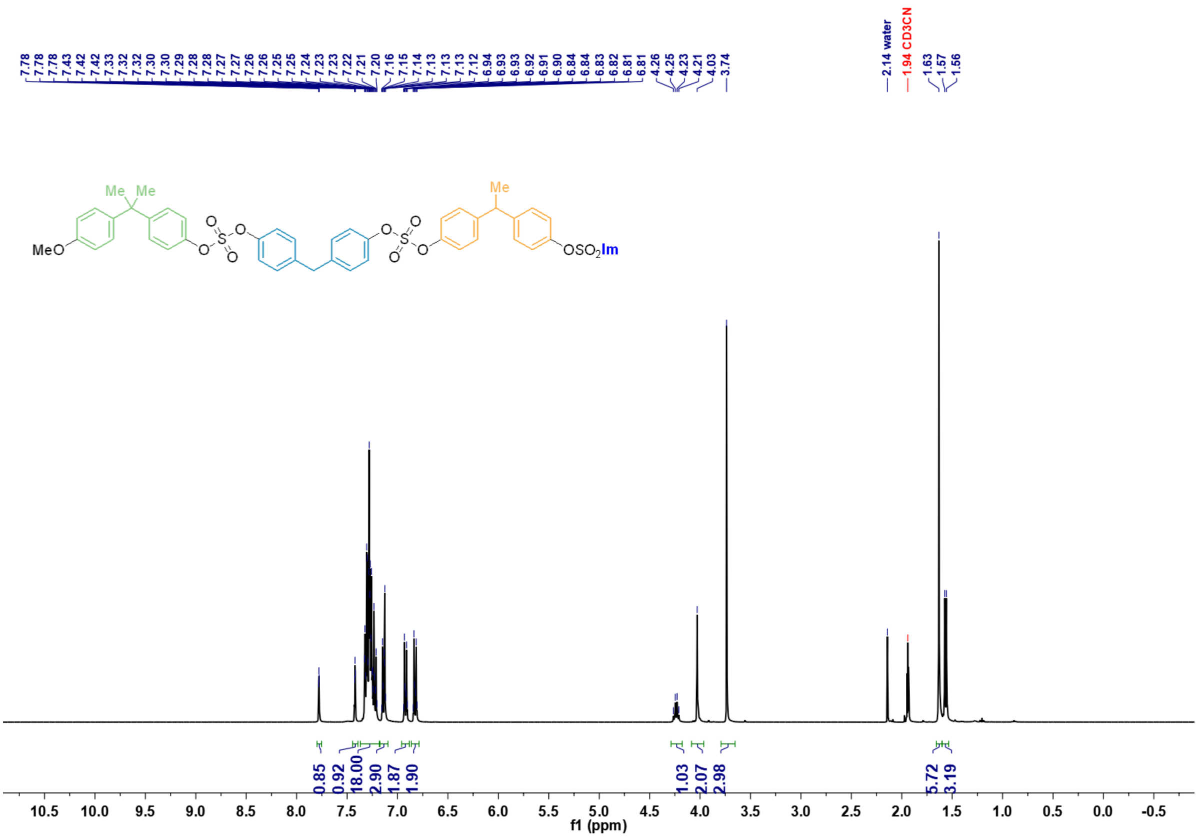

^{13}C NMR (101 MHz, CD_3CN) of **31**

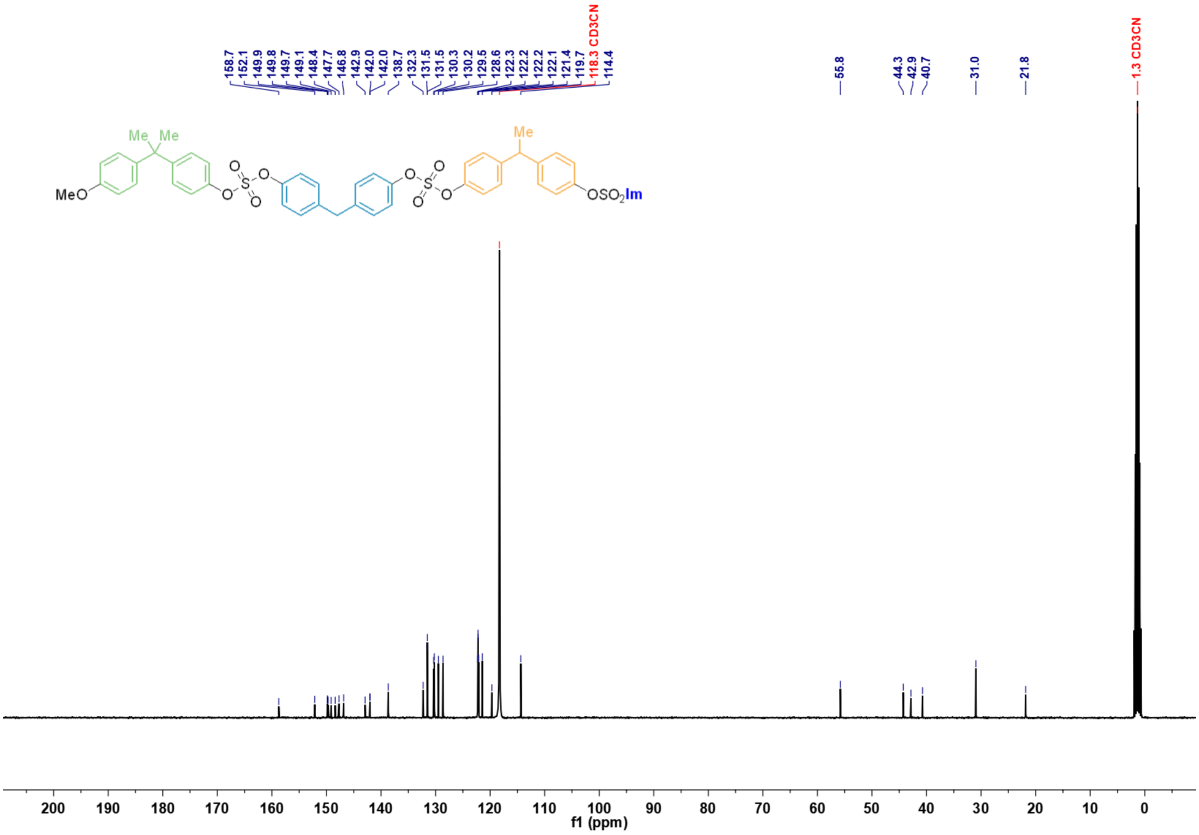

^{1}H NMR (400 MHz, CD_3CN) of **32**

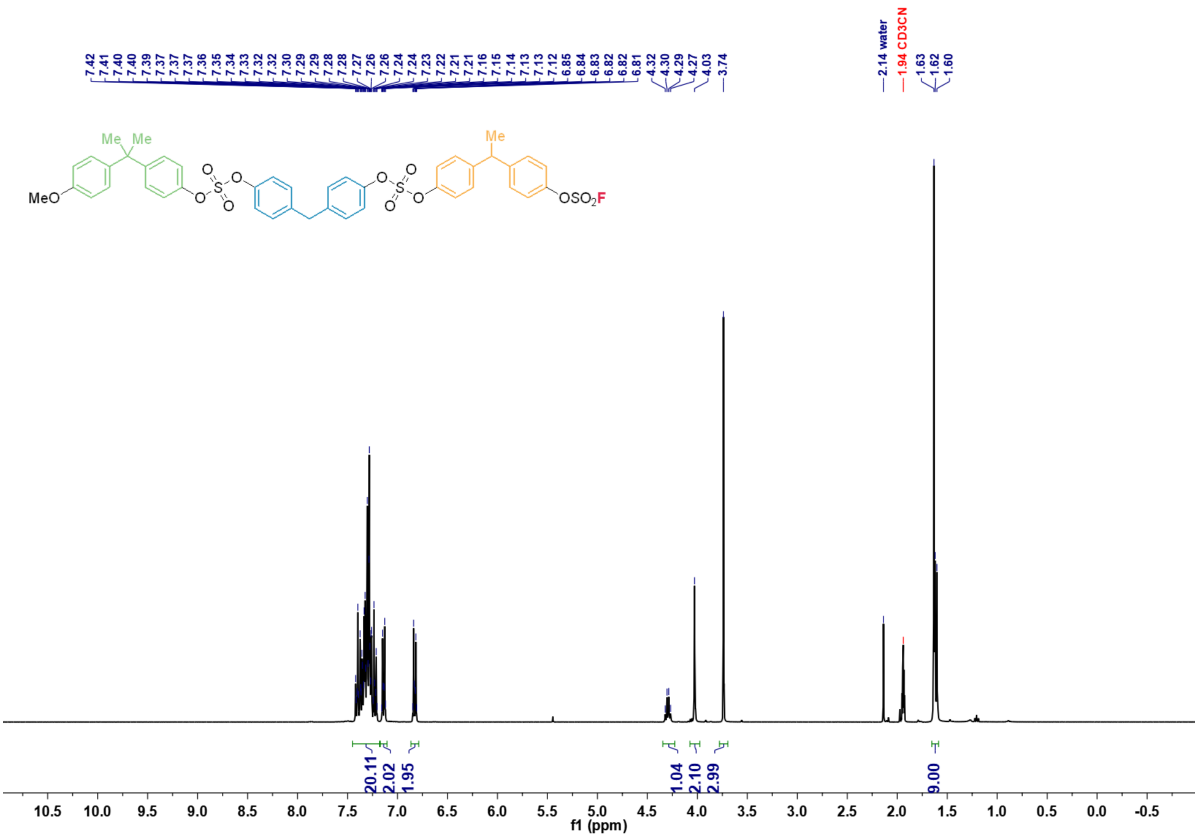

^{13}C NMR (101 MHz, CD_3CN) of **32**

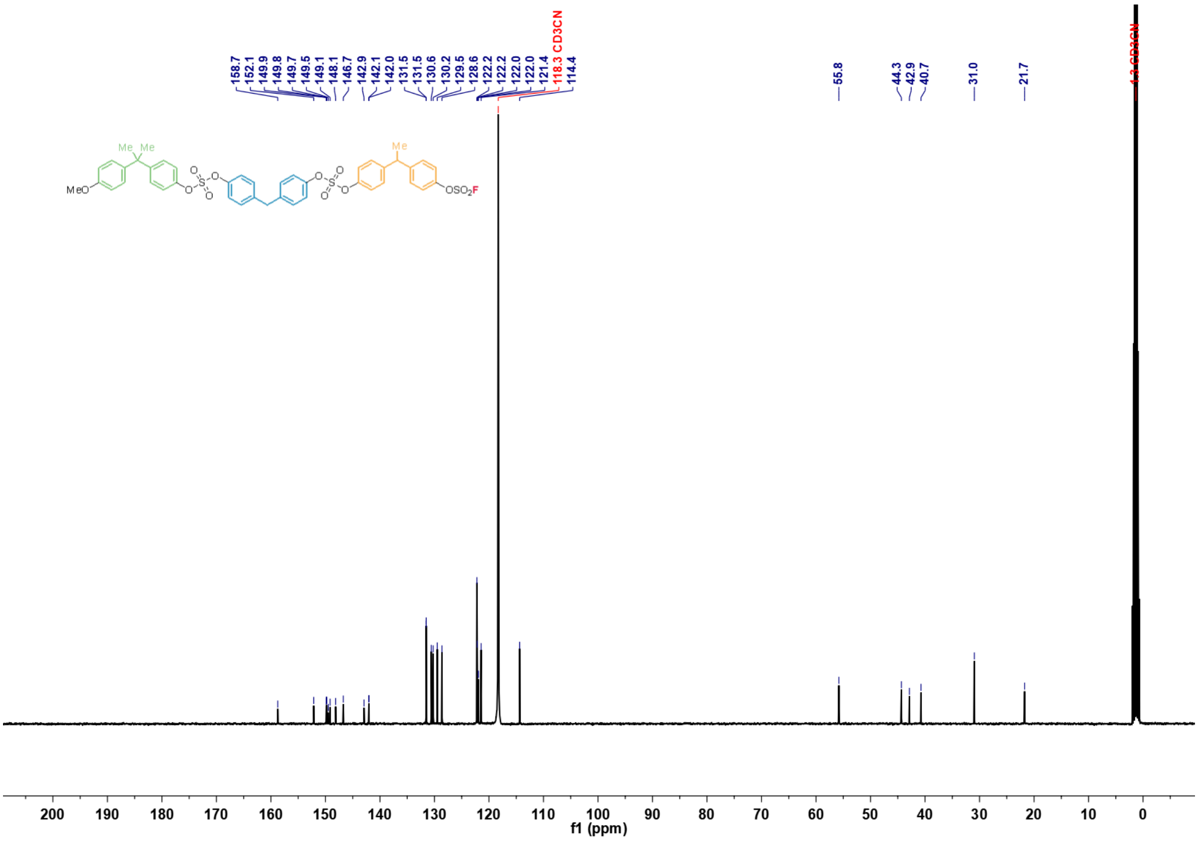

^{19}F NMR (376 MHz, CD_3CN) of **32**

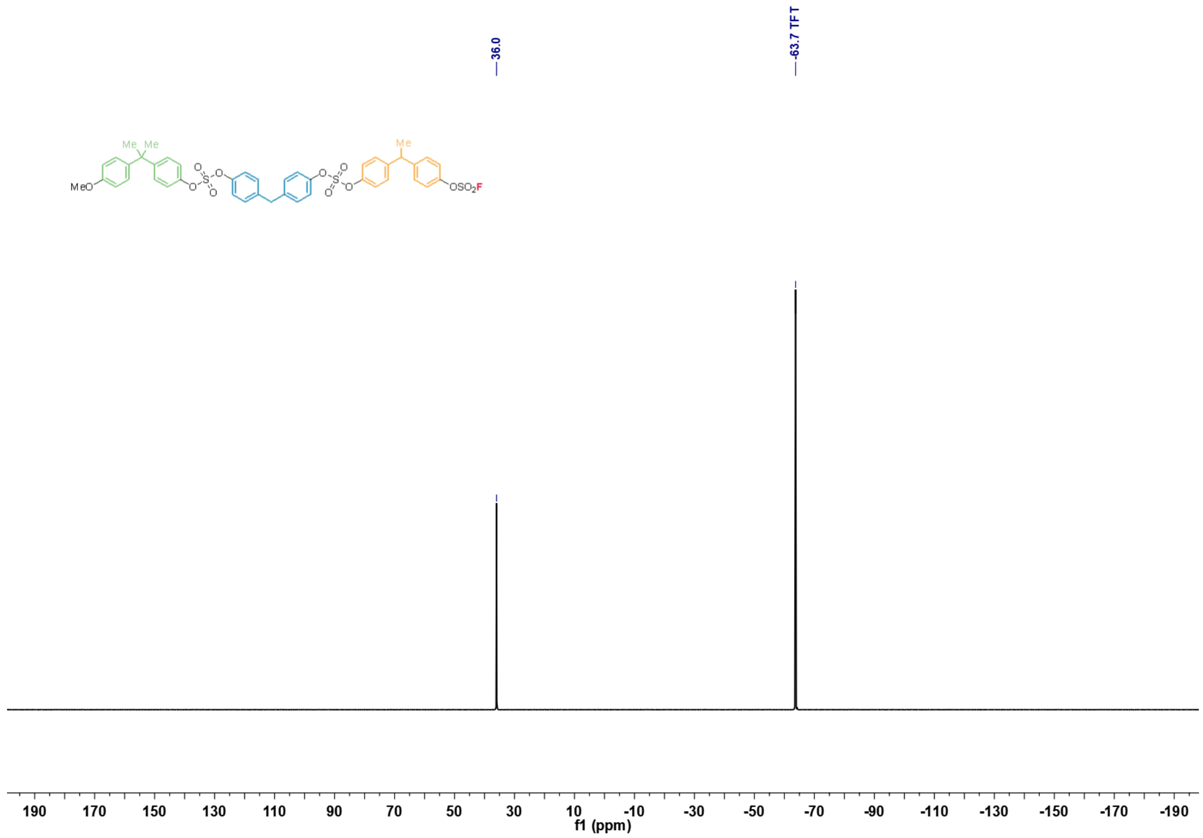

^{1}H NMR (400 MHz, CD_3CN) of **33**

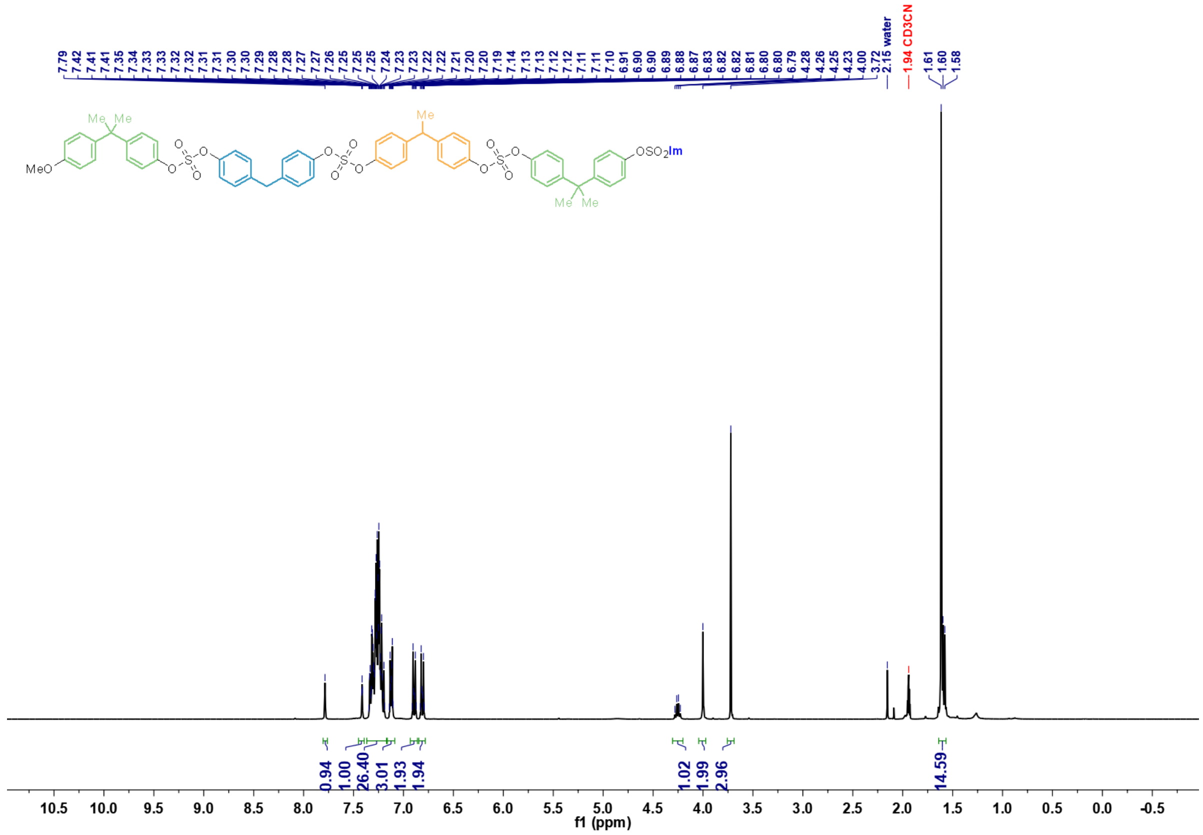

^{13}C NMR (101 MHz, CD_3CN) of **33**

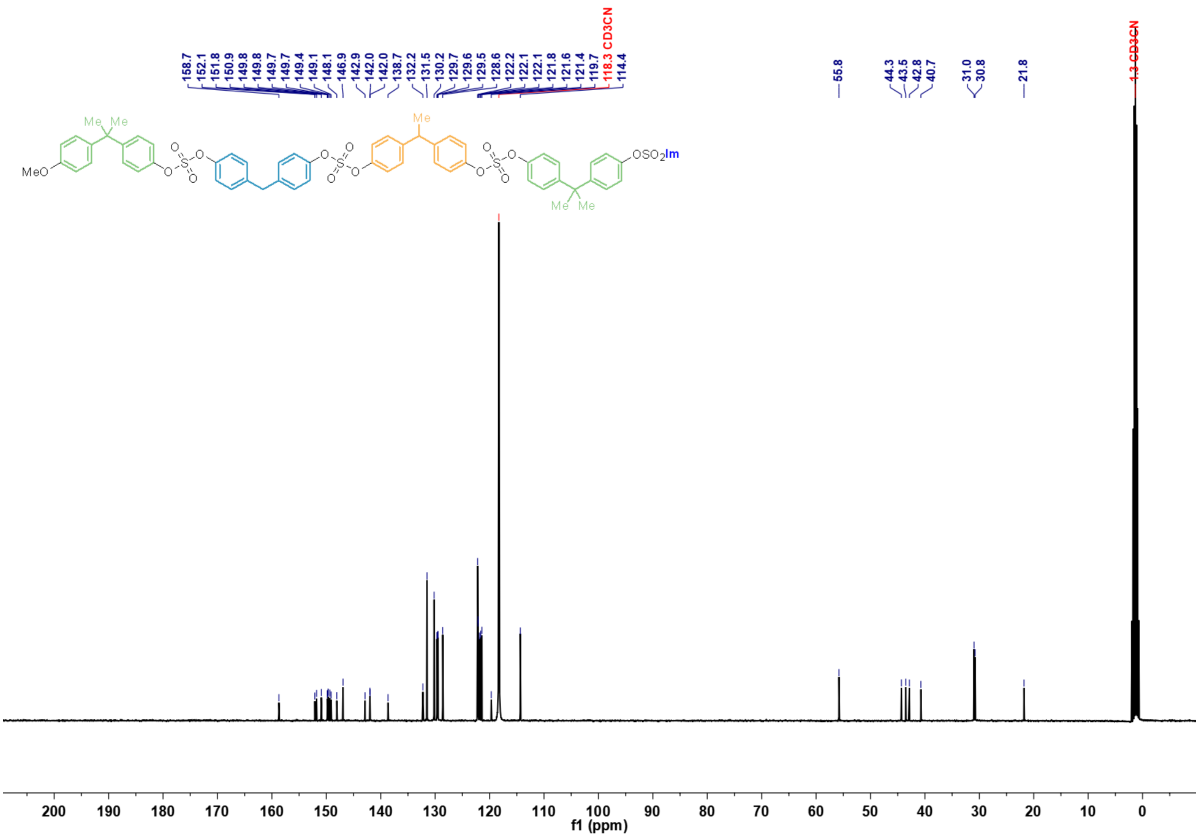

^{1}H NMR (400 MHz, CD_3CN) of **34**

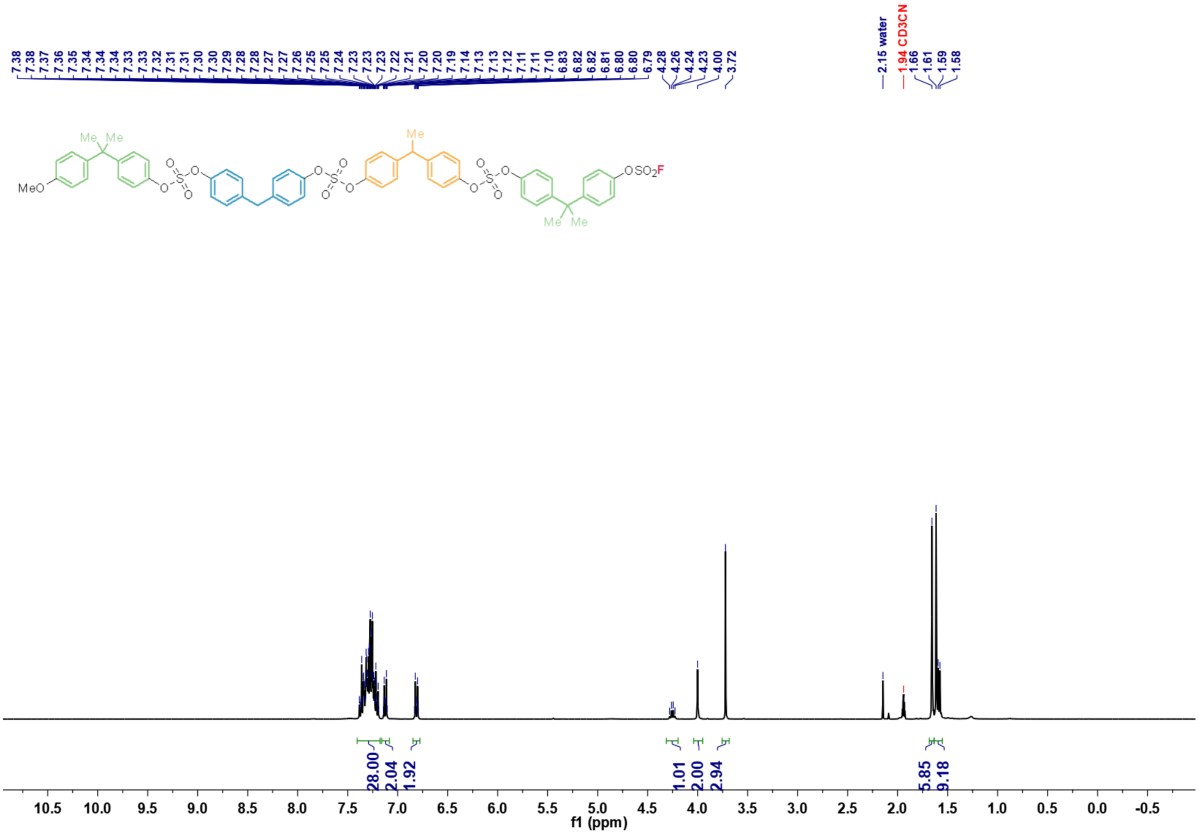

^{13}C NMR (101 MHz, CD_3CN) of **34**

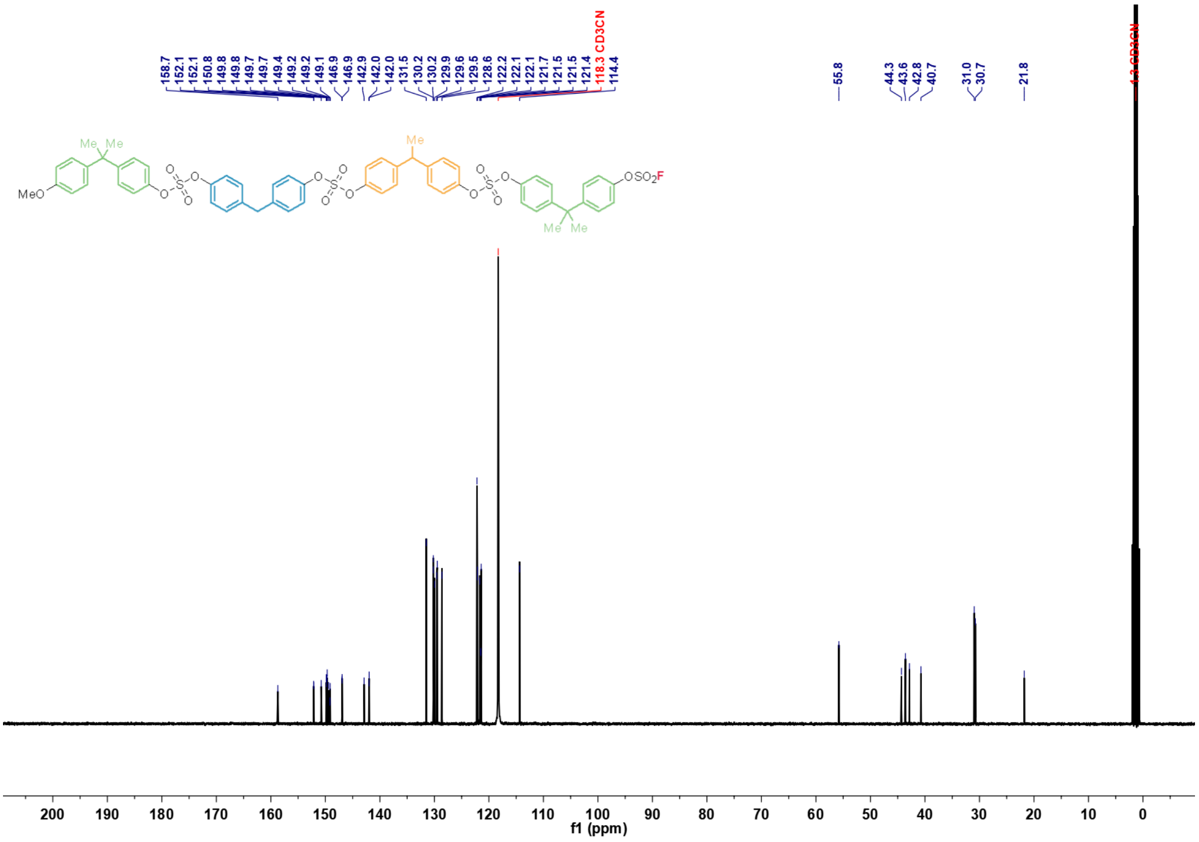

^{19}F NMR (376 MHz, CD_3CN) of **34**

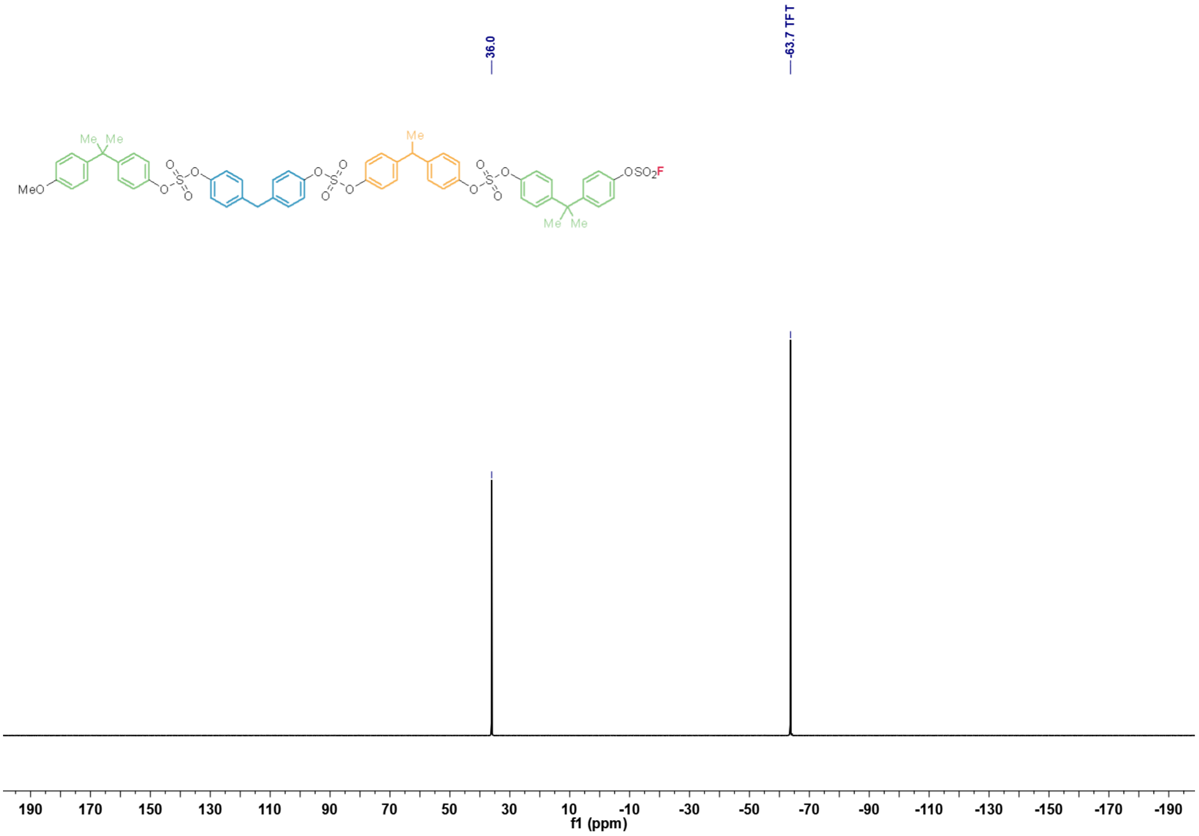

^{1}H NMR (400 MHz, CD_3CN) of **35**

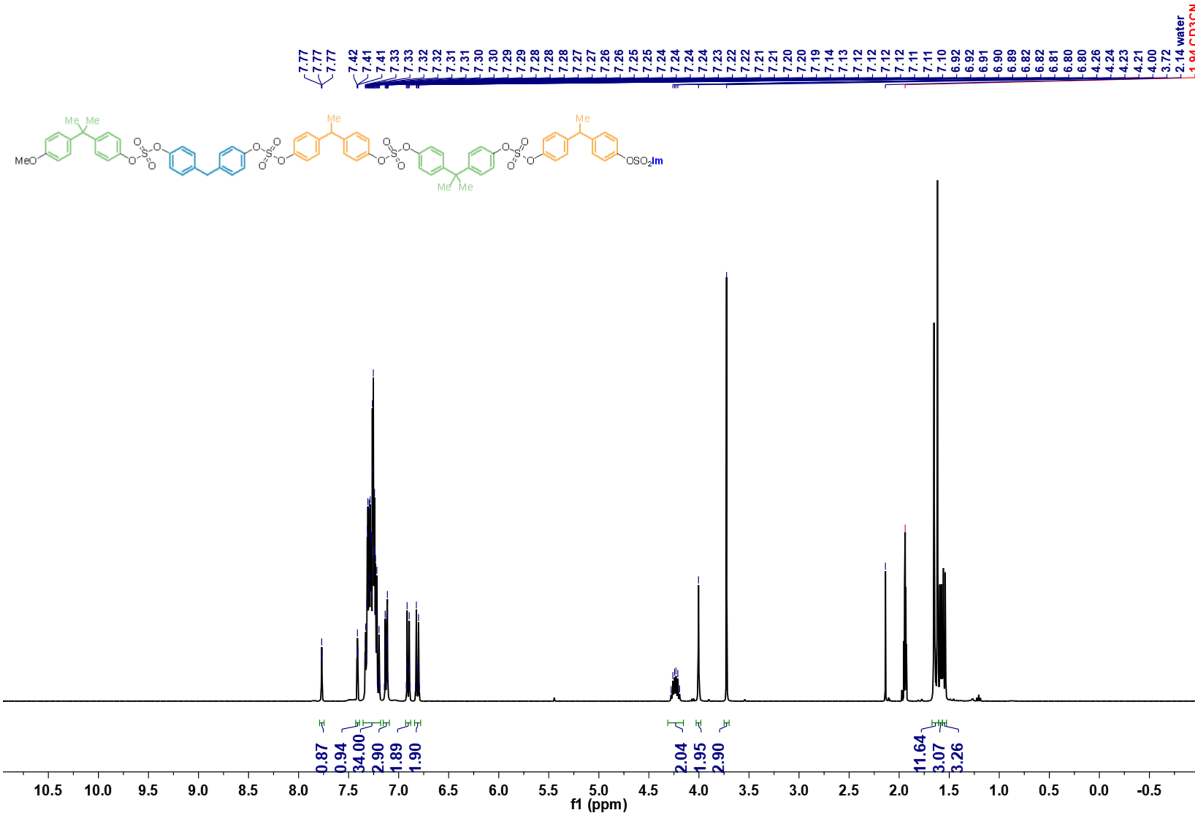

^{13}C NMR (101 MHz, CD_3CN) of **35**

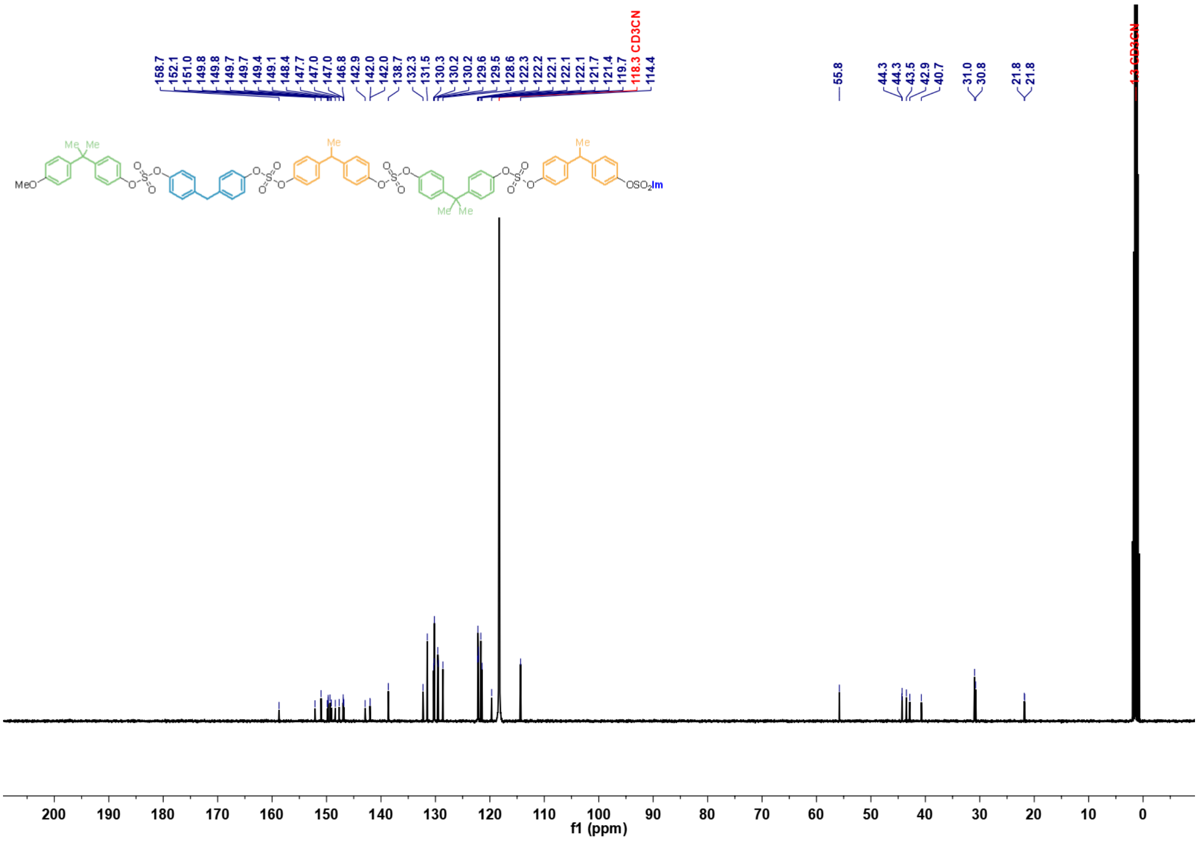

^{1}H NMR (400 MHz, $CDCl_3$) of **36**

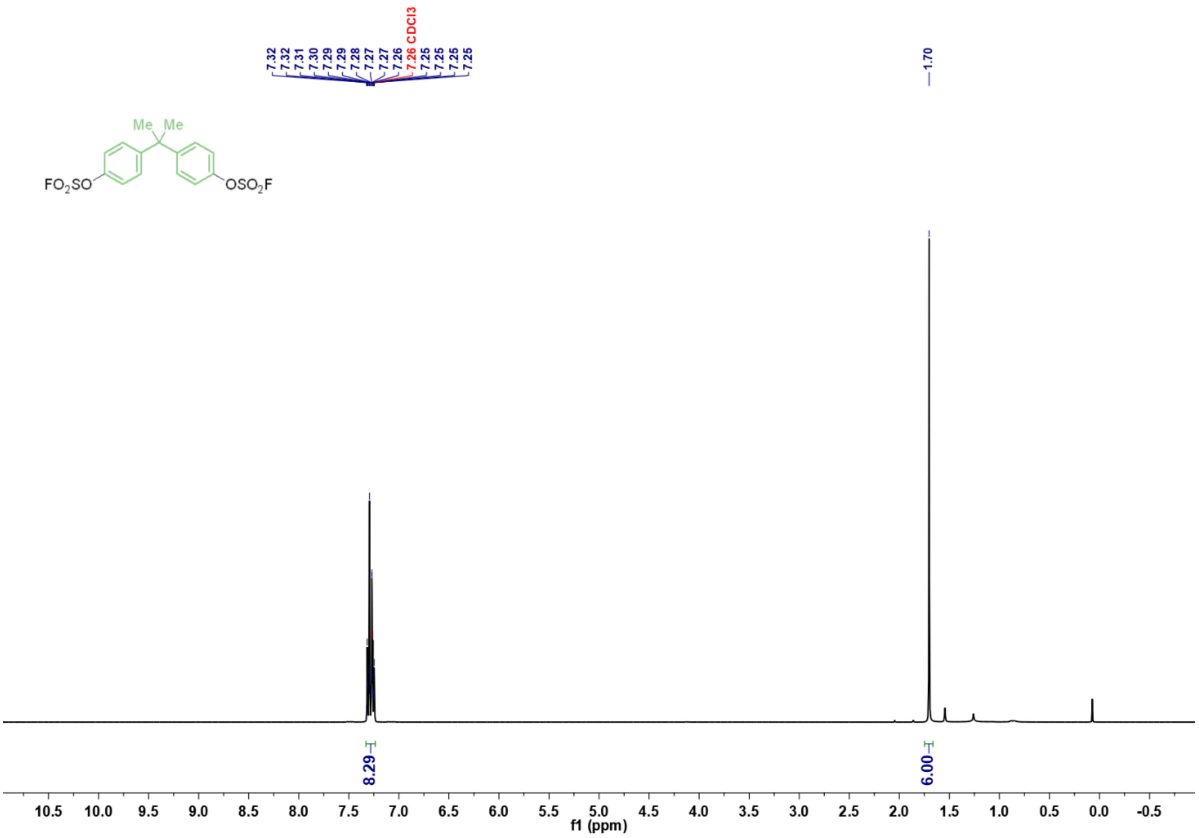

^{19}F NMR (376 MHz, $CDCl_3$) of **36**

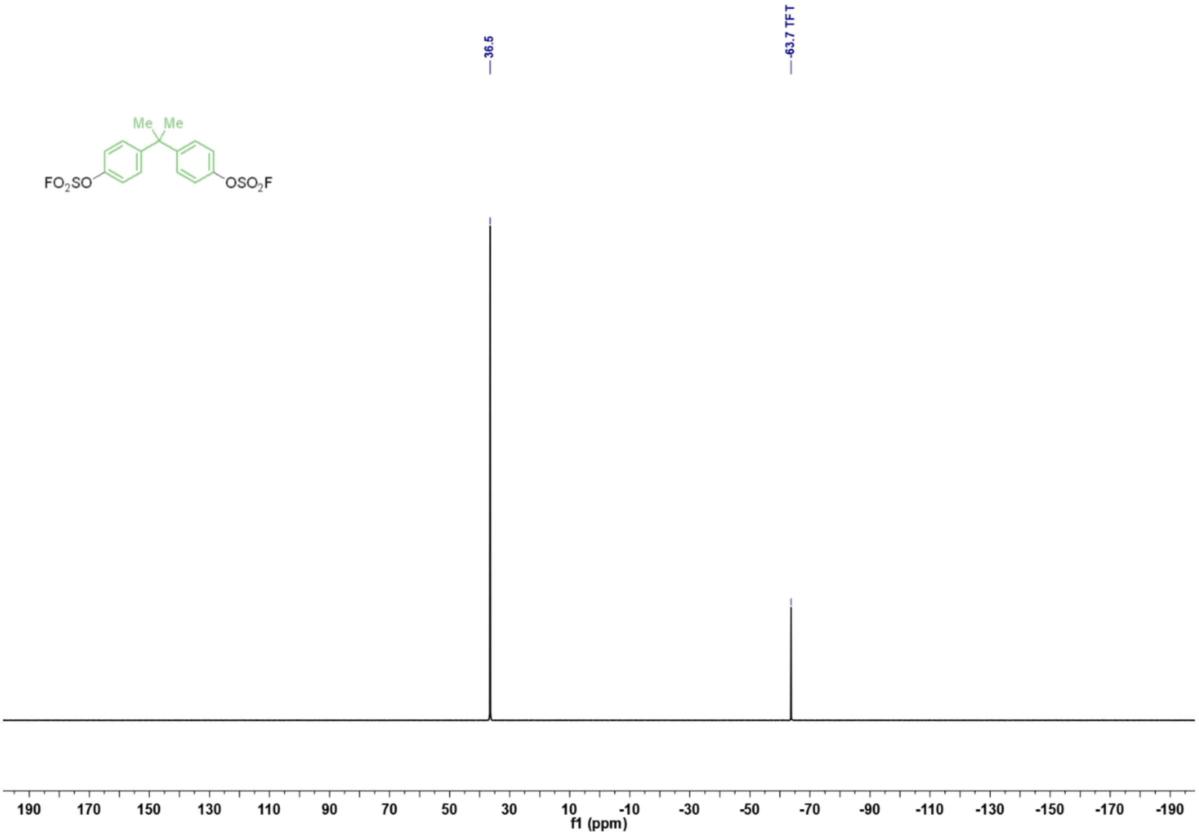

^{1}H NMR (400 MHz, CD_3CN) of **37**

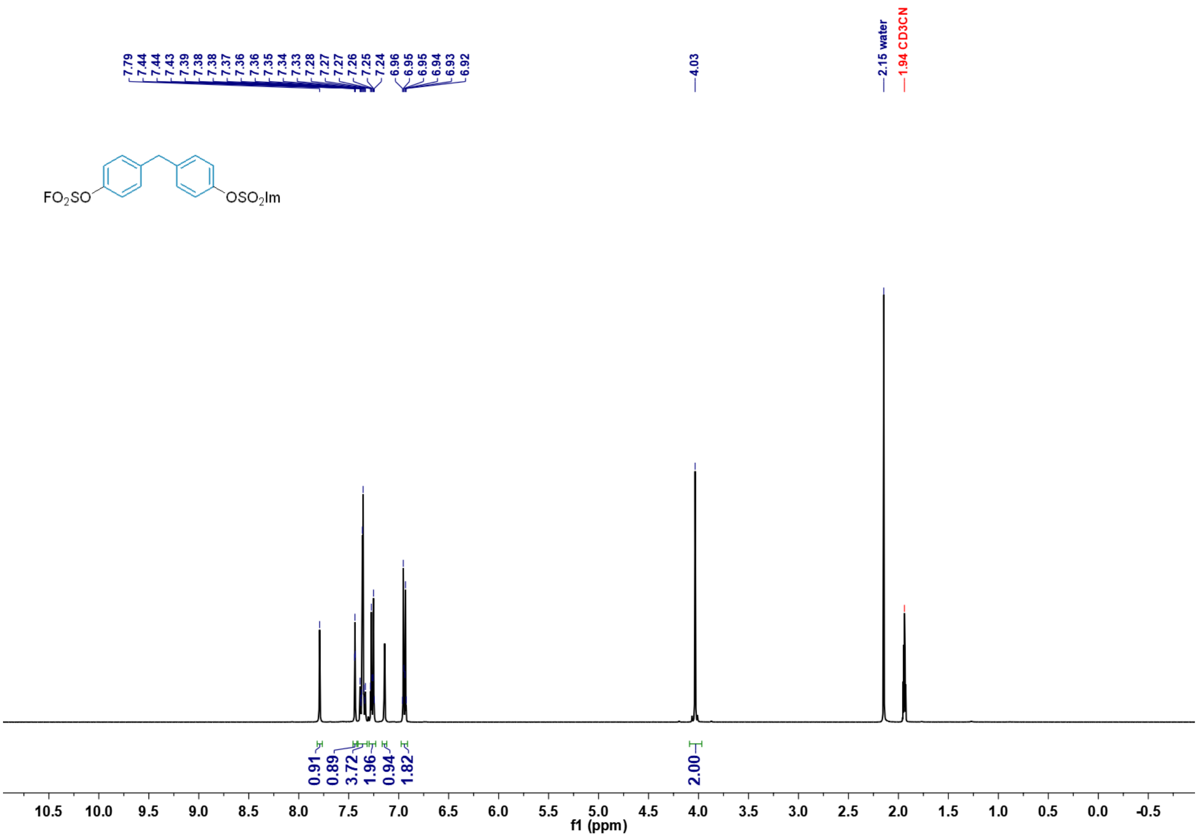

^{13}C NMR (101 MHz, CD_3CN) of **37**

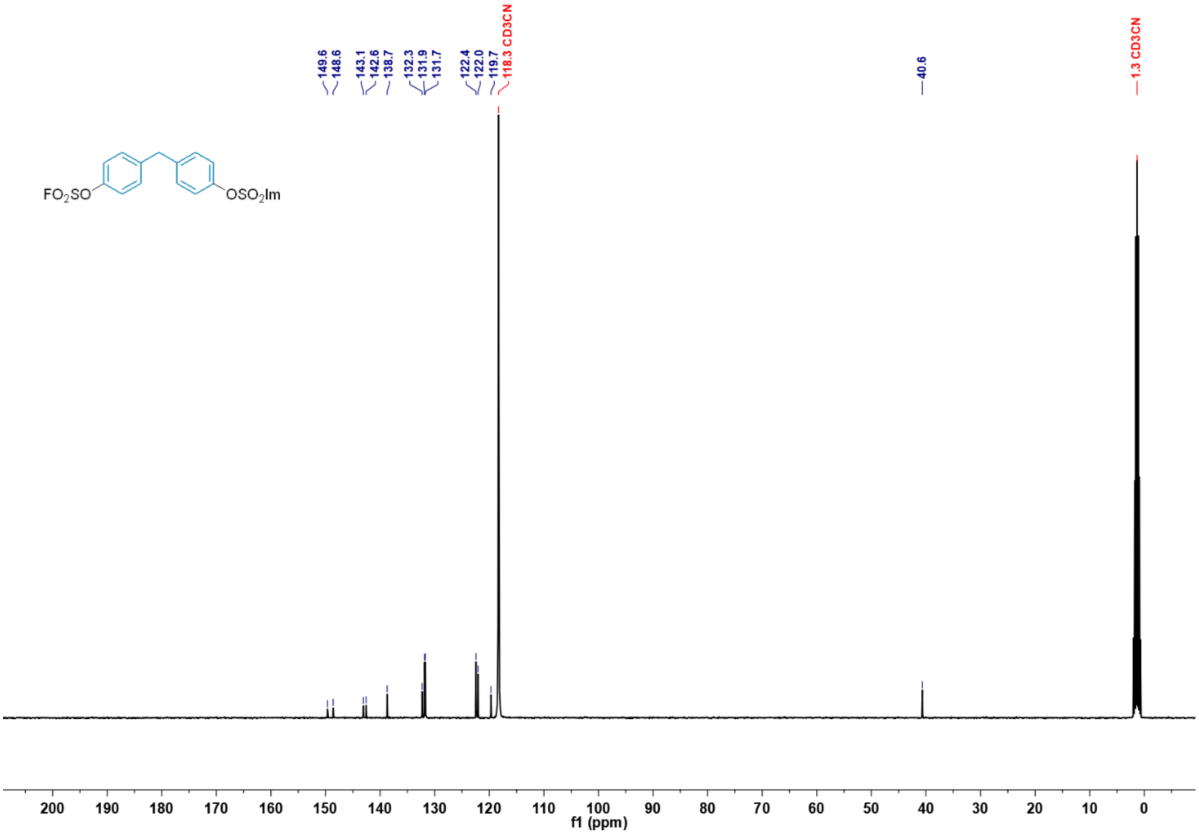

^{19}F NMR (376 MHz, CD_3CN) of **37**

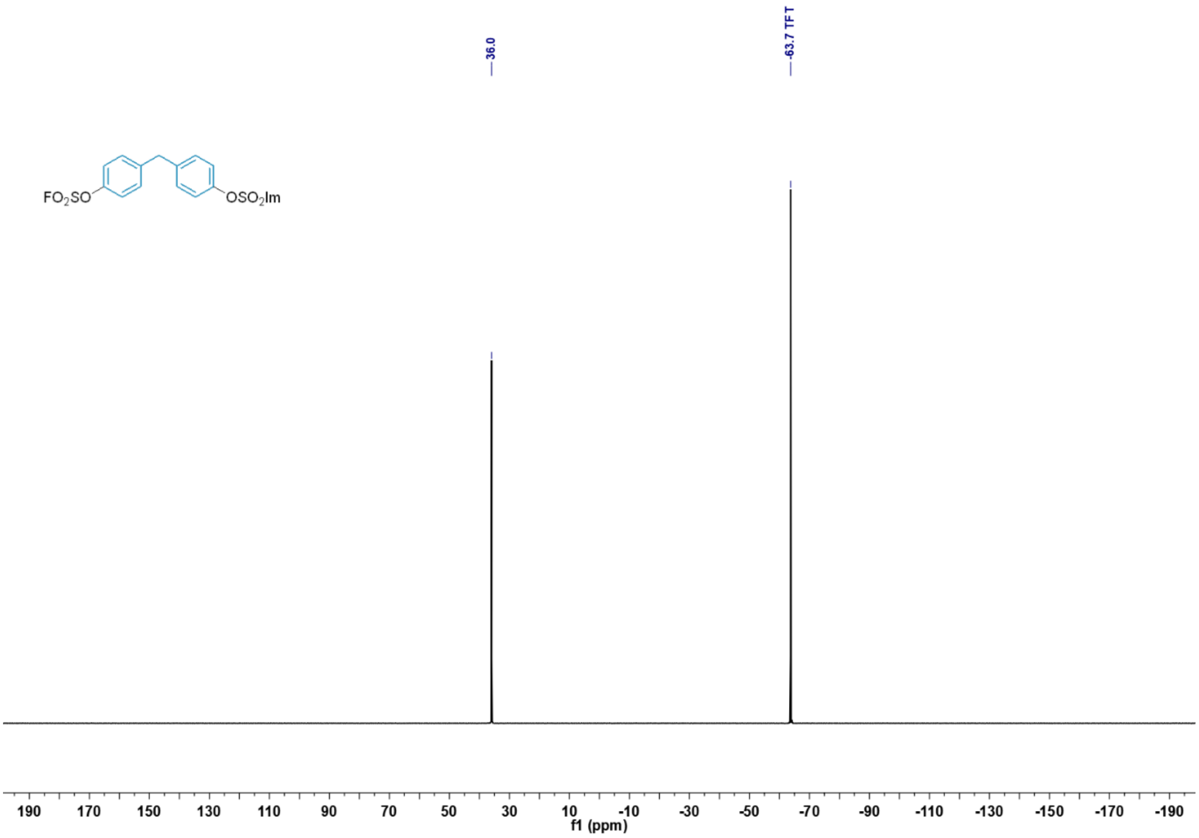

^{1}H NMR (400 MHz, DMSO-d_6) of **38**

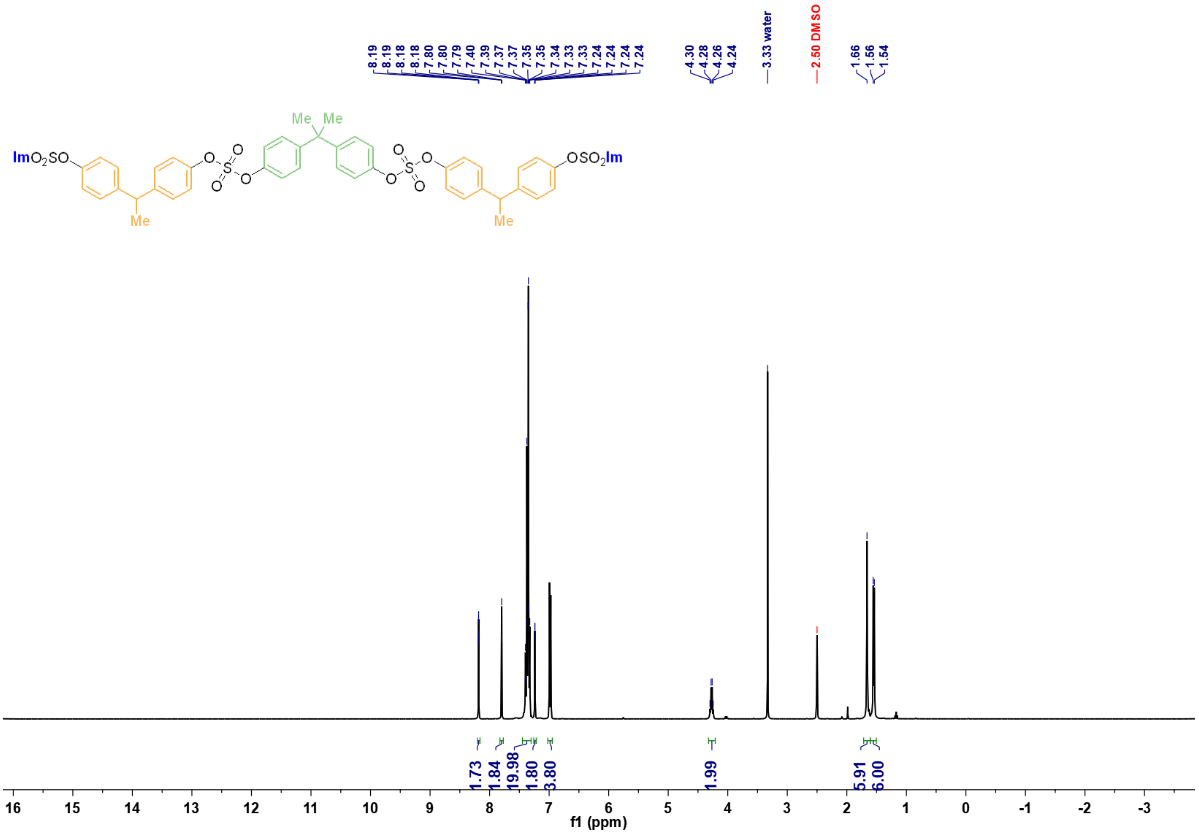

^{13}C NMR (101 MHz, DMSO-d_6) of **38**

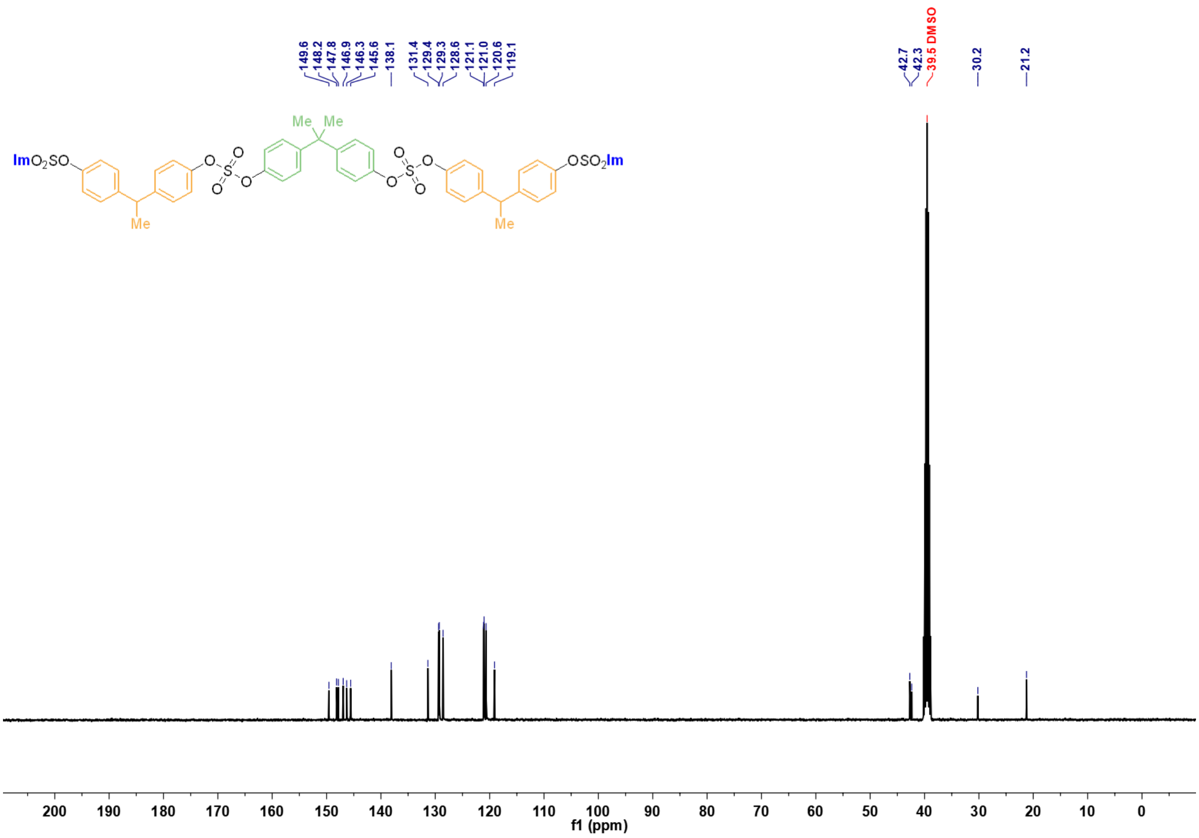

^{1}H NMR (400 MHz, DMSO-d_6) of **39**

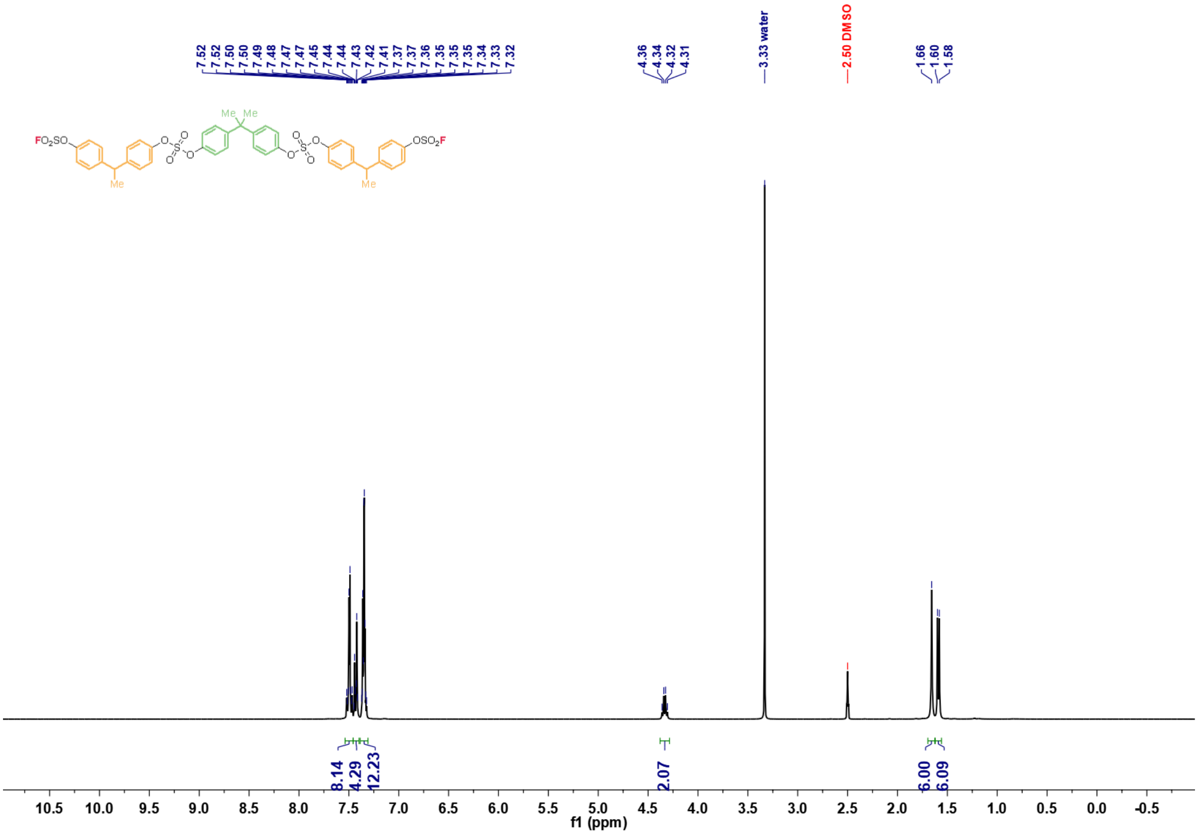

^{13}C NMR (101 MHz, DMSO-d_6) of **39**

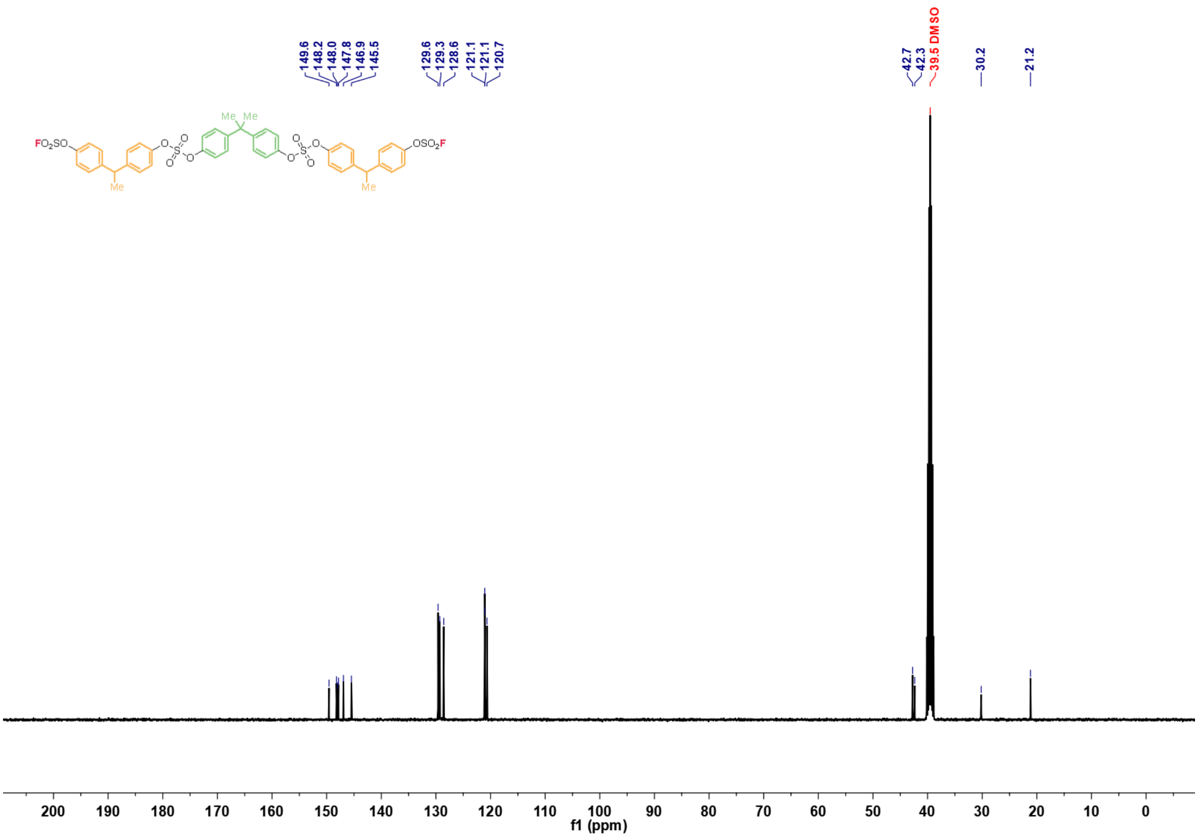

^{19}F NMR (376 MHz, DMSO-d_6) of **39**

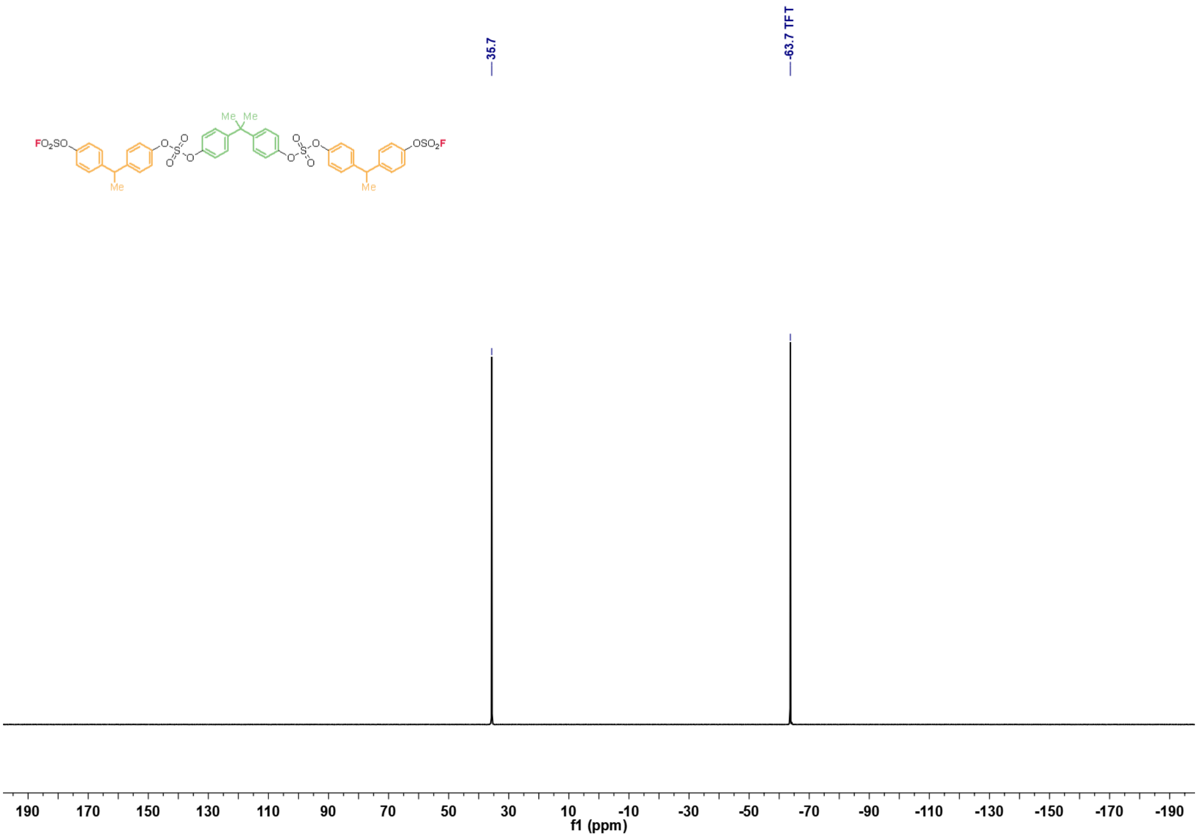

^{1}H NMR (400 MHz, CD_3CN) of **40**

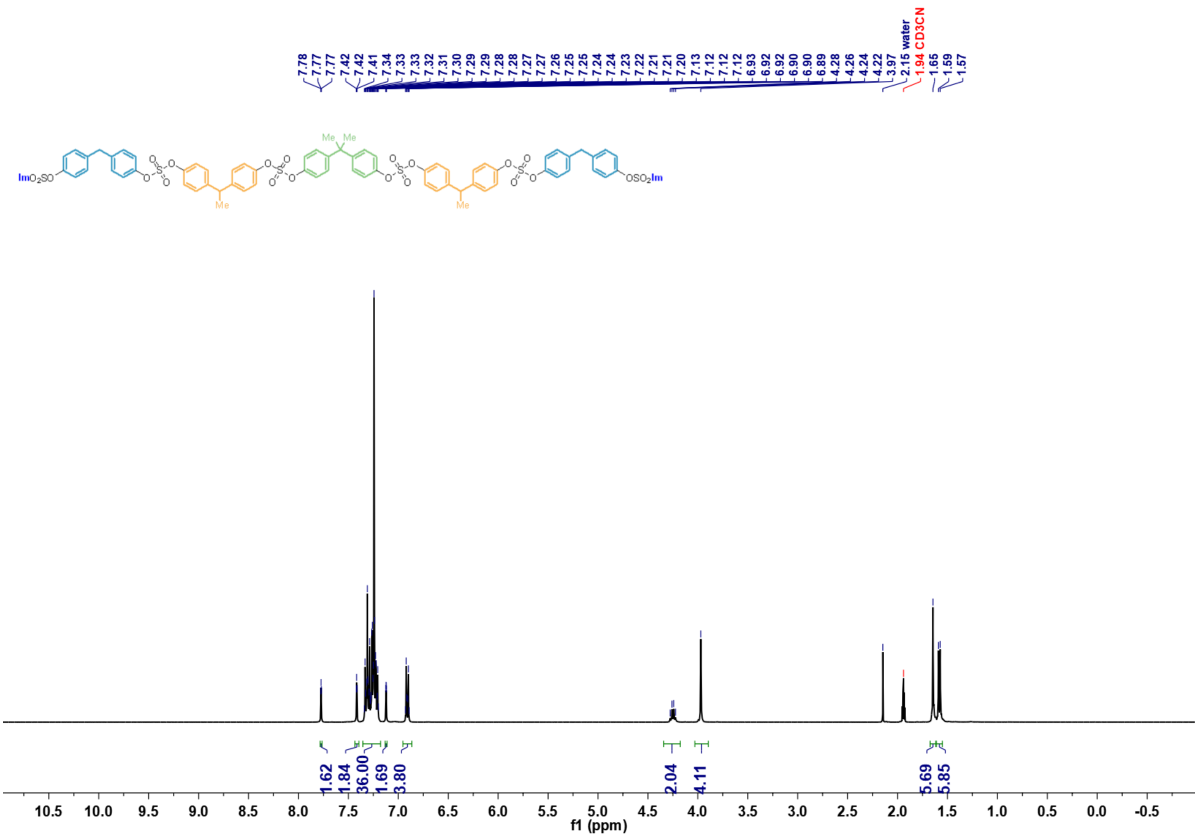

^{13}C NMR (101 MHz, CD_3CN) of **40**

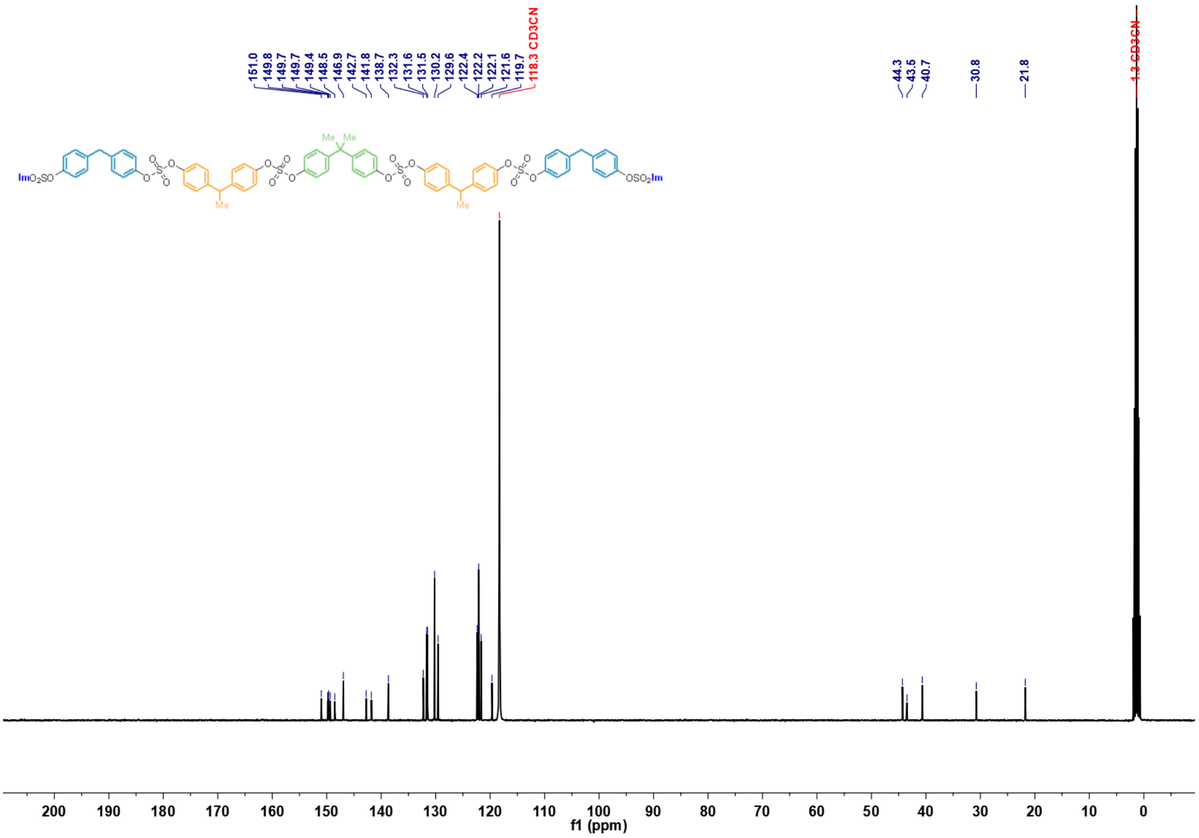

^{1}H NMR (400 MHz, CD_3CN) of **41**

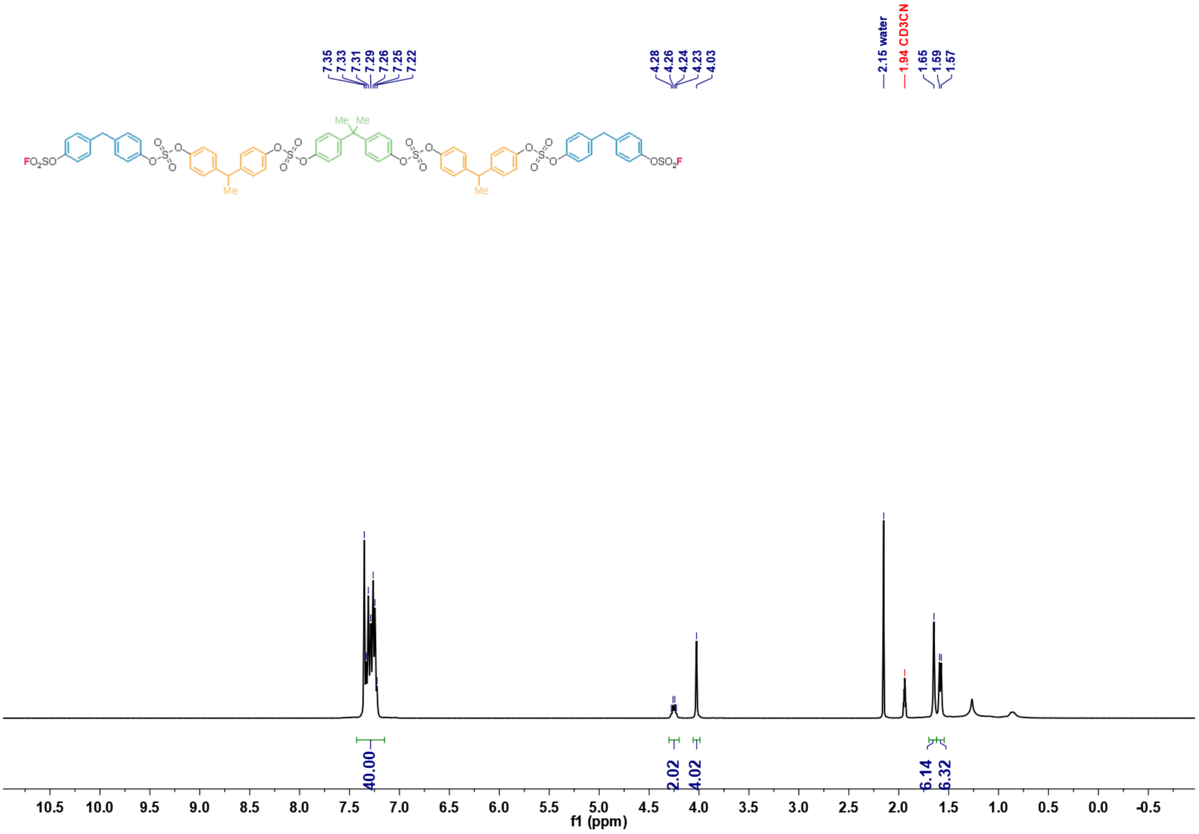

^{13}C NMR (101 MHz, CD_3CN) of **41**

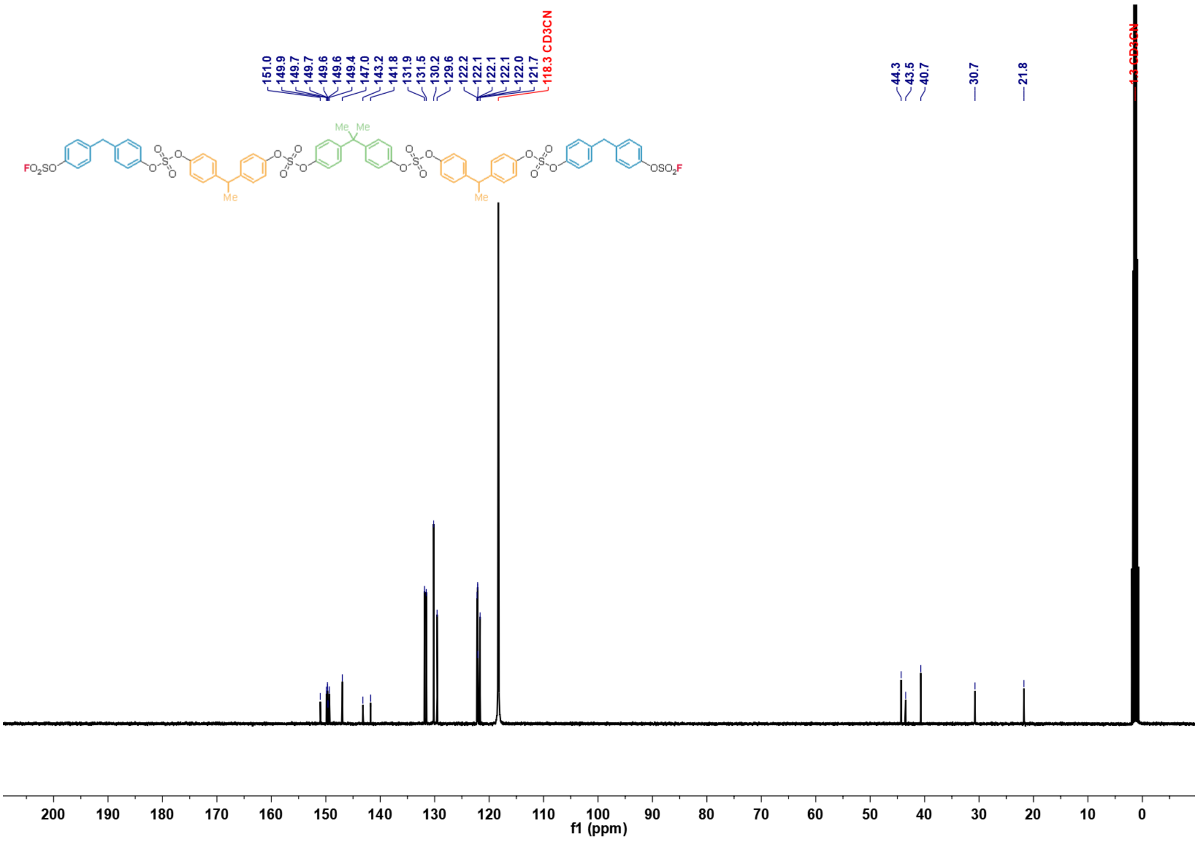

^{19}F NMR (376 MHz, CD_3CN) of **41**

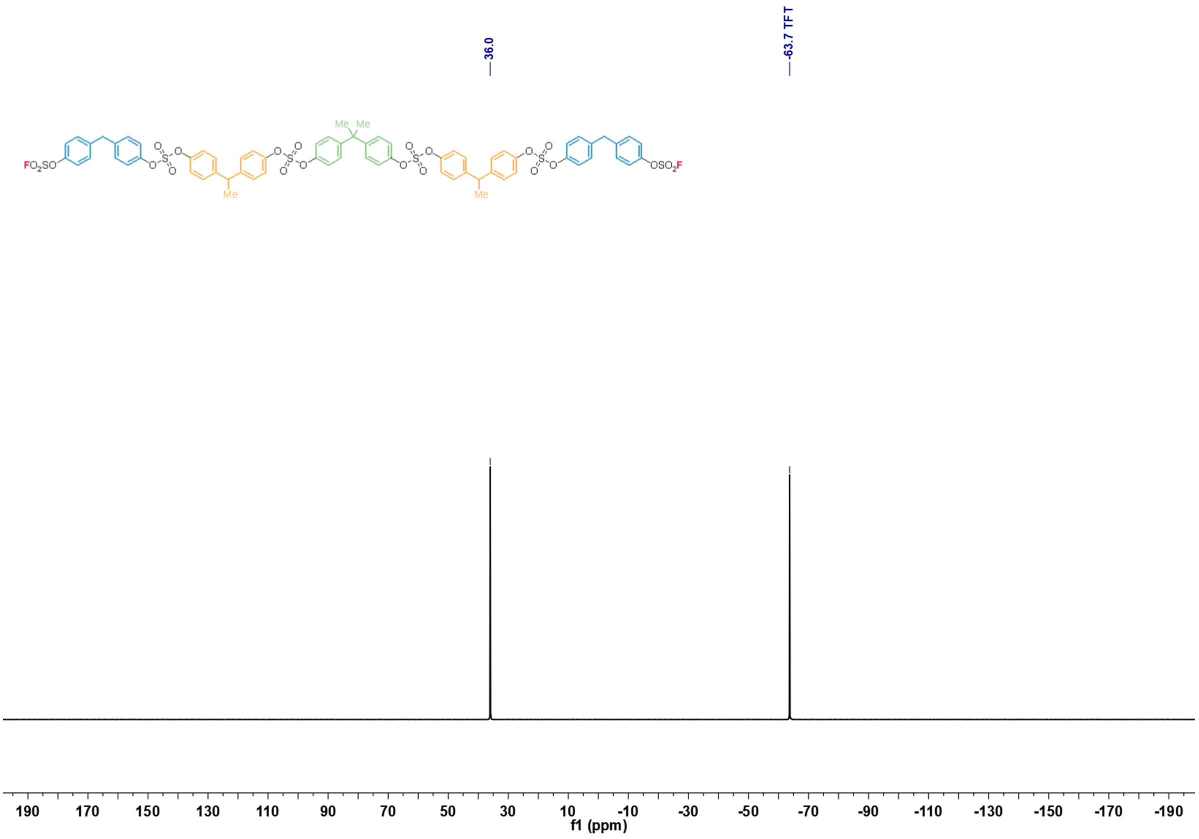

^{1}H NMR (400 MHz, CD_3CN) of **42**

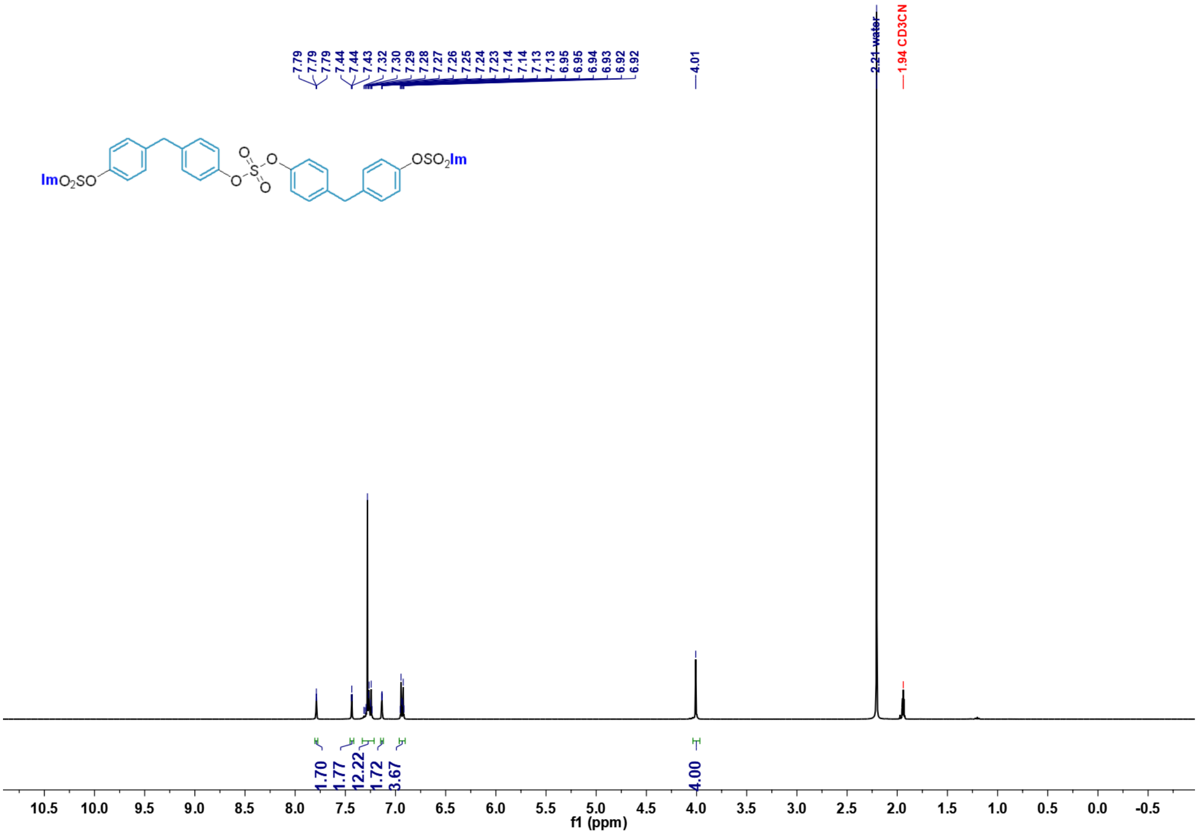

^{13}C NMR (101 MHz, CD_3CN) of **42**

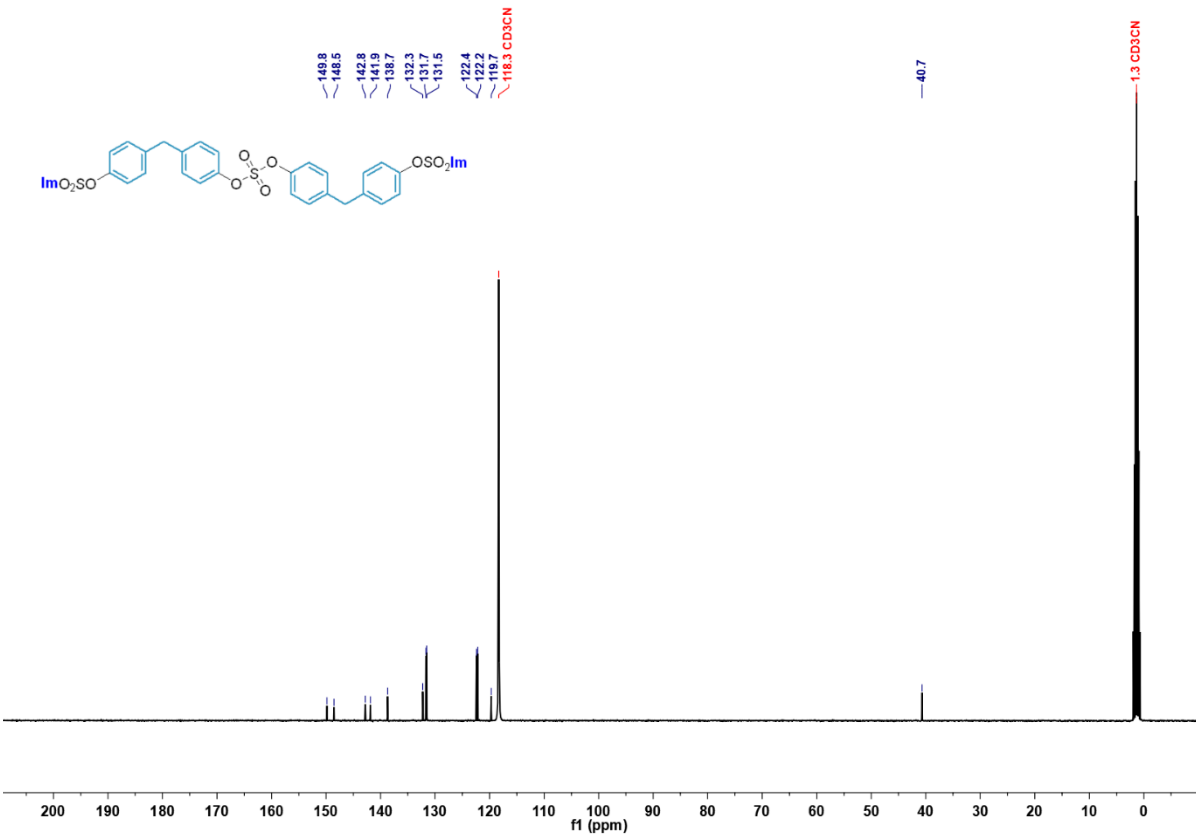

^{1}H NMR (400 MHz, CD_3CN) of **43**

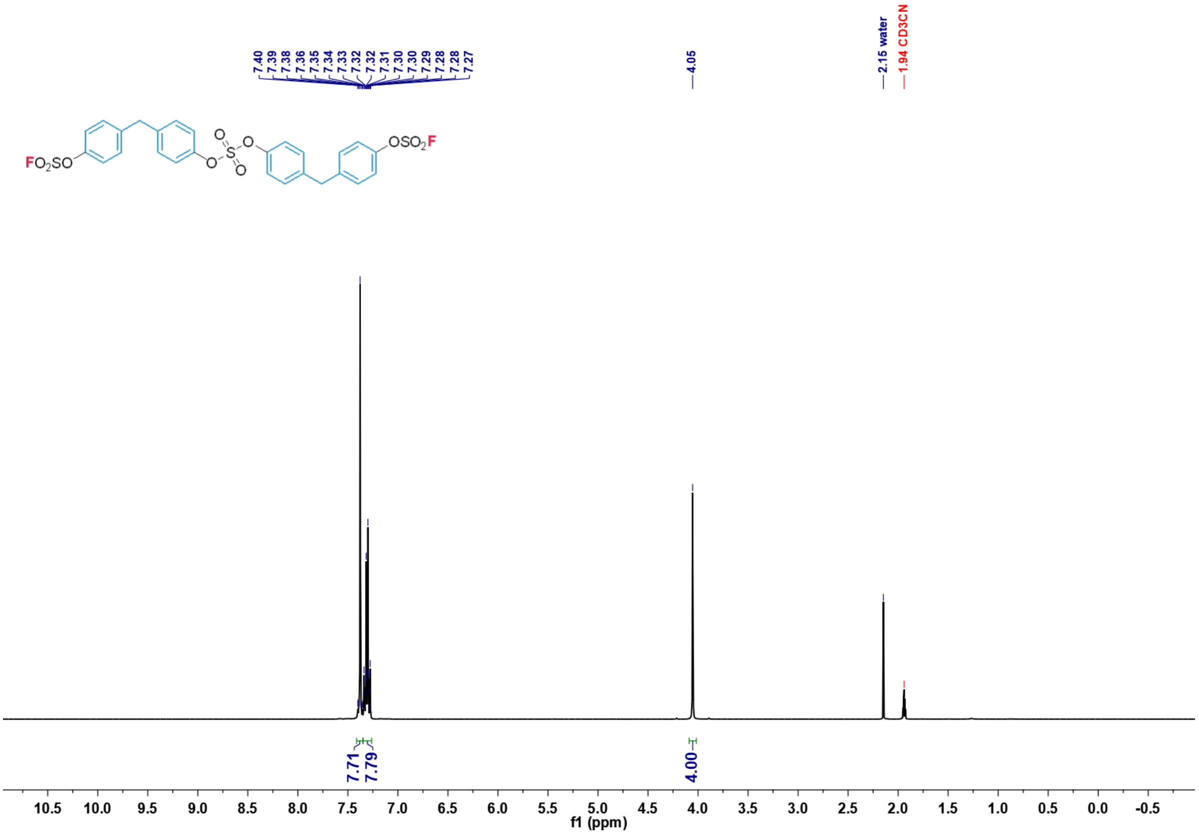

^{13}C NMR (101 MHz, CD_3CN) of **43**

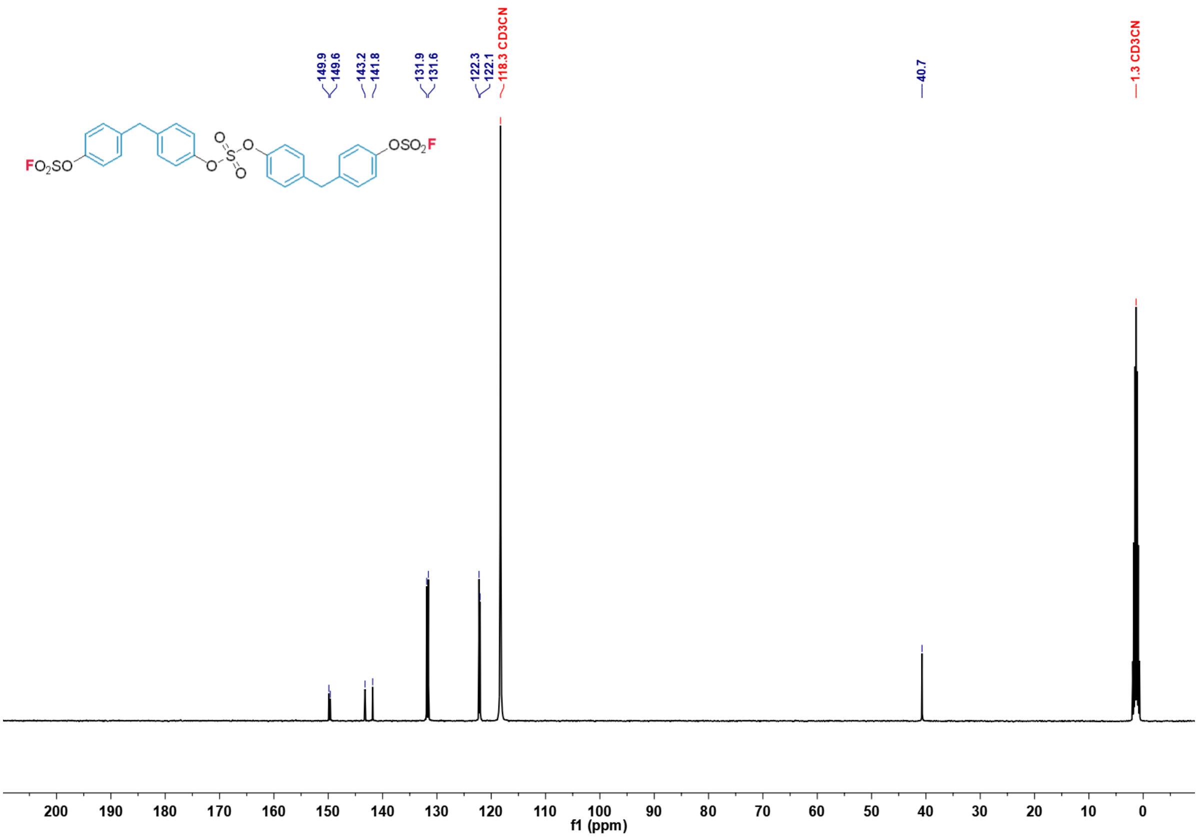

^{19}F NMR (376 MHz, CD_3CN) of **43**

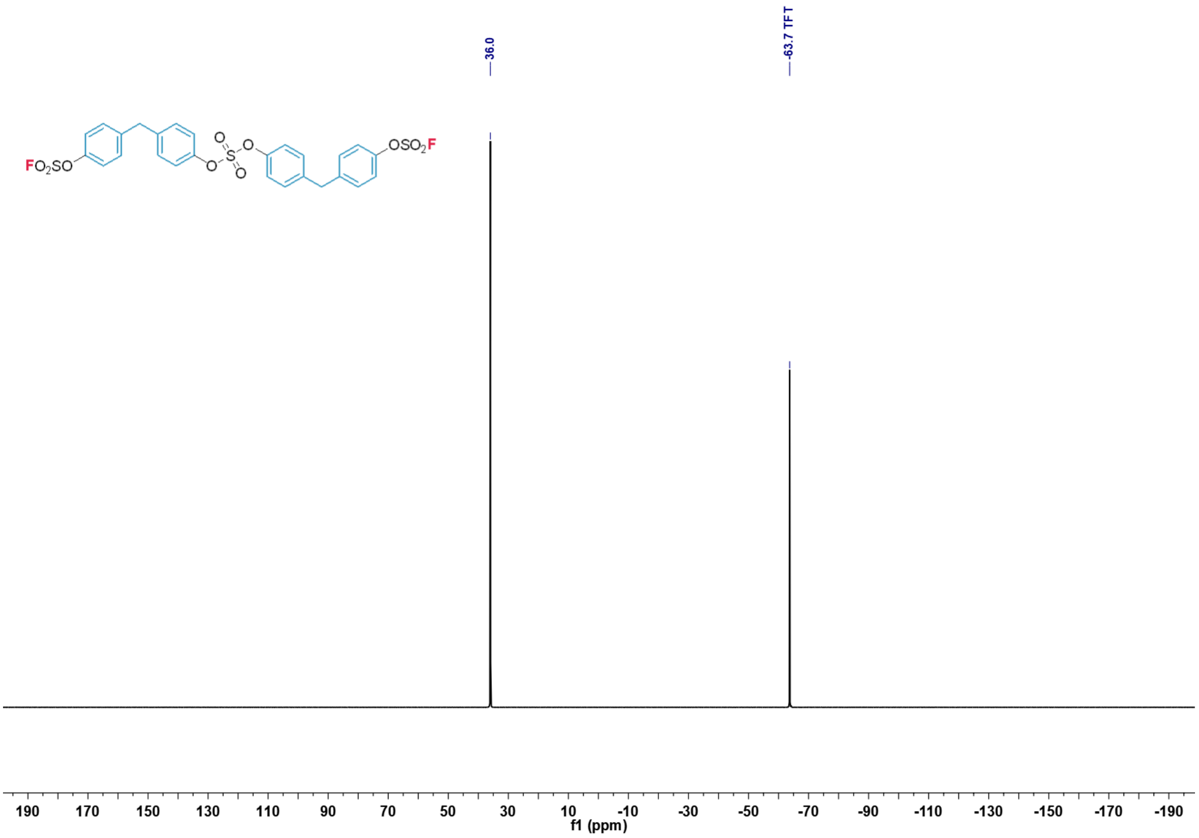

^{1}H NMR (400 MHz, CD_3CN) of **44**

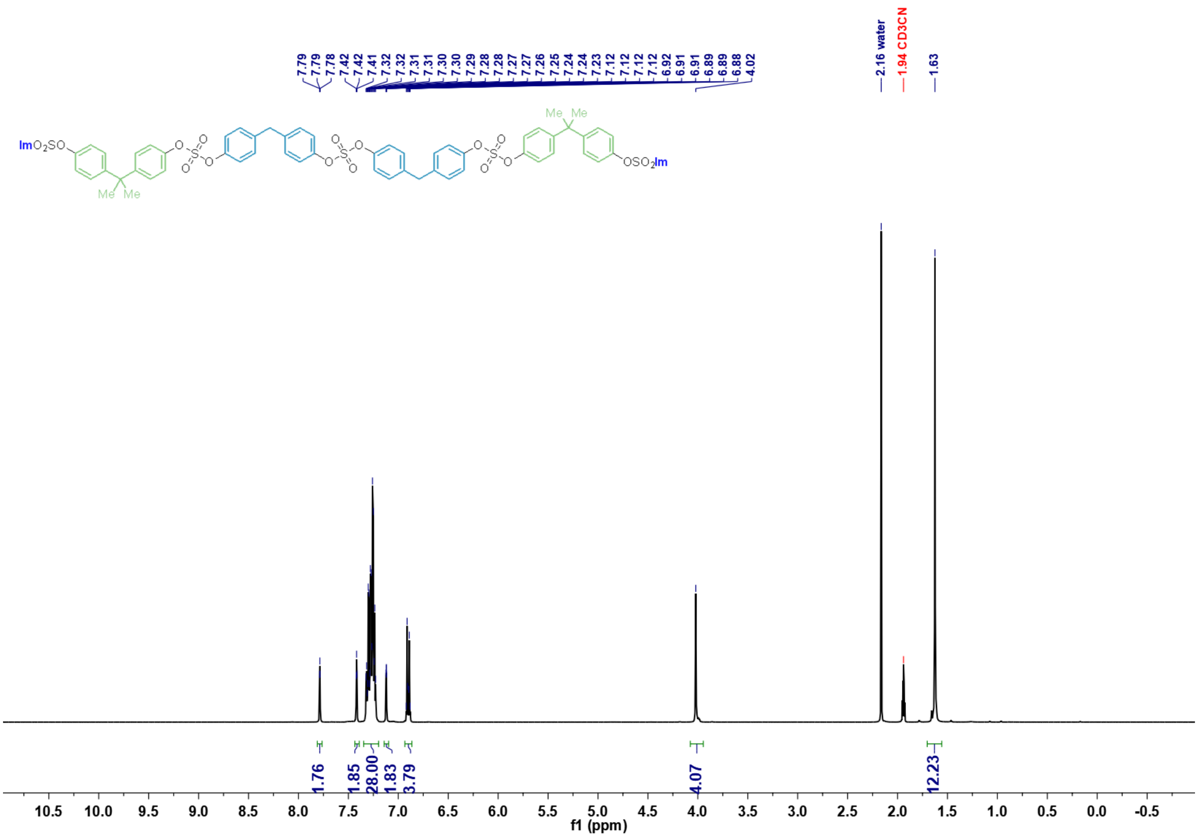

^{13}C NMR (101 MHz, CD_3CN) of **44**

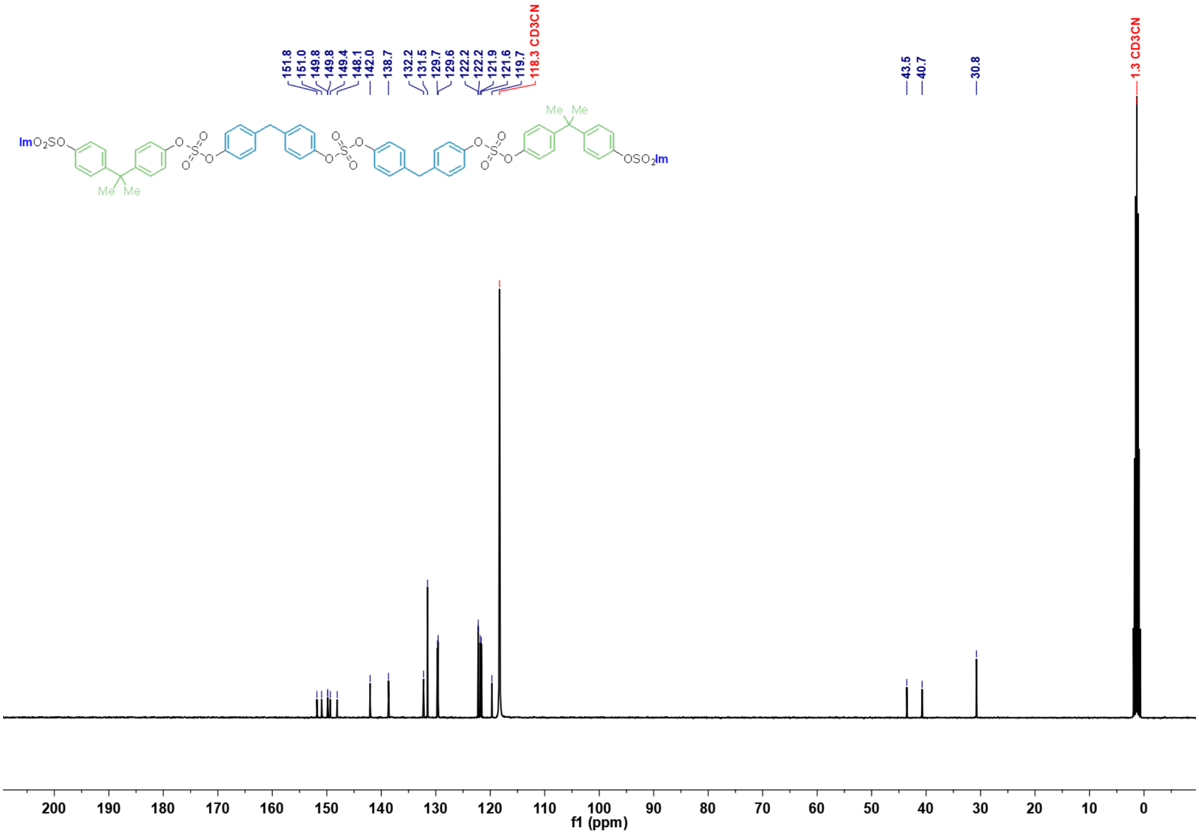

^{1}H NMR (400 MHz, CD_3CN) of **45**

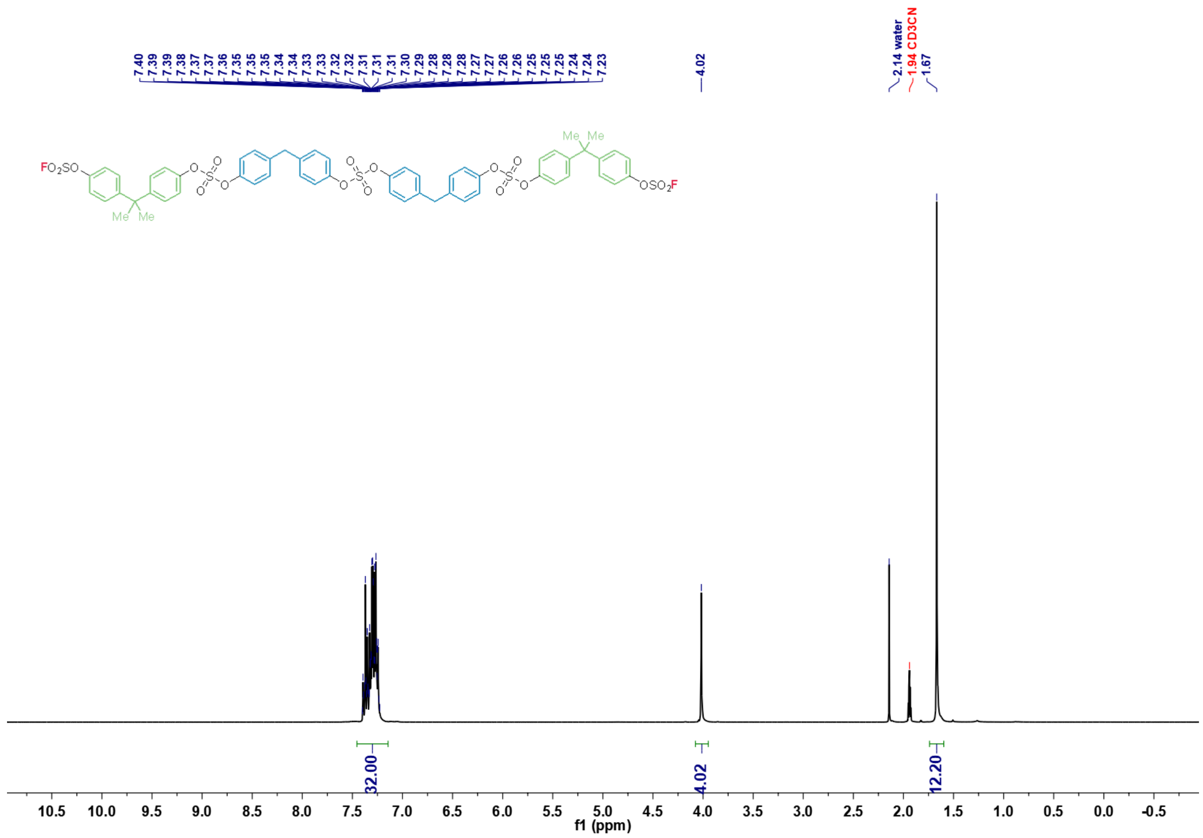

^{13}C NMR (101 MHz, CD_3CN) of **45**

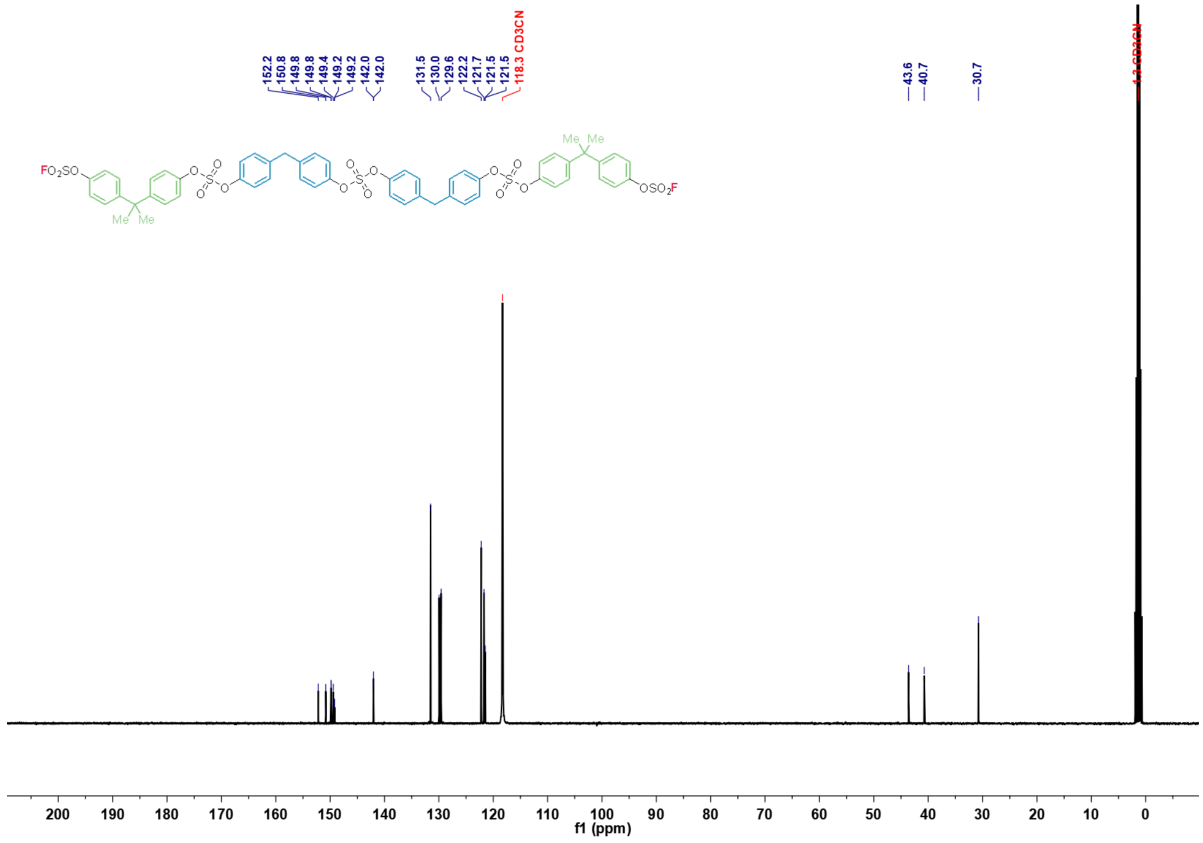

^{19}F NMR (376 MHz, CD_3CN) of **45**

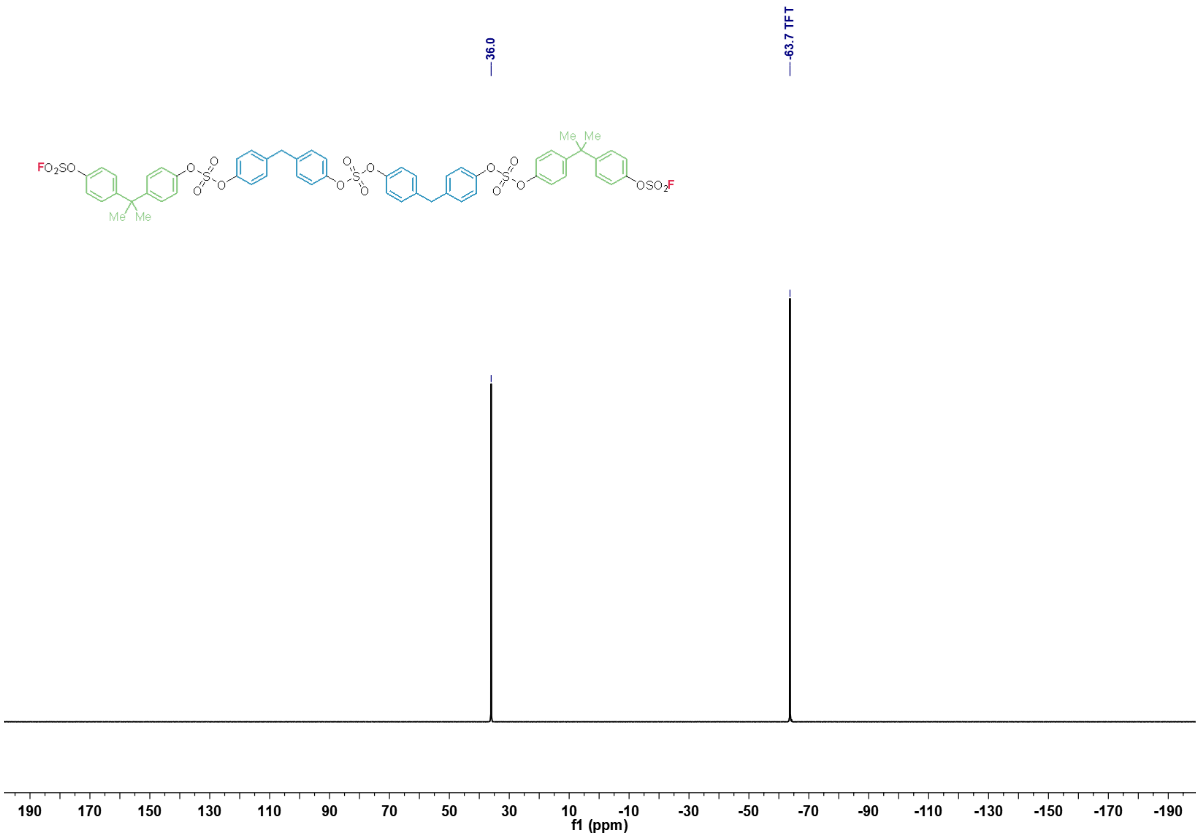

^{1}H NMR (400 MHz, CD_3CN) of **46**

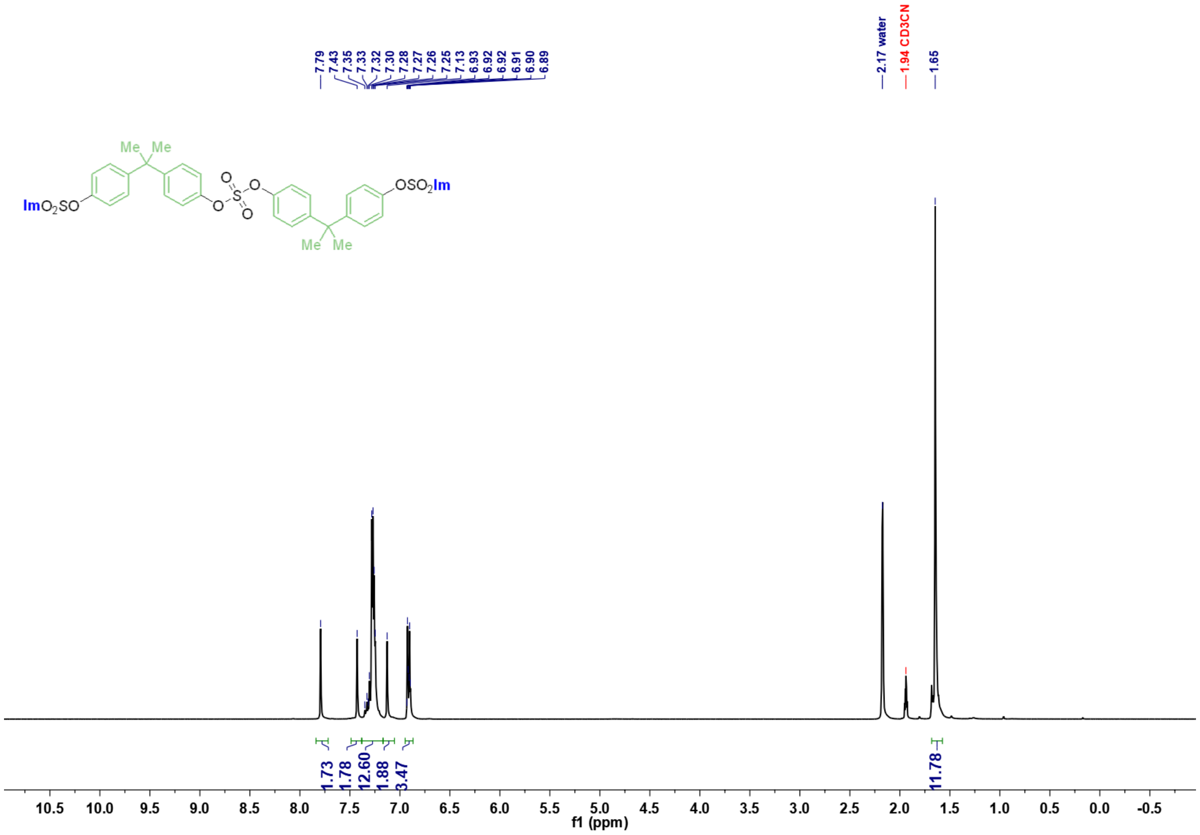

^{13}C NMR (101 MHz, CD_3CN) of **46**

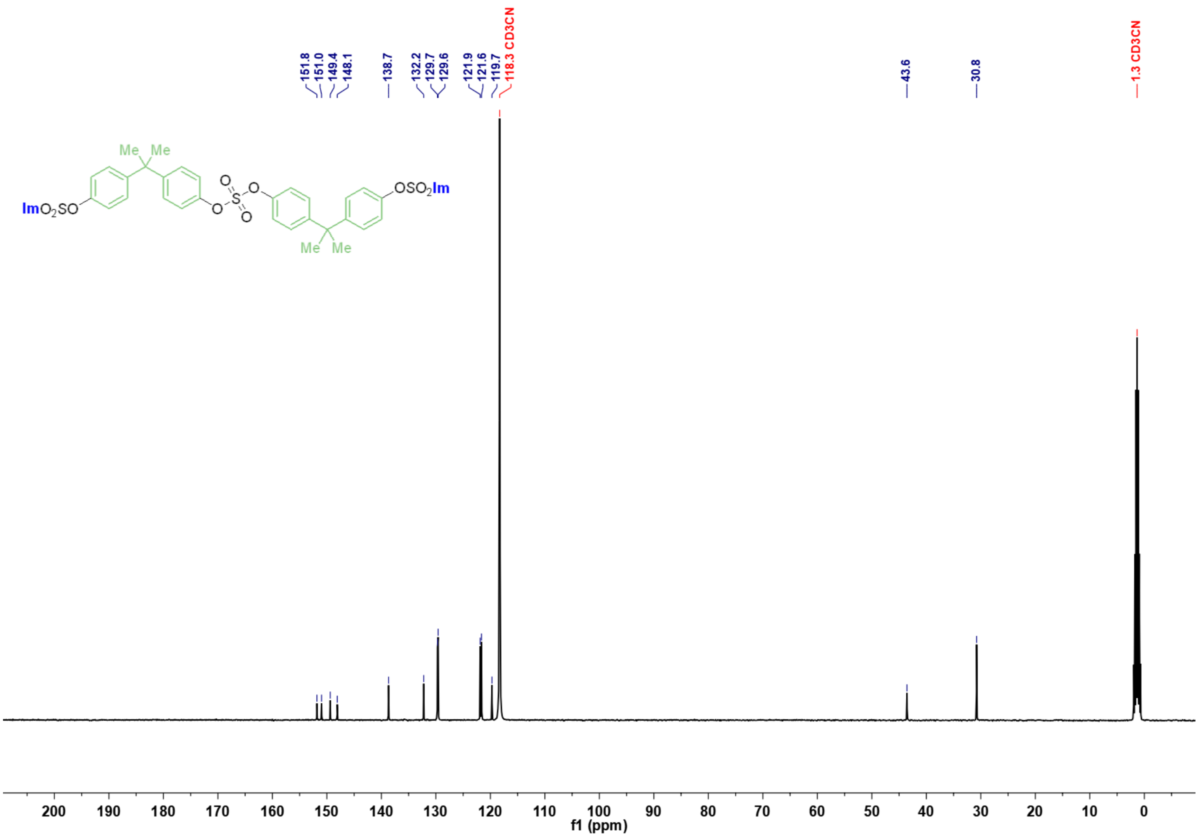

^{1}H NMR (400 MHz, CD_3CN) of **47**

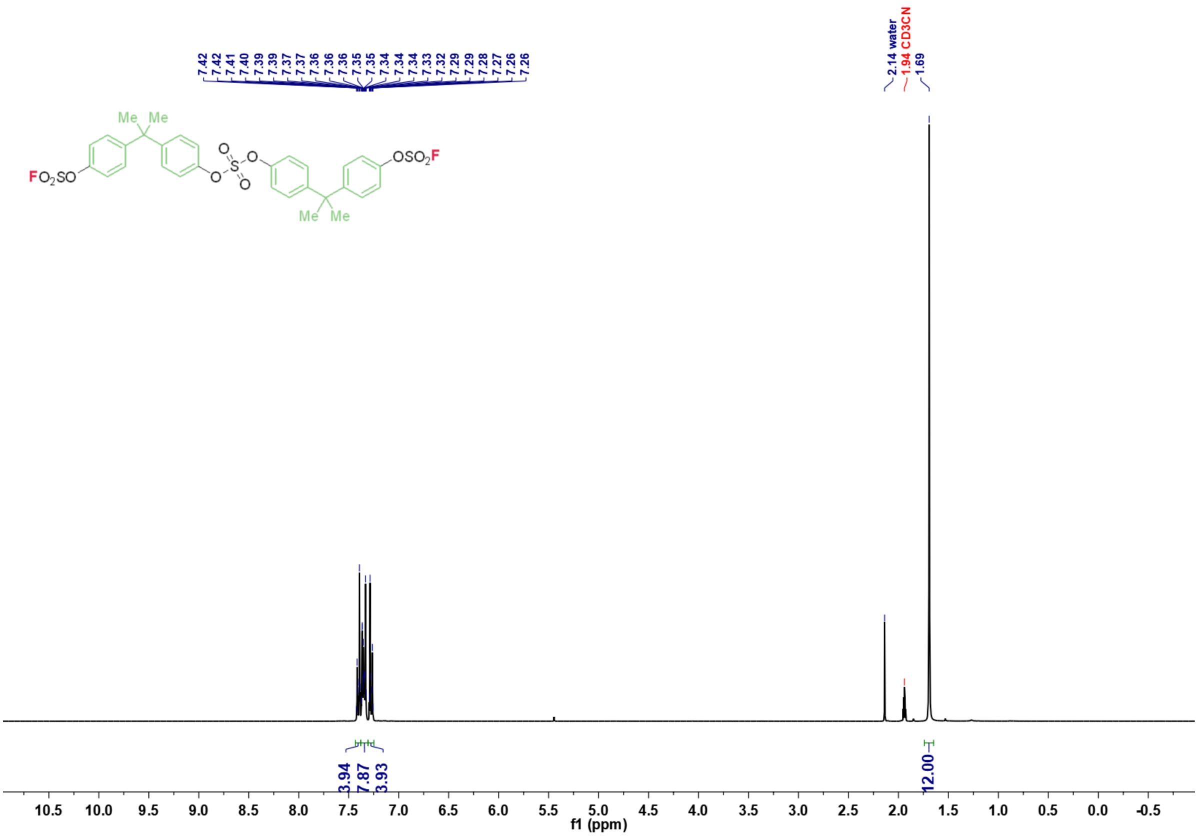

^{13}C NMR (101 MHz, CD_3CN) of **47**

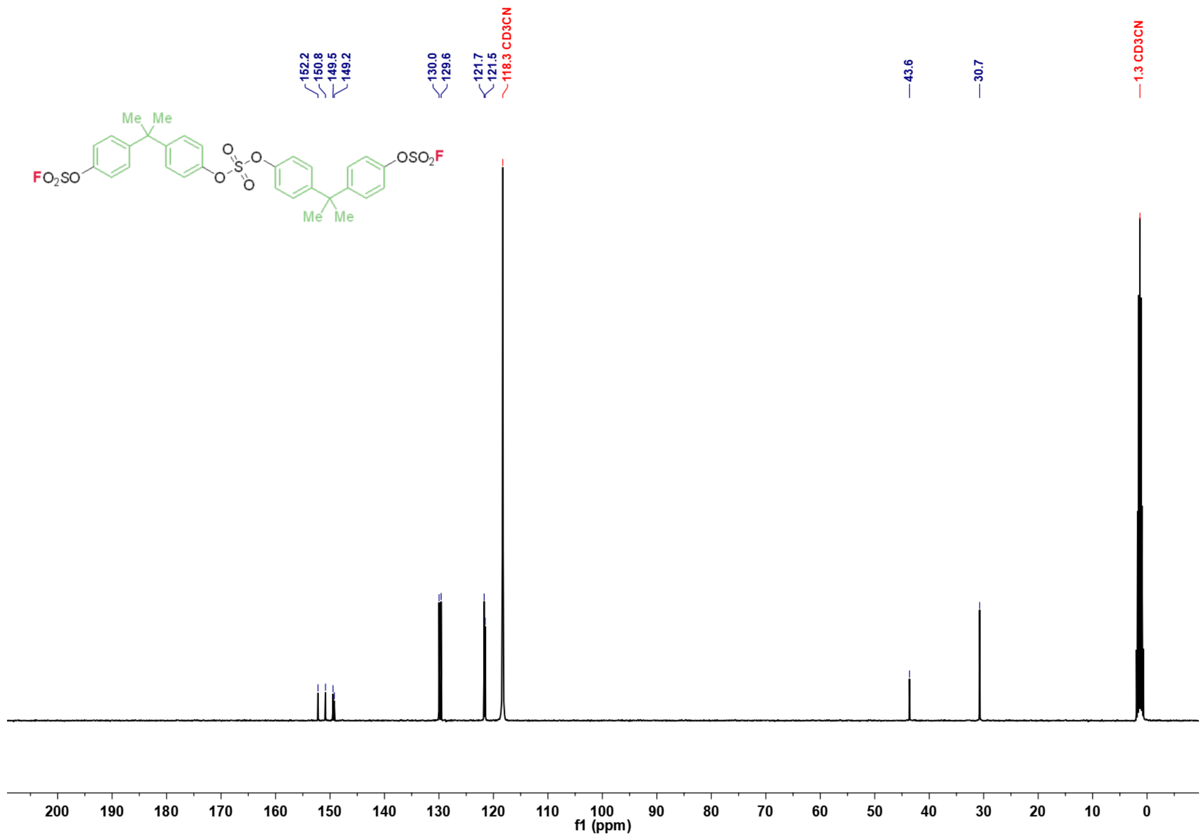

^{19}F NMR (376 MHz, CD_3CN) of **47**

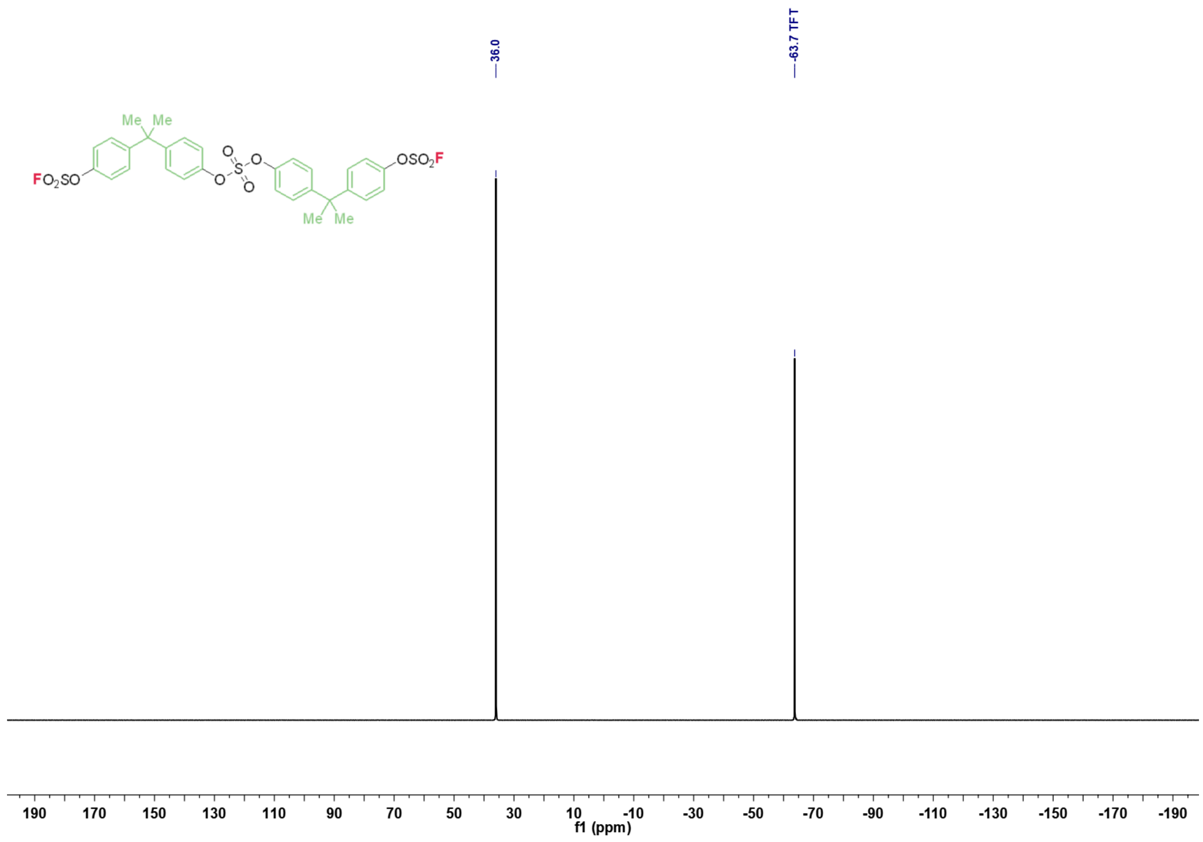

^{1}H NMR (400 MHz, DMSO-d_6) of **48**

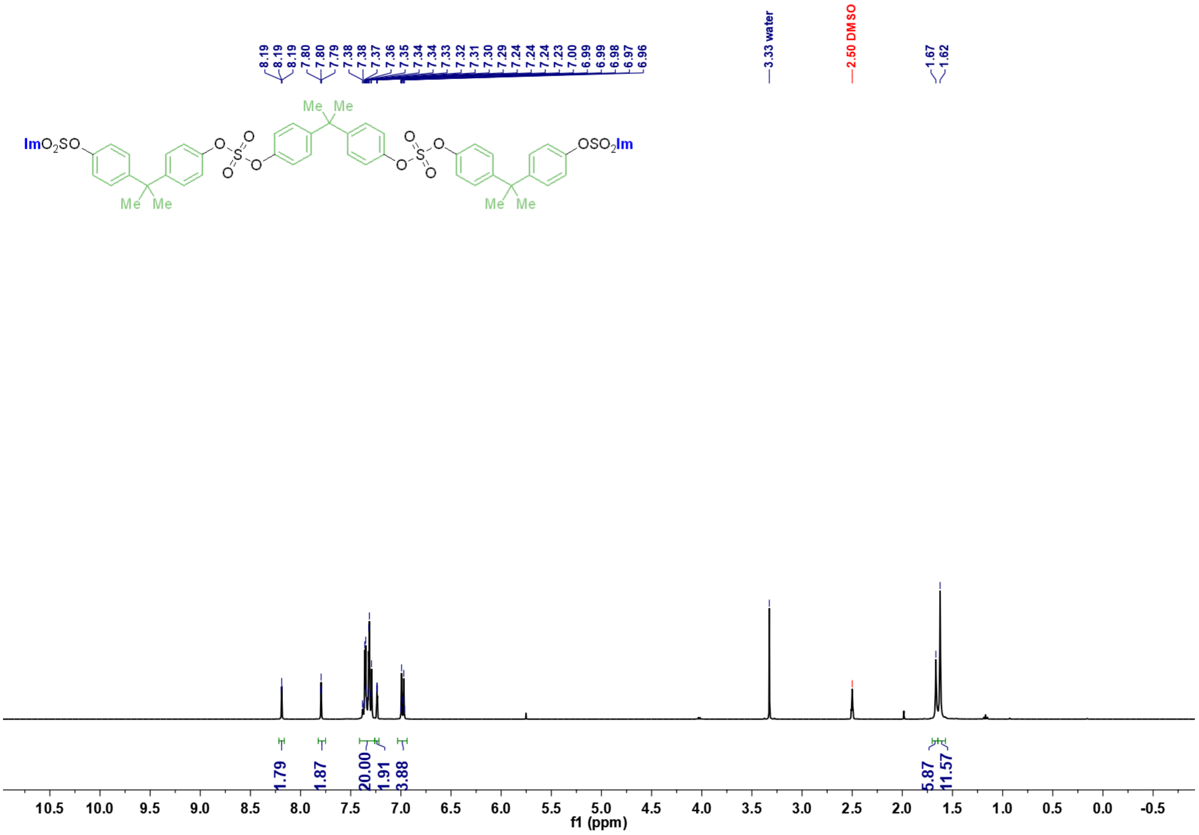

^{13}C NMR (101 MHz, DMSO-d_6) of **48**

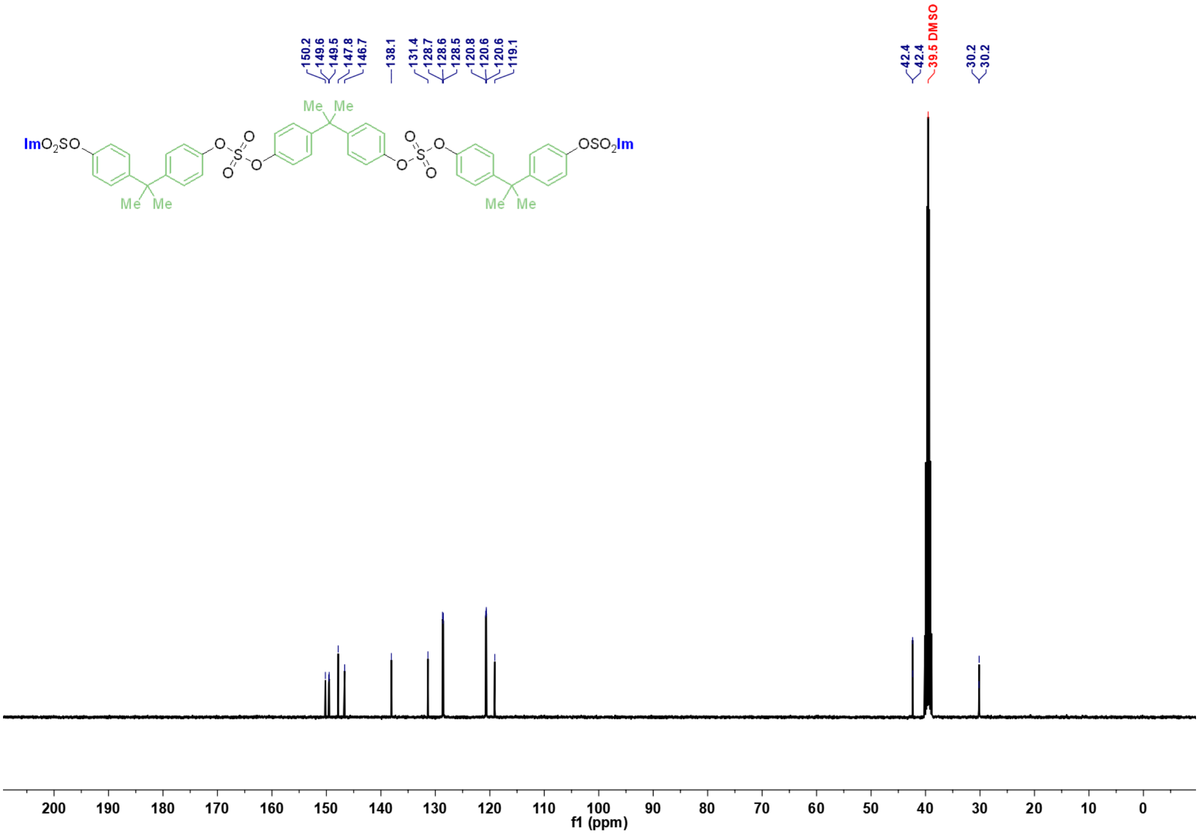

^{1}H NMR (400 MHz, DMSO-d_6) of **49**

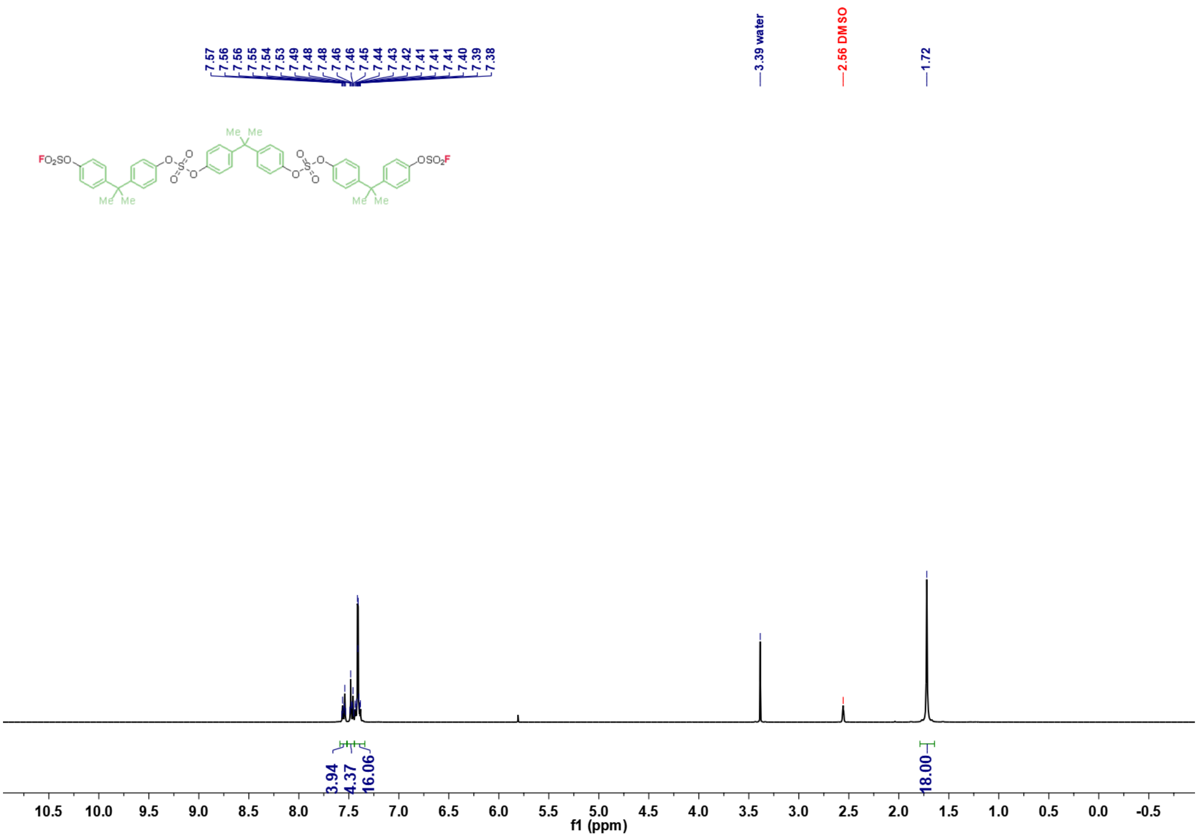

^{13}C NMR (101 MHz, DMSO-d_6) of **49**

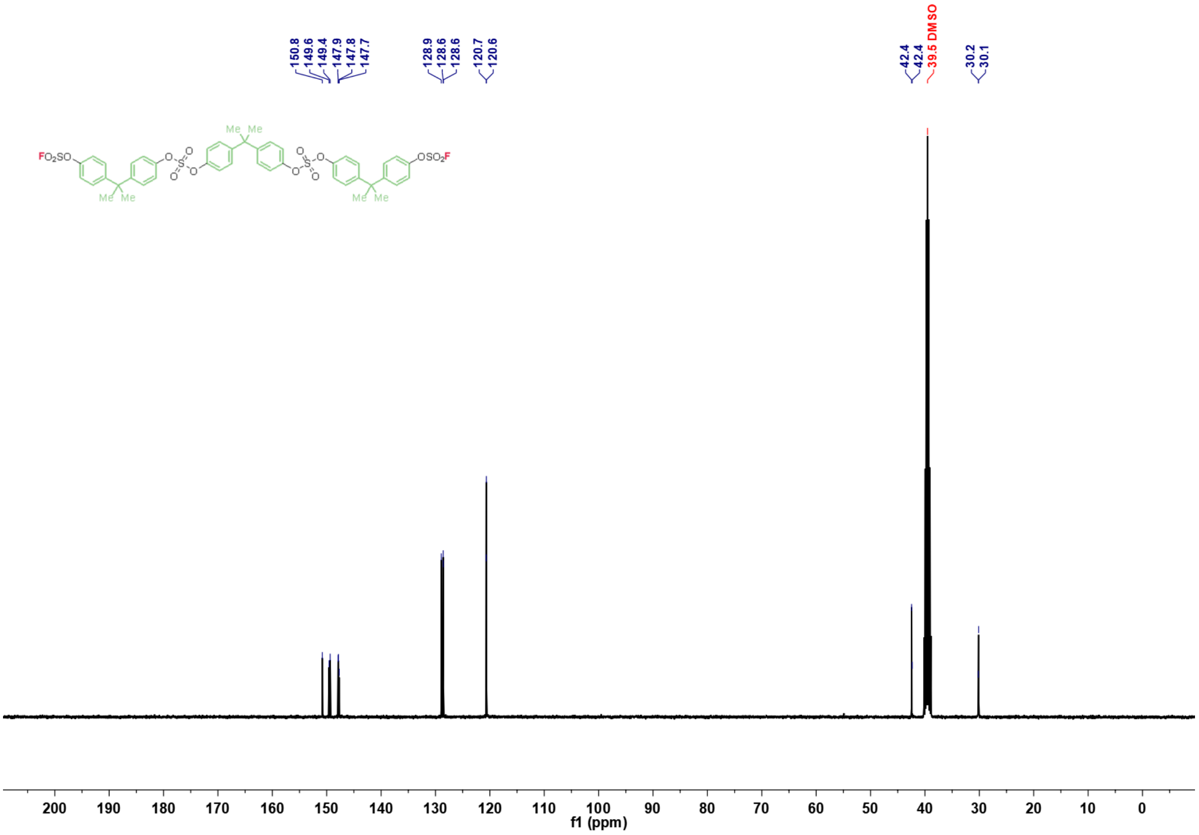

^{19}F NMR (376 MHz, DMSO-d_6) of **49**

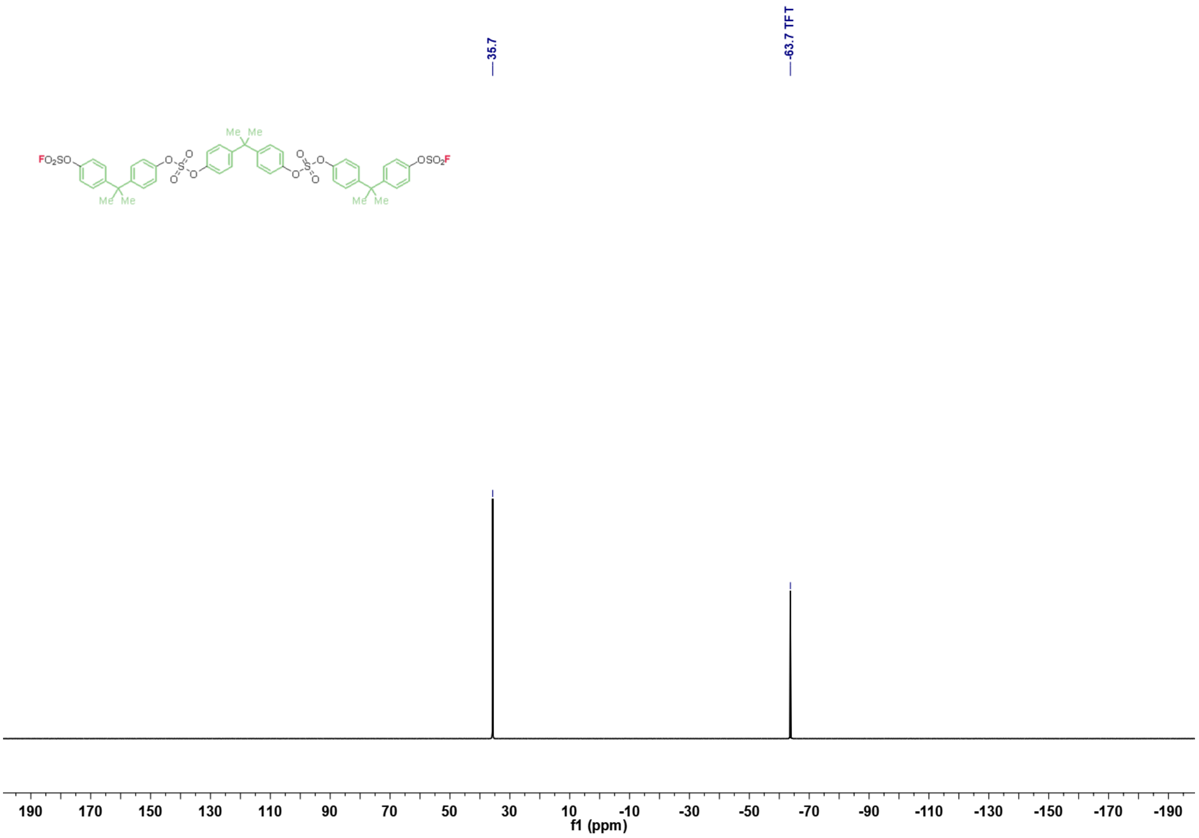

^{1}H NMR (400 MHz, CD_3CN) of **50**

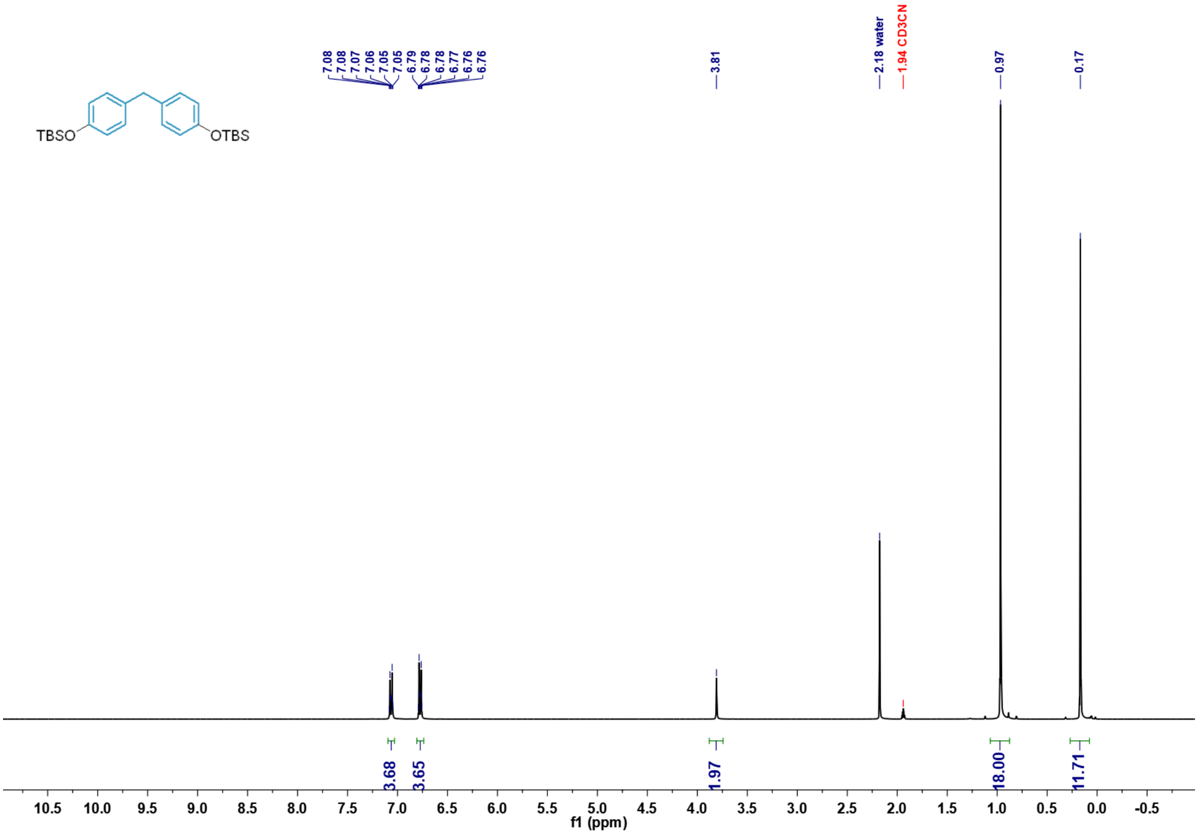

^{13}C NMR (101 MHz, CD_3CN) of **50**

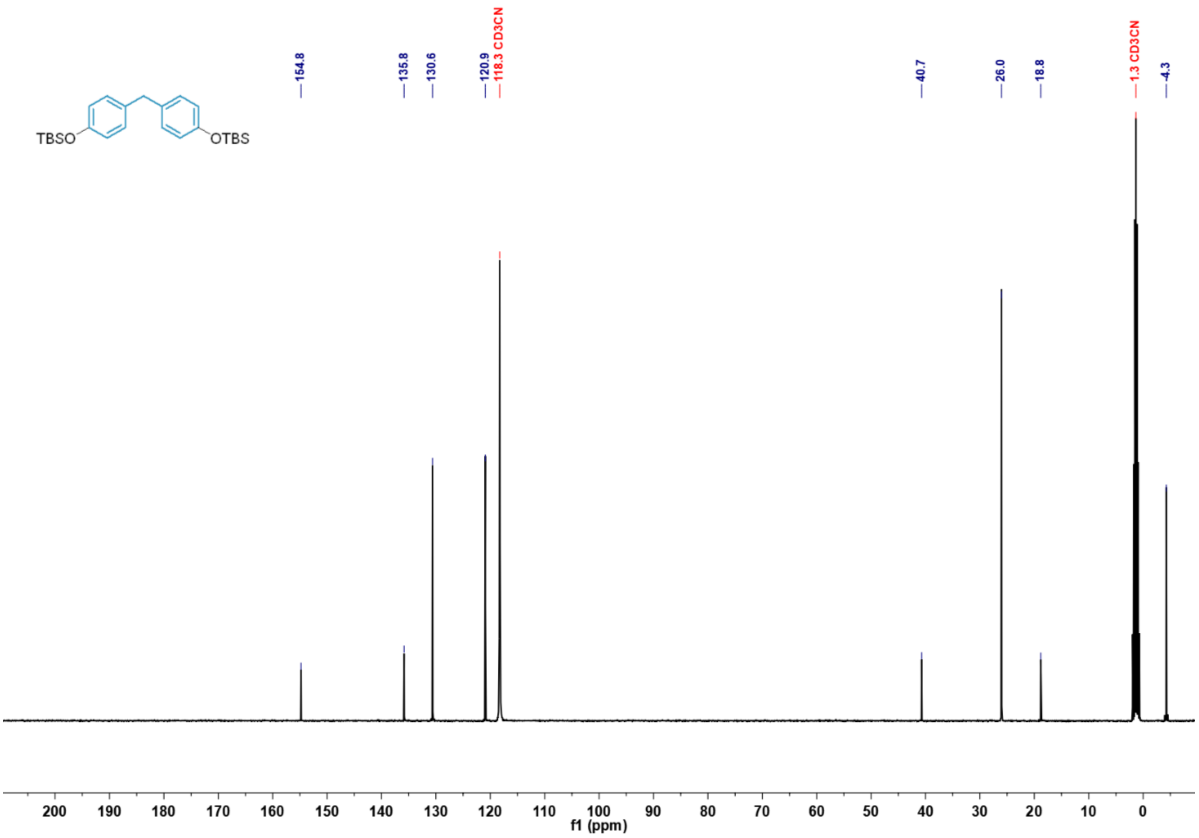

^{1}H NMR (400 MHz, $CDCl_3$) of **51**

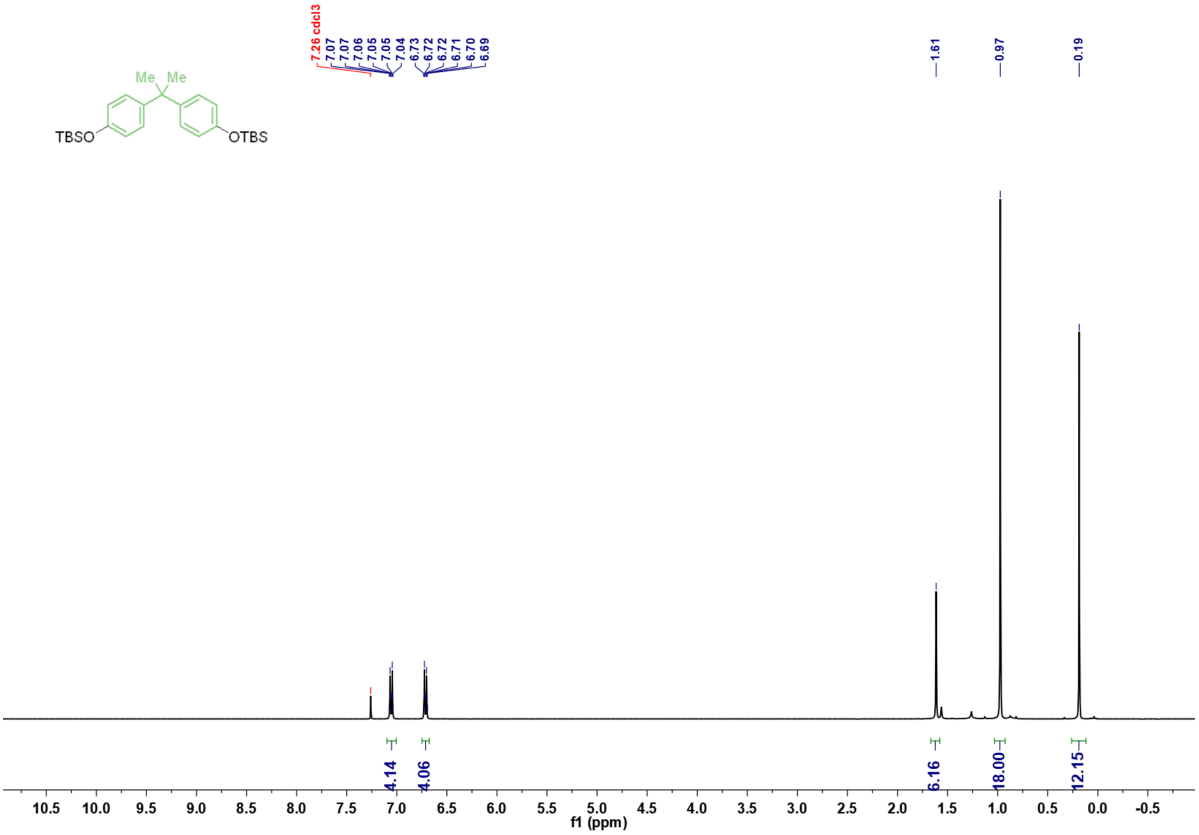

^{1}H NMR (400 MHz, $CDCl_3$) of **52**

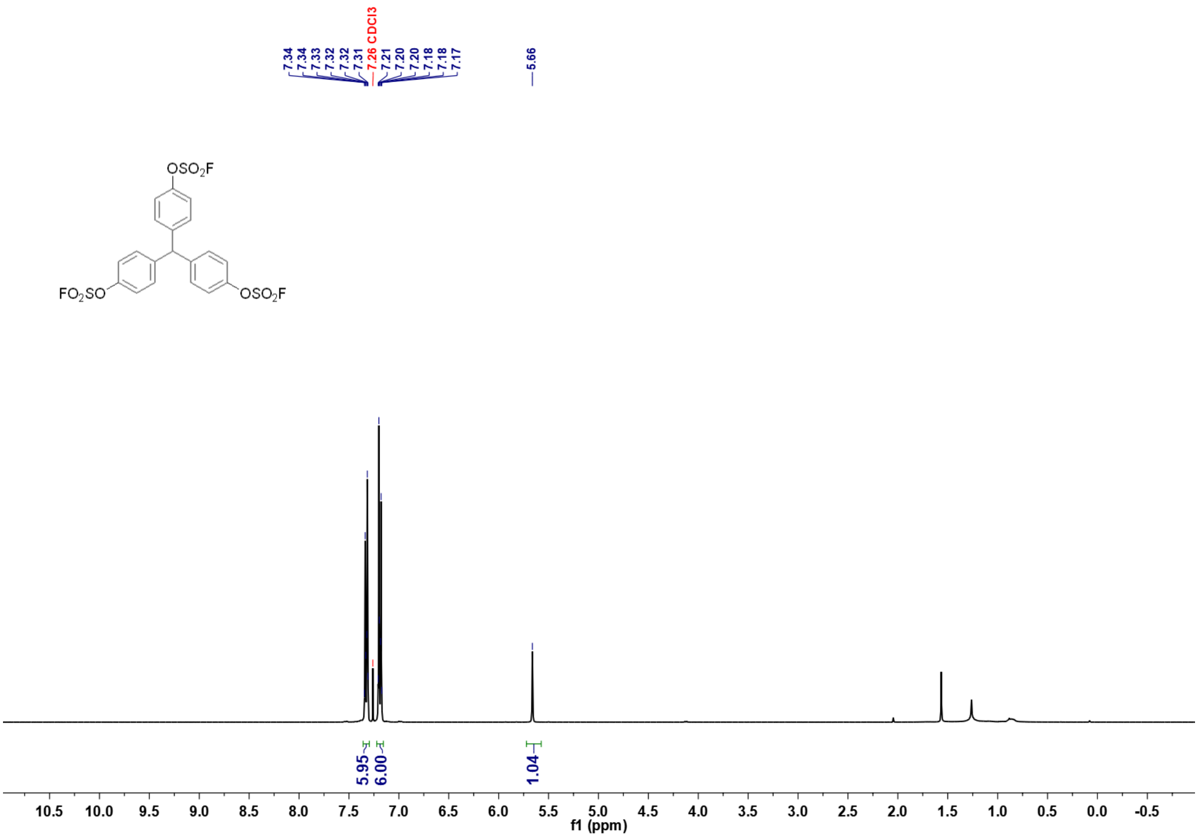

^{13}C NMR (101 MHz, $CDCl_3$) of **52**

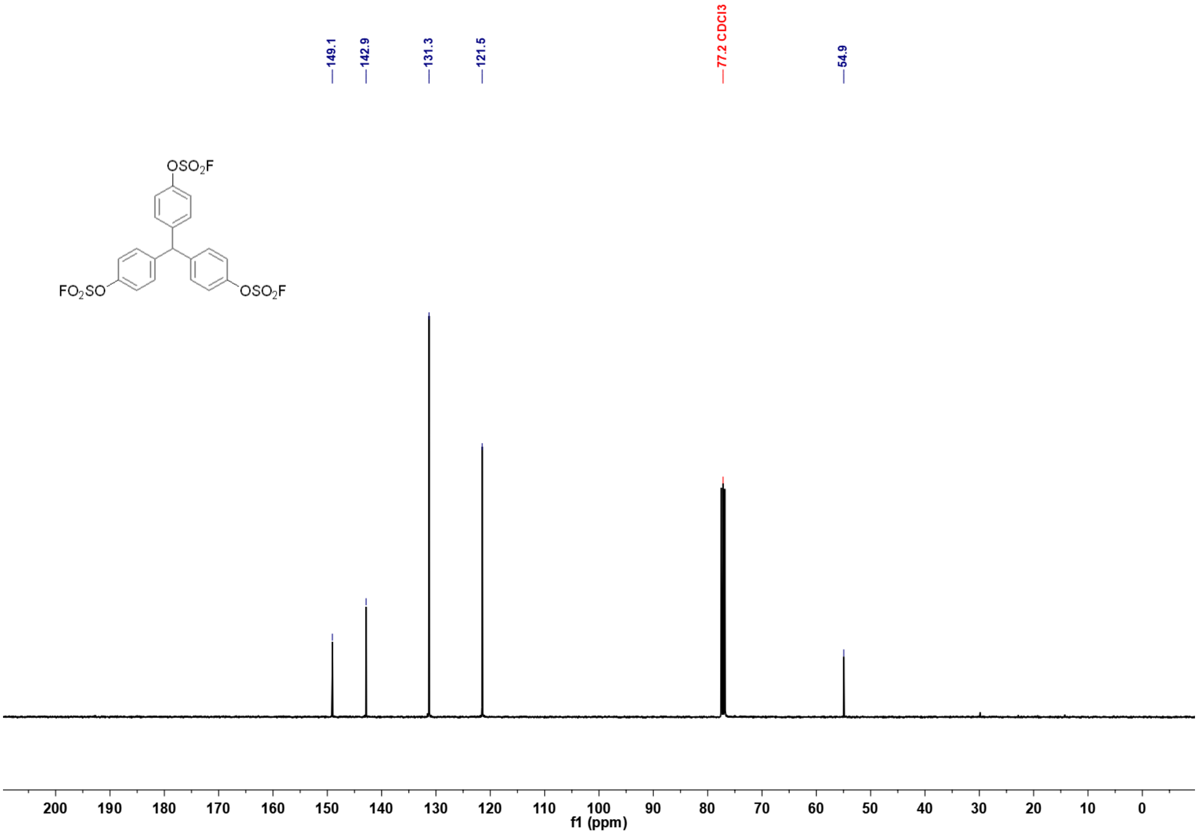

^{19}F NMR (376 MHz, $CDCl_3$) of **52**

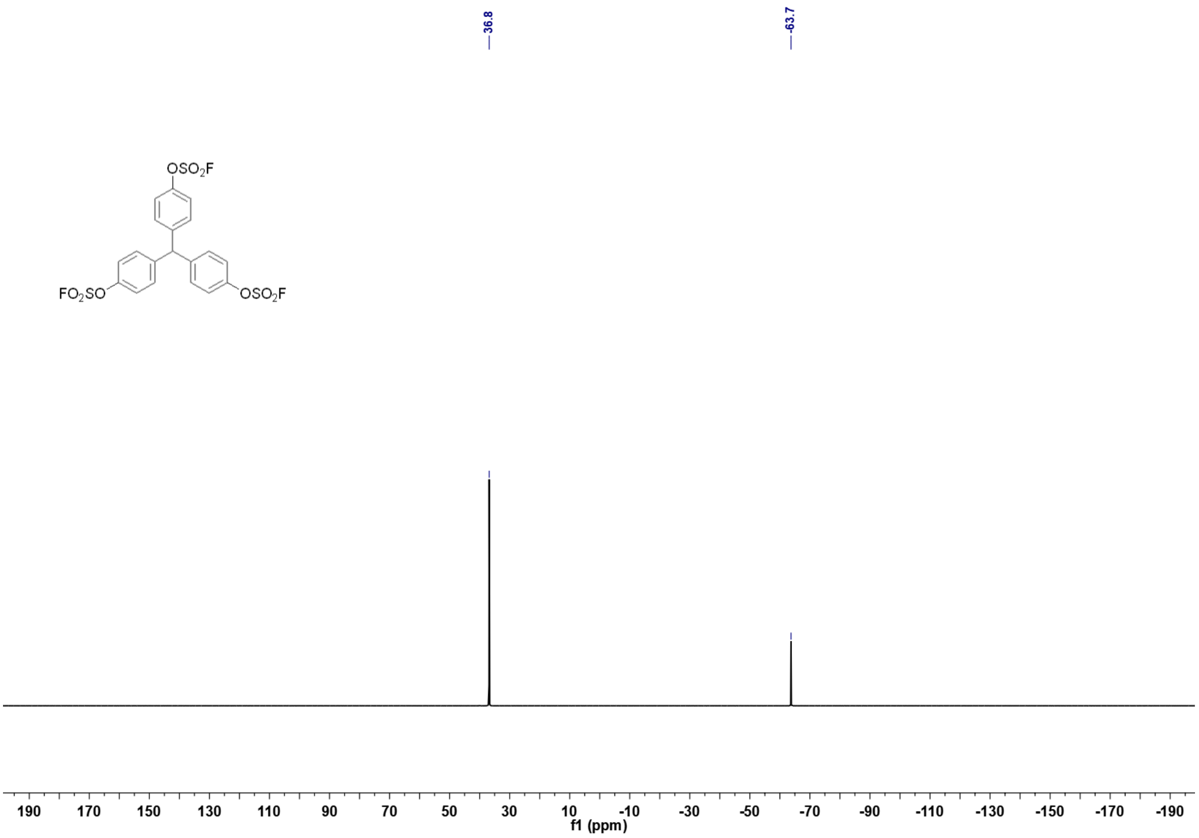

^{1}H NMR (400 MHz, DMSO-d_6) of **53**

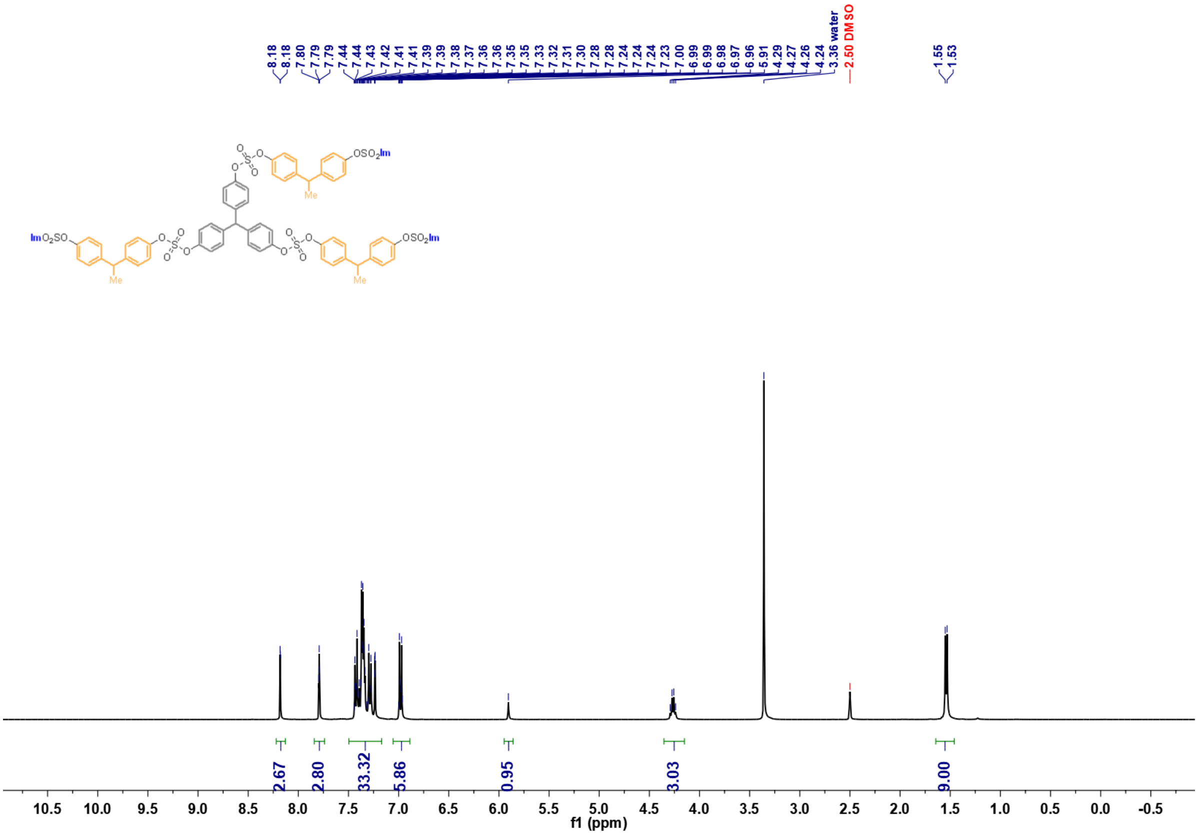

^{13}C NMR (101 MHz, DMSO-d_6) of **53**

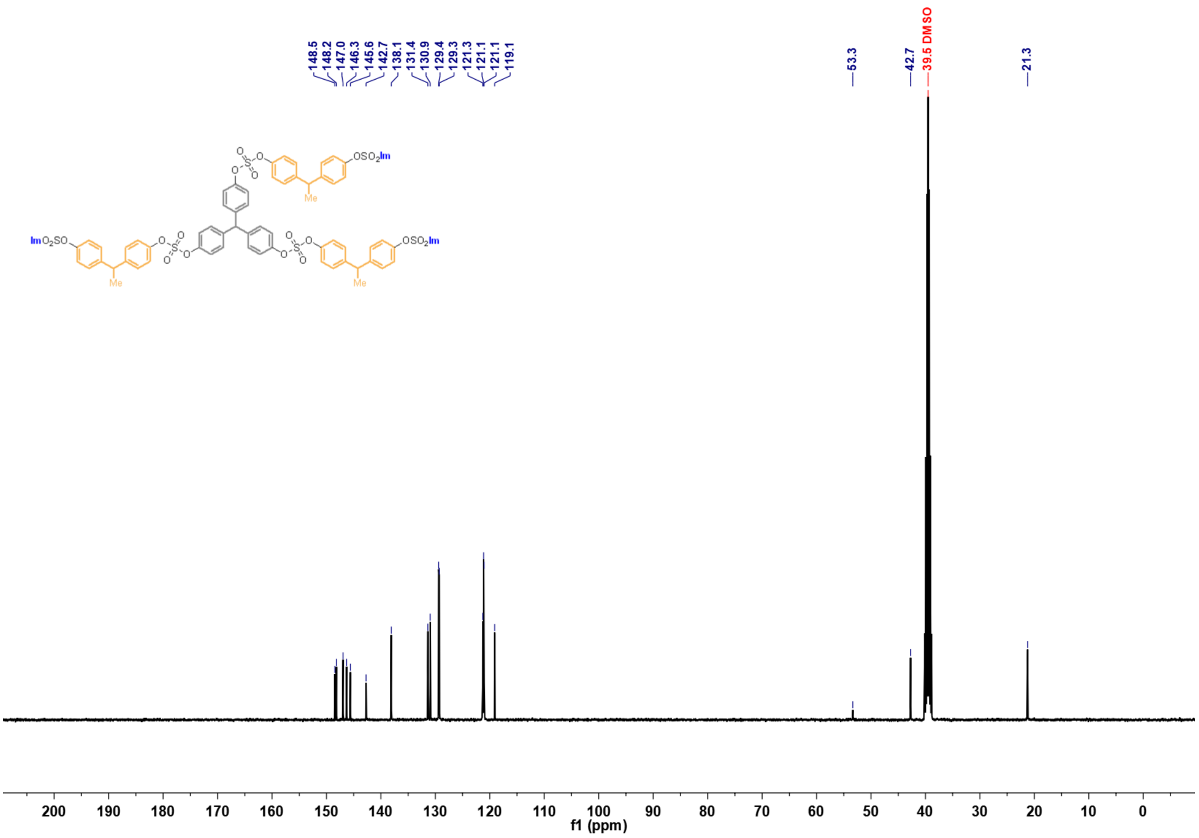

^{1}H NMR (400 MHz, DMSO-d_6) of **54**

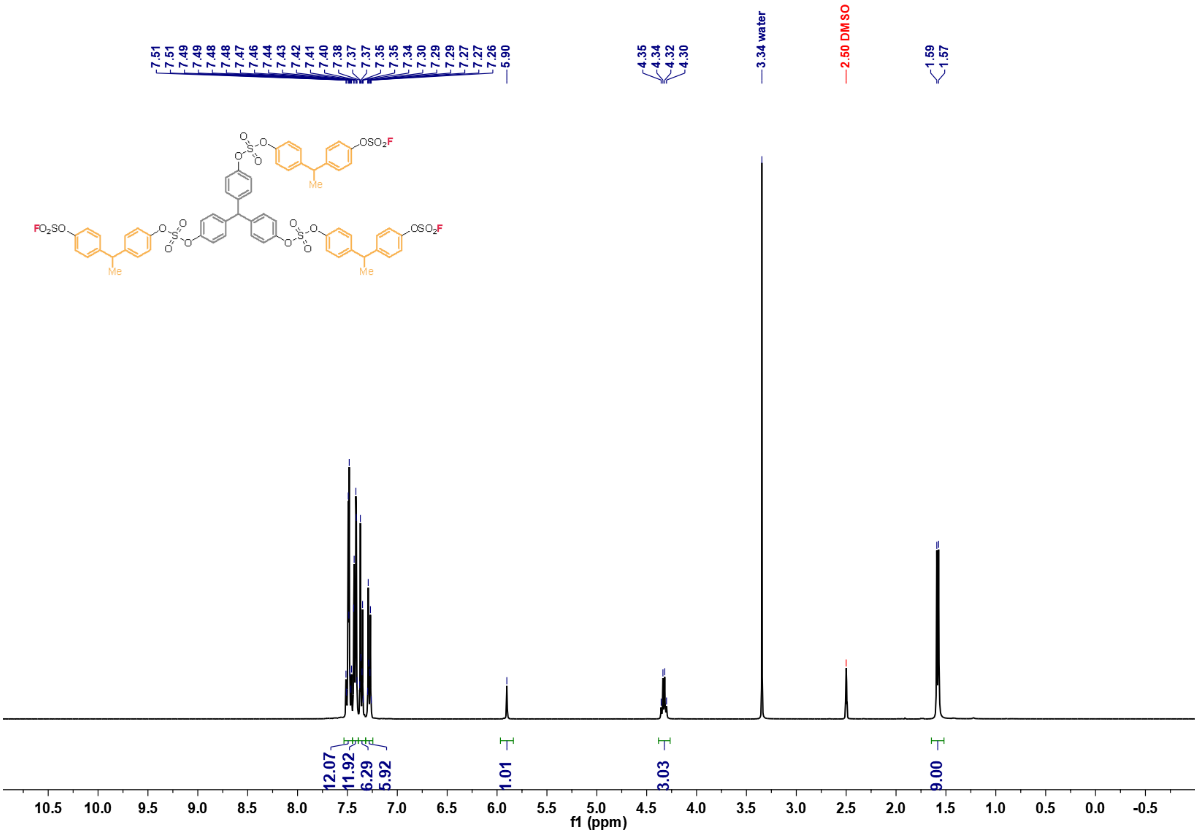

^{13}C NMR (101 MHz, DMSO-d_6) of **54**

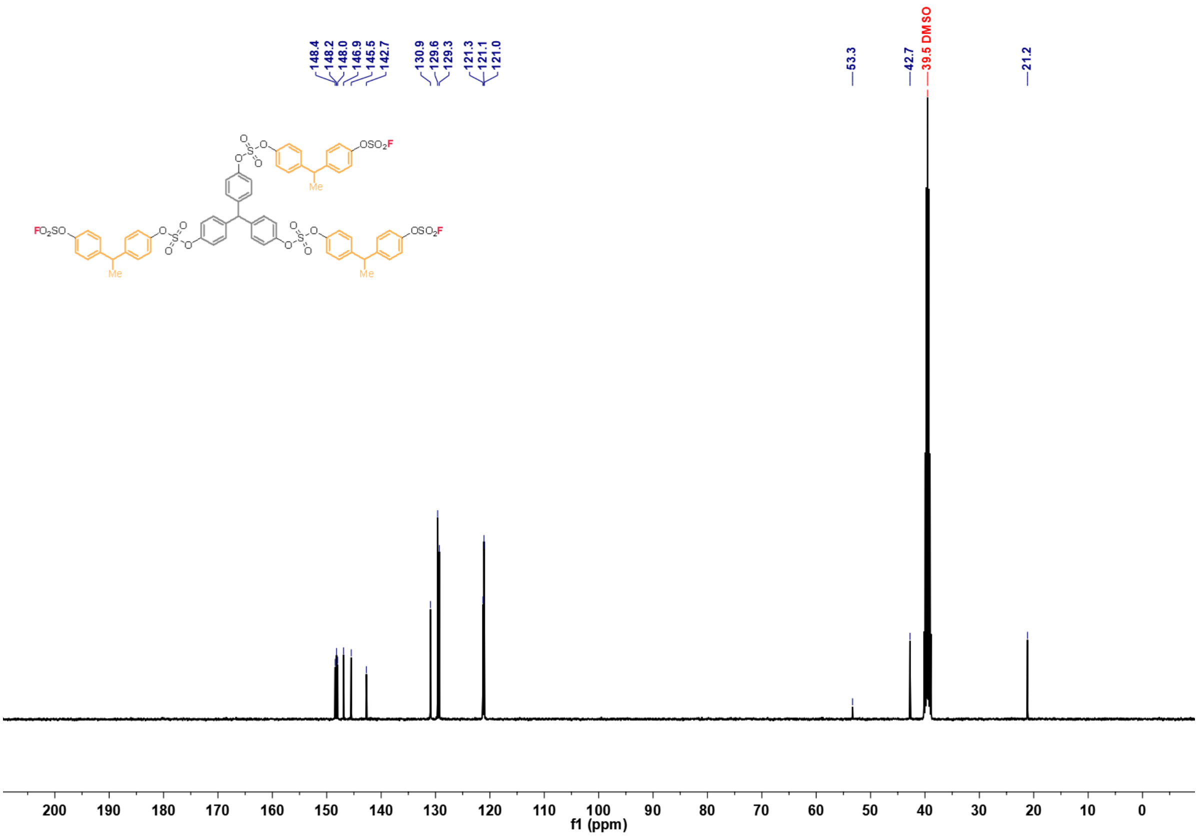

^{19}F NMR (376 MHz, DMSO-d_6) of **54**

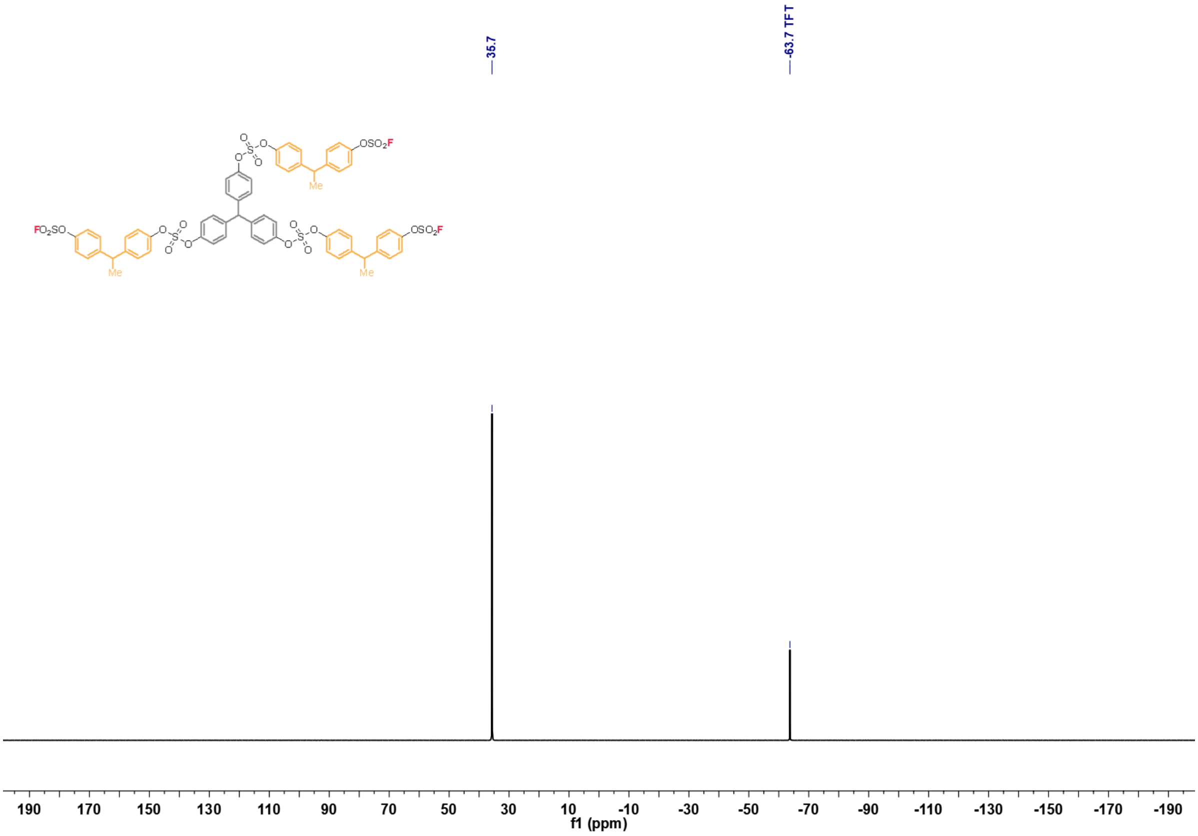

^{1}H NMR (400 MHz, $CDCl_3$) of **55**

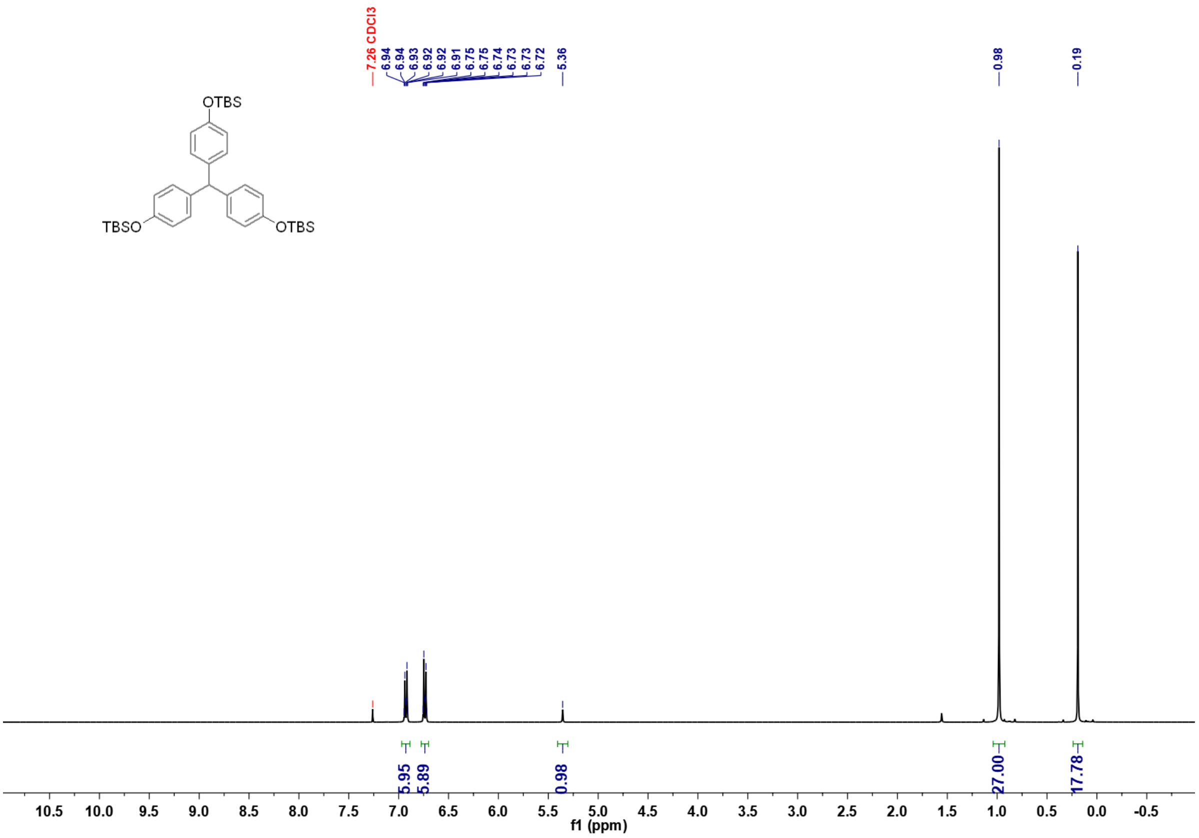

^{13}C NMR (101 MHz, $CDCl_3$) of **55**

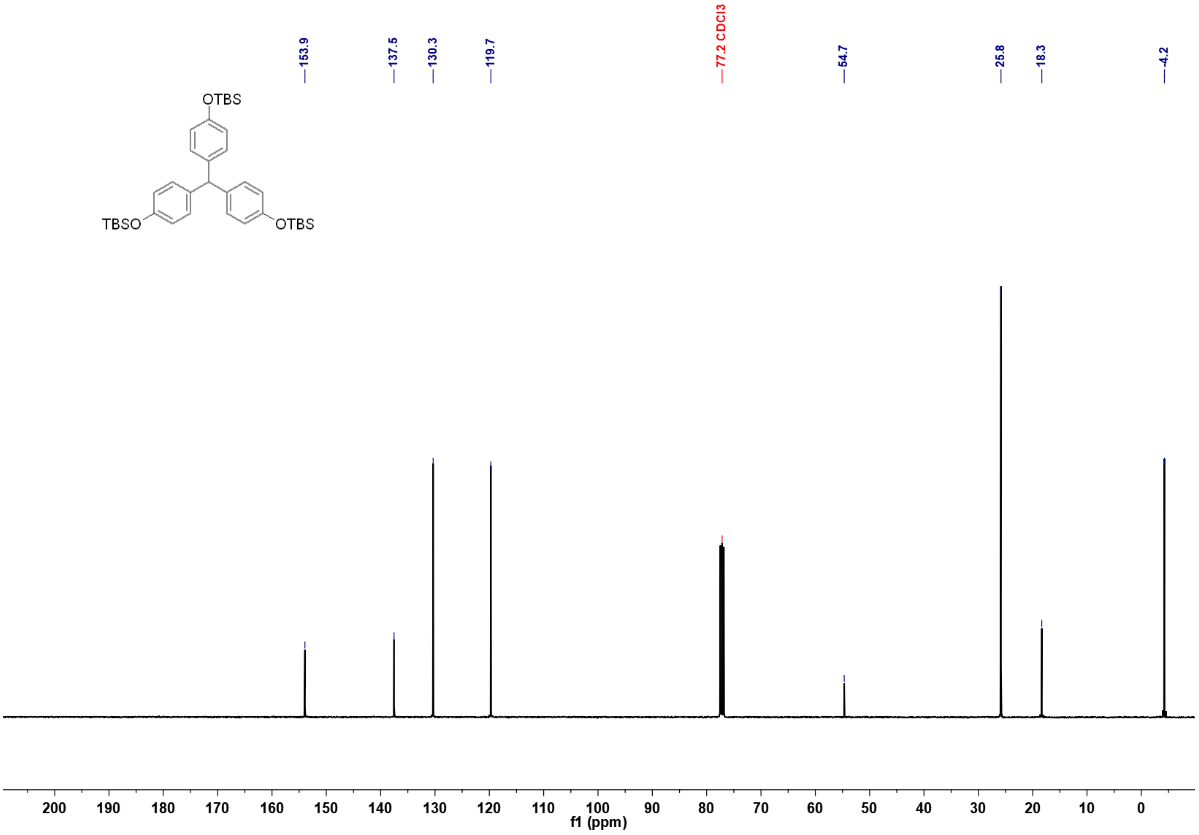

^{1}H NMR (400 MHz, CD_2Cl_2) of **P-1**

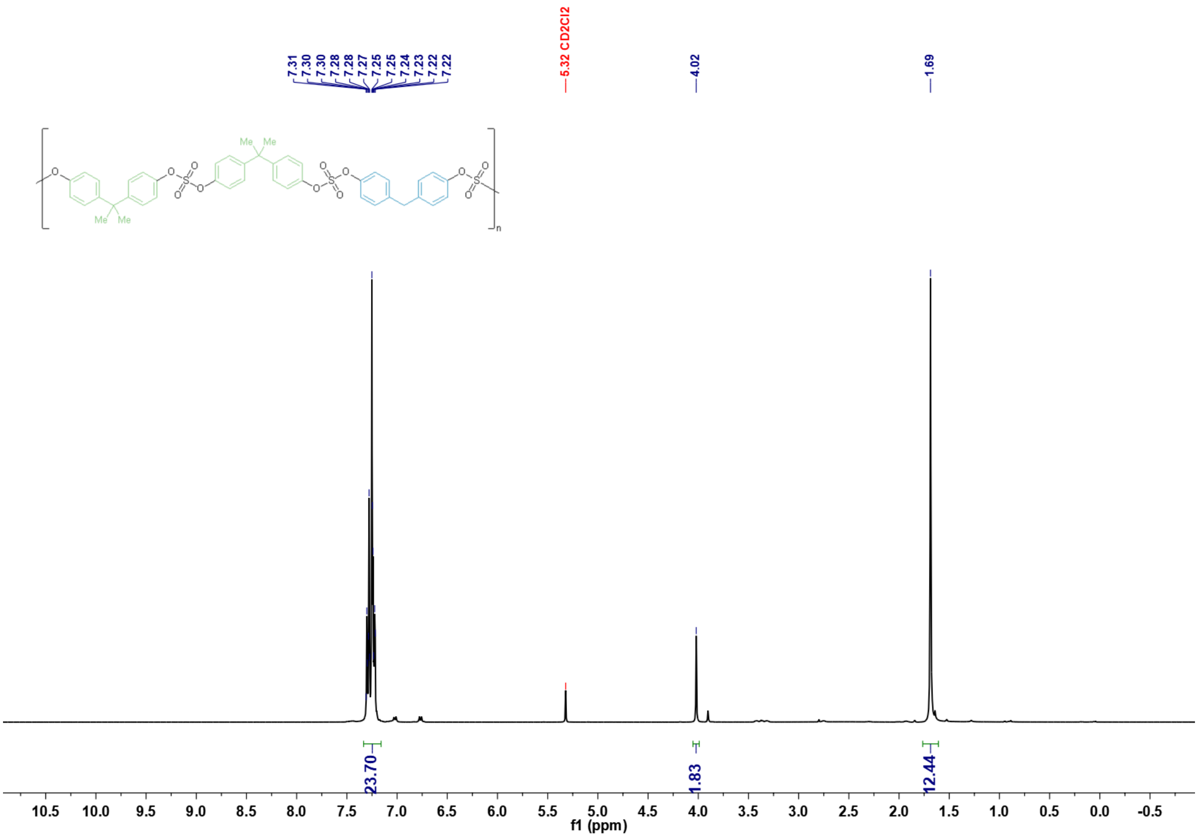

^{13}C NMR (101 MHz, CD_2Cl_2) of **P-1**

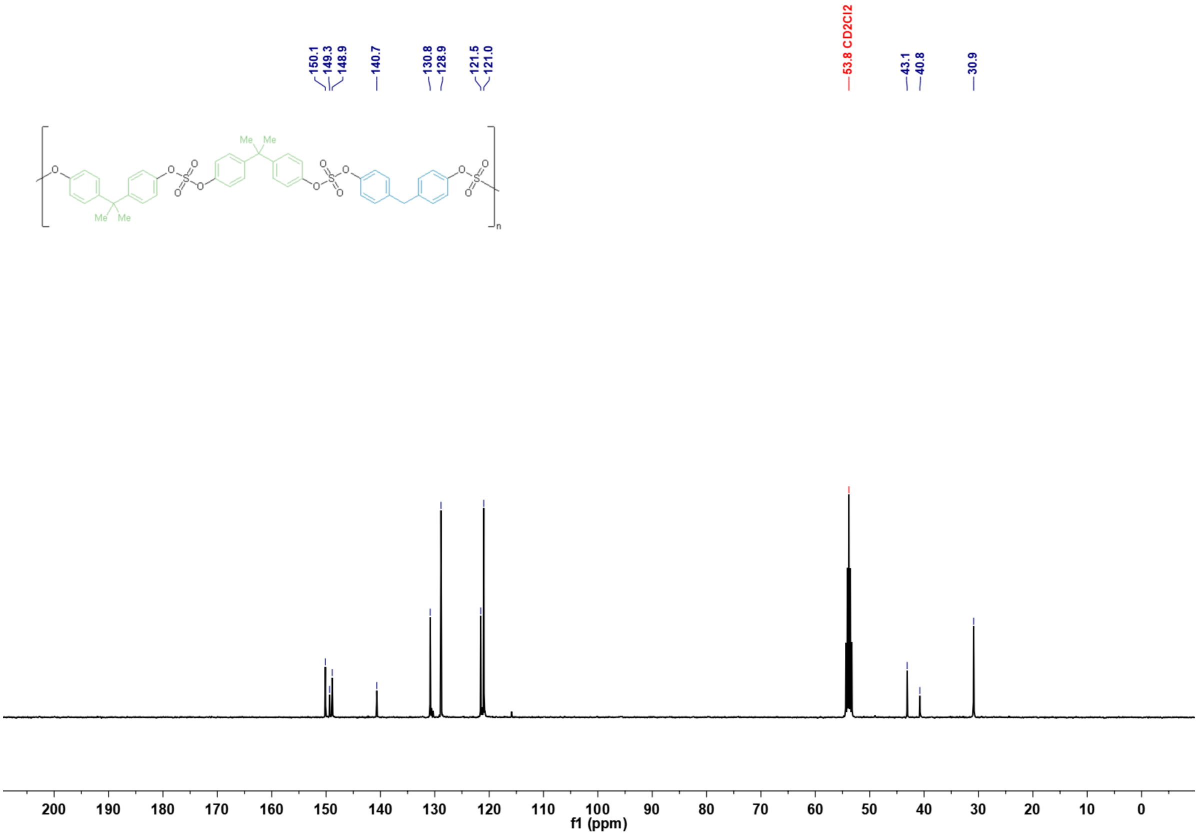

^{1}H NMR (400 MHz, CD_2Cl_2) of **P-2**

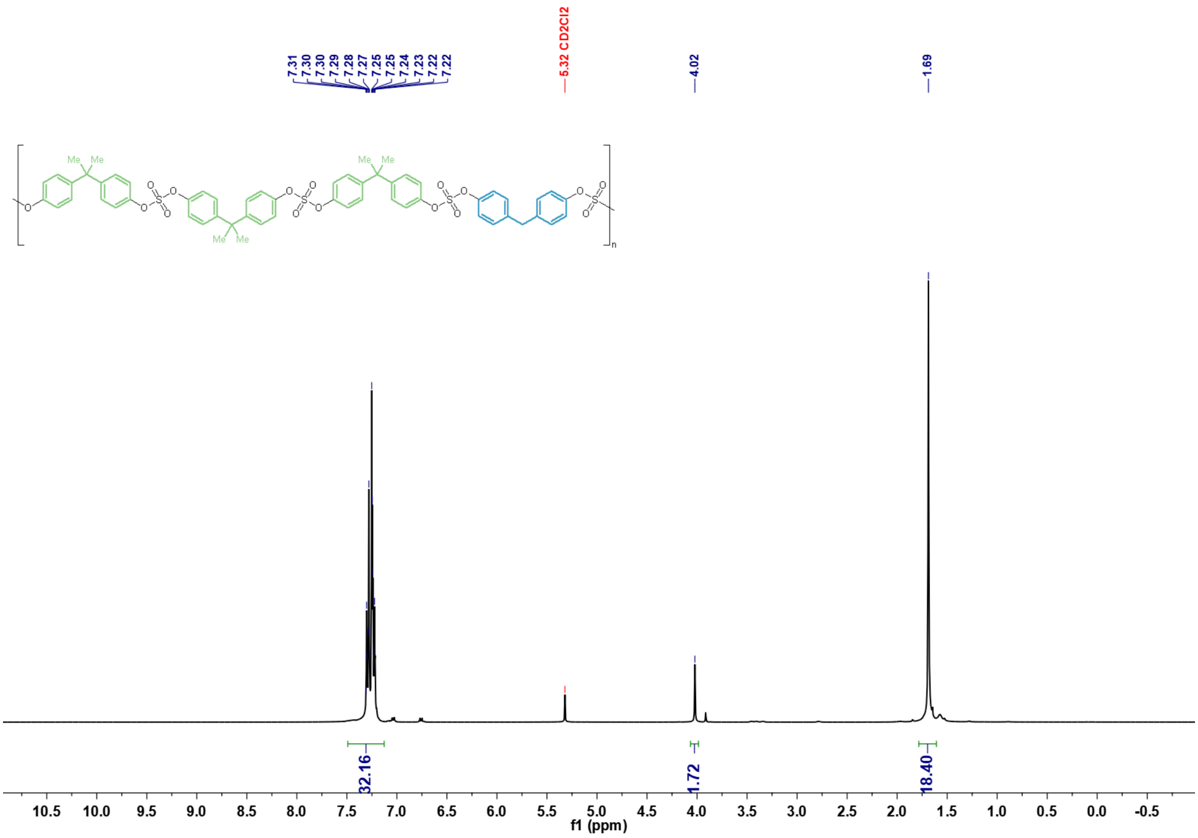

^{13}C NMR (101 MHz, CD_2Cl_2) of **P-2**

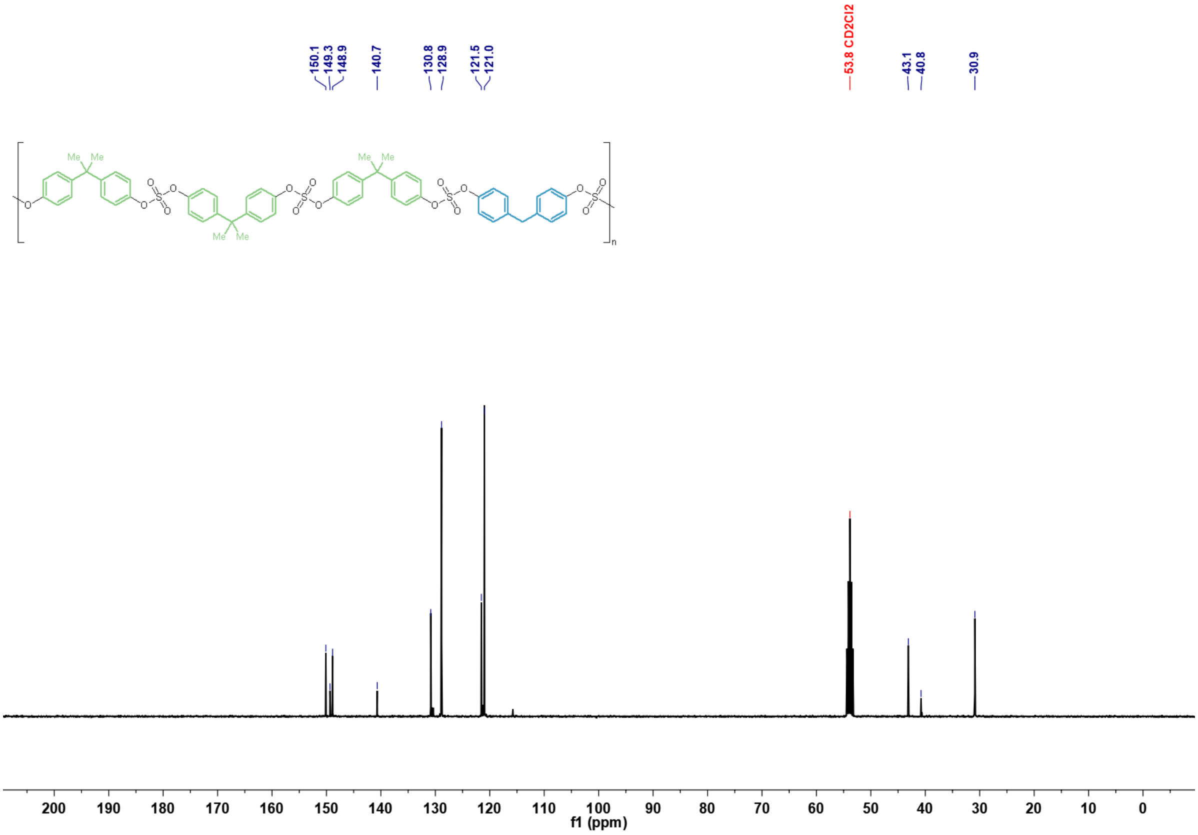

^{1}H NMR (400 MHz, CD_2Cl_2) of **P-3**

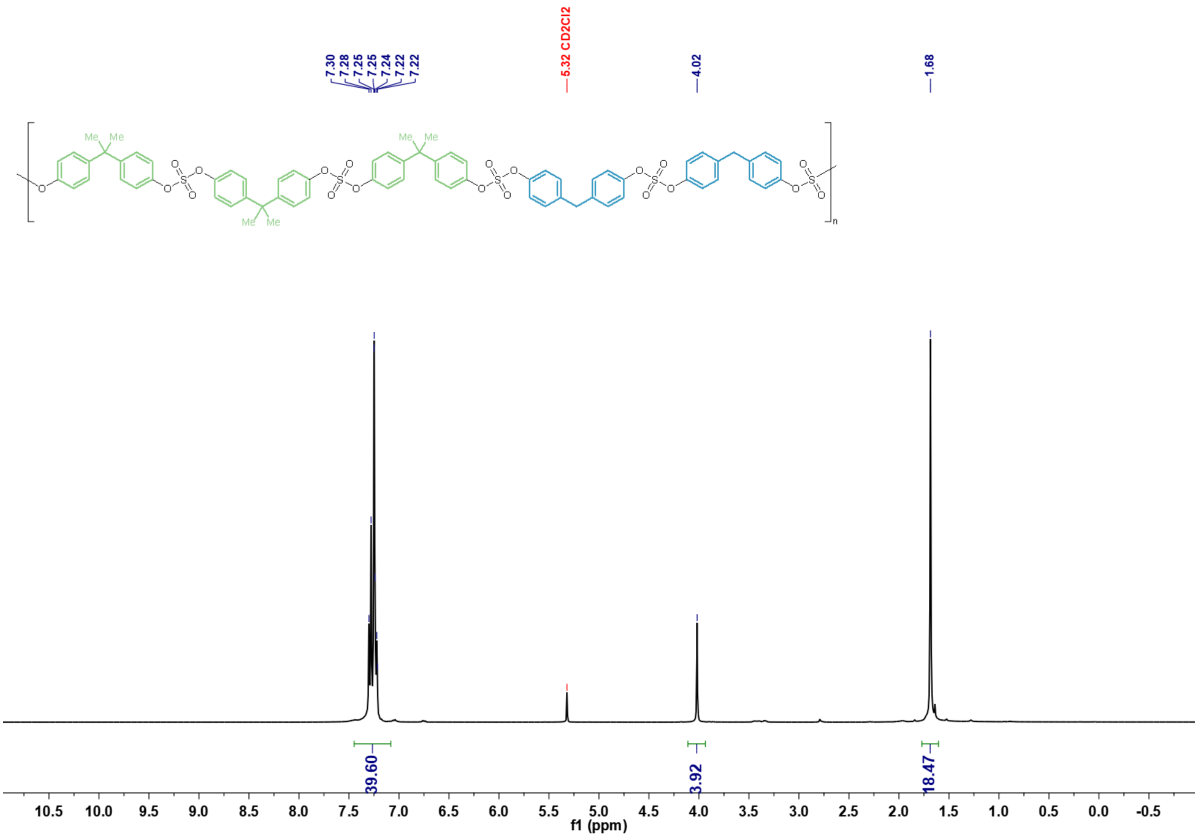

^{13}C NMR (101 MHz, CD_2Cl_2) of **P-3**

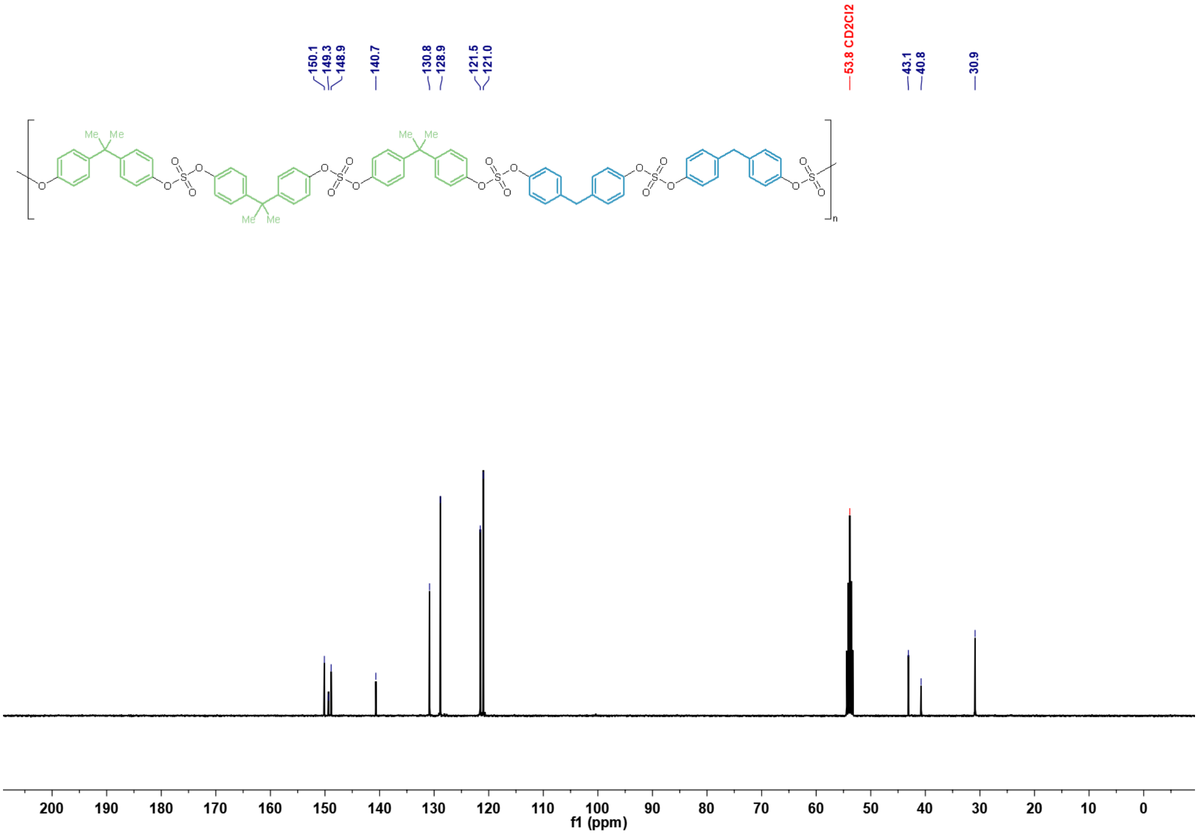

^{1}H NMR (400 MHz, $CDCl_3$) of **5'**

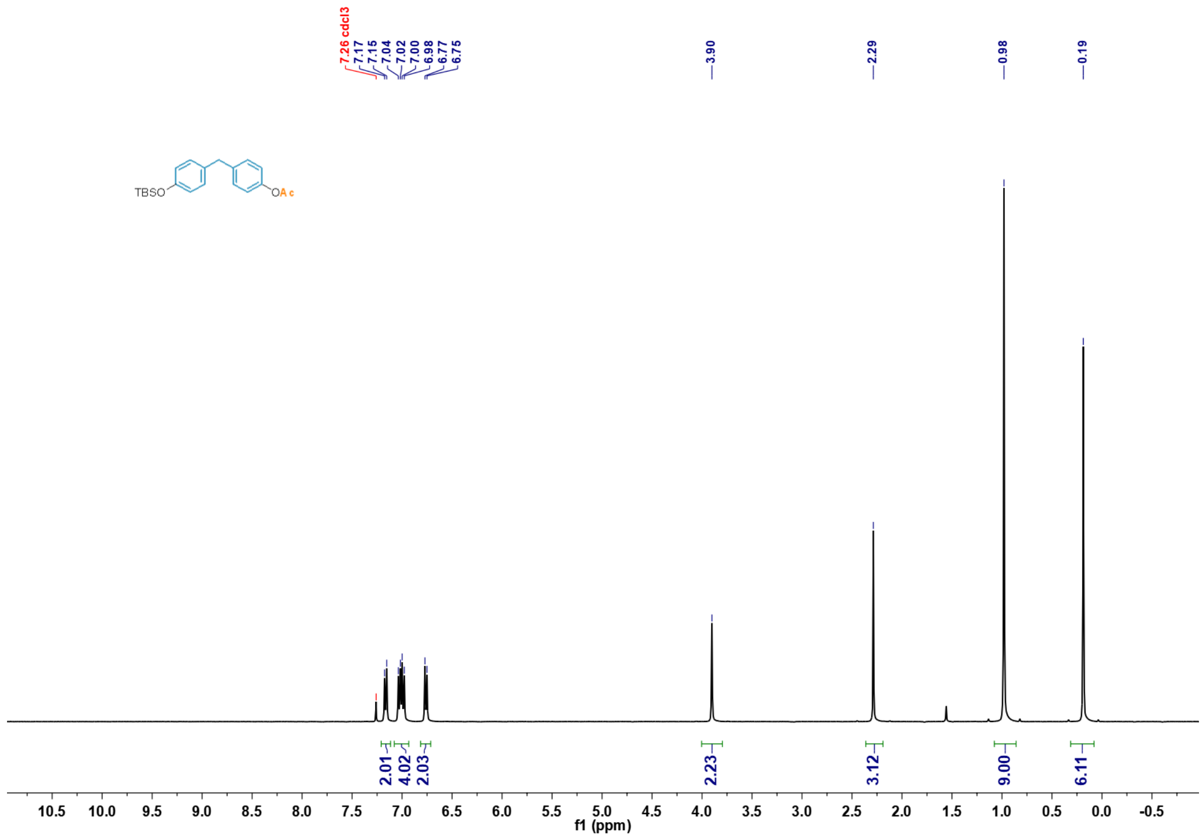

^{13}C NMR (101 MHz, $CDCl_3$) of **5'**

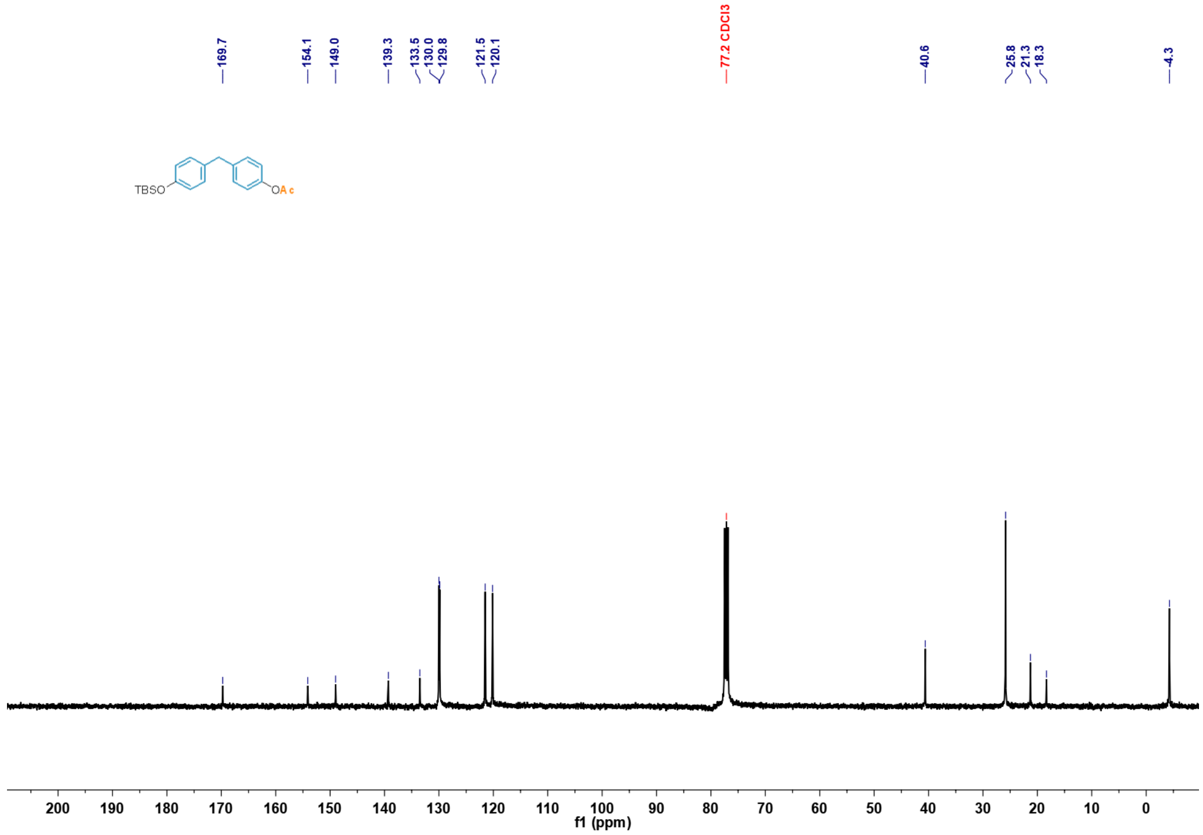

^{1}H NMR (400 MHz, $CDCl_3$) of **8'**

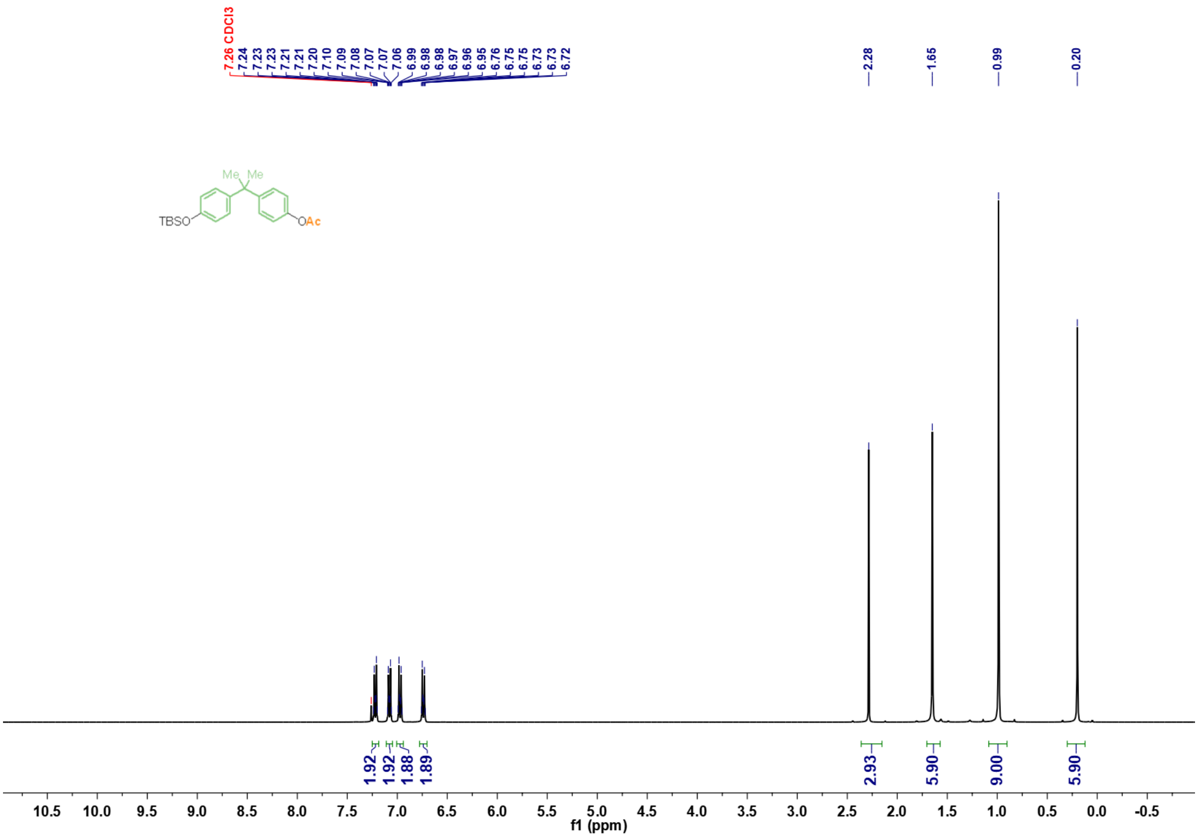

^{13}C NMR (101 MHz, $CDCl_3$) of **8'**

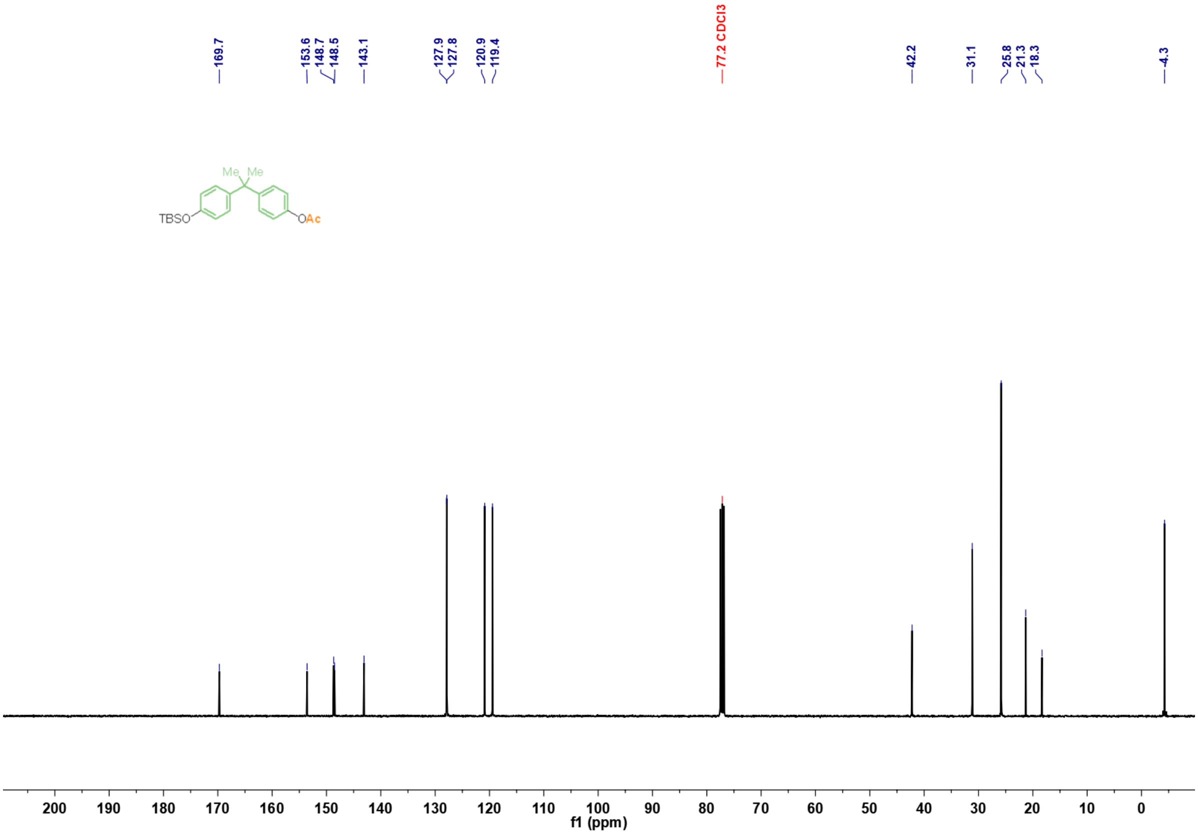

^{1}H NMR (400 MHz, Acetone-d_6) of **19'**

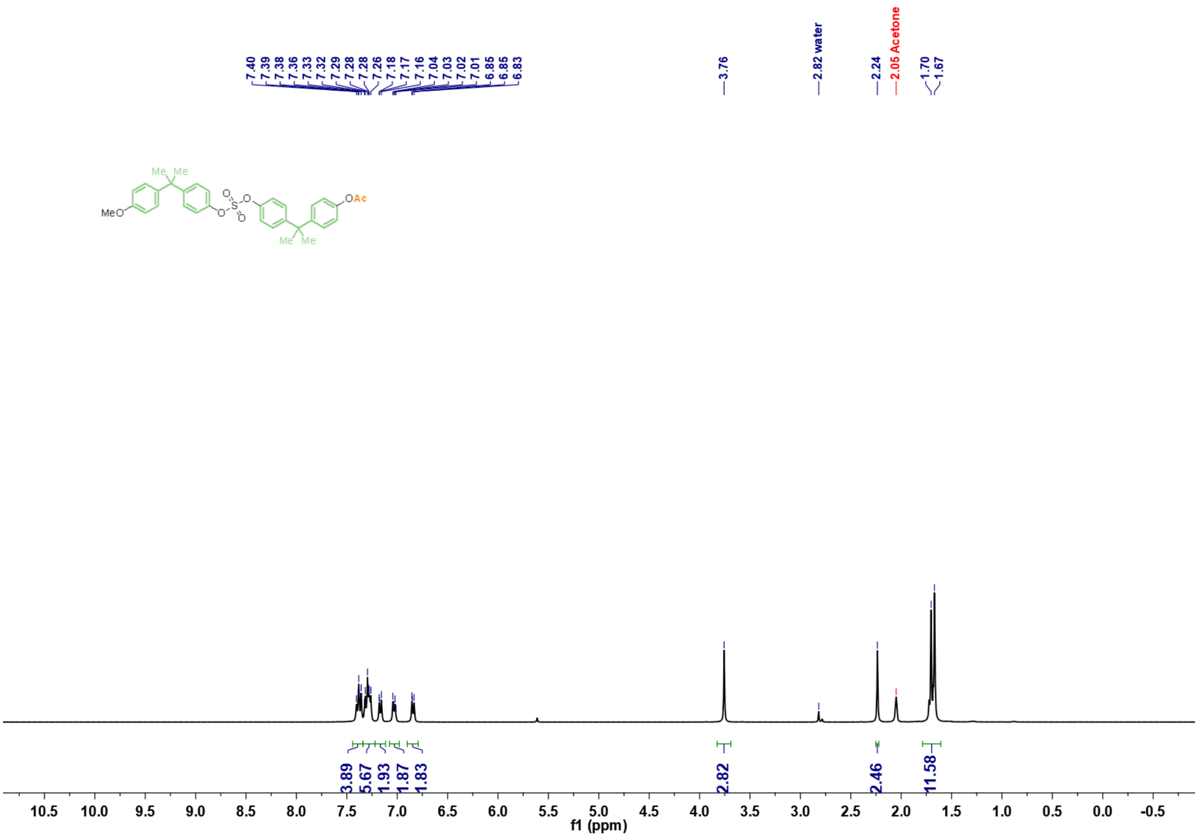

^{13}C NMR (101 MHz, Acetone-d_6) of **19'**

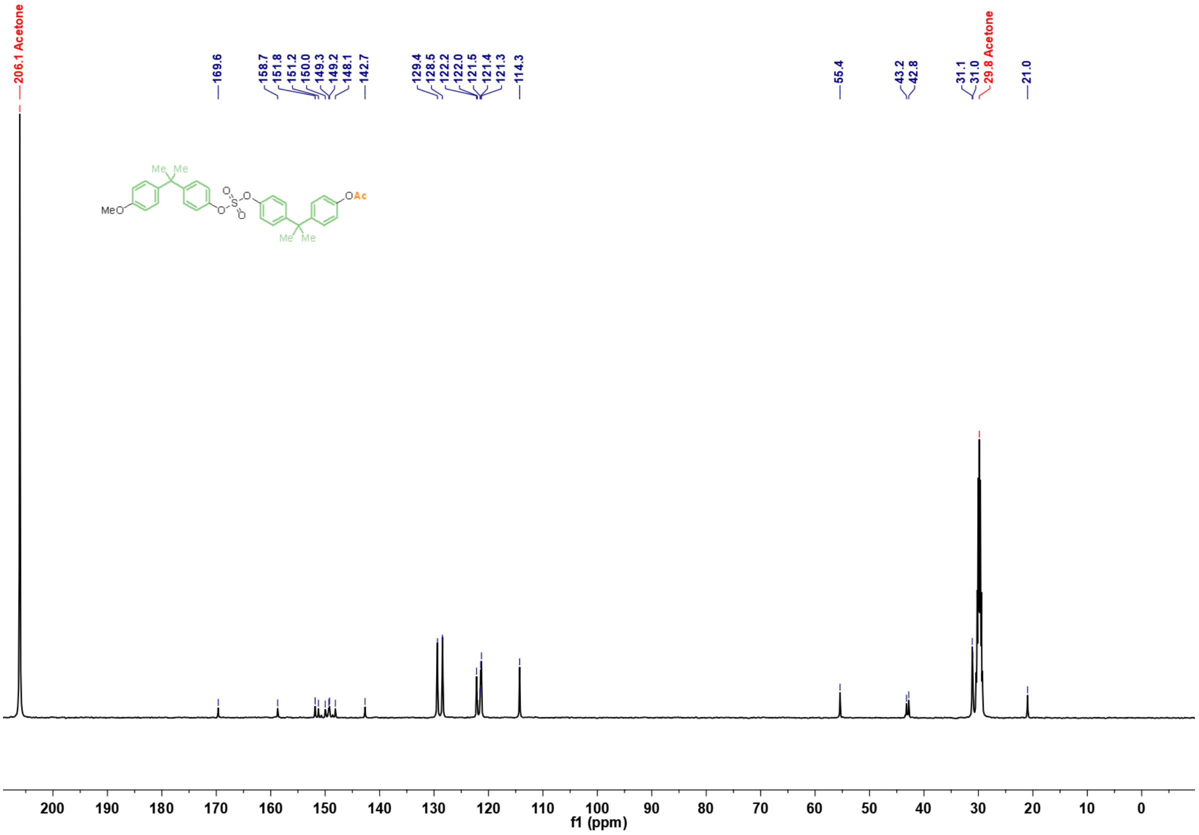

^{1}H NMR (400 MHz, Acetone-d_6) of **19''**

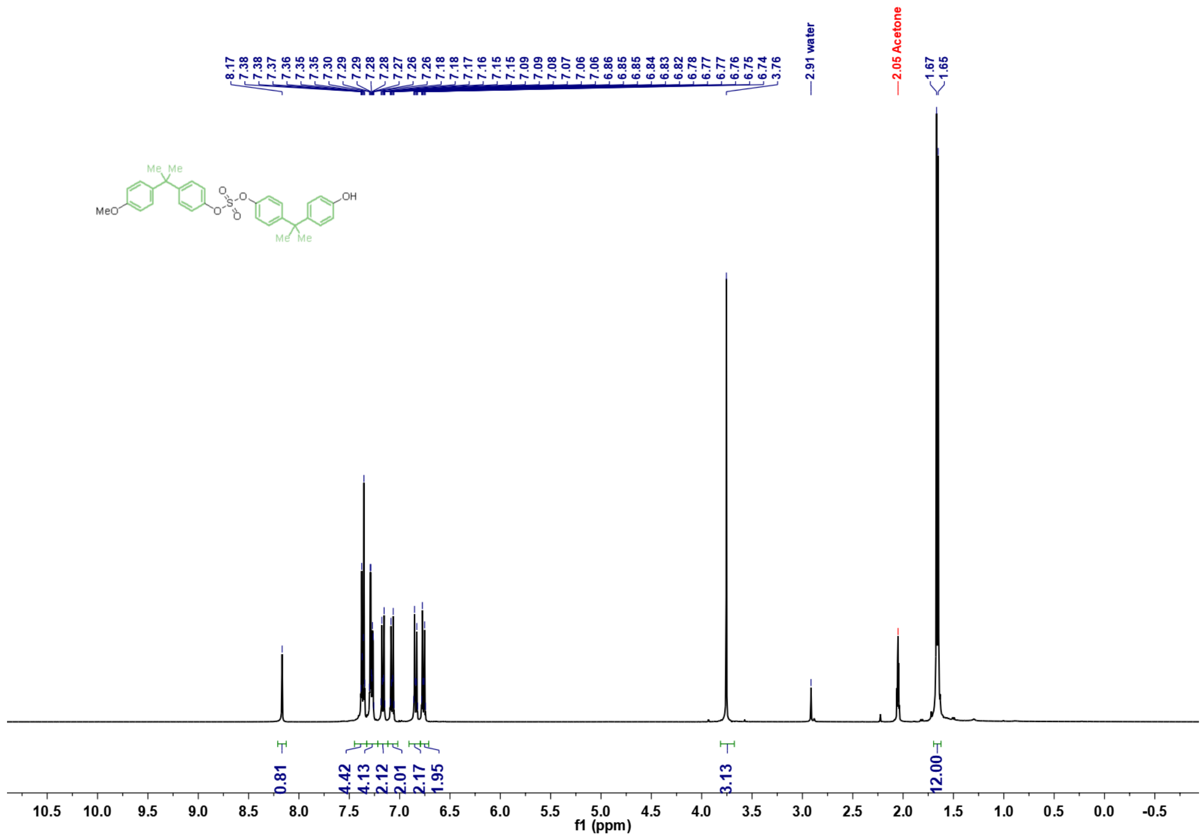

^{13}C NMR (101 MHz, Acetone-d_6) of **19''**

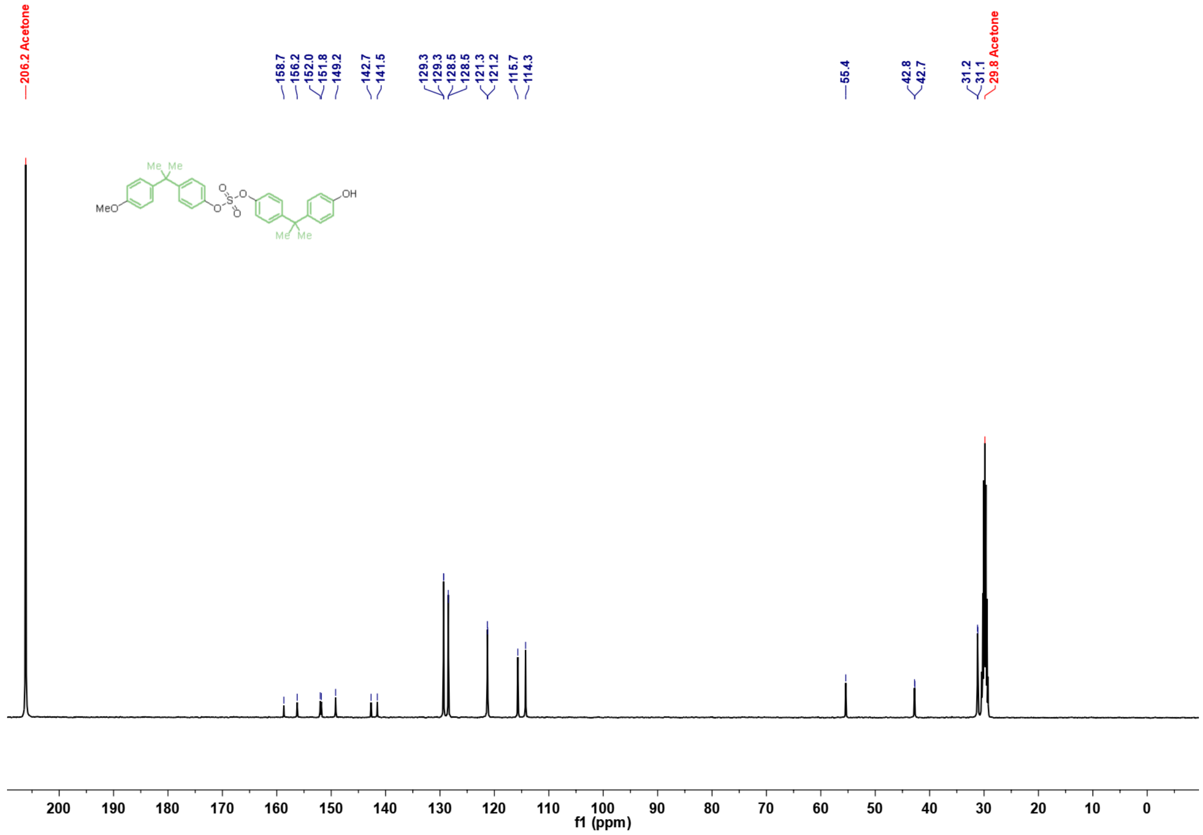

^{1}H NMR (400 MHz, Acetone-d_6) of **21'**

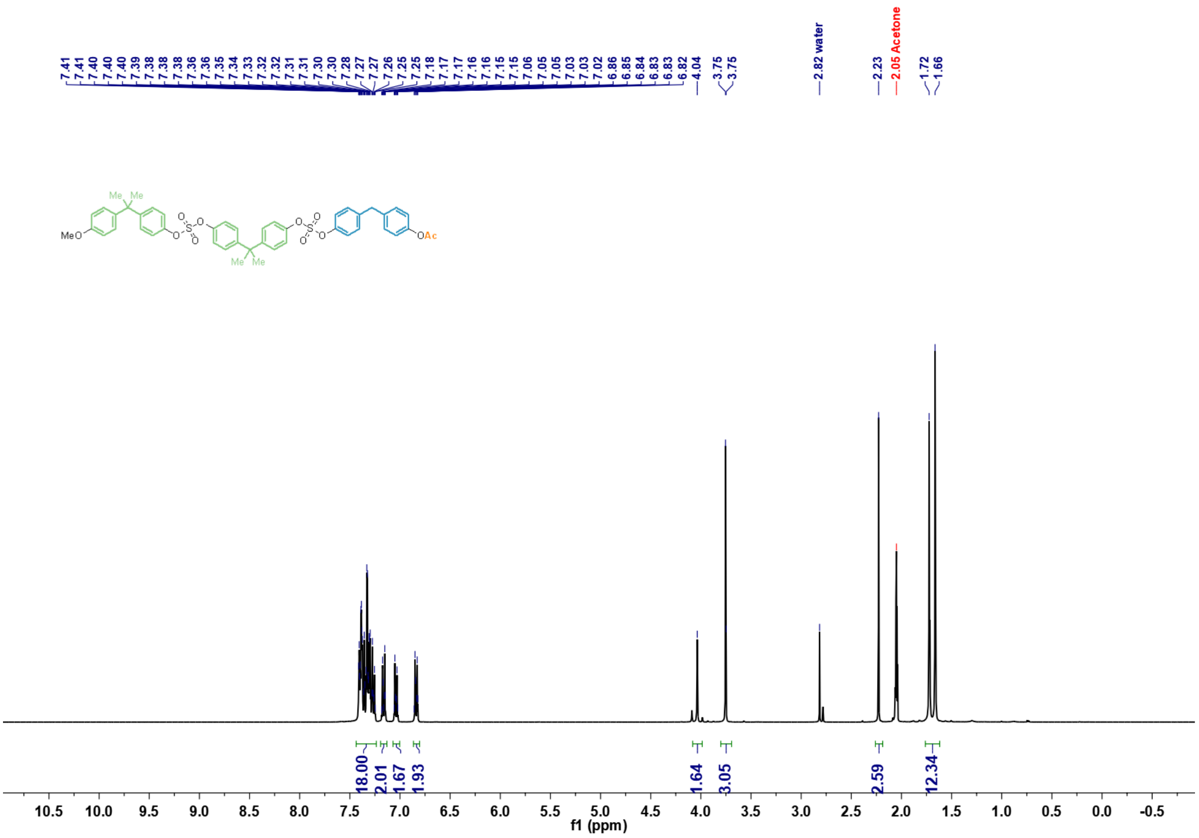

^{13}C NMR (101 MHz, Acetone-d_6) of **21'**

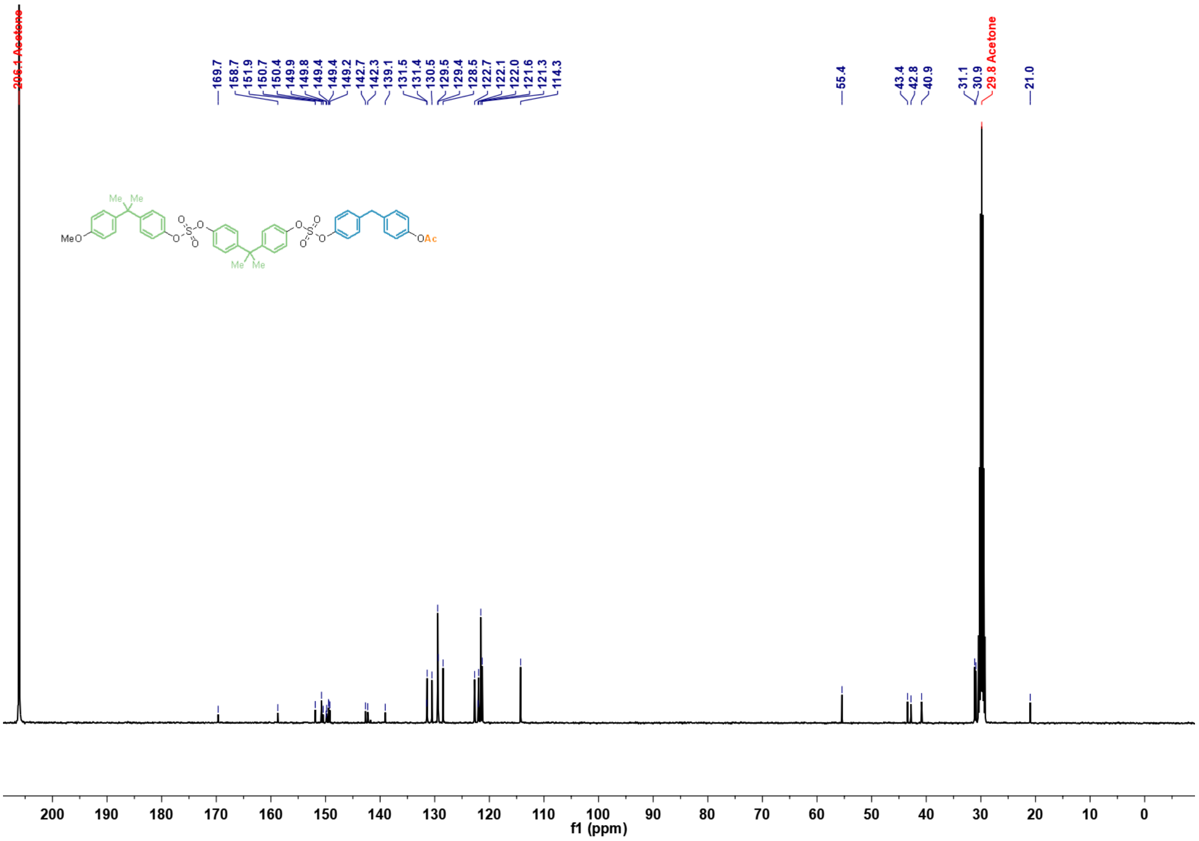

^{1}H NMR (400 MHz, Acetone-d_6) of **21''**

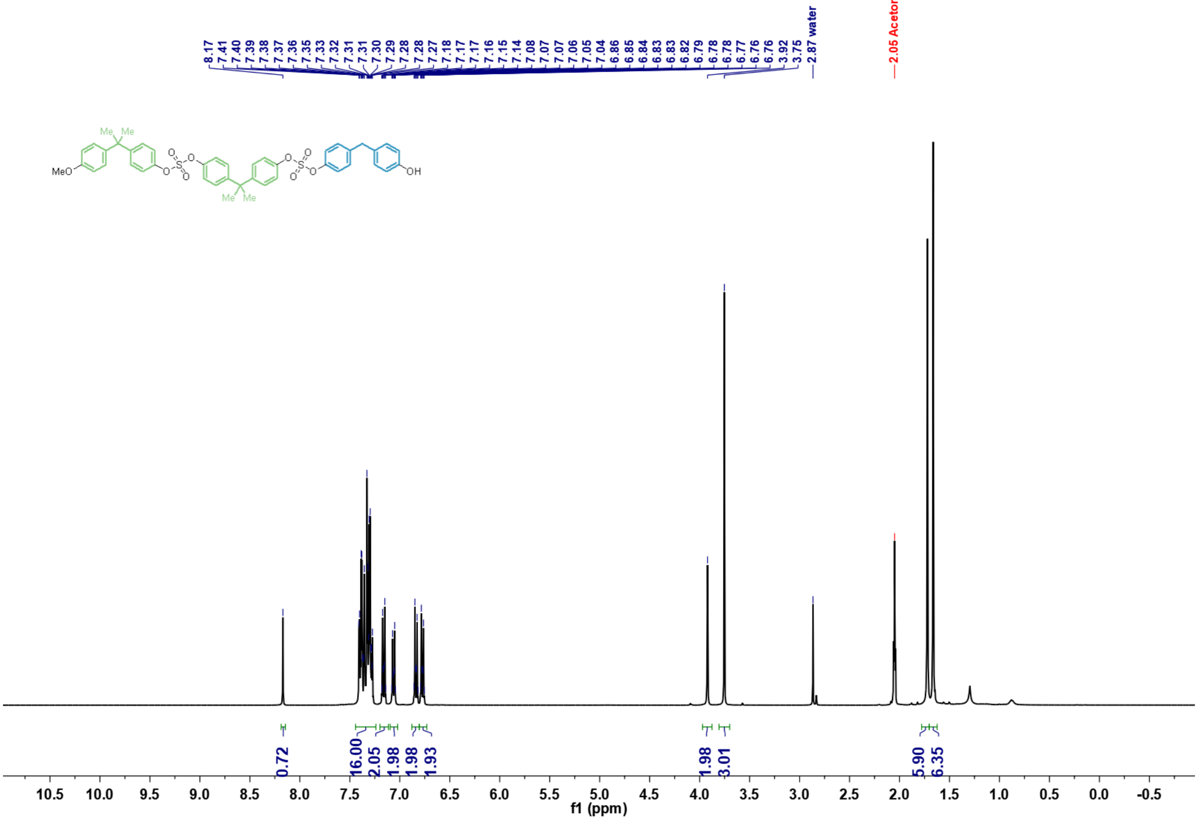

^{13}C NMR (101 MHz, Acetone-d_6) of **21''**

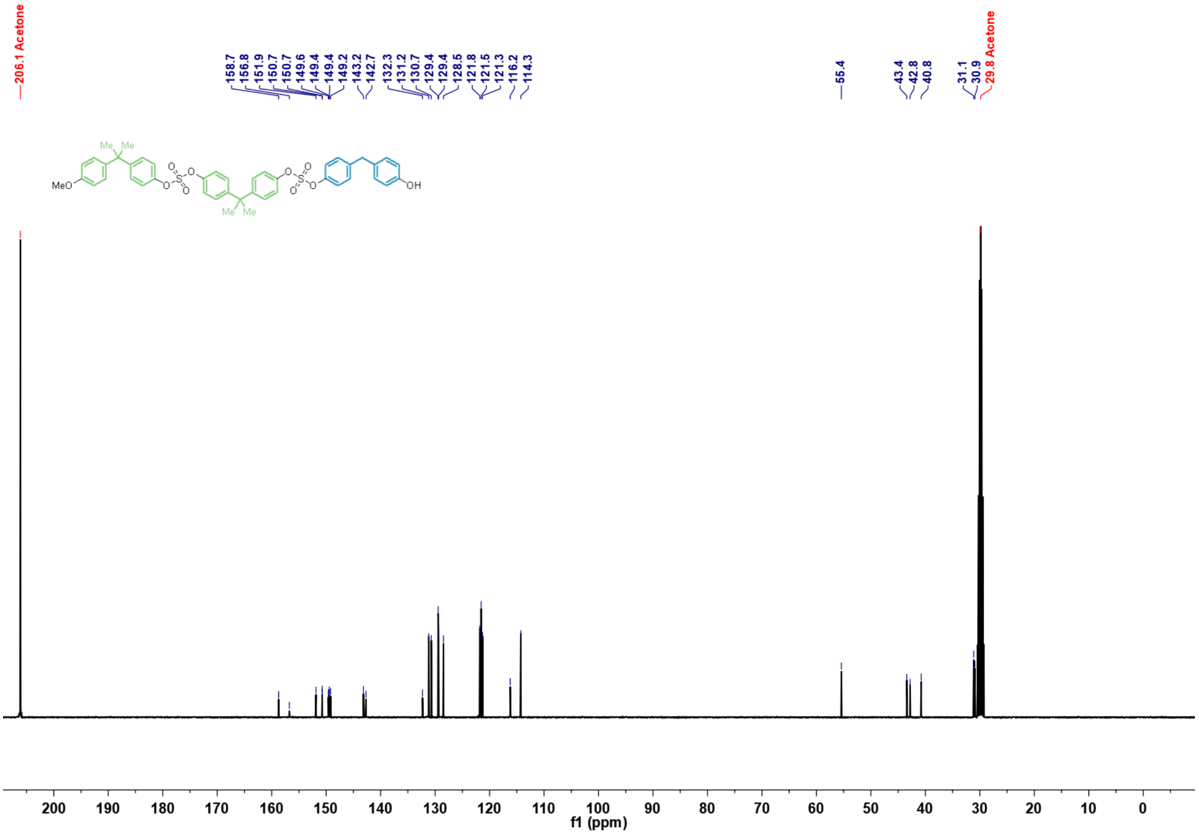

^{1}H NMR (400 MHz, DMSO-d_6) of **Side P**

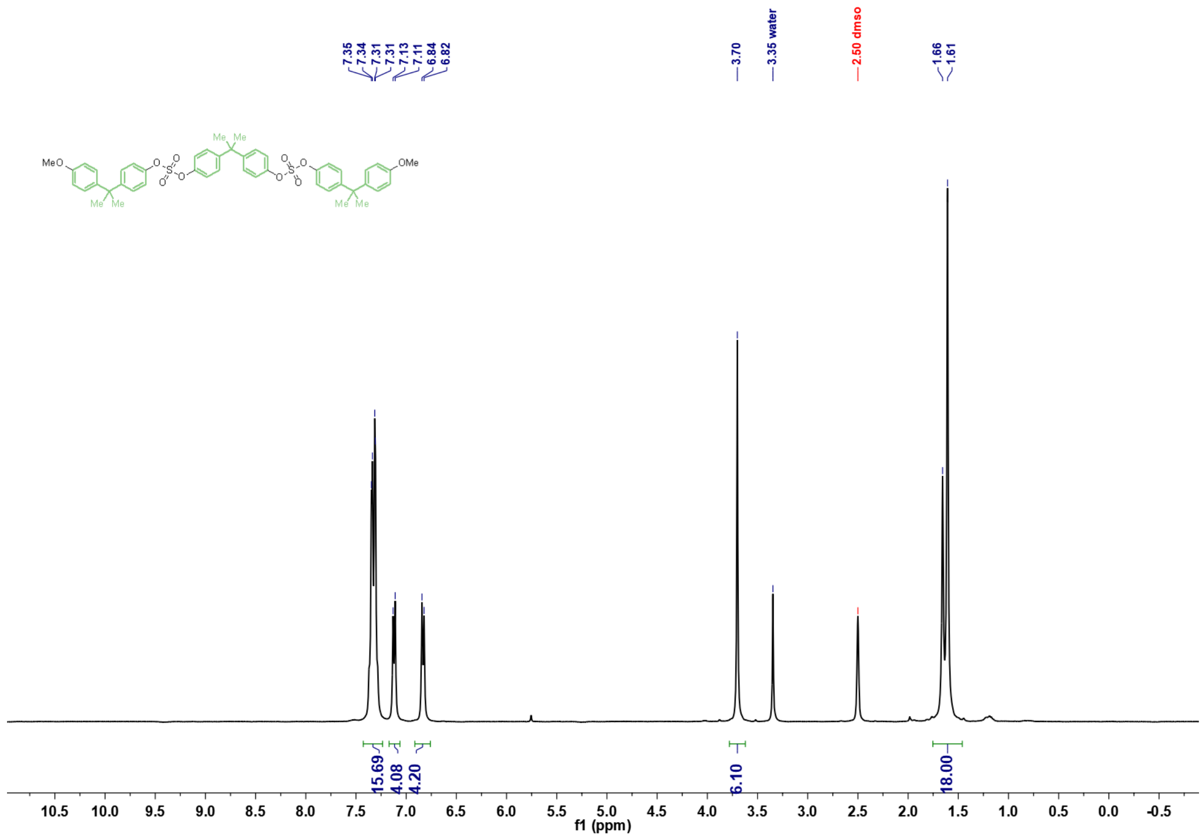

^{13}C NMR (101 MHz, DMSO-d_6) of **Side P**

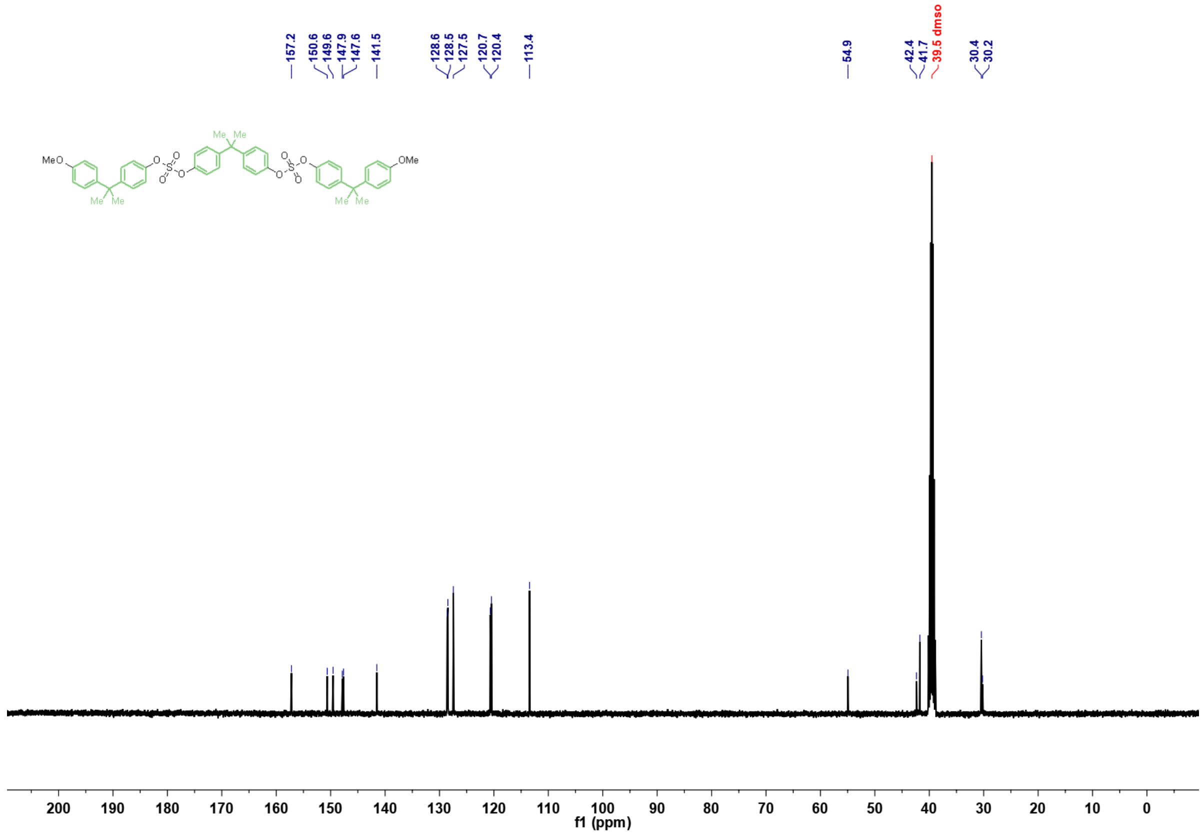

11. References

(1) Hanada, S., Yuasa, A., Kuroiwa, H., Motoyama, Y. & Nagashima, H. Hydrosilanes are not always reducing agents for carbonyl compounds, II: Ruthenium-catalyzed deprotection of *tert*-butyl groups in carbamates, carbonates, esters, and ethers. *Eur. J. Org. Chem.* 1021–1025 (2010).

(2) Wang, Z. et al. Organocatalytic asymmetric synthesis of 1,1-diarylethanes by transfer hydrogenation. *J. Am. Chem. Soc.* **137**, 383–389 (2015).

(3) de Bruyn, P. J., Foo, L. M., Lim, A. S. C., Looney, M. G. & Solomon, D. H. The chemistry of novolac resins. part 4. the strategic synthesis of model compounds. *Tetrahedron* **53**, 13915–13932 (1997).

(4) Jia, X. G., Pornsuriyasak, P. & Demchenko, A. V. Templated oligosaccharide synthesis: driving forces and mechanistic aspects. *J. Org. Chem.* **81**, 12232–12246 (2016).

(5) Kwon, Y.-D. et al. Synthesis of ^{18}F-labeled aryl fluorosulfates via nucleophilic radiofluorination. *Org. Lett.* **23**, 2766–2771 (2021).

(6) (a) Dong, J., Sharpless, K. B., Kwisnek, L., Oakdale, J. S. & Fokin, V. V. SuFEx-based synthesis of polysulfates. *Angew. Chem. Int. Ed.* **53**, 9466–9470 (2014); (b) Gao, B. et al. Bifluoride-catalysed sulfur(VI) fluoride exchange reaction for the synthesis of polysulfates and polysulfonates. *Nat. Chem.* **9**, 1083–1088 (2017).

(7) Kim, H. et al. Chain-growth sulfur(VI) fluoride exchange polycondensation: molecular weight control and synthesis of degradable polysulfates. *ACS Cent. Sci.* **7**, 1919−1928 (2021).

(8) Younker, J. M. & Hengge, A. C. A mechanistic study of the alkaline hydrolysis of diaryl sulfate diesters. *J. Org. Chem.* **69**, 9043–9048 (2004).

(9) Simpson, L. S. & Widlanski, T. S. A comprehensive approach to the synthesis of sulfate esters. *J. Am. Chem. Soc.* **128**, 1605–1610 (2006).

(10) Liang, D.-D., Pujari, S. P., Subramaniam, M., Besten, M. & Zuilhof, H. Configurationally chiral SuFEx-based polymers. *Angew. Chem. Int. Ed.* **61**, e2021161 (2022).

(11) Yeom, C.-E., Lee, S. Y., Kim, Y. J. & Kim, B. M. Mild and chemoselective deacetylation method using a catalytic amount of acetyl chloride in methanol. *Synlett* **10**, 1527–1530 (2005).

(12) Hofmann, P. E. et al. Unilaterally fluorinated acenes: synthesis and solid-state properties. *Angew. Chem. Int. Ed.* **59**, 16501–16505 (2020)

(13) Guo, T. et al. A new portal to SuFEx click chemistry: a stable fluorosulfuryl imidazolium salt emerging as an “$F-SO_2^+$” donor of unprecedented reactivity, selectivity, and scope. *Angew. Chem. Int. Ed.* **57**, 2605–2610 (2018).
